# Supplementary material for: Bisulfite-free epigenomics and genomics of single cells through methylation-sensitive restriction
Source: Commun Biol. 2021 Feb 1;4:153. doi: 10.1038/s42003-021-01661-w (PMC7851132; doi:10.1038/s42003-021-01661-w)
Supplement: Supplementary file 2 — Supplementary Information [file 42003_2021_1661_MOESM2_ESM.pdf]

Supplementary Information

Bisulfite-free epigenomics and genomics of single cells through methylation-sensitive restriction

Christoph Niemöller, Julius Wehrle, Julian Riba, Rainer Claus, Nathalie Renz, Janika Rhein, Sabine Bleul, Juliane M. Stosch, Justus Duyster, Christoph Plass, Pavlo Lutsik, Daniel B. Lipka, Michael Lübbert, Heiko Becker

Supplementary Contents

Title	Page
Supplementary Data 1: Unprocessed original images of gels. File names of the images refer to the respective figure the data have been used in.	Separate
Supplementary Data 2: Oligonucleotide sequences, sequencing statistics, number of covered CpGs, number of detected SNVs, ADO rate estimates and unmethylated control detection for single cells analyzed by epi-gSCAR.	Separate
Supplementary Data 3: Visual verification of the single-cell deposition for all single cells analyzed by NGS. A sequence of five images is captured at the ejection nozzle, providing evidence of single-cell deposition: Images A–C show the cell approaching the ejection nozzle. In image D a single cell is detected (inner circle) and the absence of any cells in close vicinity (outer circle) is verified. Image E shows the nozzle after droplet ejection to provide evidence that the detected cell was ejected.	Separate
Supplementary Figure 1: Distribution of HhaI recognition sites in the human genome in comparison to individual CpG methylation measurements of the Infinium HumanMethylation450 and MethylationEPIC BeadChip array across five genomic features.	4-5

Supplementary Figure 2: Agarose gel electrophoresis and Agilent 2100 Bioanalyzer electropherogram analysis of epi-gSCAR library DNA.	6-7
Supplementary Figure 3: A schematic overview of the automated bioinformatic pipeline used for readout of methylation information from epi-gSCAR libraries by NGS.	8-9
Supplementary Figure 4: Averaged methylation profiles of Kasumi-1 epi-gSCAR datasets, Kasumi-1 cell bulk WGBS and 450K array datasets for CGIs, genes grouped into three groups based on their genome-wide RNA expression level in cell-bulk and for five histone marks.	10
Supplementary Figure 5: Digestion rates of methylated and unmethylated spike-in controls as assessed by NGS and step-out PCR for unmethylated spike-in templates and a corresponding Sanger sequencing alignment.	11-12
Supplementary Figure 6: Distribution of covered CpGs from all single cells analyzed by epi-gSCAR across six genomic features.	13
Supplementary Figure 7: Lorenz plot depicting amplification biases for four exemplary epi-gSCAR datasets, two MALBAC datasets, one multiple displacement amplification control and two non-amplified gDNA controls.	14
Supplementary Figure 8: Evaluation of poly(d)A tail length for tailed scar reads in epi-gSCAR libraries of single cells K_01 - K_07 analyzed by NGS.	15-16
Supplementary Figure 9: Number of CpGs for union and intersect of all possible combinations of individual epi-gSCAR datasets for Kasumi-1 and OCI-AML3.	17-18

Supplementary Figure 10: Allelic dropout rate estimates in epi-gSCAR datasets for Kasumi-1 and OCI-AML3 single cells.	19
Supplementary Figure 11: Allelic dropout rate estimates for single cells K_01 – K_07.	20
Supplementary Figure 12: Exemplary Sanger sequencing chromatograms of the *KIT*, *TP53* and *DNMT3a* gene mutations in individual Kasumi-1 and OCI-AML3 epi-gSCAR libraries.	21

Supplementary Figure 1

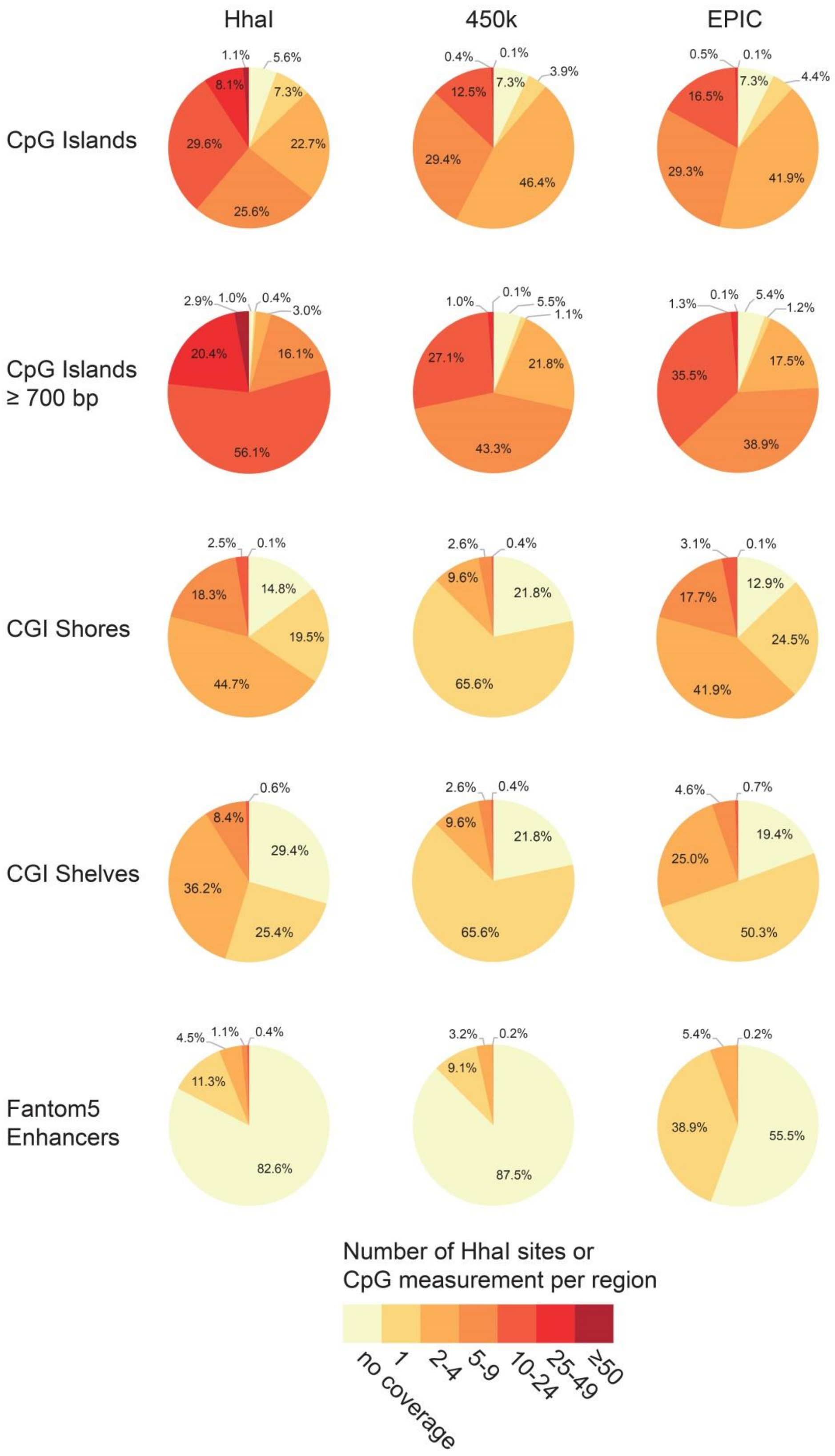

Supplementary Figure 1: Distribution of HhaI recognition sites in the human genome in comparison to individual CpG methylation measurements of the Infinium

HumanMethylation450 and MethylationEPIC BeadChip array across five genomic features. Pie charts show the numbers of HhaI sites or the numbers of individual CpG measurements in each genomic feature by incremental colors. The human genome contains 1.69 million HhaI recognition sites which can potentially be covered by epi-gSCAR as compared to over 450,000 and over 850,000 cytosine positions covered by the HumanMethylation450 BeadChip and MethylationEPIC Kit array, respectively (Pidsley, R. et al. Genome Biol. 17, 1–17 (2016)). Of all CGIs in the human genome, 94.4% contain HhaI sites and 42.4% of all CGIs contain more than 5 sites. When only CGIs ≥ 700 bp are analyzed this coverage increases to 99% and 95.6%, respectively. For CGI shore and shelves, 85.2% and 70.6% contain at least one HhaI site, respectively. With the focus on Fantom5 enhancers, coverage for HhaI (17.4%) is higher than for 450K array (12.5%), but lower as compared to EPIC array (44.5%). CGI shores are defined as 2 kb up- and downstream of CGIs (UCSC) while CGI shelves are defined as 2kb flanking on either side from CGI shores. For the analysis of enhancer coverage, we used the set of permissive enhancers from FANTOM5 (phase 1 and 2).

Supplementary Figure 2

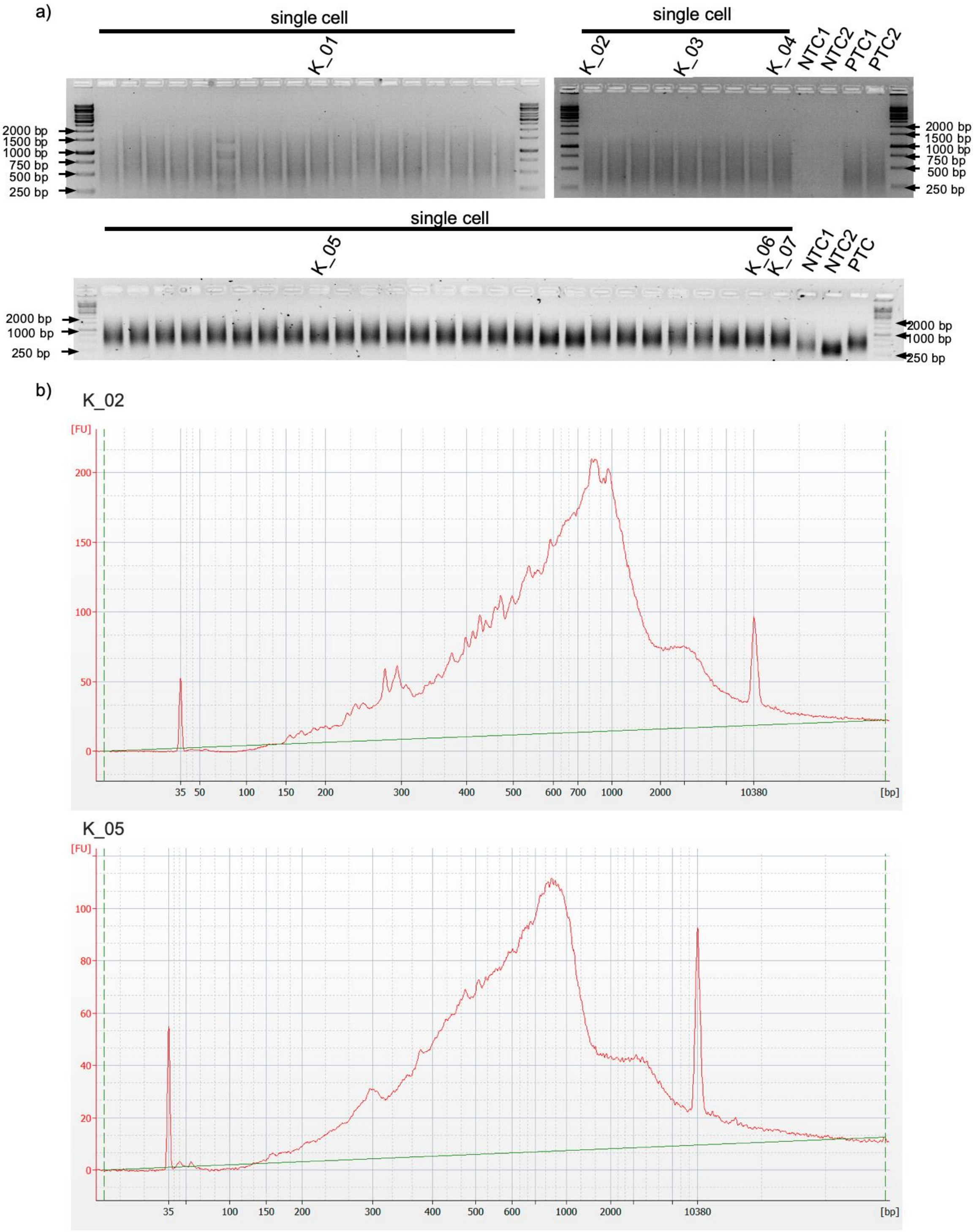

Supplementary Figure 2: (a) Agarose gel electrophoresis of library DNA from 27 single cells, each for assay variant A and B (top and bottom) shows successful amplification

for all single cells subjected to epi-gSCAR. Amplicons show a size distribution between 100 and 2500 bp. No template-controls (NTC) with wells not containing a single cell were used to control for contamination and positive controls (PTC) containing 30 pg of Kasumi-1 gDNA were used to control for amplification success. Template-independent products of low molecular weight were only found in NTCs for assay variant B. Single cells, which have been selected for analysis by NGS, are denoted as K_01 - K_07. (b) Example Agilent 2100 Bioanalyzer electropherogram results for single cell K_02 and K_05 show highly similar size distribution profiles.

Supplementary Figure 3

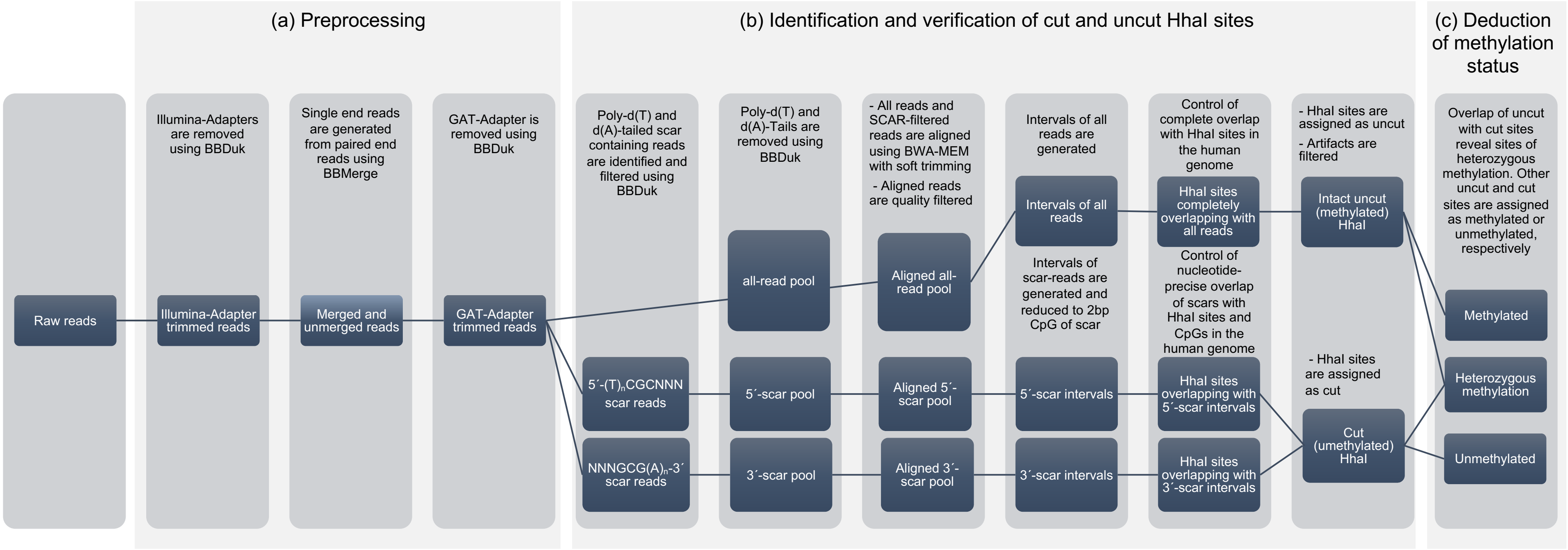

Supplementary Figure 3: A schematic overview of the automated bioinformatic pipeline used for readout of methylation information from epi-gSCAR libraries by NGS. (a) Preprocessing: Trimming of Illumina adapters is followed by merging of paired-end reads and GAT-adapter sequence trimming. (b) Identification and verification of cut and uncut HhaI sites: Pre-processed reads are filtered for reads containing 5´ poly(d)T or 3´ poly(d)A tailed HhaI scars separately followed by poly(d)T and poly(d)A trimming. 5´ poly(d)T and 3´ poly(d)A tails of GAT-adapter trimmed reads are removed analogously. The generated files containing all reads (all-read file), 5´-scar reads (5'-scar file) and 3´-scar reads (3'-scar file) are aligned to the human reference genome (version GRCh37/hg19) and alignments are quality-

filtered. CpG intervals (5´-CG and CG-3´) are generated, controlled for nucleotide-precise overlap with HhaI and CpGs in the human genome and assigned as cut HhaI sites. HhaI sites in the human genome, which are completely covered by reads of the all-read file, are assigned as uncut HhaI sites. (c) Deduction of methylation status: Overlap of uncut with cut sites revealed sites of heterozygous methylation. All other uncut and cut HhaI sites were assigned as methylated or unmethylated, respectively.

Supplementary Figure 4

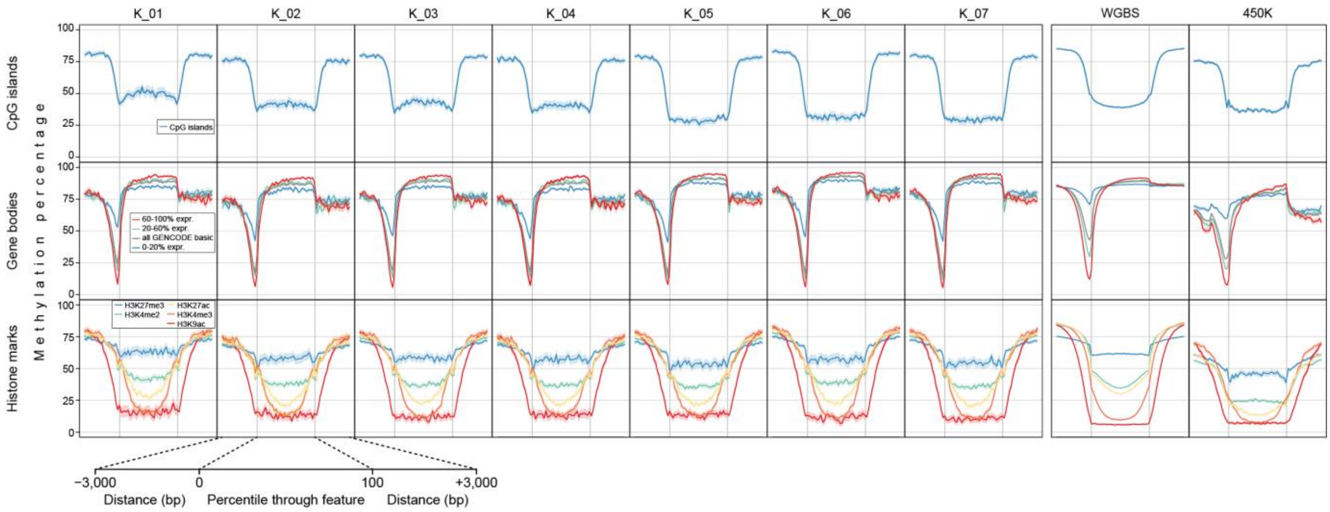

Supplementary Figure 4: Averaged methylation profiles of single cell K_01 - K_07, Kasumi-1 cell bulk WGBS and 450K array. Shown are profiles for CGIs (blue line), genes grouped into three groups based on their genome-wide RNA expression level in cell-bulk (0-20%, blue line; 20-60%, green line; 60-100%, red line; all GENCODE basic, grey line) as FPKM (fragments per million mapped reads per kilobase exon) and for five histone marks (H3K27me3, blue line; H3K4me2, green line; H3K27ac, yellow line; H3K4me3, orange line; H3K9ac, red line). Shown is the mean methylation across 150bp-windows for each feature set and 3kb up- and downstream.

Supplementary Figure 5

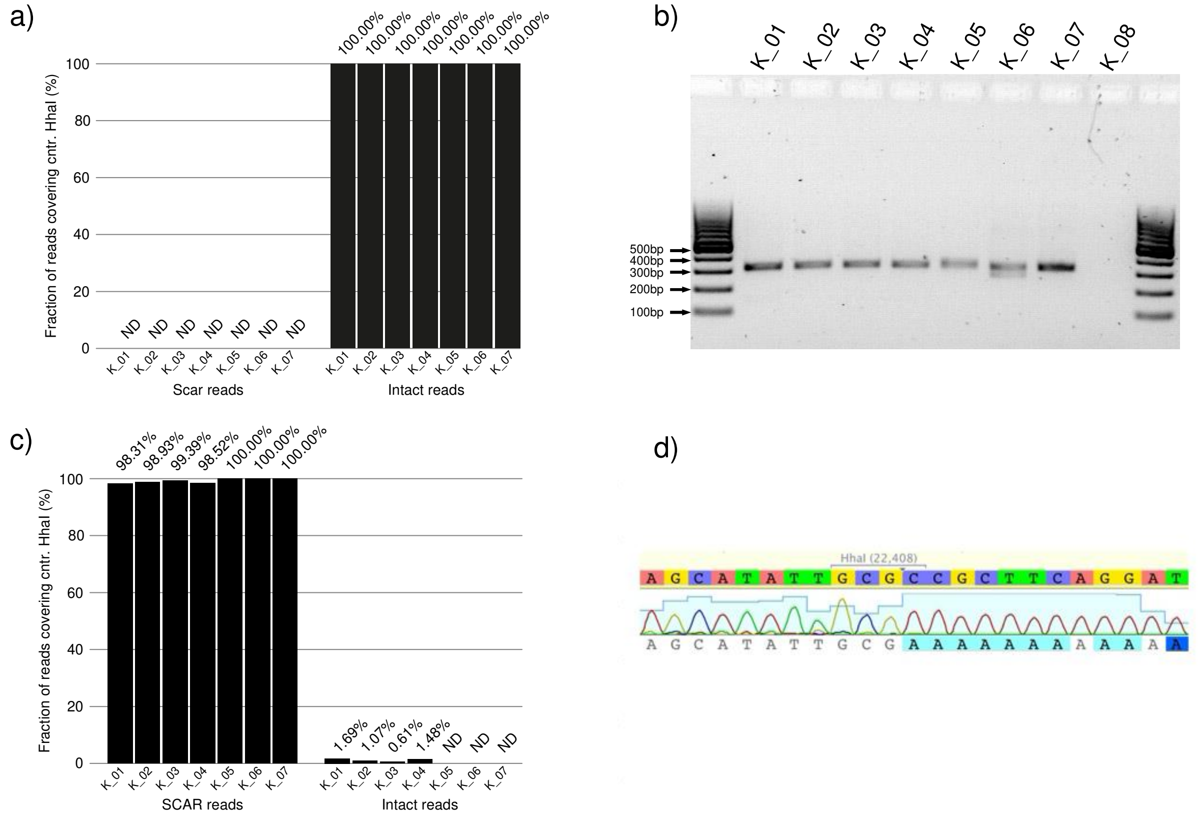

Supplementary Figure 5: Digestion rates of methylated and unmethylated spike-in controls as assessed by NGS and step-out PCR for unmethylated spike-in templates and exemplary corresponding Sanger sequencing alignment for single cell K_07. In order to control for digestion efficiency, methylated and unmethylated exogenous control DNA was spiked into each single-cell reaction. Each control amplicon contains one HhaI site (see method section). Of each amplicon 30 ag (assay variant A, single cell K_01 – K_04) or 3 ag (assay variant B, single cell K_05 - K_07) was spiked in. This corresponds to approximately 110 or 11 and 60 or 6 molecules of unmethylated and methylated control amplicons, respectively. (a) Fraction of scar and intact reads covering the methylated spike-in control HhaI site for single cell K_01 - K_07. The all-read file was filtered for reads matching the methylated control amplicon and aligned to the enterobacteria phage lambda reference genome. Then, local coverage of intact and scar reads at the control HhaI site was calculated and displayed as a fraction of all reads at this position. (b) Analysis of unmethylated spike-in controls for single cell K_01 - K_07 by step-out PCR. For all single cells, only scar reads could be detected via step-out PCR. (c) Fraction of scar and intact reads covering the unmethylated spike-in control HhaI site. The all-read file was filtered for reads matching the

unmethylated control amplicon and aligned to the enterobacteria phage lambda reference genome and digestion efficiency was calculated as described above. (d) Exemplary Sanger sequencing result confirming that only scar amplicons could be detected by step-out PCR. The alignment shows the reference sequence (top) and the aligned Sanger chromatogram obtained from single cell K_07. ND, not detectable; NTC, no template control.

Supplementary Figure 6

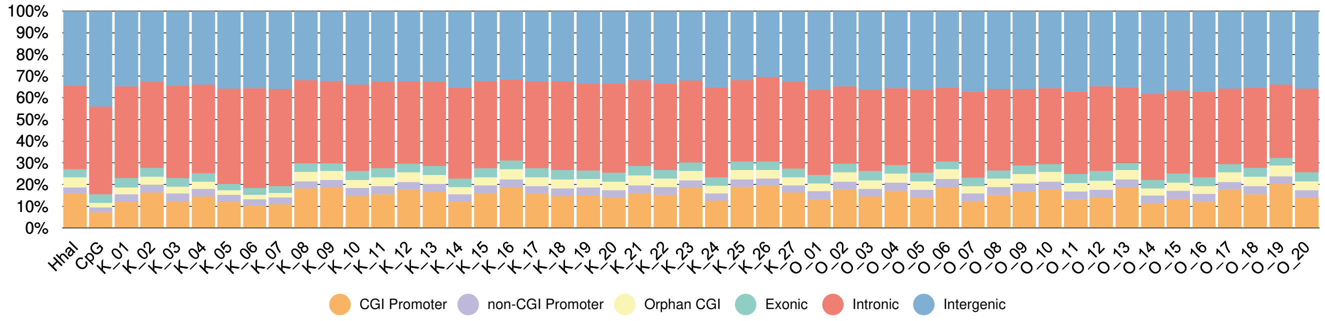

Supplementary Figure 6: Distribution of covered CpGs from all single cells analyzed by epi-gSCAR across six genomic features. Genomic coordinates of these features are based on the human assembly GRCh37 (hg19) and obtained as follows: Promoters are defined as 2 kb flanking the transcription start sites of genes (gencode basic). CGI promoters (orange) were obtained by merging intervals of promoters with overlapping CGIs (UCSC) while the residual promoters are defined as non-CGI promoters (violet). CGIs not overlapping with any promoter regions were defined as orphan CGIs (yellow). Exons (green) and introns (red) are obtained by subtracting CGI promoters and non-CGI promoters from gene exons and introns. All other regions are defined as intergenic (blue).

Supplementary Figure 7

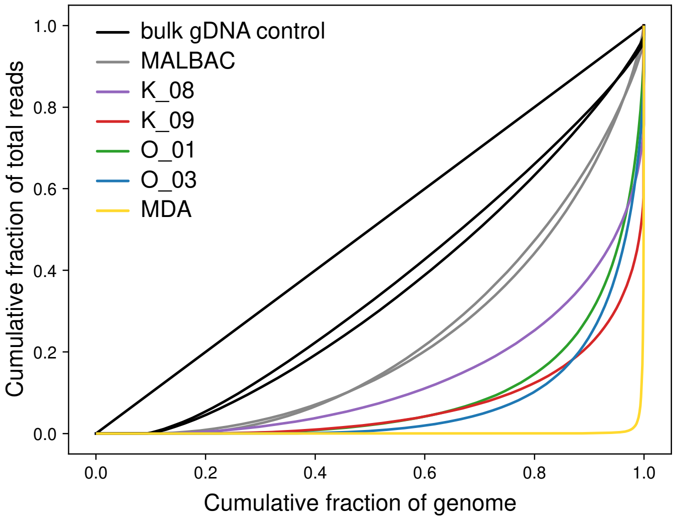

Supplementary Figure 7: Lorenz plot depicting amplification biases in 10 kb windows for four exemplary single cells analyzed by epi-gSCAR (K_08, violet line; K_09, red line; O_01, green line; O_03, blue line), two MALBAC datasets (grey line) downsampled to comparable number of reads per single cell (19,419,823, 23,573,394 and 20,594,112 reads), one multiple displacement amplification (MDA) control (yellow line) and two non-amplified gDNA controls (black line). The diagonal line indicates perfectly uniform coverage while biased coverage results in deviations from the diagonal.

Supplementary Figure 8

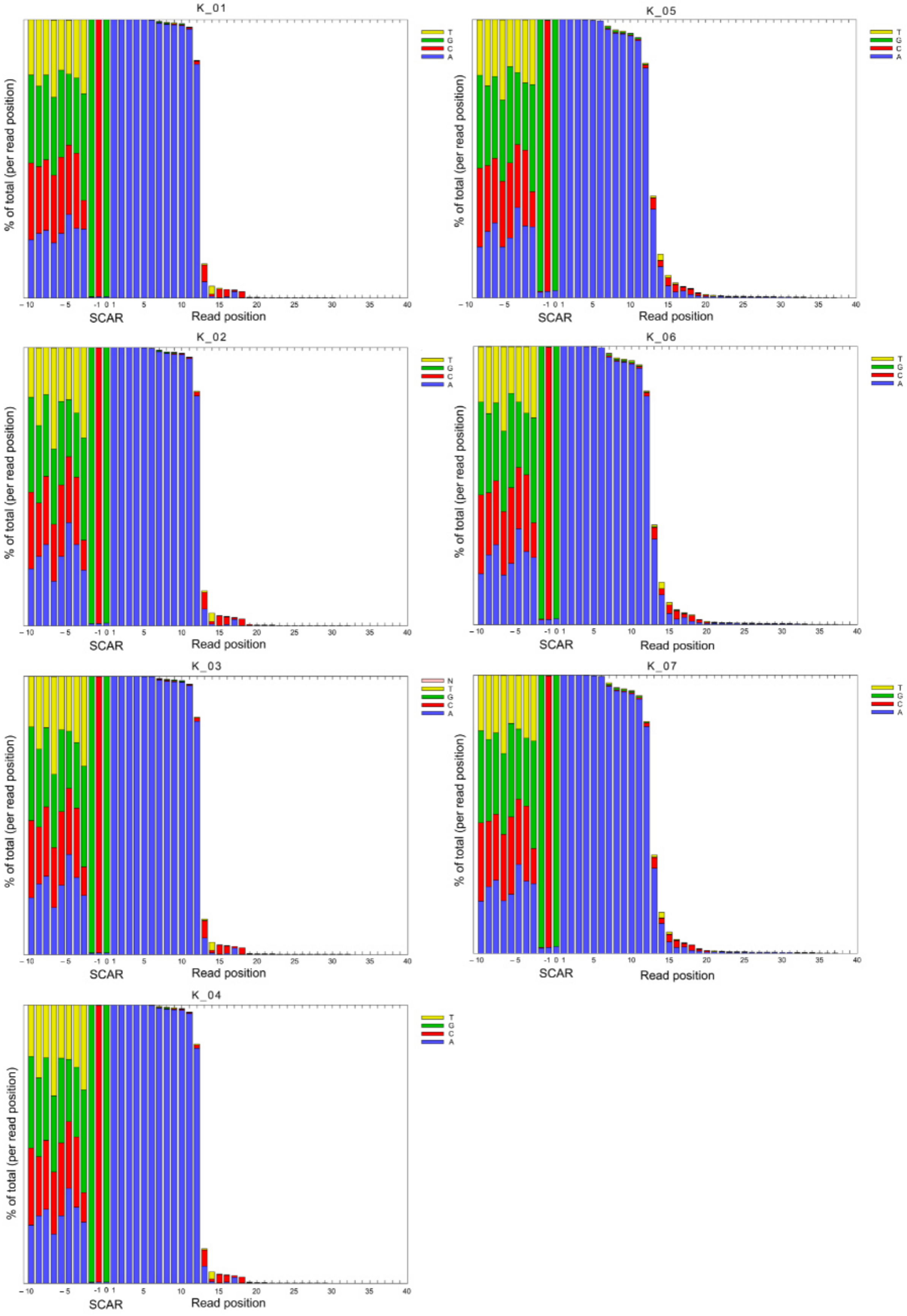

Supplementary Figure 8: Evaluation of poly(d)A tail length for tailed scar reads in epi-gSCAR libraries of single cells K_01 - K_07 analyzed by NGS. Reads of the 3´ poly(d)A tailed HhaI scar read file (Supplementary Figure 3) were filtered for the motif GCGAAAAAA with high stringency (hamming distance = 0) and trimmed 10 bp in 5´ and 40 bp in 3´ direction relative to the last scar position. The stacked histogram represents the proportion of each nucleotide at a given read position (T, yellow; G, green; C, red; A, blue). The length of poly(d)A-tails was successfully limited to 12 nucleotides with the use of anchored adapters (single cell K_01 - K_04). Using non-anchored adapters (K_05 - K_07) resulted only in a slight increase of the poly(d)A-tail length (up to approximately 16 nucleotides).

Supplementary Figure 9

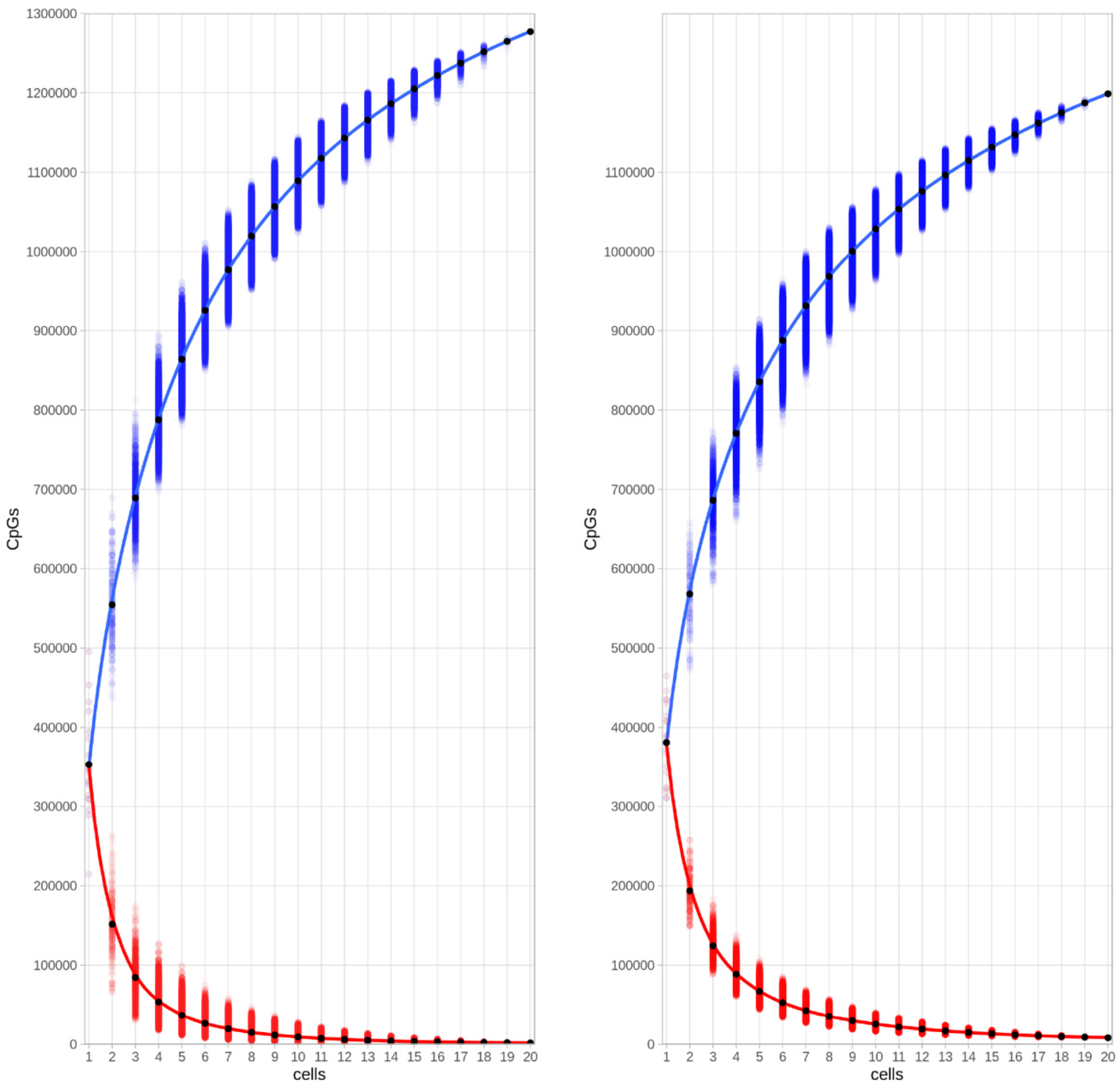

Supplementary Figure 9: Number of CpGs for union (red) and intersect (blue) of all possible combinations of individual epi-gSCAR datasets (2 to 20 single cells) for Kasumi-1 (left) and OCI-AML3 (right). The intersect shows that the total number of covered CpGs in multiple epi-gSCAR libraries increases with the number of analyzed cells. Conversely, in union the number of CpGs common to multiple epi-gSCAR libraries decreases when the number of analyzed datasets is increased. The Kasumi-1 and OCI-AML3 pseudo-bulk datasets contain the mean beta values for each CpG position covered in at least two single cell datasets and single cell beta values if only covered by one single cell. Accordingly, the pseudobulk datasets are composed of

mean values of 2-20 single cell beta values and single cell beta values (combination of 20 single cells in intersect).

Supplementary Figure 10

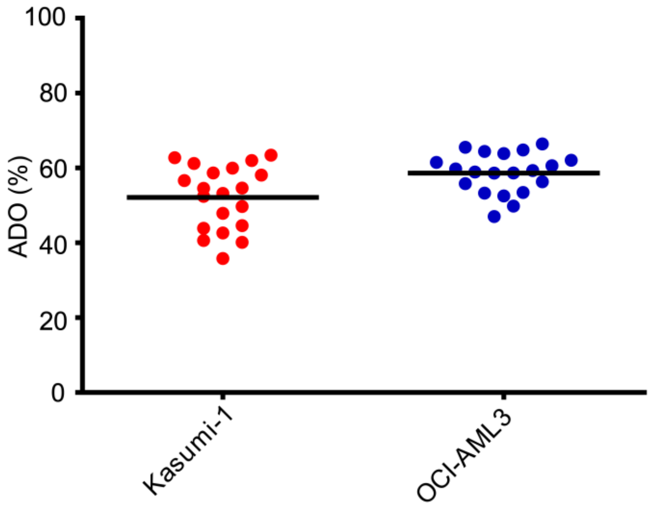

Supplementary Figure 10: Allelic dropout (ADO) rate estimates for K_08 – K_27 (red dots) and O_01 – O_20 (blue dots) calculated as the fraction of SNPs called as heterozygous in bulk DNA sample (Genome-Wide Human SNP Array 6.0) although called homozygous in single cells for loci with coverage ≥ 20x.

Supplementary Figure 11

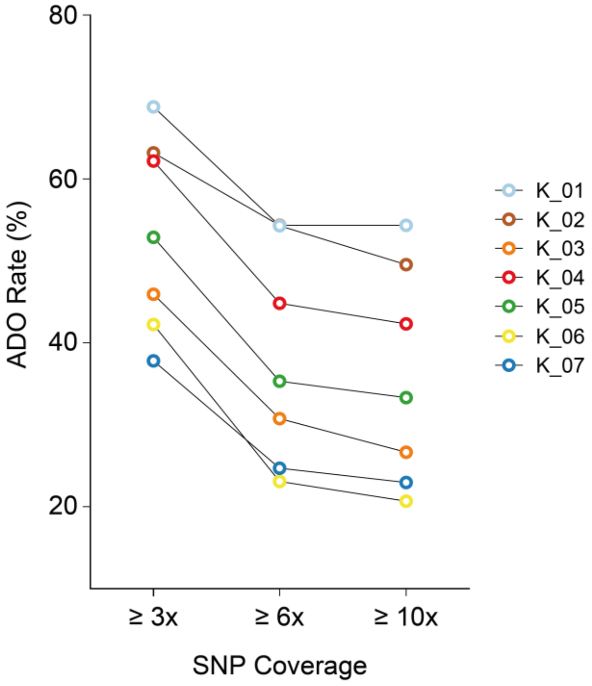

Supplementary Figure 11: Allelic dropout (ADO) rate estimates for single cells K_01 – K_07 (K_01, light blue circles; K_02, brown circles; K_03, orange circles; K_04, red circles; K_05, green circles; K_06, yellow circles; K_07, blue circles) calculated as the fraction of SNPs called as heterozygous in bulk DNA sample (Cytoscan 750K array; VAF=0.45-0.55) though called homozygous in single cells for loci with ≥ 3x, ≥ 6x and ≥ 10x. ADO rate decreased when minimal locus coverage is increased.

Supplementary Figure 12

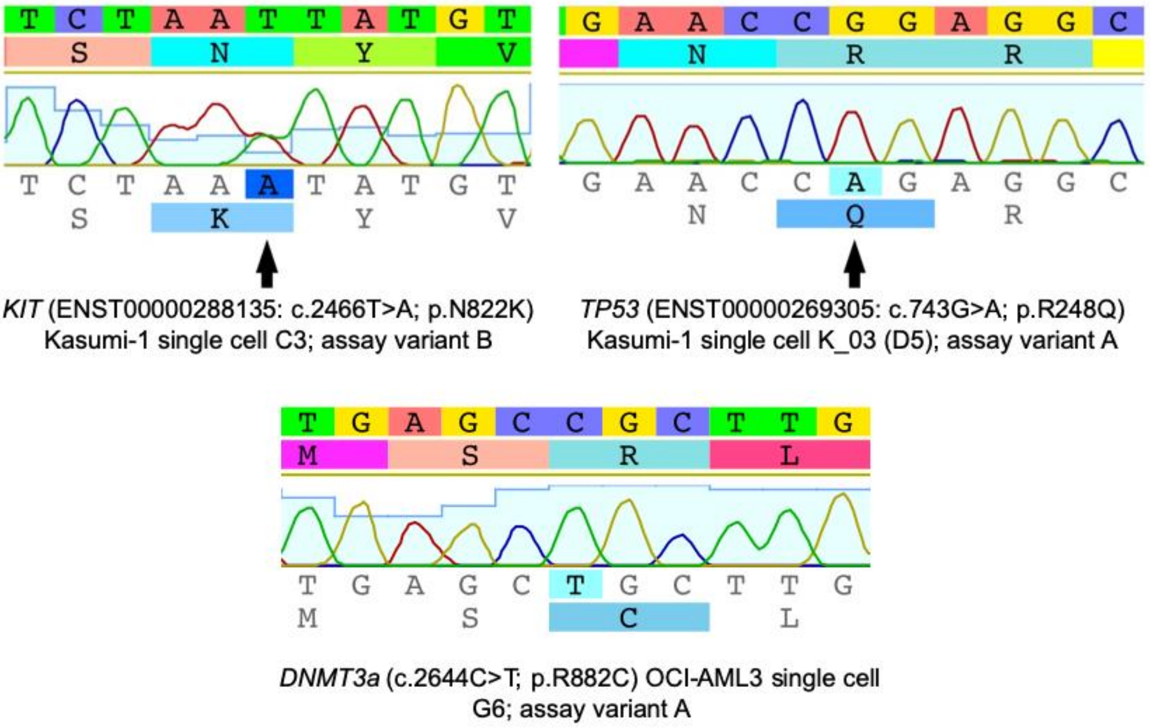

Supplementary Figure 12: Exemplary Sanger sequencing chromatograms of the *KIT* (c.2466T>A; p.N822K; Kasumi-1 single cell C3), *TP53* (c.743G>A; p.R248Q; Kasumi-1 single cell D5) and *DNMT3a* (c.2644C>T p.R882C; OCI-AML3 single cell G6) gene mutations in individual epi-gSCAR libraries. *KIT* and *DNMT3a* were analyzed by regular PCR and *TP53* was analyzed by step-out PCR.
