# Supplementary material for: Bisulfite-free epigenomics and genomics of single cells through methylation-sensitive restriction
Source: Commun Biol. 2021 Feb 1;4:153. doi: 10.1038/s42003-021-01661-w (PMC7851132; doi:10.1038/s42003-021-01661-w)
Supplement: Supplementary file 3 — Description of Additional Supplementary Files [file 42003_2021_1661_MOESM3_ESM.pdf]

Description of Additional Supplementary Files

File name: Supplementary Data 1

Description: Unprocessed original images of gels. File names of the images refer to the respective figure the data have been used in.

File name: Supplementary Data 2

Description: Oligonucleotide sequences, sequencing statistics, number of covered CpGs, number of detected SNVs, ADO rate estimates and unmethylated control detection for single cells analysed by epi-gSCAR.

File name: Supplementary Data 3

Description: Visual verification of the single-cell deposition for all single cells analyzed by NGS. A sequence of five images is captured at the ejection nozzle, providing evidence of single-cell deposition: Images A–C show the cell approaching the ejection nozzle. In image D a single cell is detected (inner circle) and the absence of any cells in close vicinity (outer circle) is verified. Image E shows the nozzle after droplet ejection to provide evidence that the detected cell was ejected.
