# Supplementary figures and images for: Bisulfite-free epigenomics and genomics of single cells through methylation-sensitive restriction (part 1 of 3)
Source: Commun Biol. 2021 Feb 1;4:153. doi: 10.1038/s42003-021-01661-w (PMC7851132; doi:10.1038/s42003-021-01661-w)

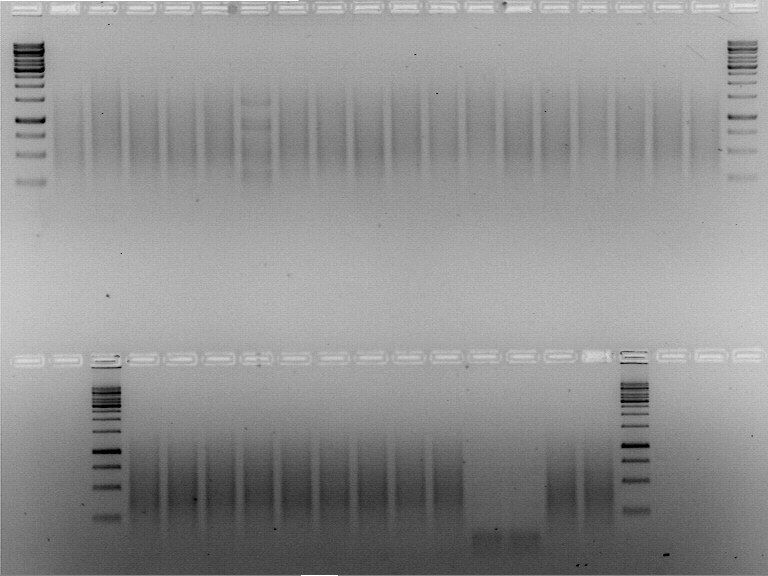

Supplement: Supplementary file 4 — Supplementary Data 1 [file 42003_2021_1661_MOESM4_ESM.zip › Supplementary Data 1/Supplementary_Figure_2a_1.JPG]

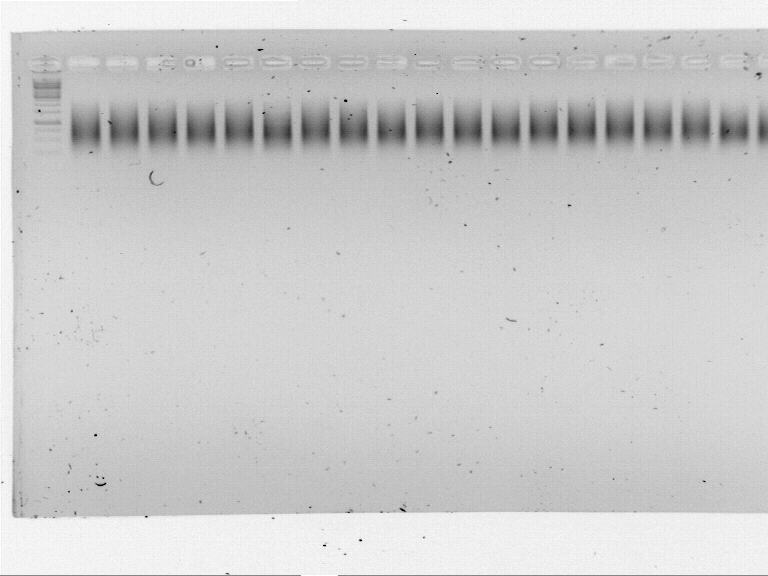

Supplement: Supplementary file 4 — Supplementary Data 1 [file 42003_2021_1661_MOESM4_ESM.zip › Supplementary Data 1/Supplementary_Figure_2a_2.JPG]

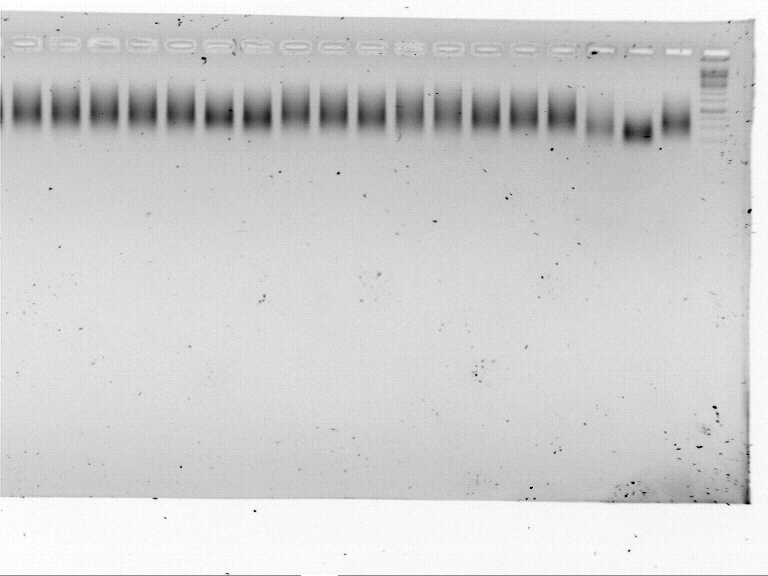

Supplement: Supplementary file 4 — Supplementary Data 1 [file 42003_2021_1661_MOESM4_ESM.zip › Supplementary Data 1/Supplementary_Figure_2a_3.JPG]

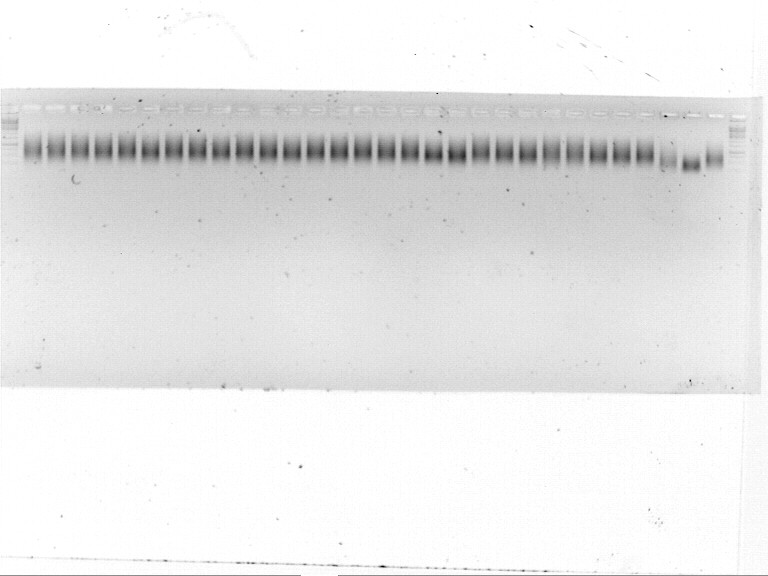

Supplement: Supplementary file 4 — Supplementary Data 1 [file 42003_2021_1661_MOESM4_ESM.zip › Supplementary Data 1/Supplementary_Figure_2a_4.JPG]

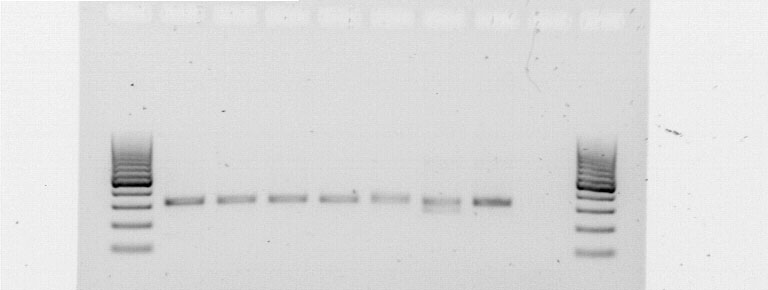

Supplement: Supplementary file 4 — Supplementary Data 1 [file 42003_2021_1661_MOESM4_ESM.zip › Supplementary Data 1/Supplementary_Figure_5b_1.JPG]

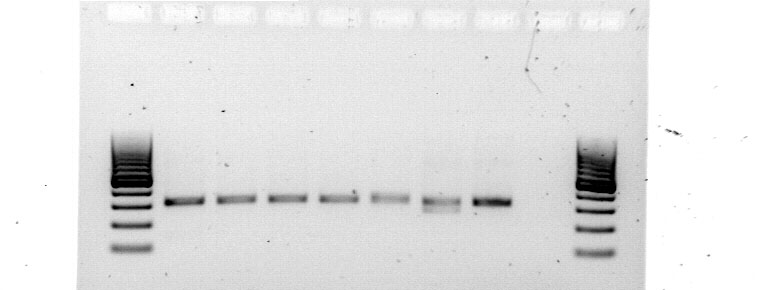

Supplement: Supplementary file 4 — Supplementary Data 1 [file 42003_2021_1661_MOESM4_ESM.zip › Supplementary Data 1/Supplementary_Figure_5b_2.jpg]

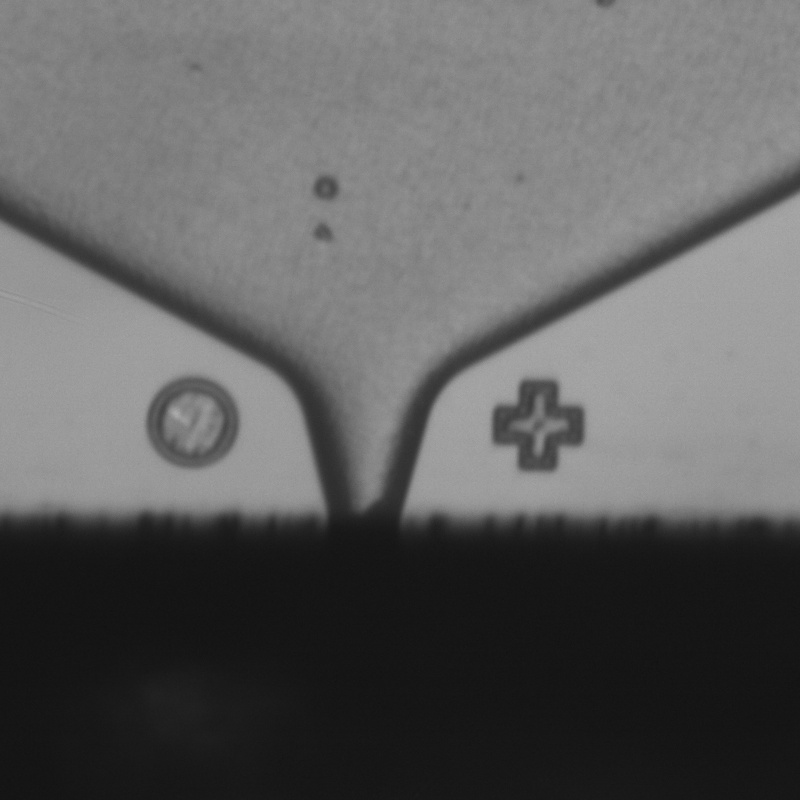

Supplement: Supplementary file 6 — Supplementary Data 3 [file 42003_2021_1661_MOESM6_ESM.zip › Supplementary Data 3 corrected/K_21_A.jpg]

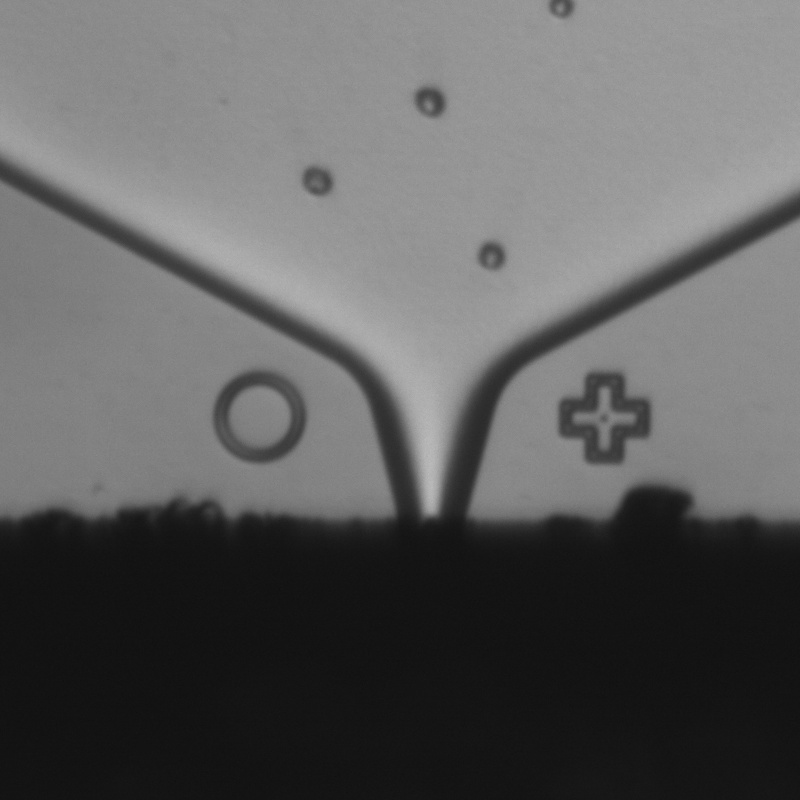

Supplement: Supplementary file 6 — Supplementary Data 3 [file 42003_2021_1661_MOESM6_ESM.zip › Supplementary Data 3 corrected/K_07_B.jpg]

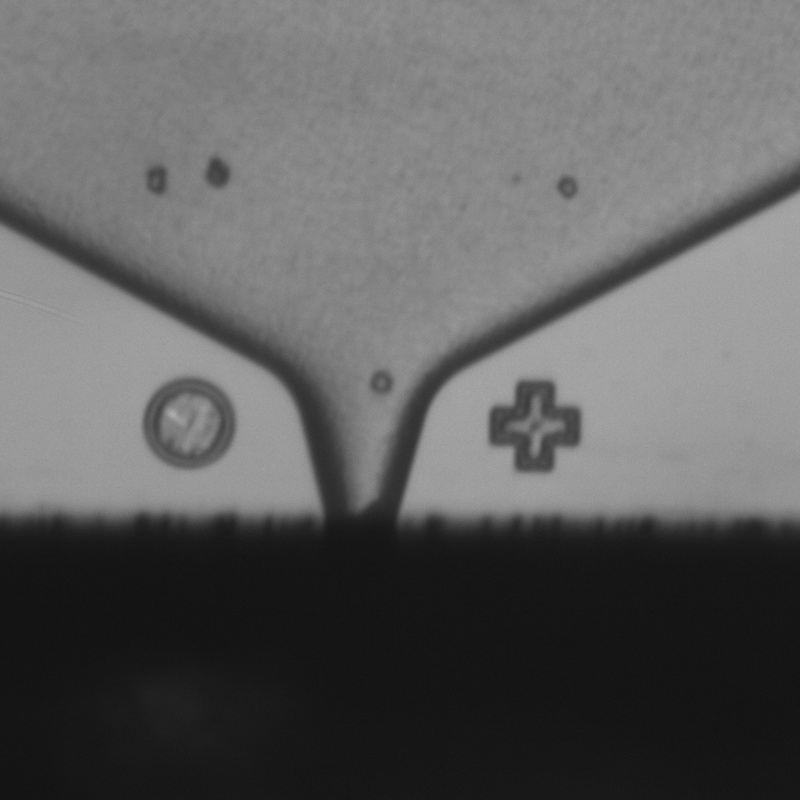

Supplement: Supplementary file 6 — Supplementary Data 3 [file 42003_2021_1661_MOESM6_ESM.zip › Supplementary Data 3 corrected/K_23_C.jpg]

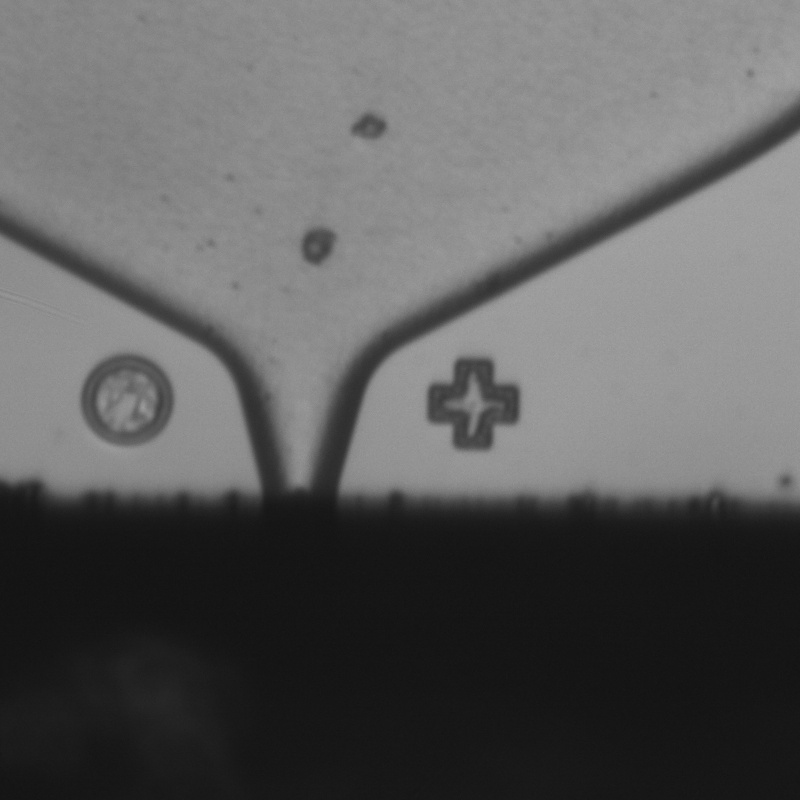

Supplement: Supplementary file 6 — Supplementary Data 3 [file 42003_2021_1661_MOESM6_ESM.zip › Supplementary Data 3 corrected/O_14_B.jpg]

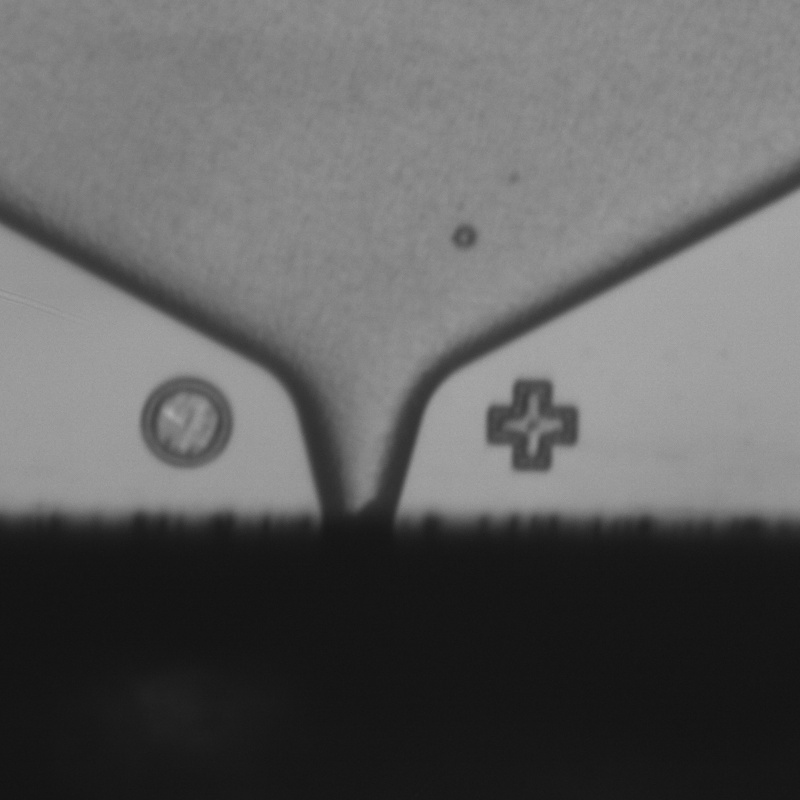

Supplement: Supplementary file 6 — Supplementary Data 3 [file 42003_2021_1661_MOESM6_ESM.zip › Supplementary Data 3 corrected/K_18_E.jpg]

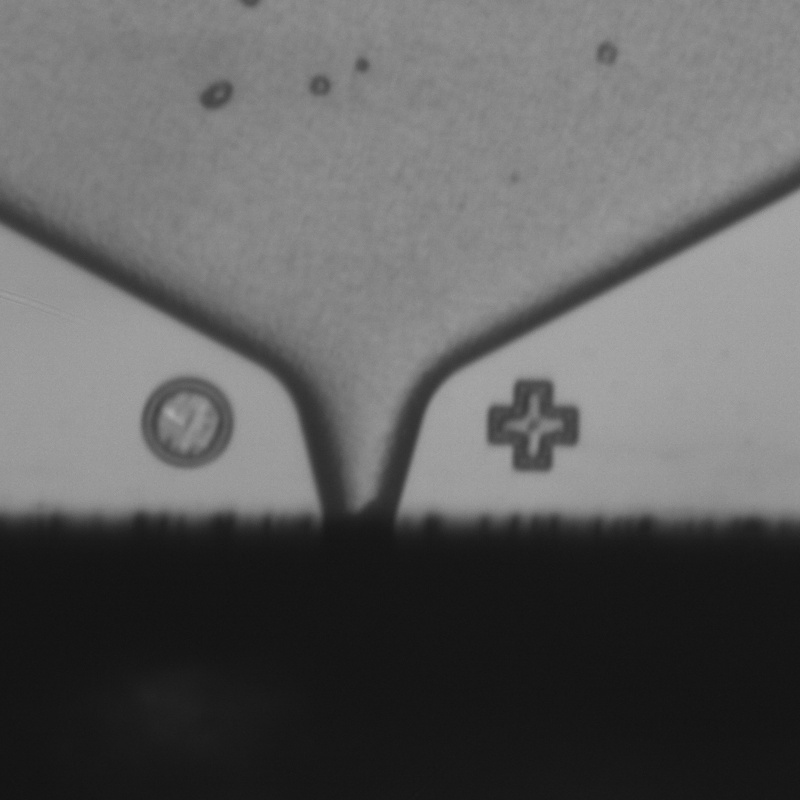

Supplement: Supplementary file 6 — Supplementary Data 3 [file 42003_2021_1661_MOESM6_ESM.zip › Supplementary Data 3 corrected/K_25_E.jpg]

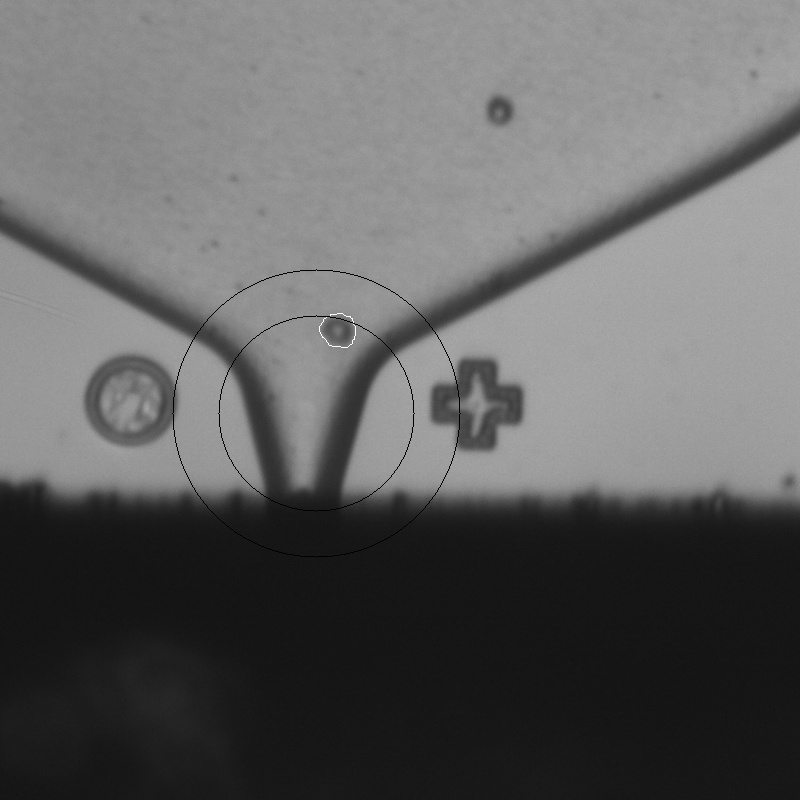

Supplement: Supplementary file 6 — Supplementary Data 3 [file 42003_2021_1661_MOESM6_ESM.zip › Supplementary Data 3 corrected/O_12_D.jpg]

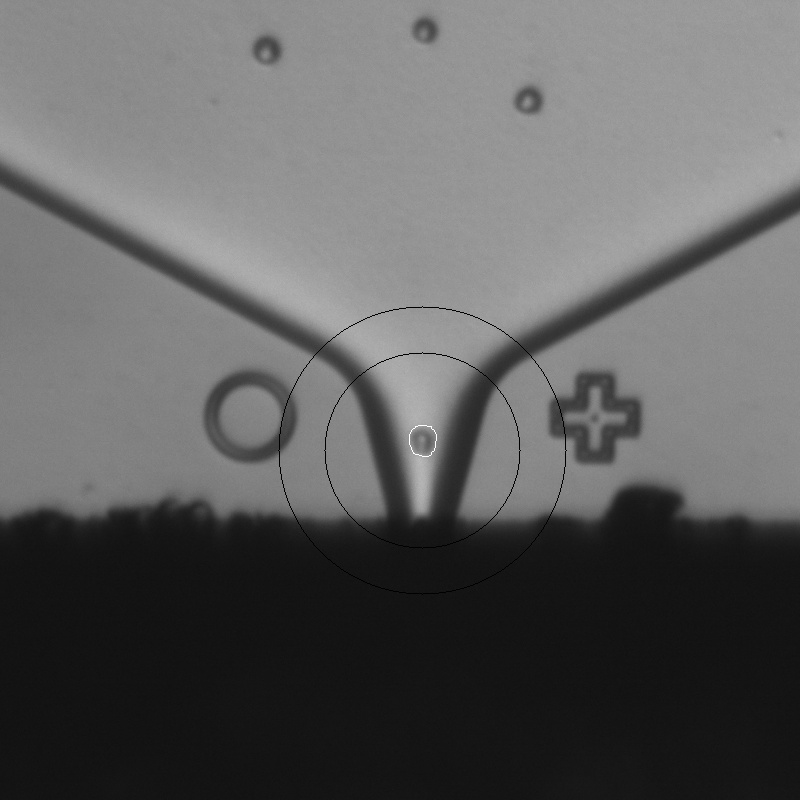

Supplement: Supplementary file 6 — Supplementary Data 3 [file 42003_2021_1661_MOESM6_ESM.zip › Supplementary Data 3 corrected/K_01_D.jpg]

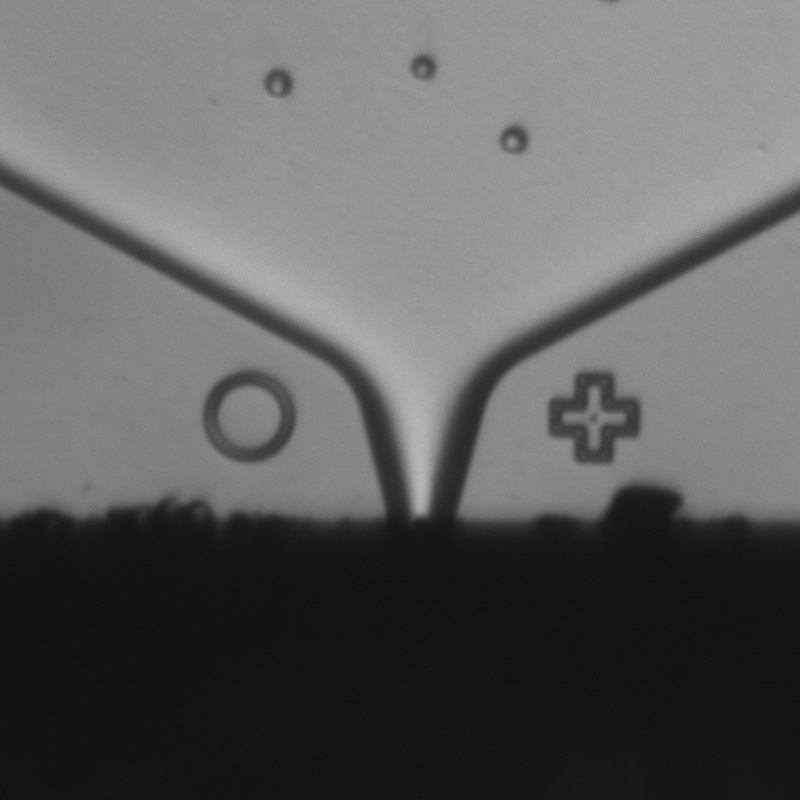

Supplement: Supplementary file 6 — Supplementary Data 3 [file 42003_2021_1661_MOESM6_ESM.zip › Supplementary Data 3 corrected/K_01_E.jpg]

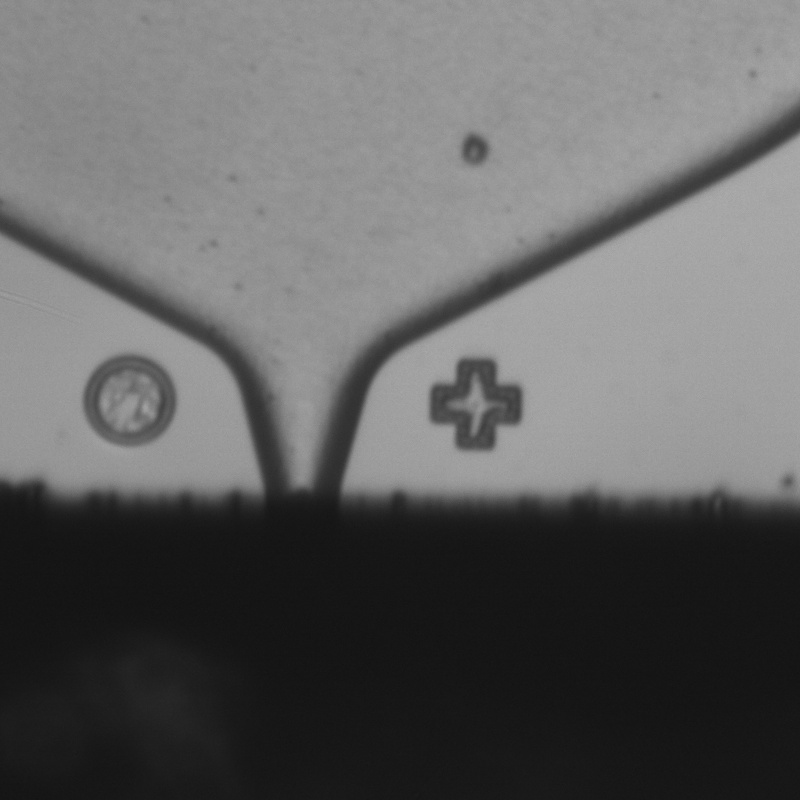

Supplement: Supplementary file 6 — Supplementary Data 3 [file 42003_2021_1661_MOESM6_ESM.zip › Supplementary Data 3 corrected/O_12_E.jpg]

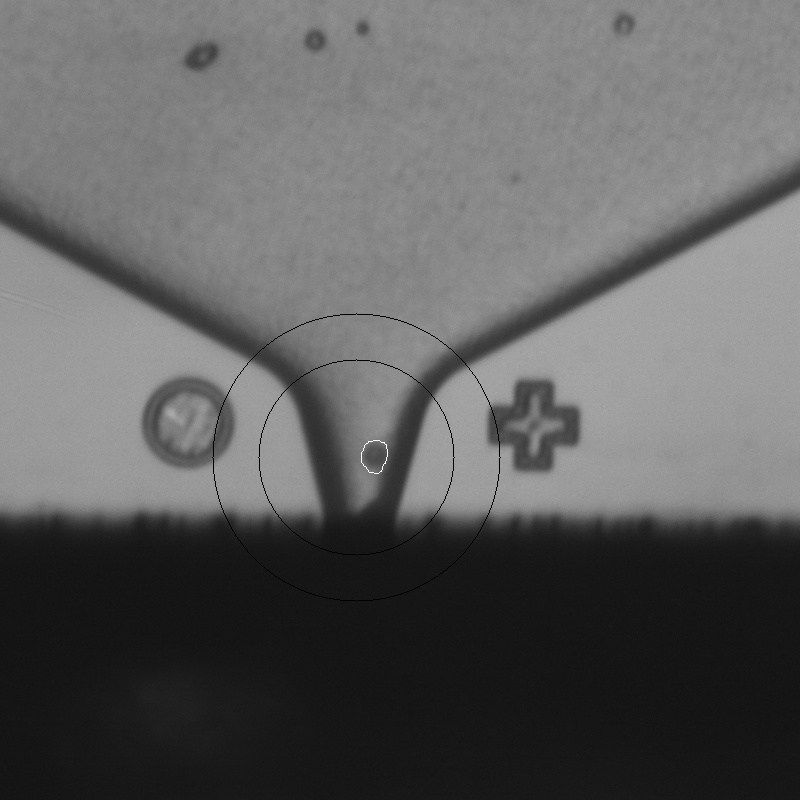

Supplement: Supplementary file 6 — Supplementary Data 3 [file 42003_2021_1661_MOESM6_ESM.zip › Supplementary Data 3 corrected/K_25_D.jpg]

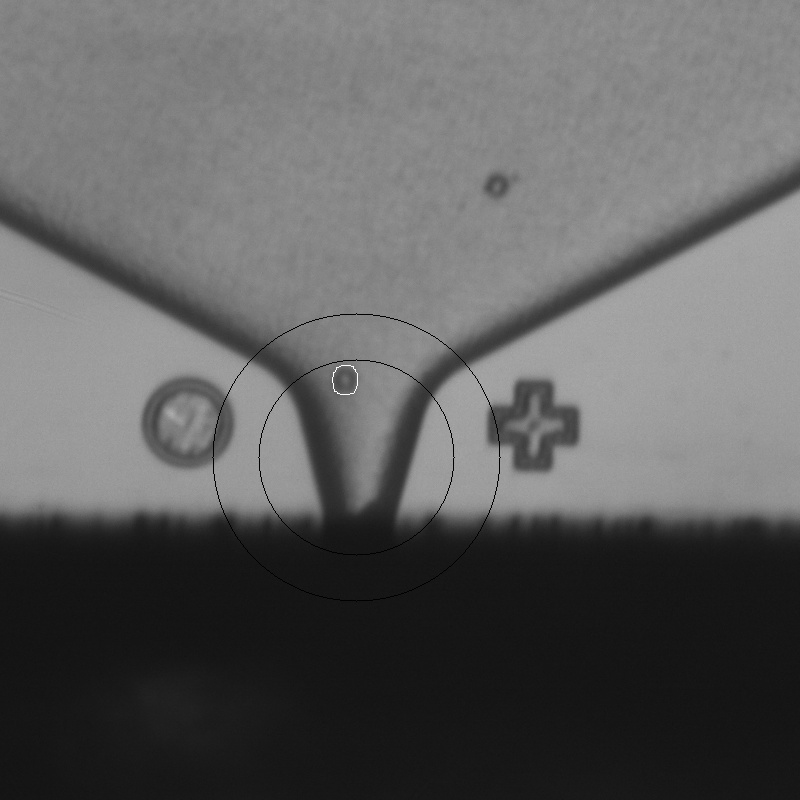

Supplement: Supplementary file 6 — Supplementary Data 3 [file 42003_2021_1661_MOESM6_ESM.zip › Supplementary Data 3 corrected/K_18_D.jpg]

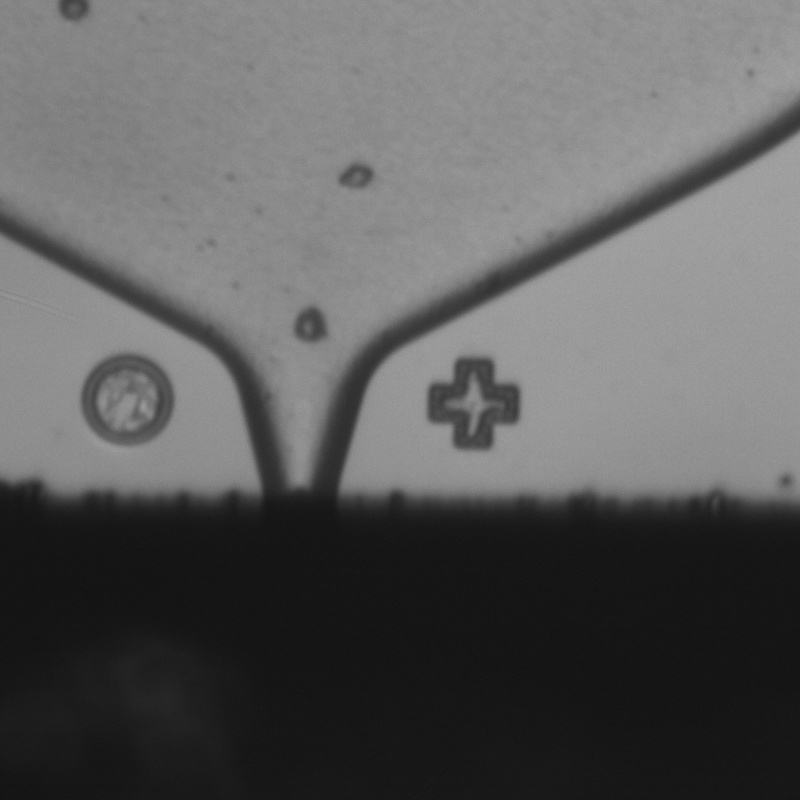

Supplement: Supplementary file 6 — Supplementary Data 3 [file 42003_2021_1661_MOESM6_ESM.zip › Supplementary Data 3 corrected/O_14_C.jpg]

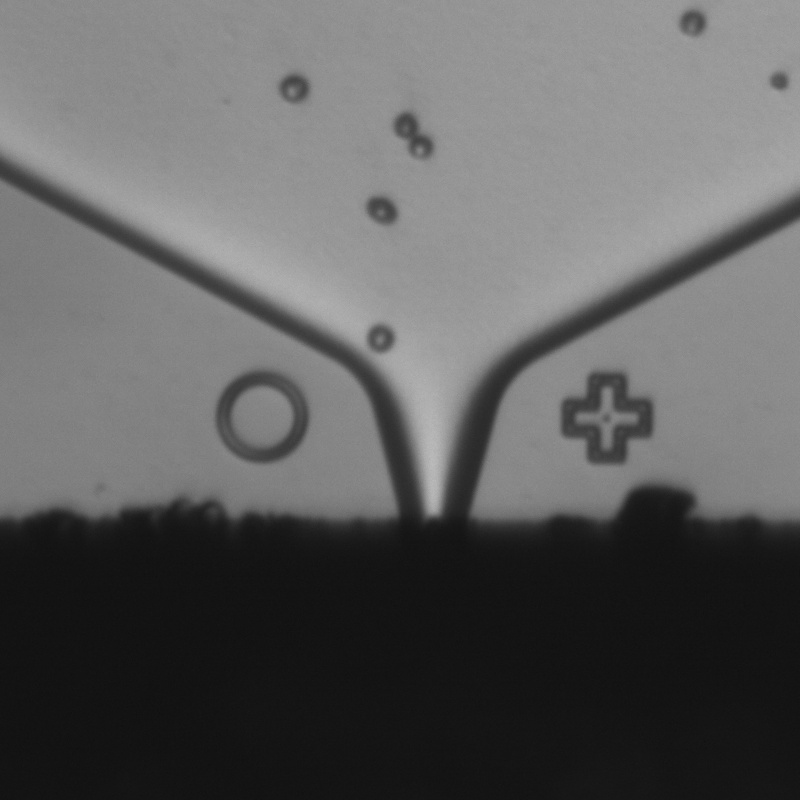

Supplement: Supplementary file 6 — Supplementary Data 3 [file 42003_2021_1661_MOESM6_ESM.zip › Supplementary Data 3 corrected/K_05_A.jpg]

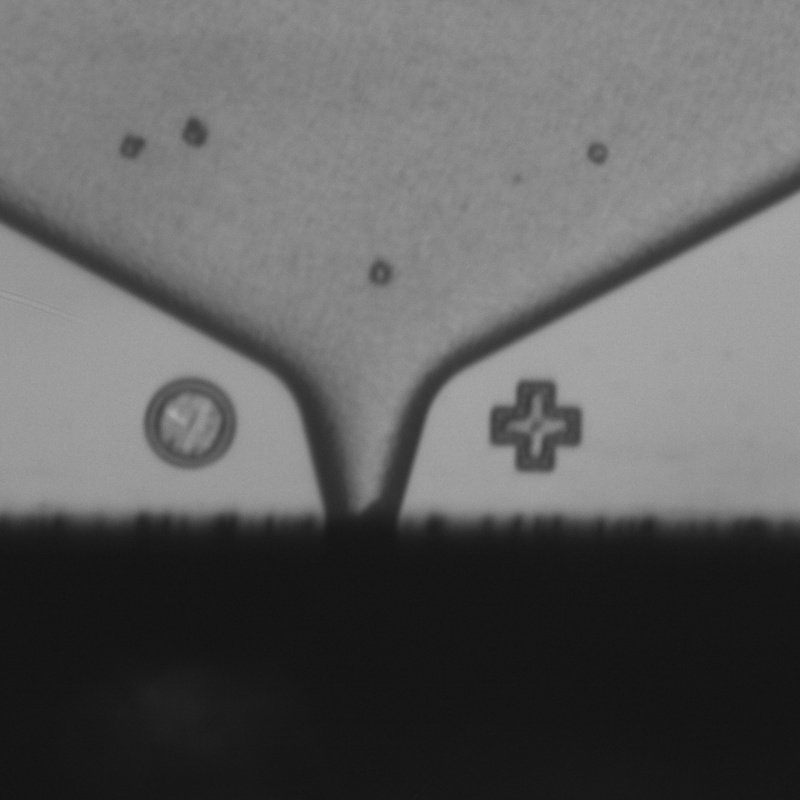

Supplement: Supplementary file 6 — Supplementary Data 3 [file 42003_2021_1661_MOESM6_ESM.zip › Supplementary Data 3 corrected/K_23_B.jpg]

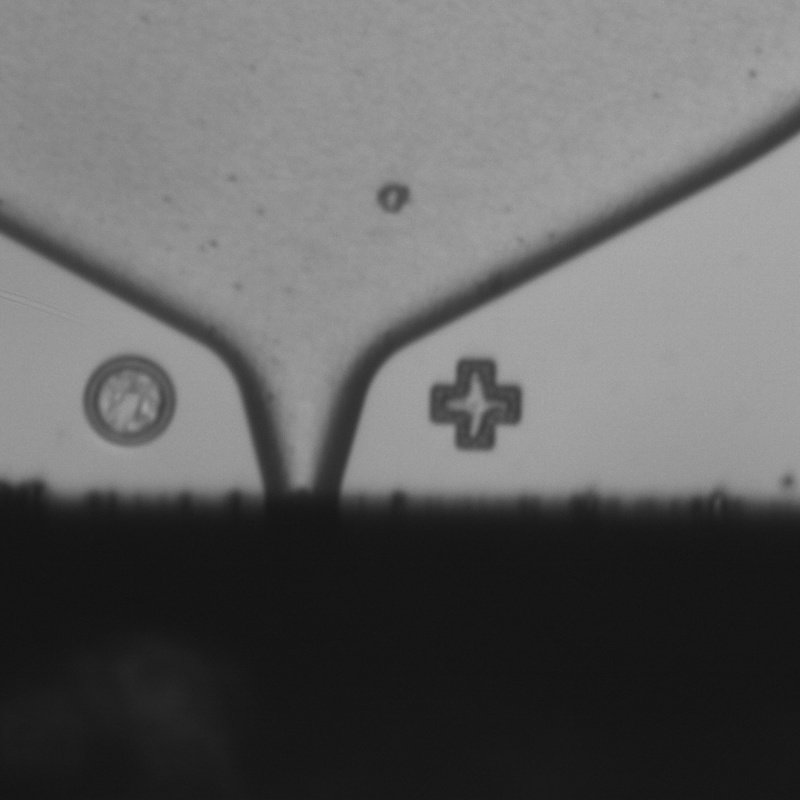

Supplement: Supplementary file 6 — Supplementary Data 3 [file 42003_2021_1661_MOESM6_ESM.zip › Supplementary Data 3 corrected/O_16_A.jpg]

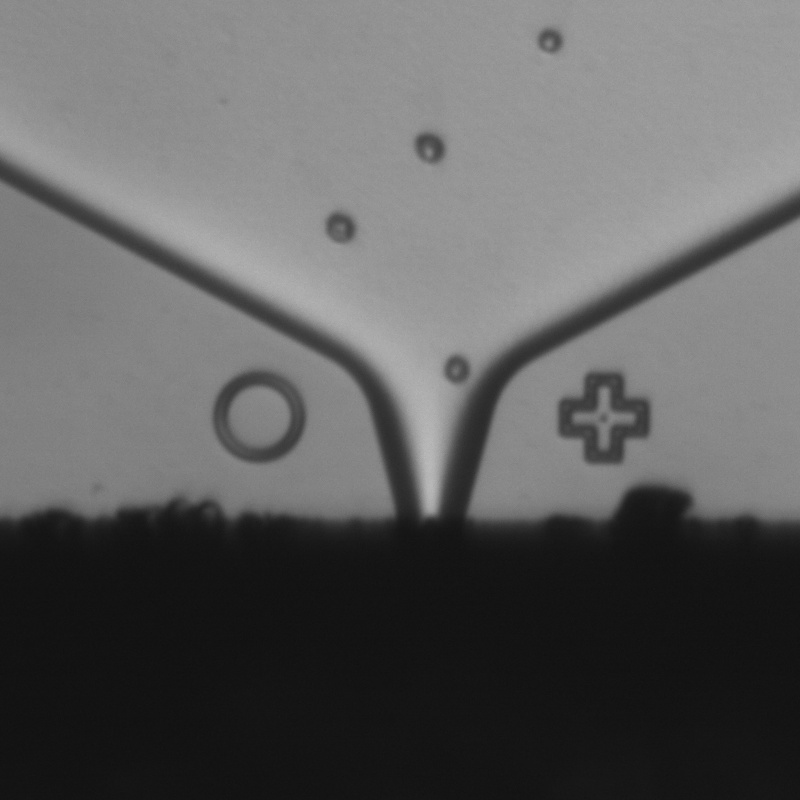

Supplement: Supplementary file 6 — Supplementary Data 3 [file 42003_2021_1661_MOESM6_ESM.zip › Supplementary Data 3 corrected/K_07_C.jpg]

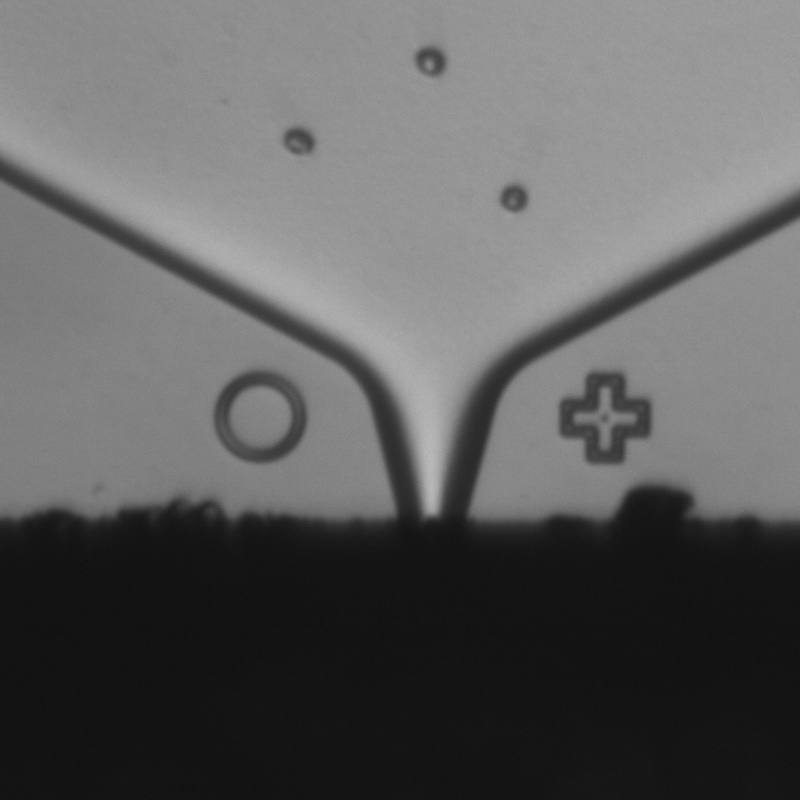

Supplement: Supplementary file 6 — Supplementary Data 3 [file 42003_2021_1661_MOESM6_ESM.zip › Supplementary Data 3 corrected/K_07_A.jpg]

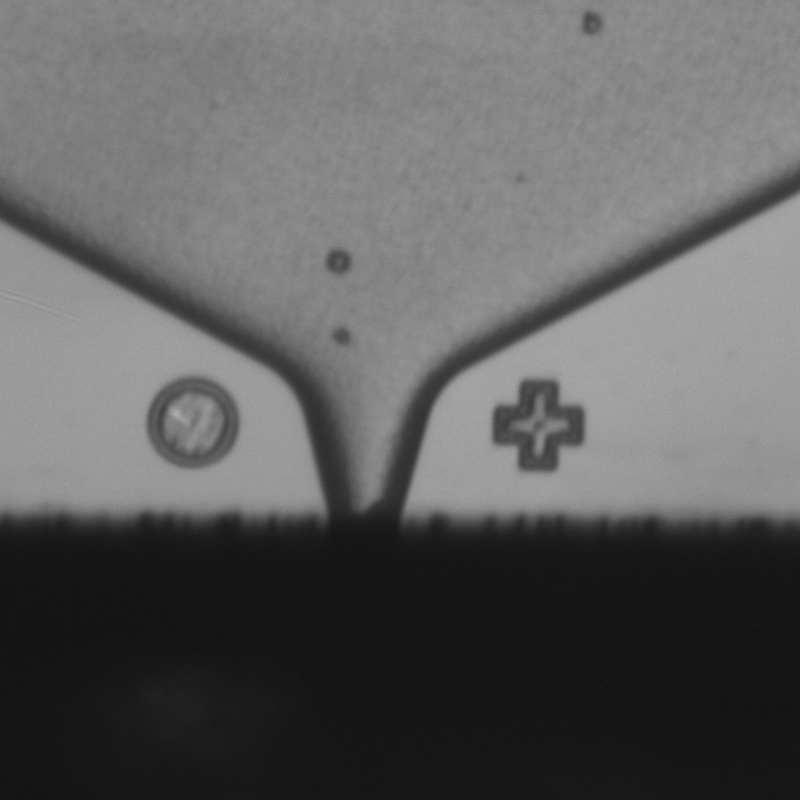

Supplement: Supplementary file 6 — Supplementary Data 3 [file 42003_2021_1661_MOESM6_ESM.zip › Supplementary Data 3 corrected/K_21_B.jpg]

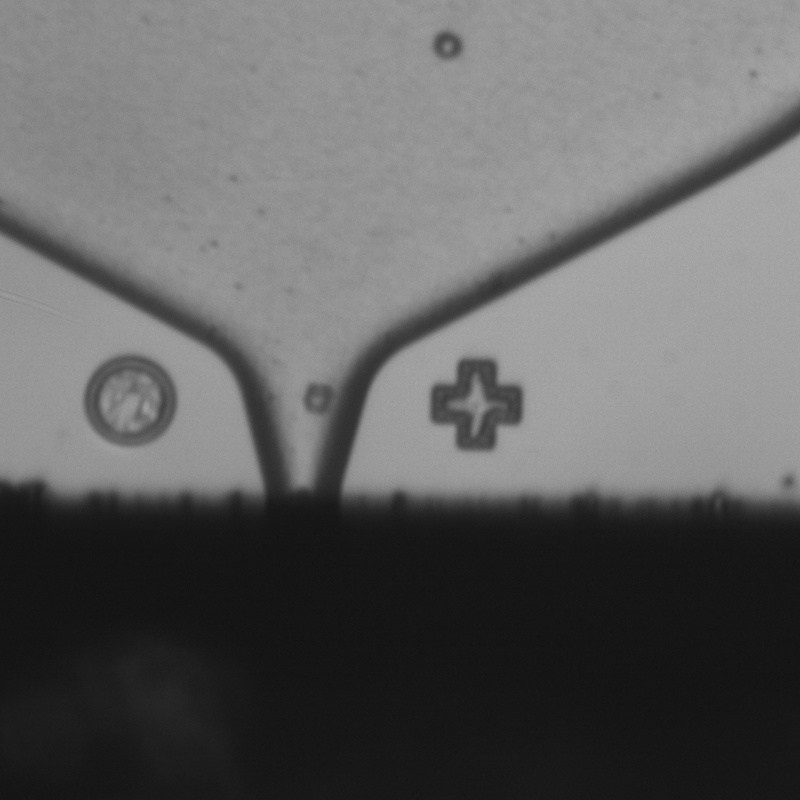

Supplement: Supplementary file 6 — Supplementary Data 3 [file 42003_2021_1661_MOESM6_ESM.zip › Supplementary Data 3 corrected/O_16_C.jpg]

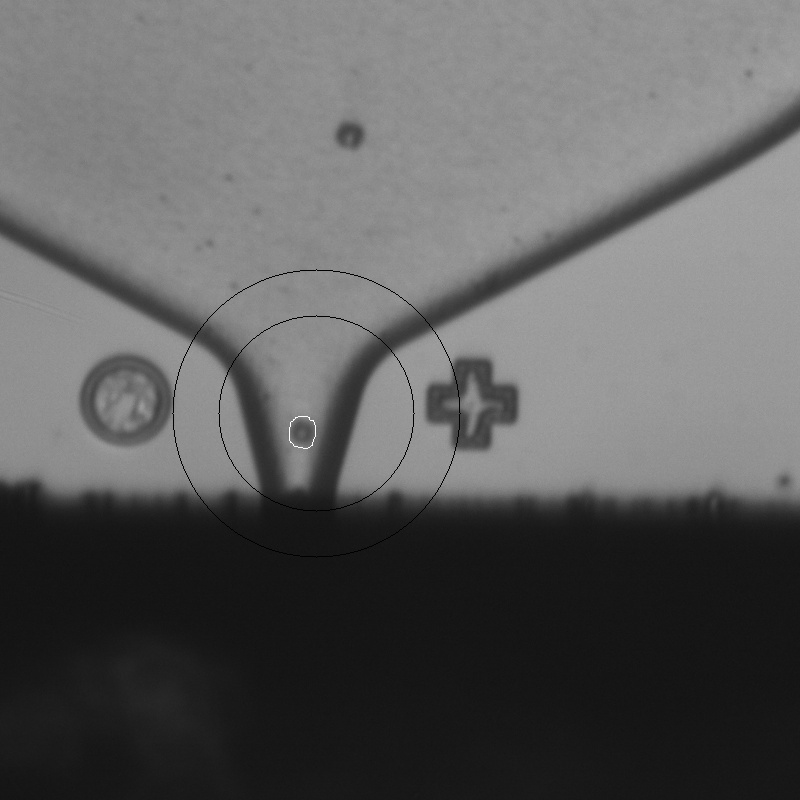

Supplement: Supplementary file 6 — Supplementary Data 3 [file 42003_2021_1661_MOESM6_ESM.zip › Supplementary Data 3 corrected/O_09_D.jpg]

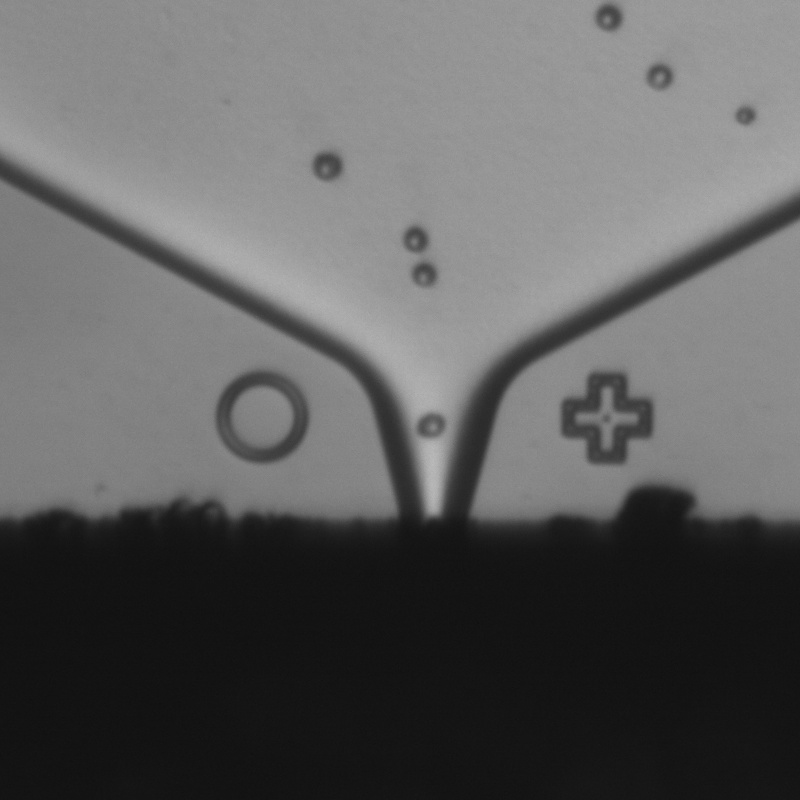

Supplement: Supplementary file 6 — Supplementary Data 3 [file 42003_2021_1661_MOESM6_ESM.zip › Supplementary Data 3 corrected/K_05_C.jpg]

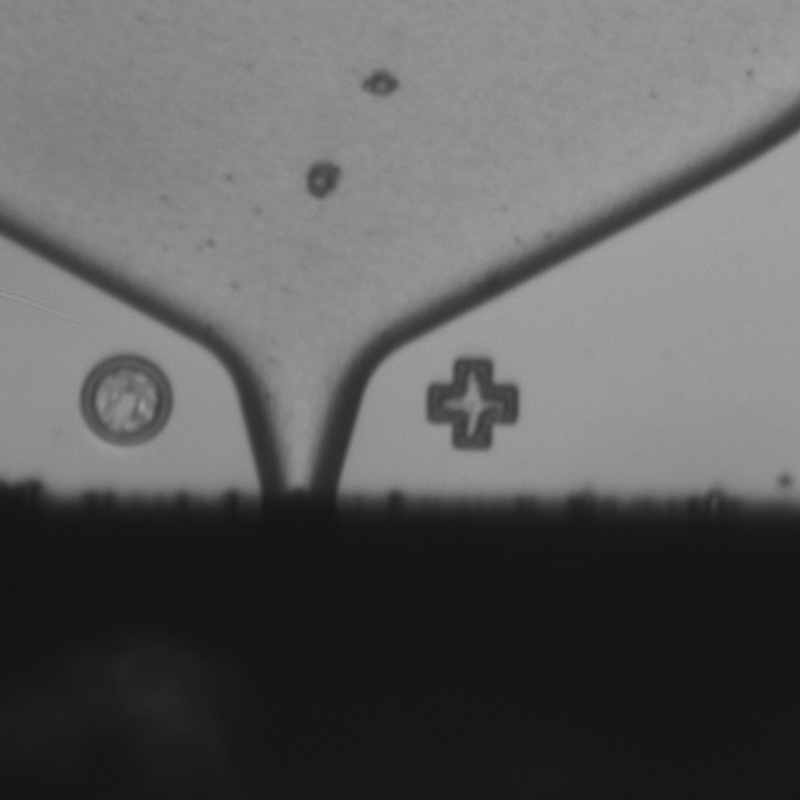

Supplement: Supplementary file 6 — Supplementary Data 3 [file 42003_2021_1661_MOESM6_ESM.zip › Supplementary Data 3 corrected/O_14_A.jpg]

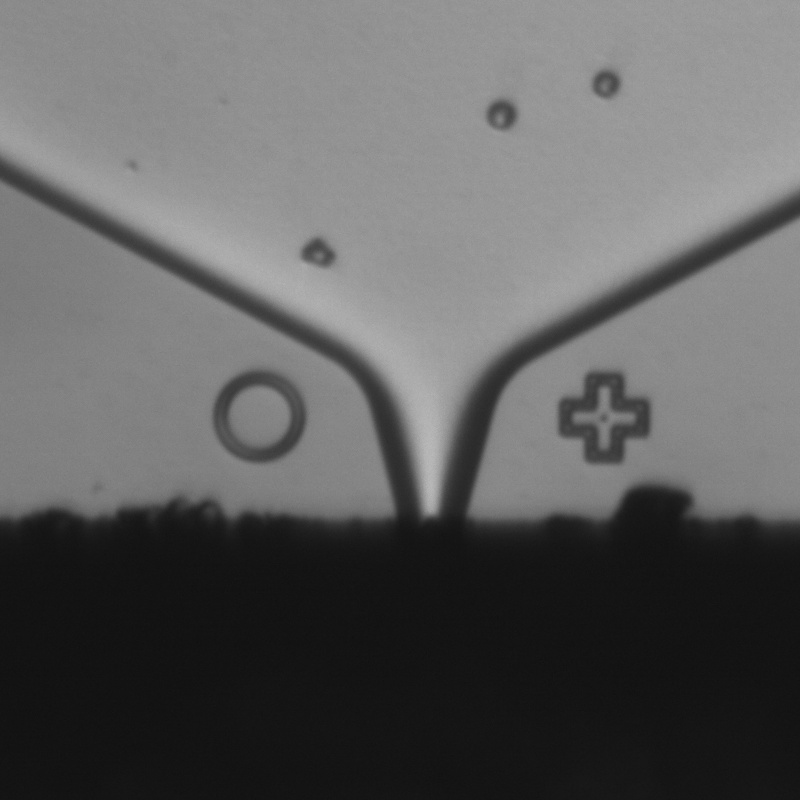

Supplement: Supplementary file 6 — Supplementary Data 3 [file 42003_2021_1661_MOESM6_ESM.zip › Supplementary Data 3 corrected/K_03_E.jpg]

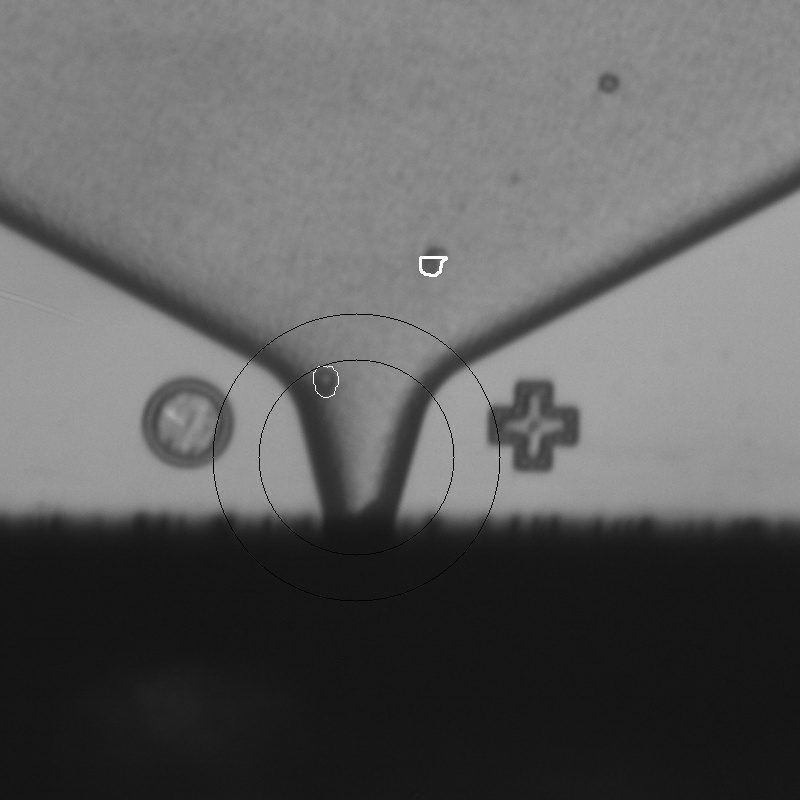

Supplement: Supplementary file 6 — Supplementary Data 3 [file 42003_2021_1661_MOESM6_ESM.zip › Supplementary Data 3 corrected/K_11_D1.jpg]

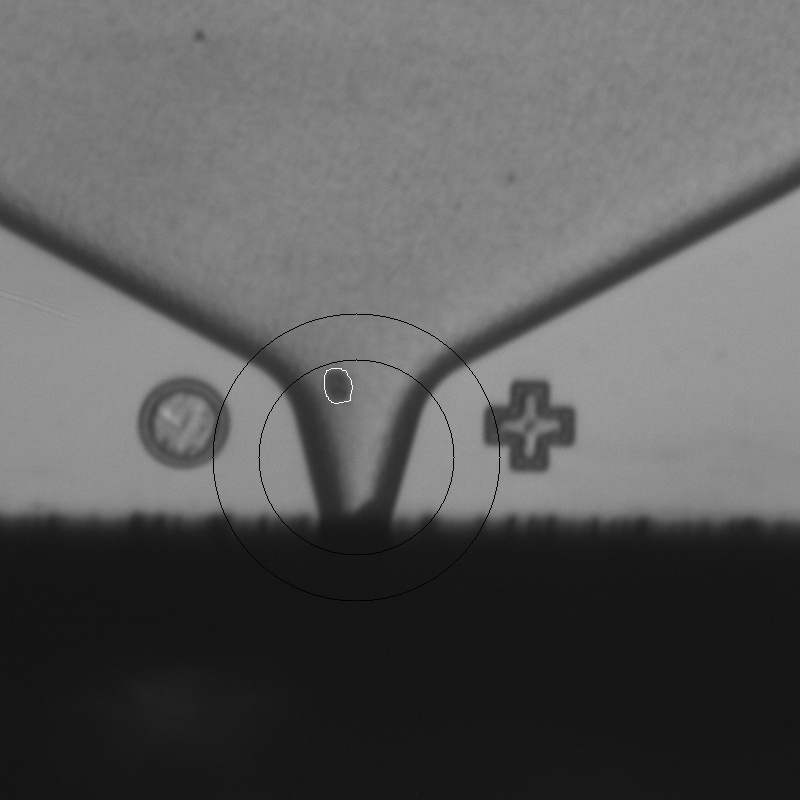

Supplement: Supplementary file 6 — Supplementary Data 3 [file 42003_2021_1661_MOESM6_ESM.zip › Supplementary Data 3 corrected/K_27_D.jpg]

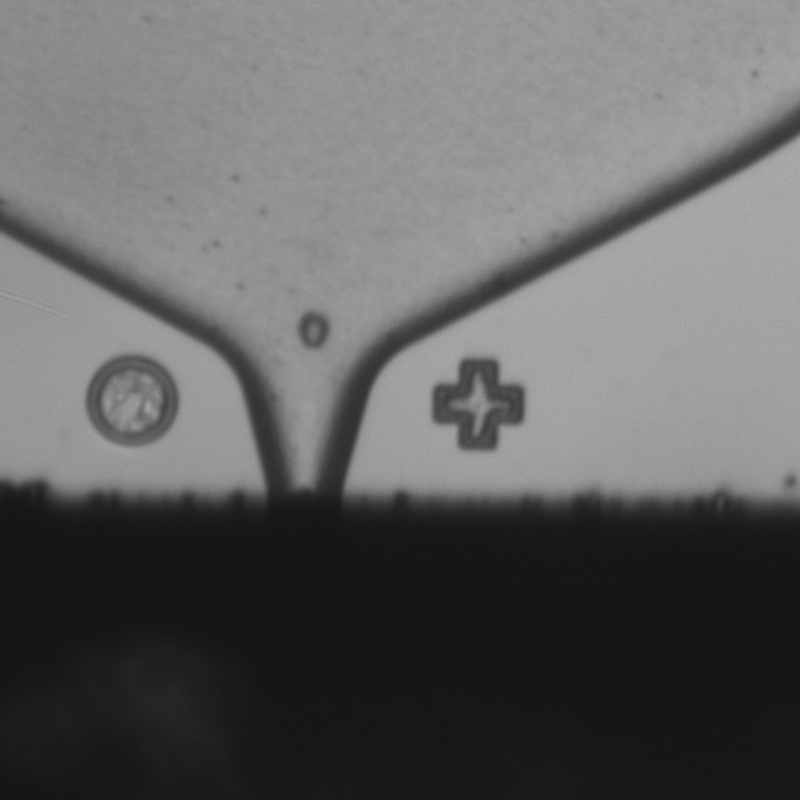

Supplement: Supplementary file 6 — Supplementary Data 3 [file 42003_2021_1661_MOESM6_ESM.zip › Supplementary Data 3 corrected/O_10_E.jpg]

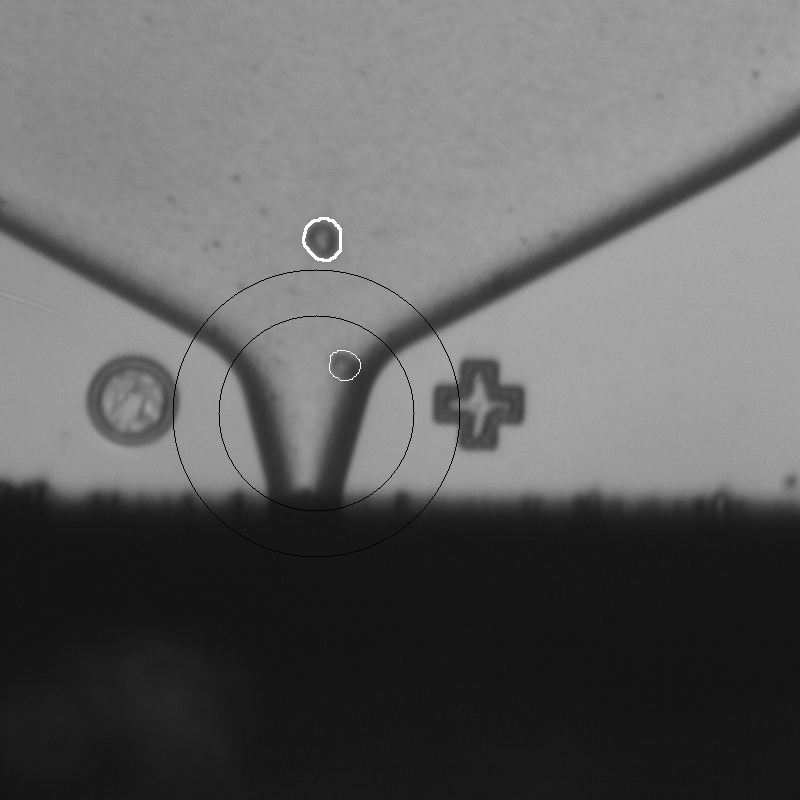

Supplement: Supplementary file 6 — Supplementary Data 3 [file 42003_2021_1661_MOESM6_ESM.zip › Supplementary Data 3 corrected/O_10_D.jpg]

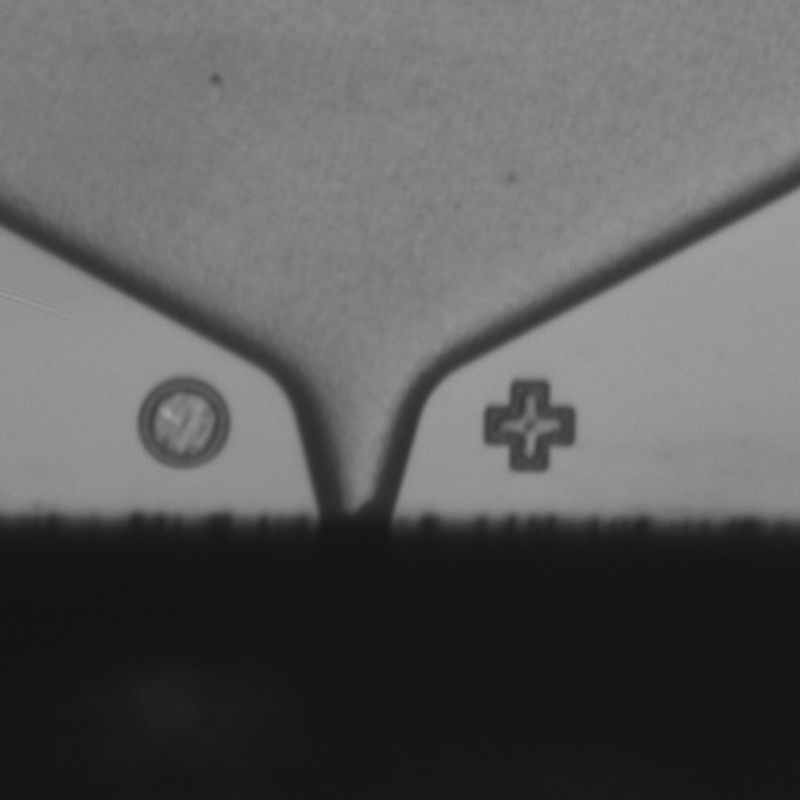

Supplement: Supplementary file 6 — Supplementary Data 3 [file 42003_2021_1661_MOESM6_ESM.zip › Supplementary Data 3 corrected/K_27_E.jpg]

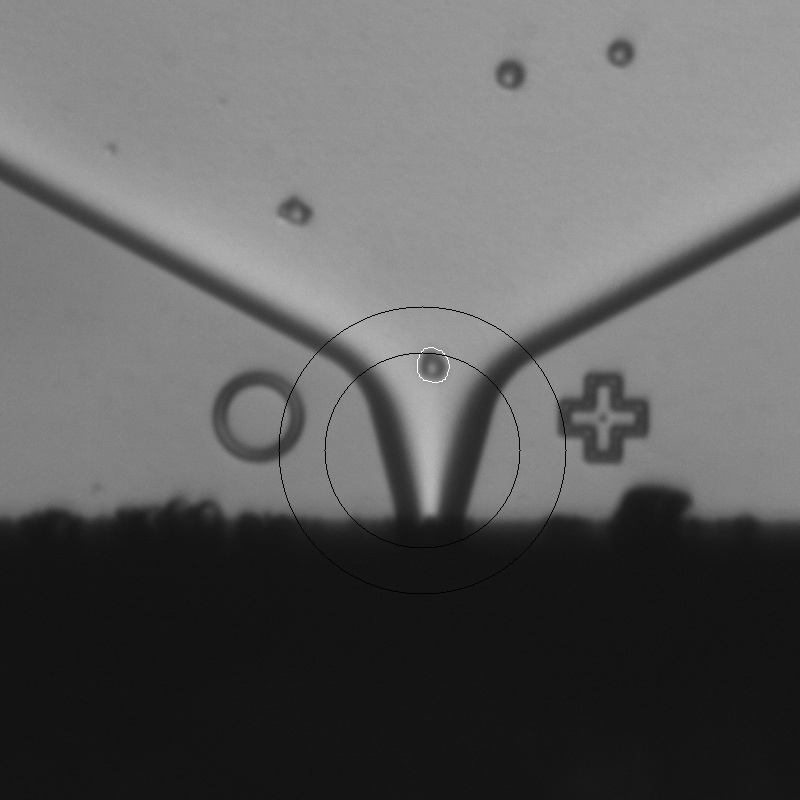

Supplement: Supplementary file 6 — Supplementary Data 3 [file 42003_2021_1661_MOESM6_ESM.zip › Supplementary Data 3 corrected/K_03_D.jpg]

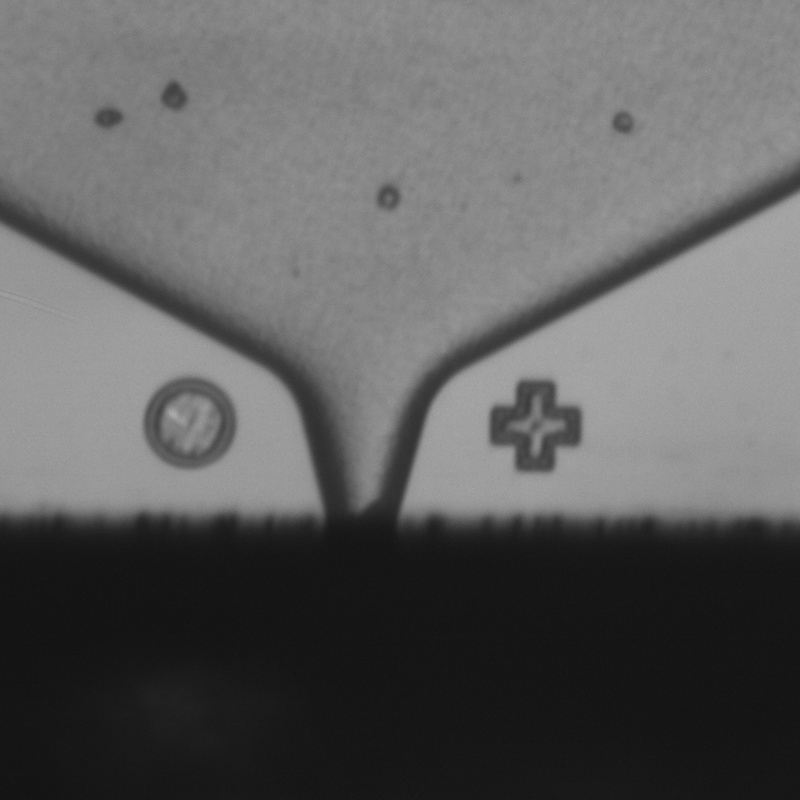

Supplement: Supplementary file 6 — Supplementary Data 3 [file 42003_2021_1661_MOESM6_ESM.zip › Supplementary Data 3 corrected/K_23_A.jpg]

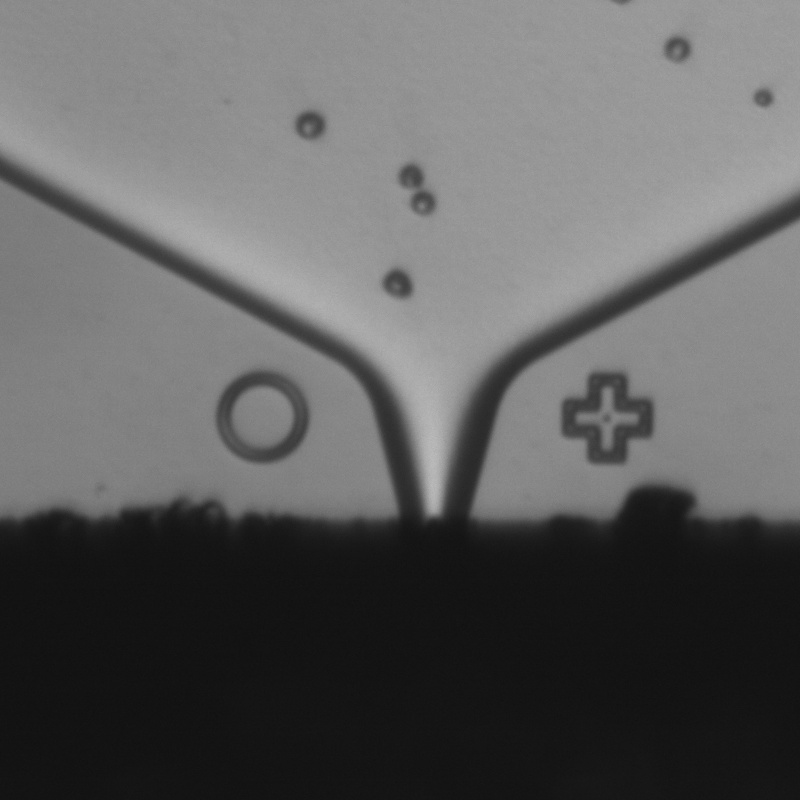

Supplement: Supplementary file 6 — Supplementary Data 3 [file 42003_2021_1661_MOESM6_ESM.zip › Supplementary Data 3 corrected/K_05_B.jpg]

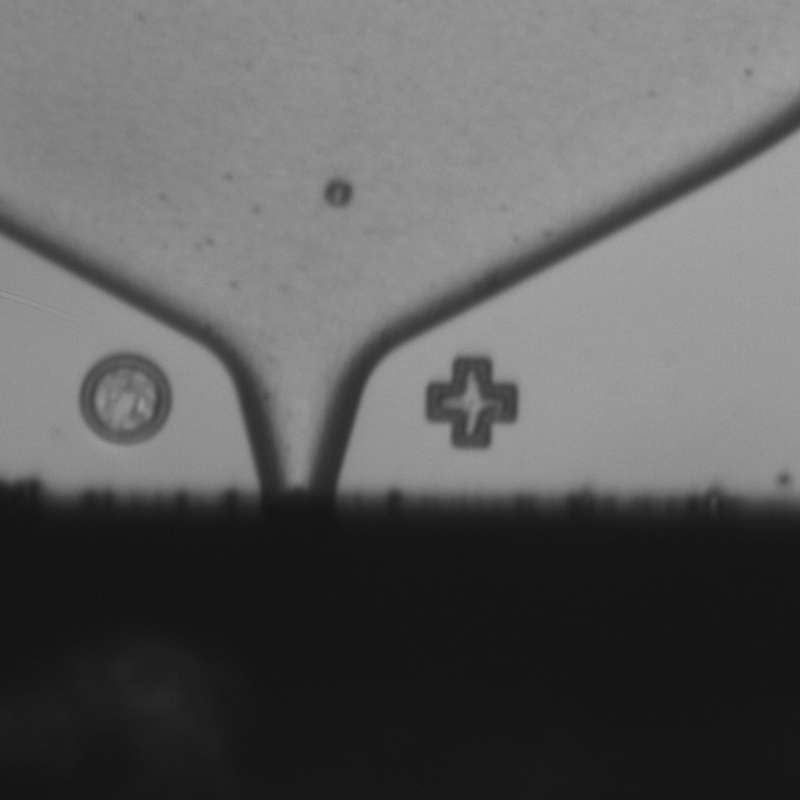

Supplement: Supplementary file 6 — Supplementary Data 3 [file 42003_2021_1661_MOESM6_ESM.zip › Supplementary Data 3 corrected/O_09_E.jpg]

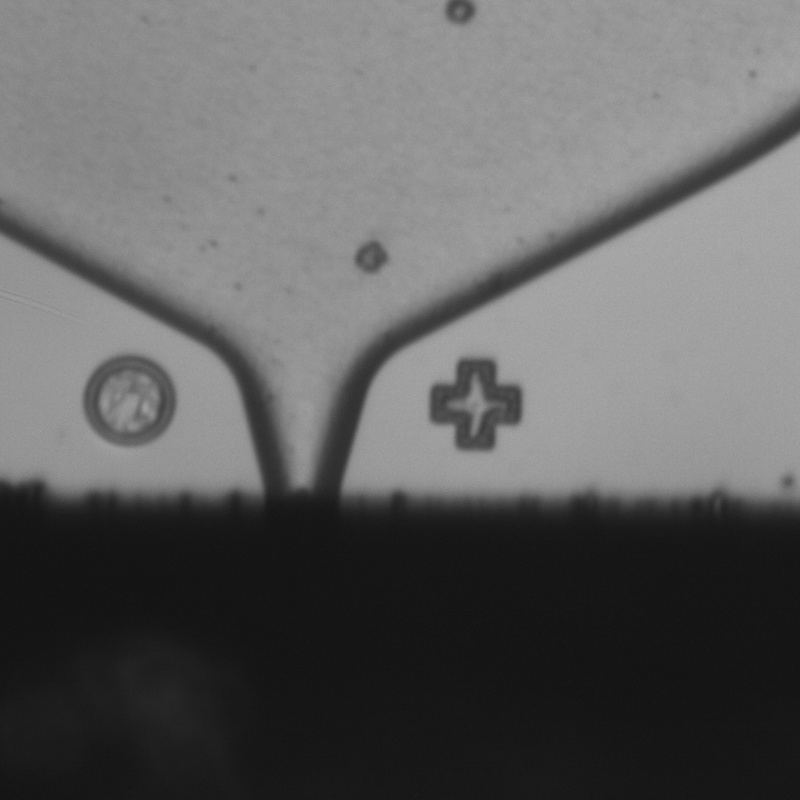

Supplement: Supplementary file 6 — Supplementary Data 3 [file 42003_2021_1661_MOESM6_ESM.zip › Supplementary Data 3 corrected/O_16_B.jpg]

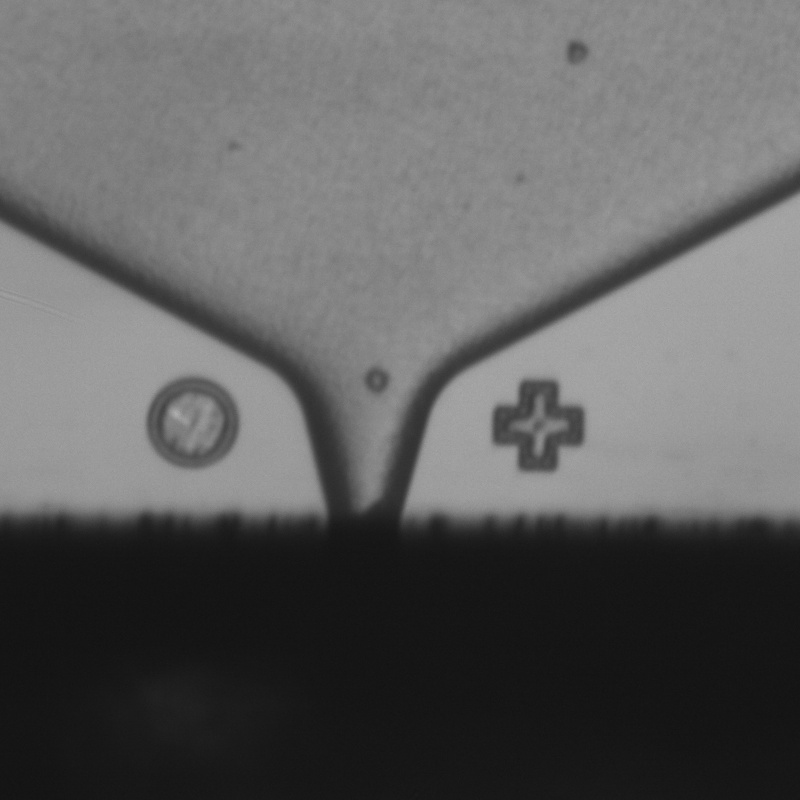

Supplement: Supplementary file 6 — Supplementary Data 3 [file 42003_2021_1661_MOESM6_ESM.zip › Supplementary Data 3 corrected/K_21_C.jpg]

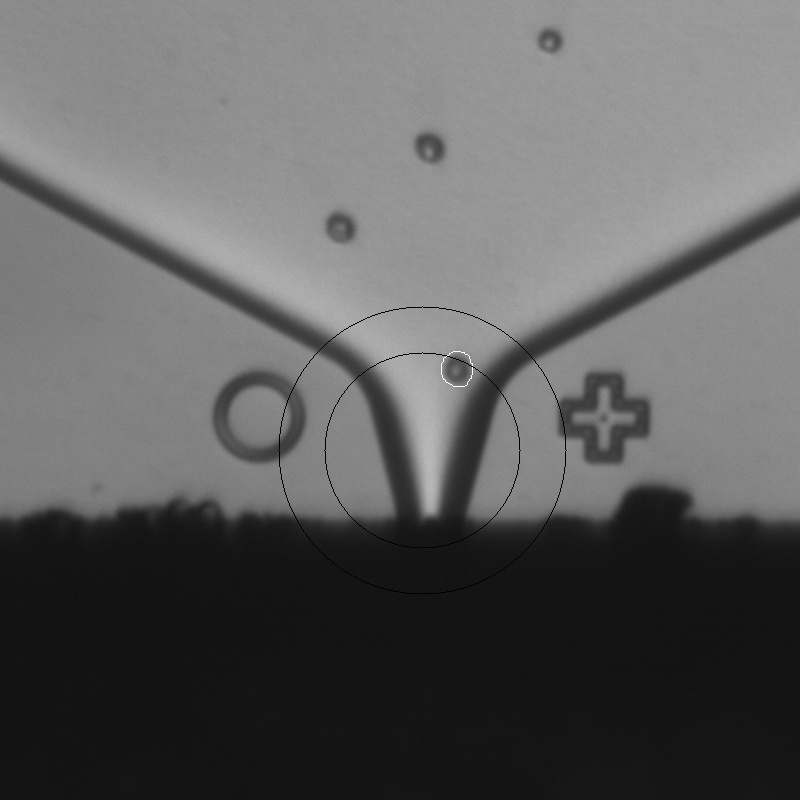

Supplement: Supplementary file 6 — Supplementary Data 3 [file 42003_2021_1661_MOESM6_ESM.zip › Supplementary Data 3 corrected/K_07_D.jpg]

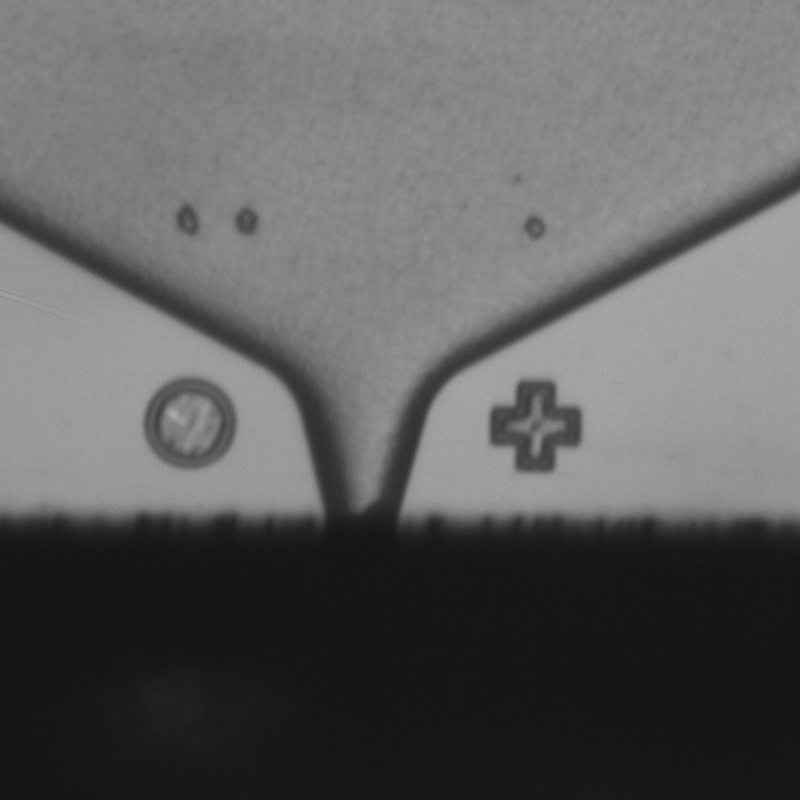

Supplement: Supplementary file 6 — Supplementary Data 3 [file 42003_2021_1661_MOESM6_ESM.zip › Supplementary Data 3 corrected/K_23_E.jpg]

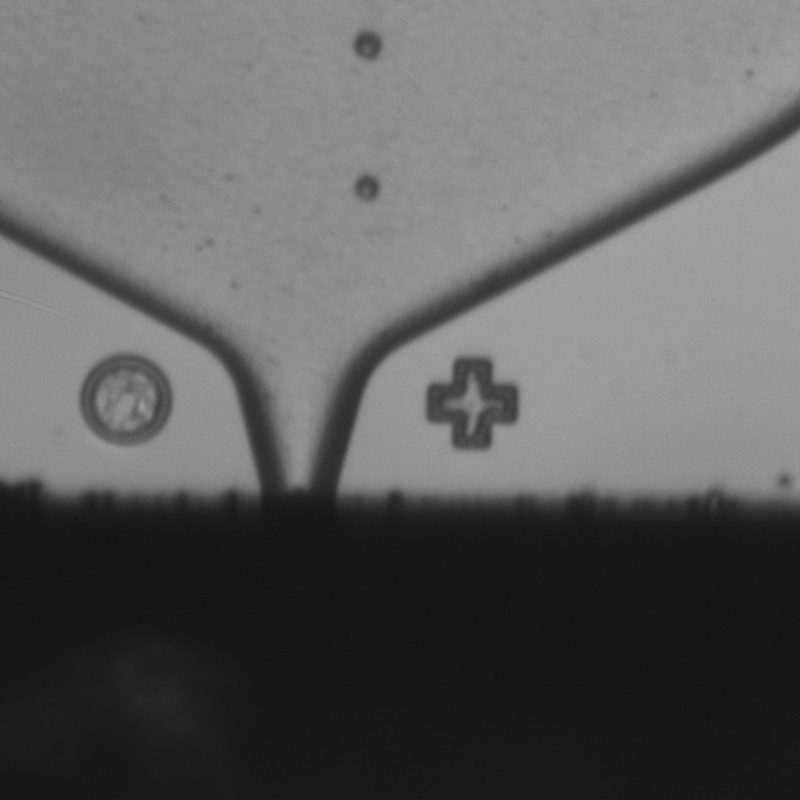

Supplement: Supplementary file 6 — Supplementary Data 3 [file 42003_2021_1661_MOESM6_ESM.zip › Supplementary Data 3 corrected/O_09_A.jpg]

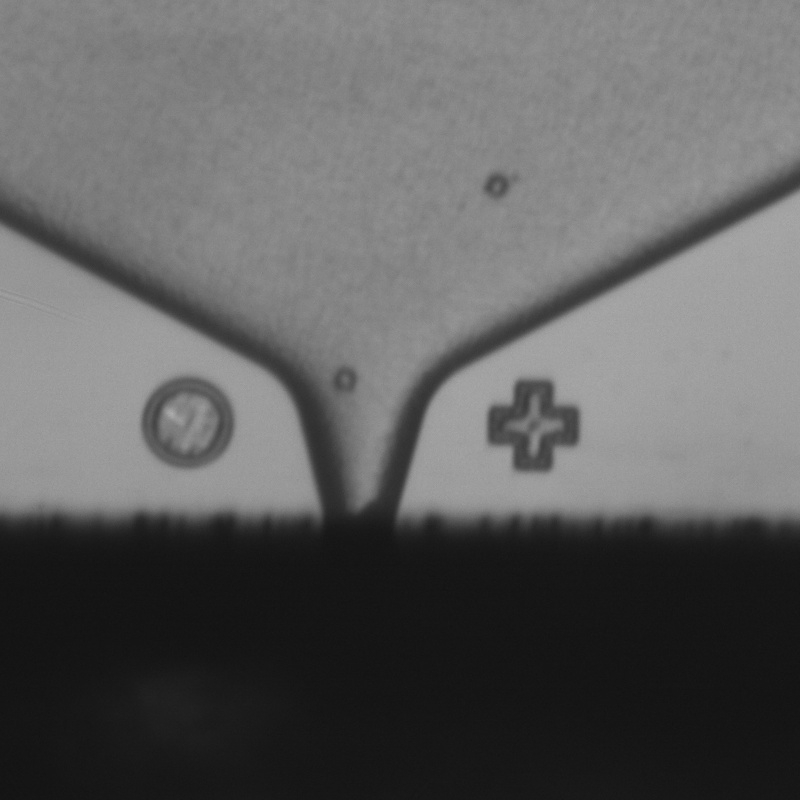

Supplement: Supplementary file 6 — Supplementary Data 3 [file 42003_2021_1661_MOESM6_ESM.zip › Supplementary Data 3 corrected/K_18_C.jpg]

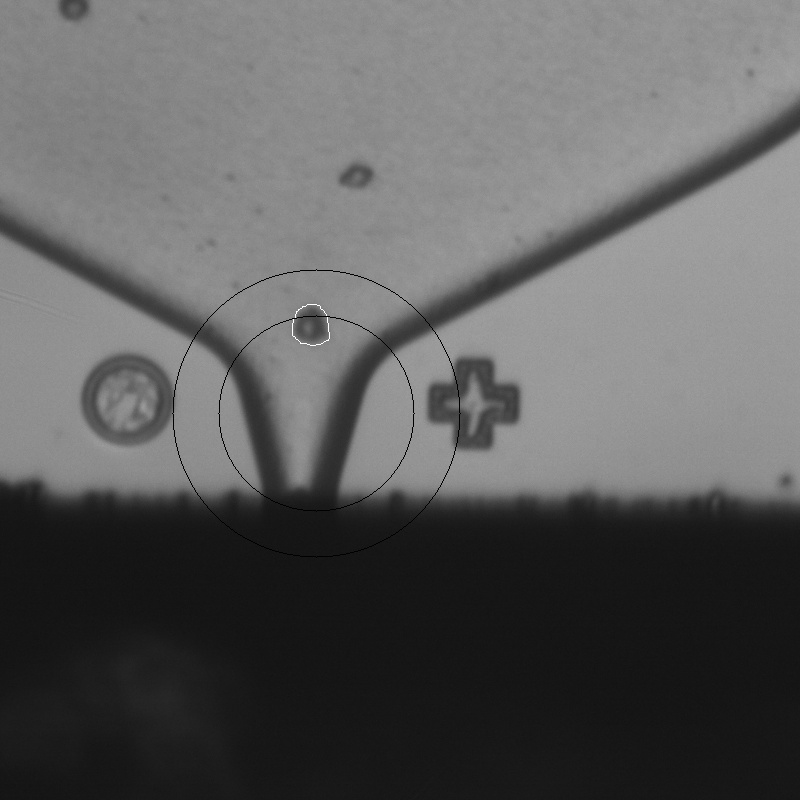

Supplement: Supplementary file 6 — Supplementary Data 3 [file 42003_2021_1661_MOESM6_ESM.zip › Supplementary Data 3 corrected/O_14_D.jpg]

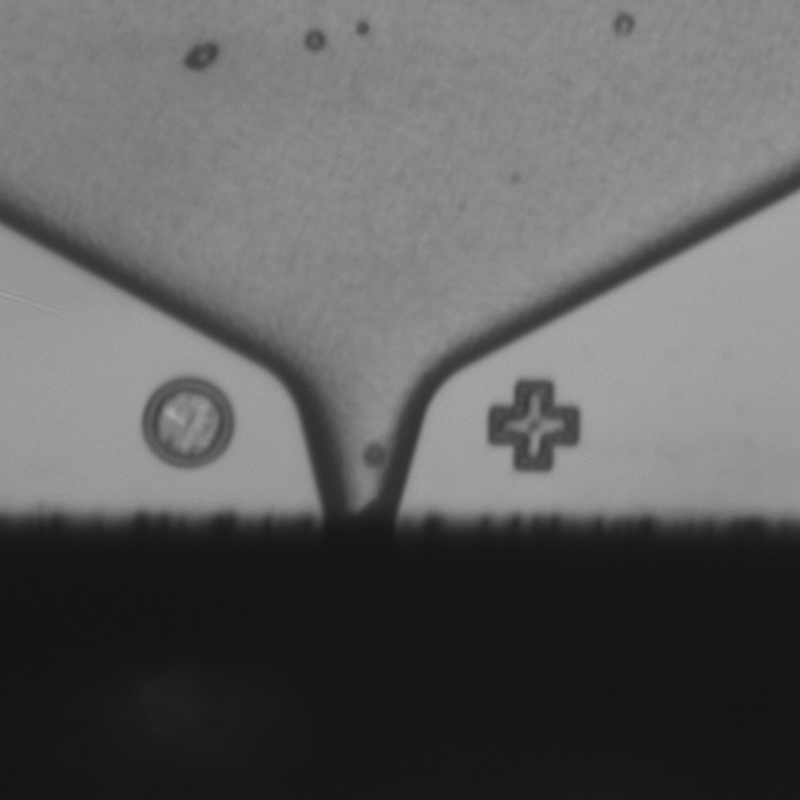

Supplement: Supplementary file 6 — Supplementary Data 3 [file 42003_2021_1661_MOESM6_ESM.zip › Supplementary Data 3 corrected/K_25_C.jpg]

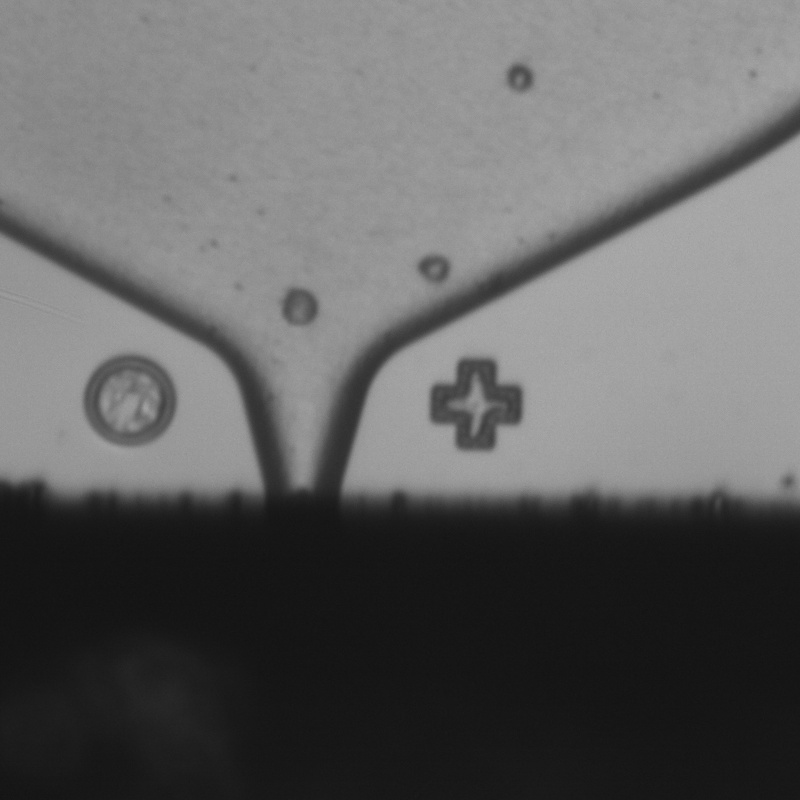

Supplement: Supplementary file 6 — Supplementary Data 3 [file 42003_2021_1661_MOESM6_ESM.zip › Supplementary Data 3 corrected/O_12_B.jpg]

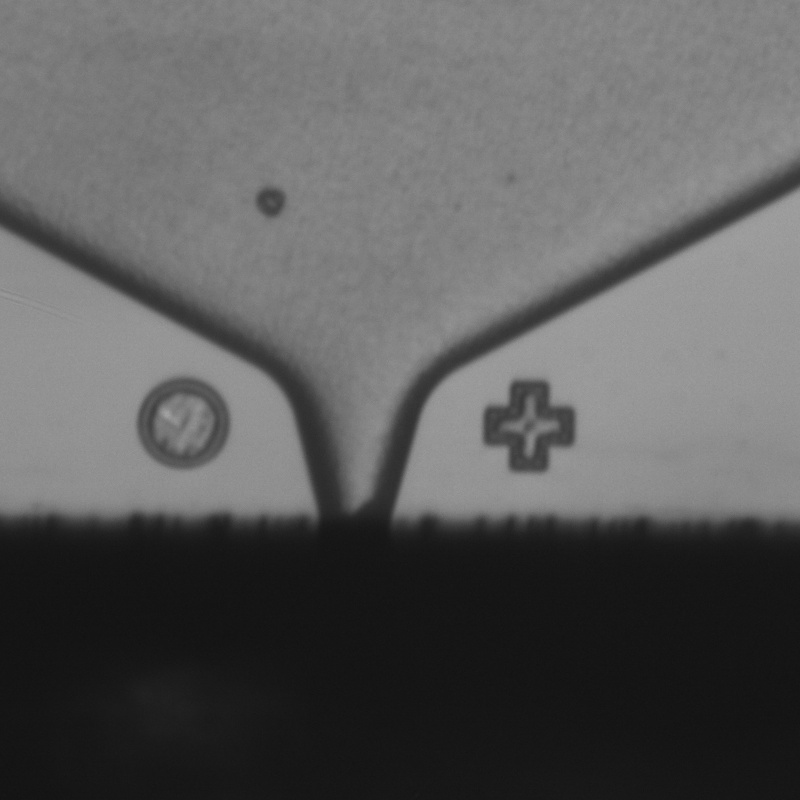

Supplement: Supplementary file 6 — Supplementary Data 3 [file 42003_2021_1661_MOESM6_ESM.zip › Supplementary Data 3 corrected/K_27_A.jpg]

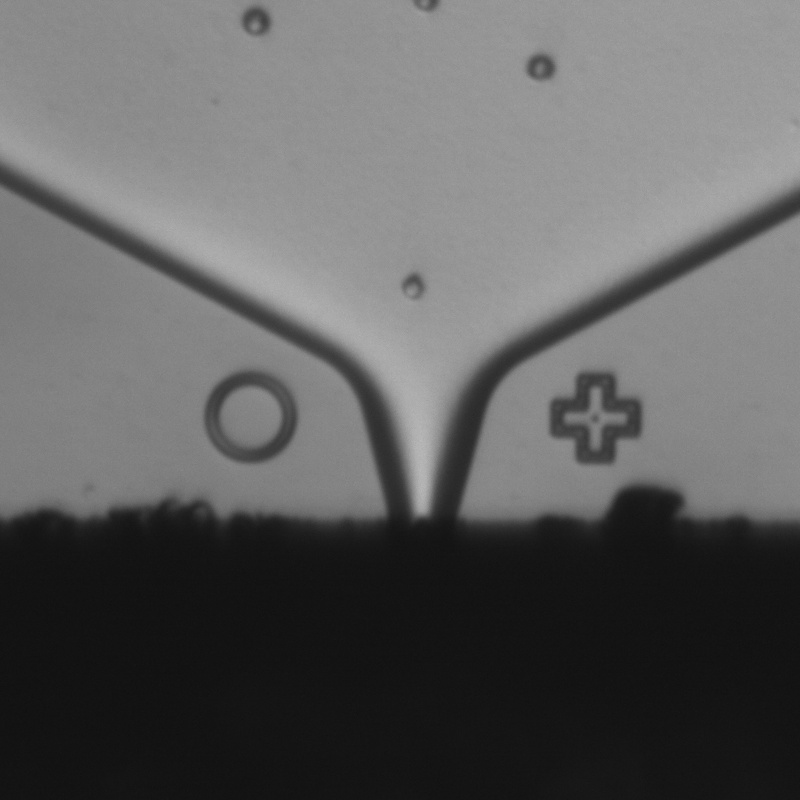

Supplement: Supplementary file 6 — Supplementary Data 3 [file 42003_2021_1661_MOESM6_ESM.zip › Supplementary Data 3 corrected/K_01_B.jpg]

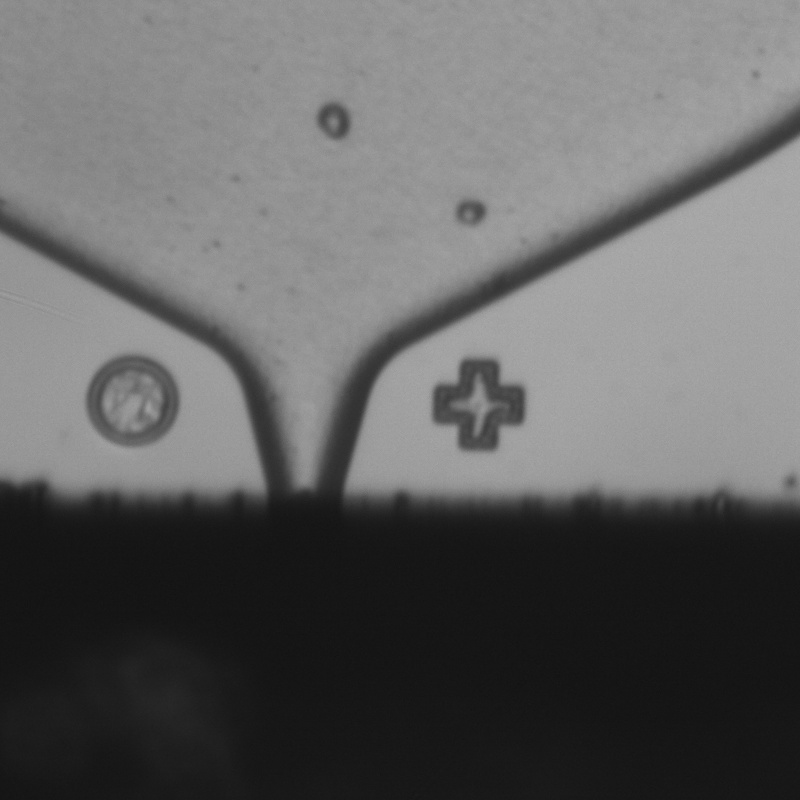

Supplement: Supplementary file 6 — Supplementary Data 3 [file 42003_2021_1661_MOESM6_ESM.zip › Supplementary Data 3 corrected/O_10_A.jpg]

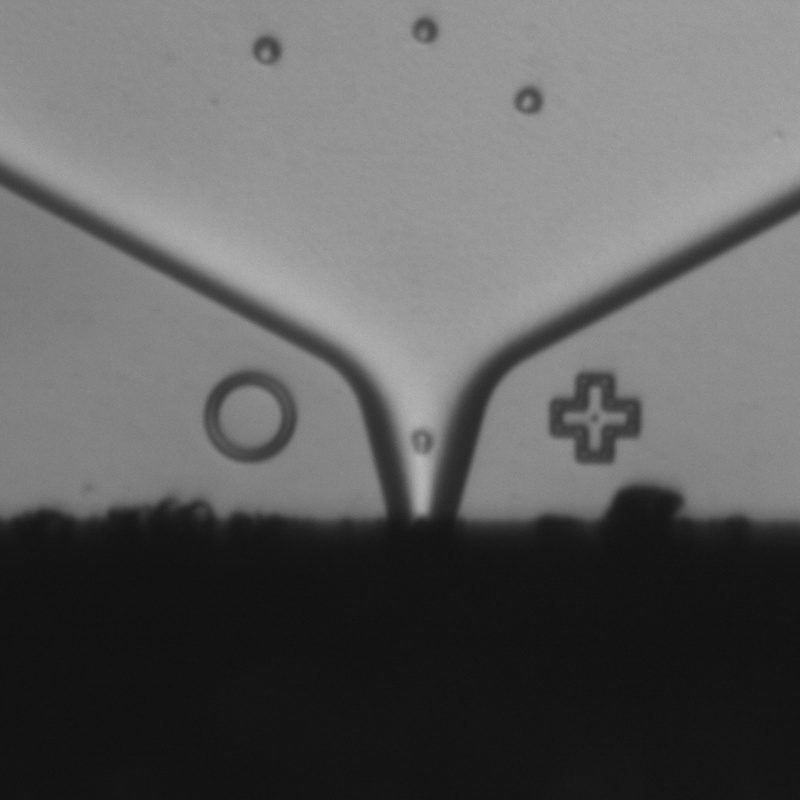

Supplement: Supplementary file 6 — Supplementary Data 3 [file 42003_2021_1661_MOESM6_ESM.zip › Supplementary Data 3 corrected/K_01_C.jpg]

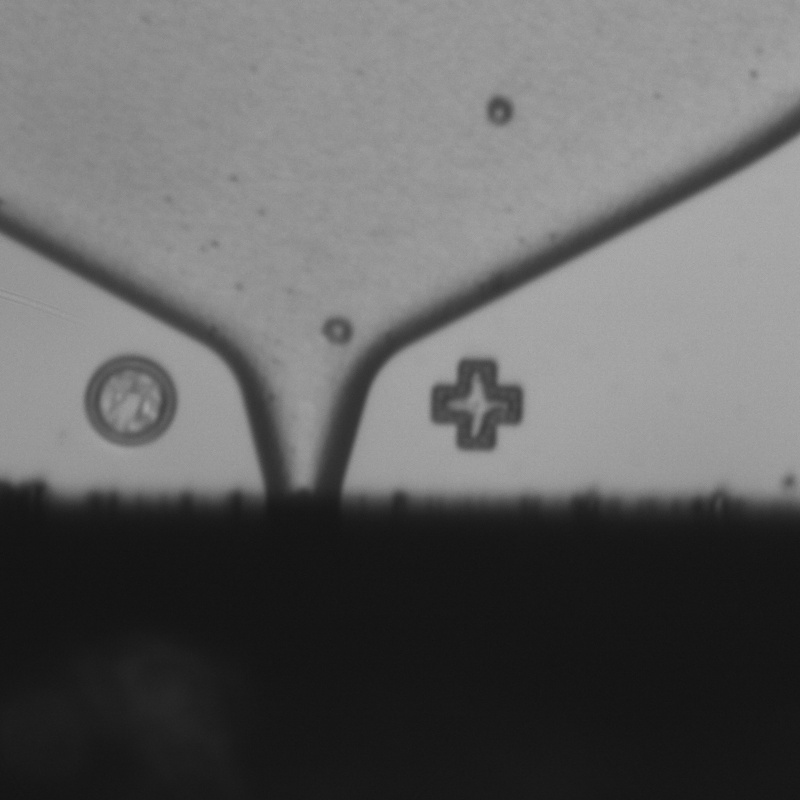

Supplement: Supplementary file 6 — Supplementary Data 3 [file 42003_2021_1661_MOESM6_ESM.zip › Supplementary Data 3 corrected/O_12_C.jpg]

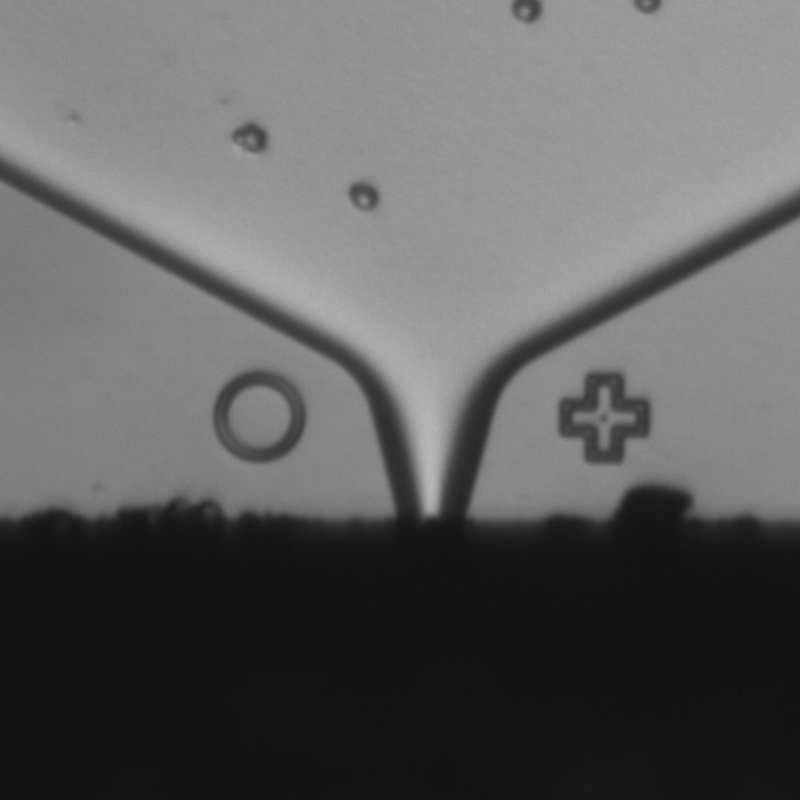

Supplement: Supplementary file 6 — Supplementary Data 3 [file 42003_2021_1661_MOESM6_ESM.zip › Supplementary Data 3 corrected/K_03_A.jpg]

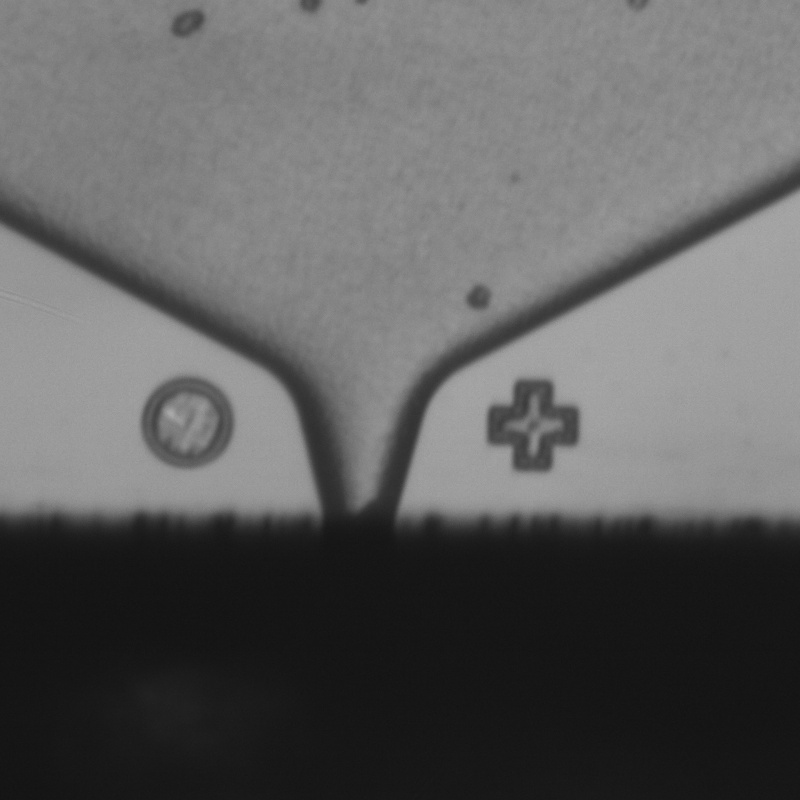

Supplement: Supplementary file 6 — Supplementary Data 3 [file 42003_2021_1661_MOESM6_ESM.zip › Supplementary Data 3 corrected/K_25_B.jpg]

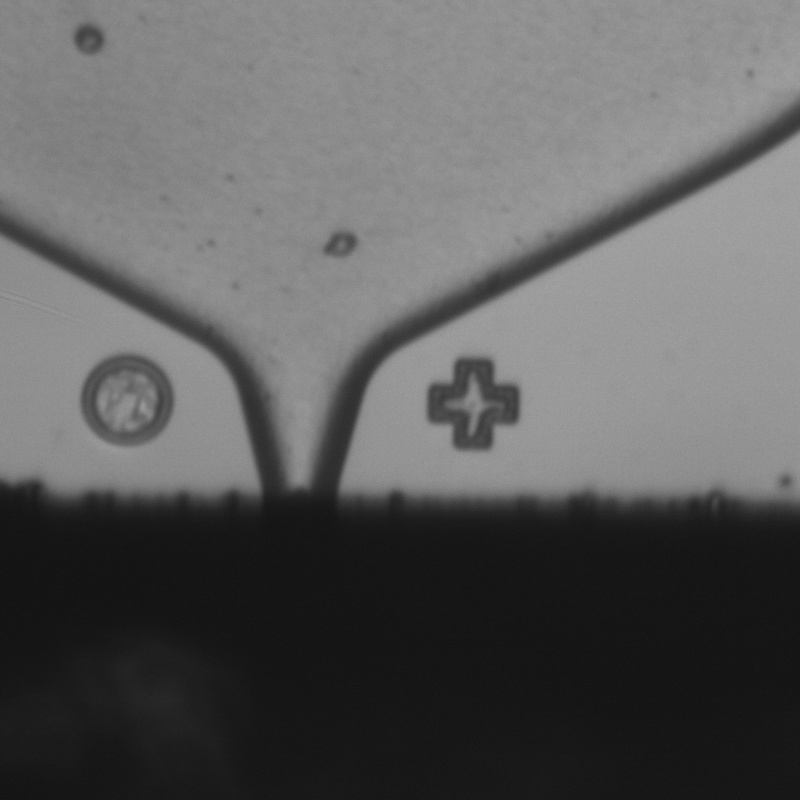

Supplement: Supplementary file 6 — Supplementary Data 3 [file 42003_2021_1661_MOESM6_ESM.zip › Supplementary Data 3 corrected/O_14_E.jpg]

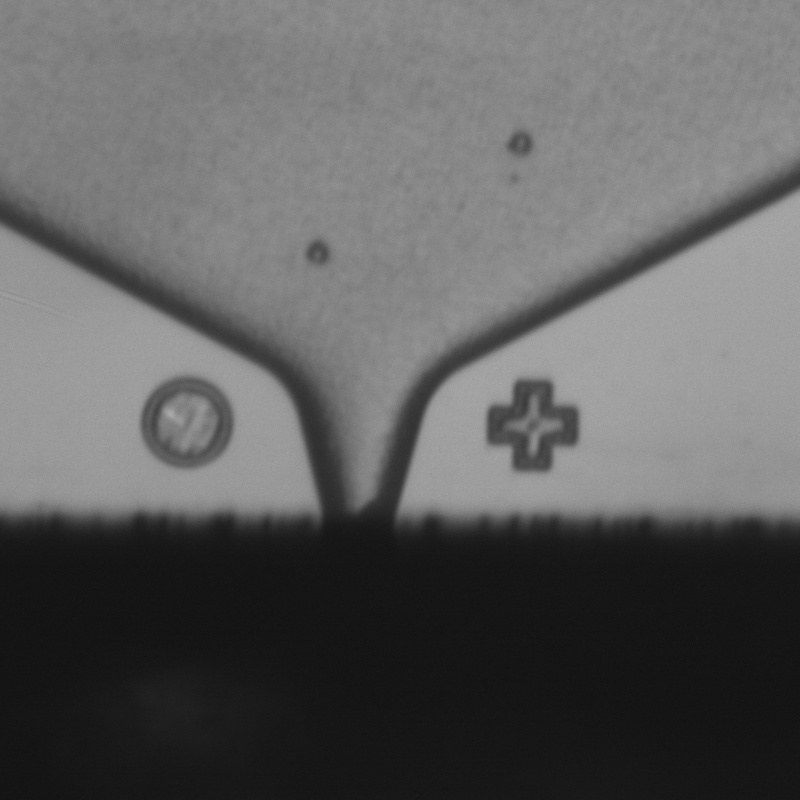

Supplement: Supplementary file 6 — Supplementary Data 3 [file 42003_2021_1661_MOESM6_ESM.zip › Supplementary Data 3 corrected/K_18_B.jpg]

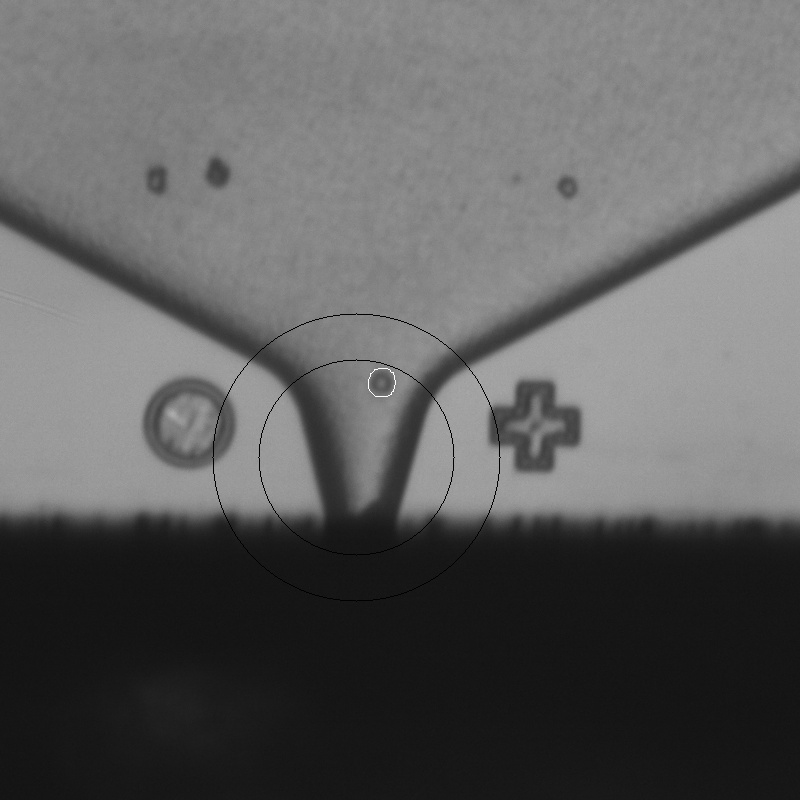

Supplement: Supplementary file 6 — Supplementary Data 3 [file 42003_2021_1661_MOESM6_ESM.zip › Supplementary Data 3 corrected/K_23_D.jpg]

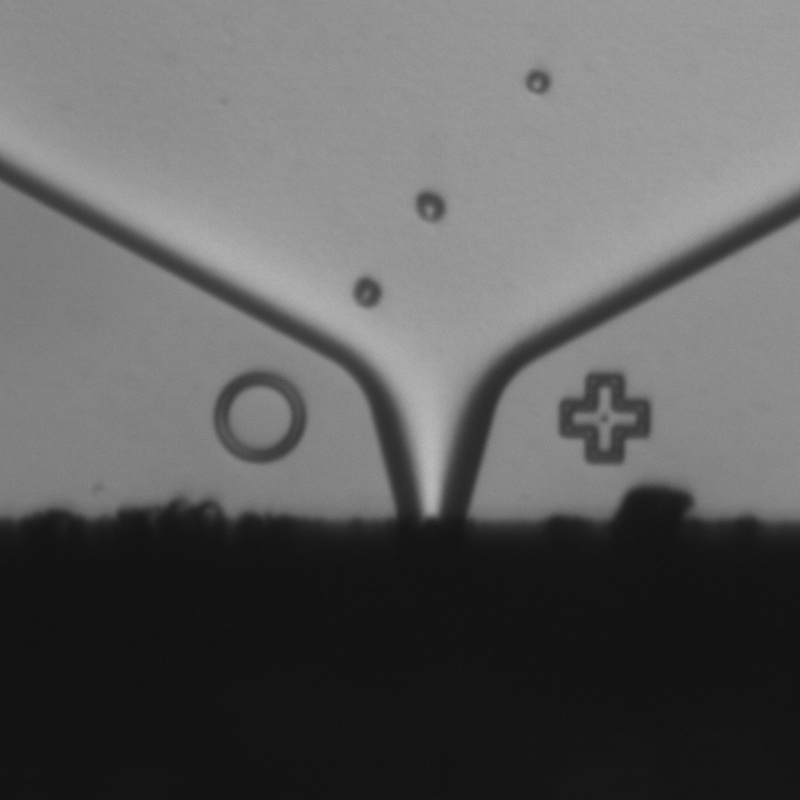

Supplement: Supplementary file 6 — Supplementary Data 3 [file 42003_2021_1661_MOESM6_ESM.zip › Supplementary Data 3 corrected/K_07_E.jpg]

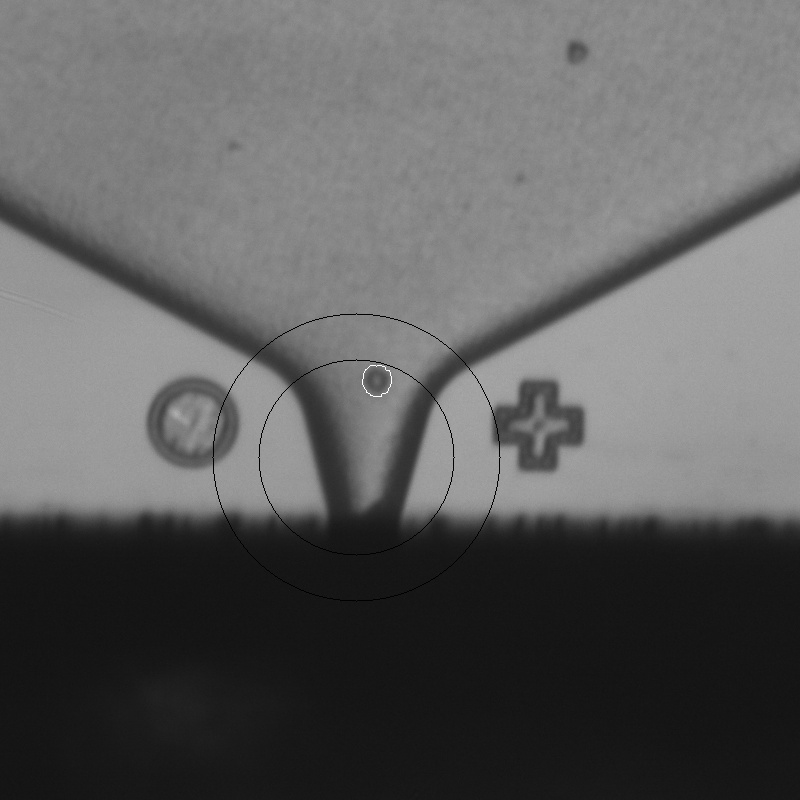

Supplement: Supplementary file 6 — Supplementary Data 3 [file 42003_2021_1661_MOESM6_ESM.zip › Supplementary Data 3 corrected/K_21_D.jpg]

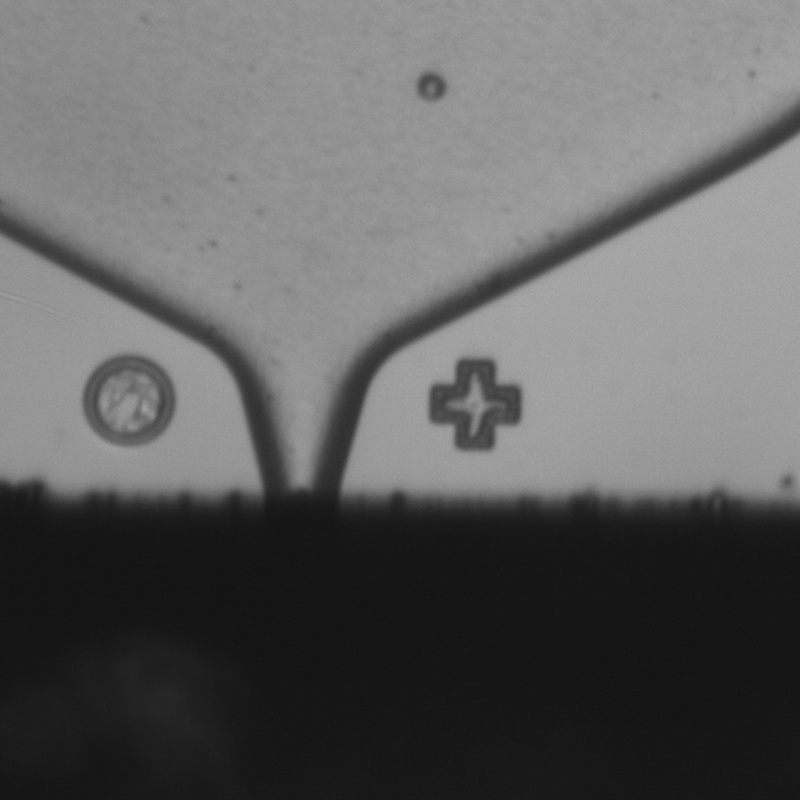

Supplement: Supplementary file 6 — Supplementary Data 3 [file 42003_2021_1661_MOESM6_ESM.zip › Supplementary Data 3 corrected/O_16_E.jpg]

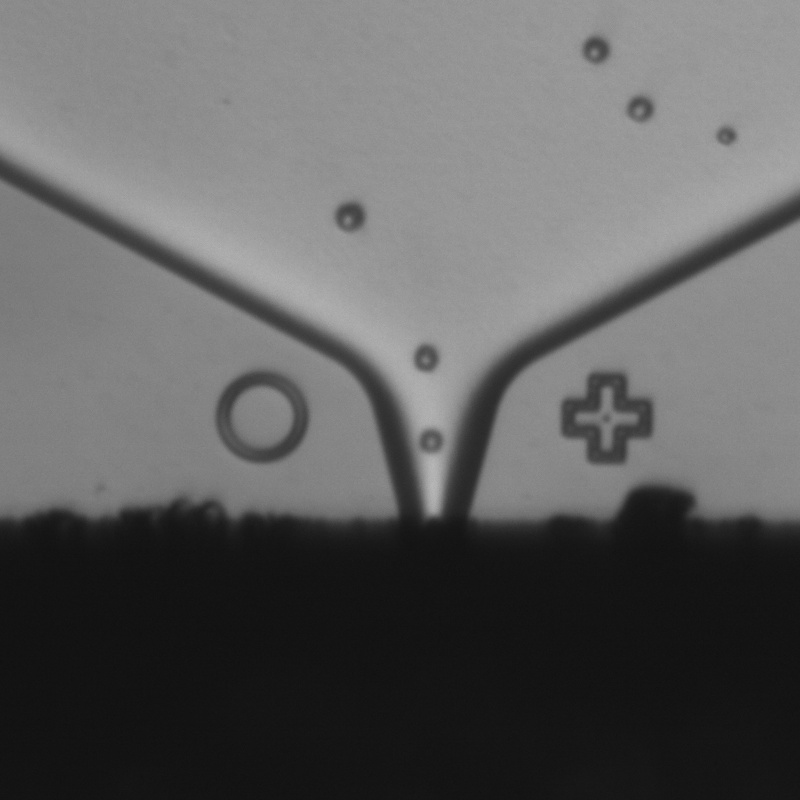

Supplement: Supplementary file 6 — Supplementary Data 3 [file 42003_2021_1661_MOESM6_ESM.zip › Supplementary Data 3 corrected/K_05_E.jpg]

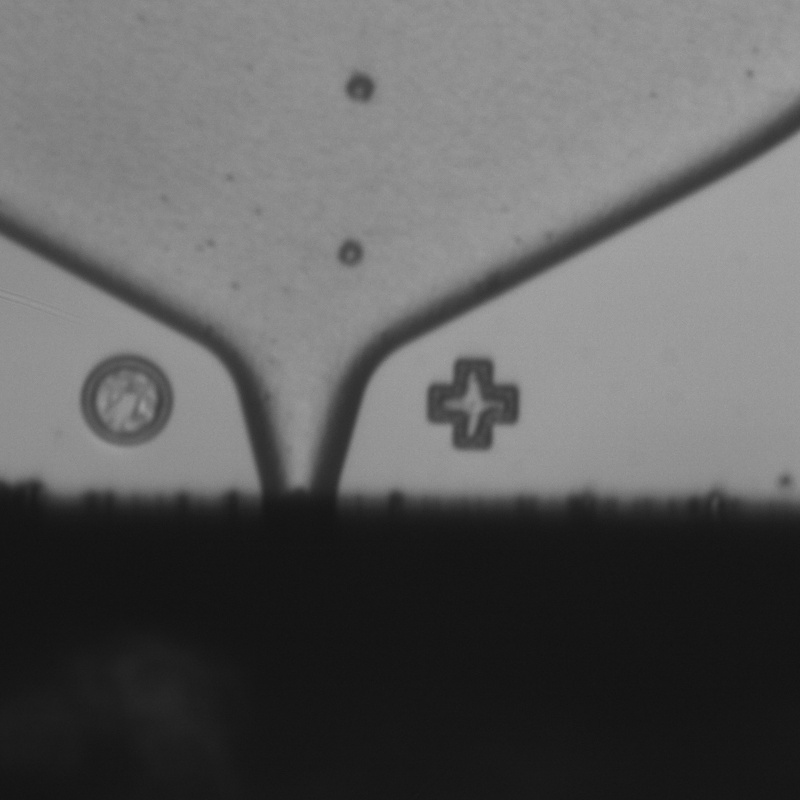

Supplement: Supplementary file 6 — Supplementary Data 3 [file 42003_2021_1661_MOESM6_ESM.zip › Supplementary Data 3 corrected/O_09_B.jpg]

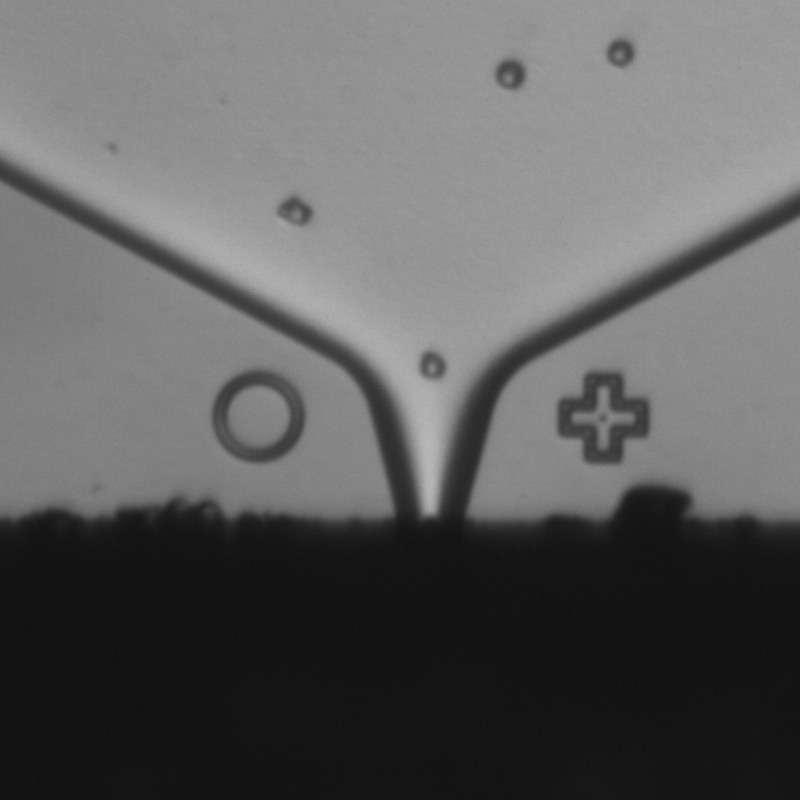

Supplement: Supplementary file 6 — Supplementary Data 3 [file 42003_2021_1661_MOESM6_ESM.zip › Supplementary Data 3 corrected/K_03_C.jpg]

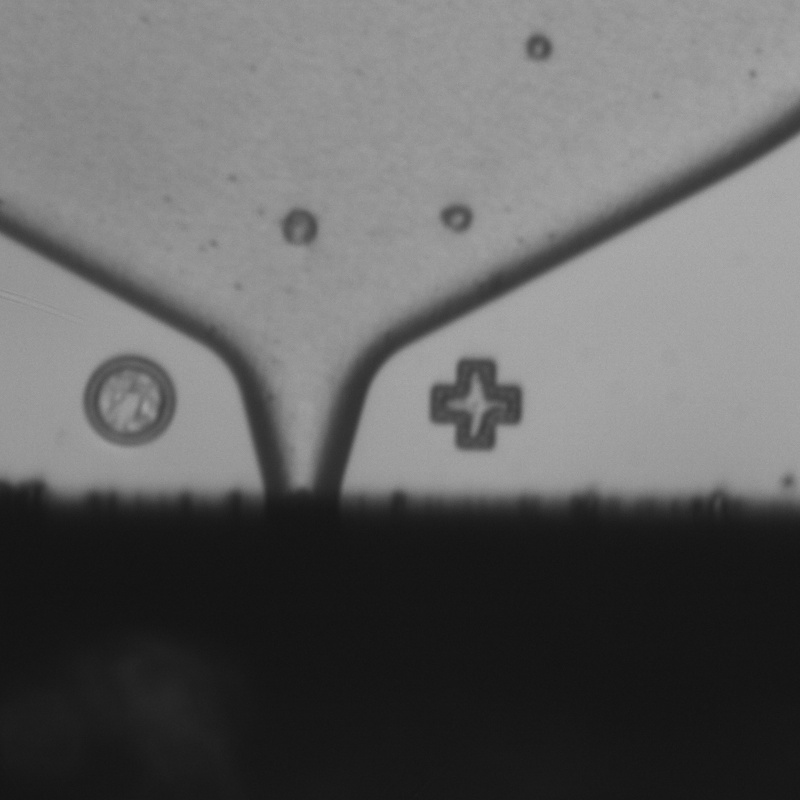

Supplement: Supplementary file 6 — Supplementary Data 3 [file 42003_2021_1661_MOESM6_ESM.zip › Supplementary Data 3 corrected/O_12_A.jpg]

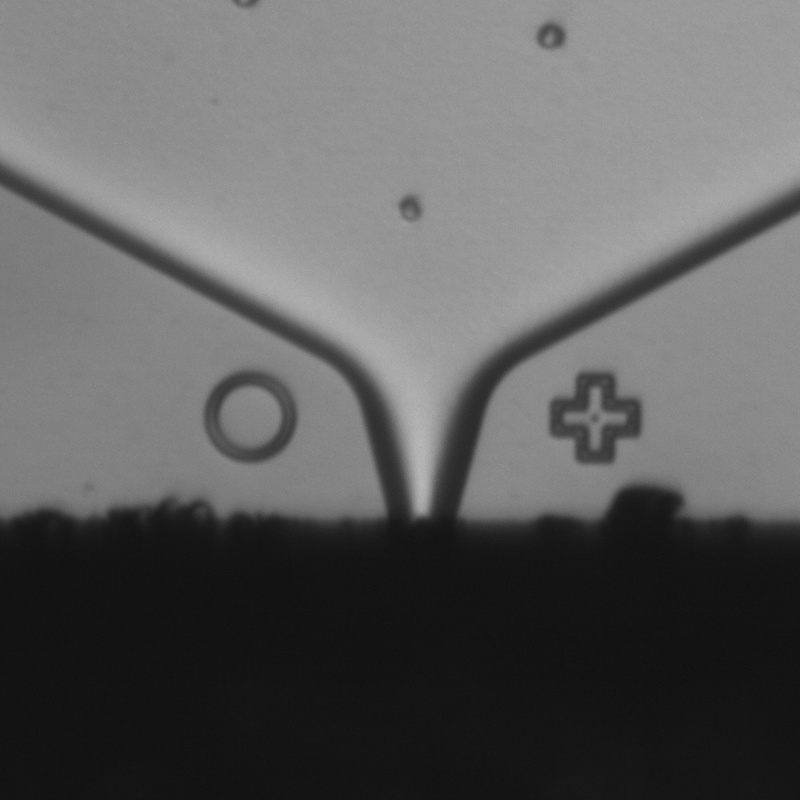

Supplement: Supplementary file 6 — Supplementary Data 3 [file 42003_2021_1661_MOESM6_ESM.zip › Supplementary Data 3 corrected/K_01_A.jpg]

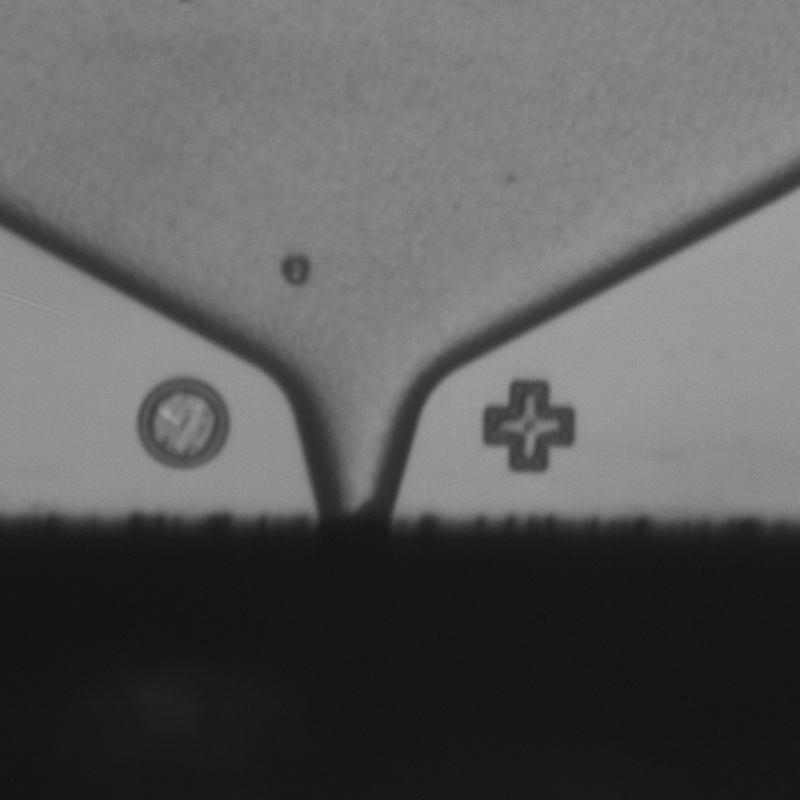

Supplement: Supplementary file 6 — Supplementary Data 3 [file 42003_2021_1661_MOESM6_ESM.zip › Supplementary Data 3 corrected/K_27_B.jpg]

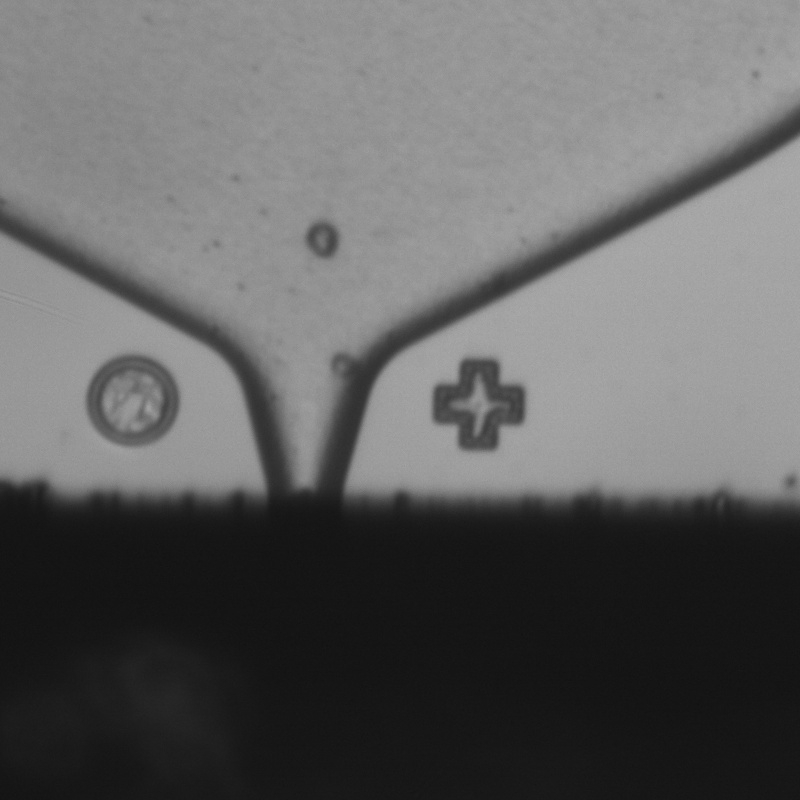

Supplement: Supplementary file 6 — Supplementary Data 3 [file 42003_2021_1661_MOESM6_ESM.zip › Supplementary Data 3 corrected/O_10_C.jpg]

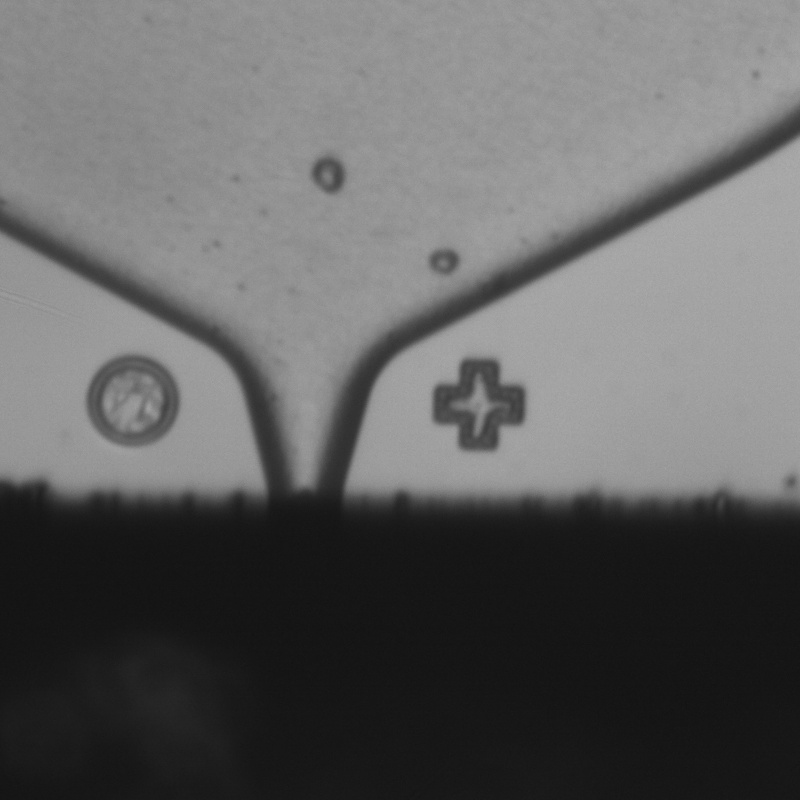

Supplement: Supplementary file 6 — Supplementary Data 3 [file 42003_2021_1661_MOESM6_ESM.zip › Supplementary Data 3 corrected/O_10_B.jpg]

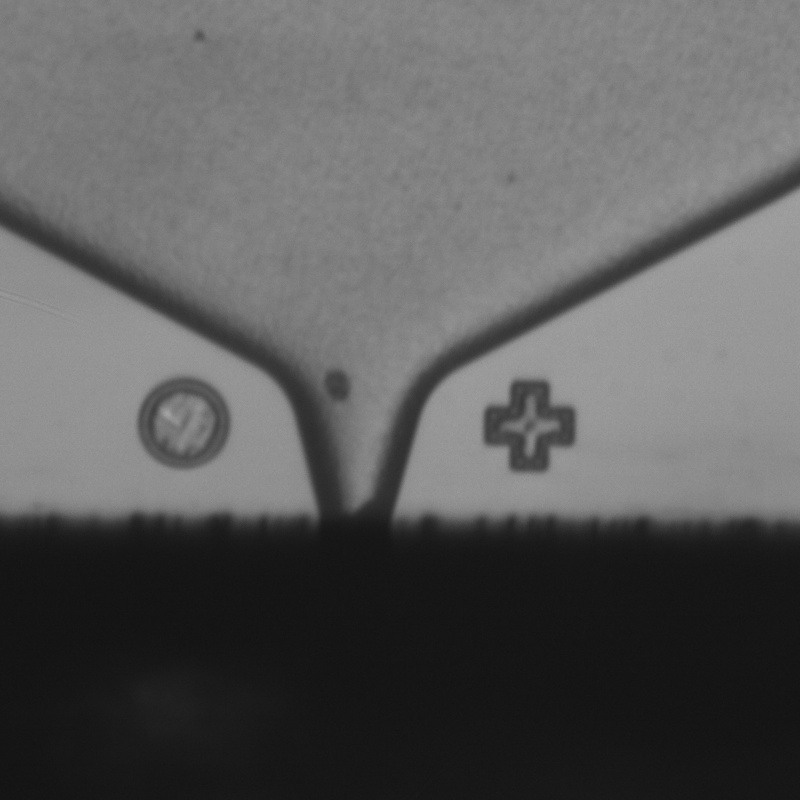

Supplement: Supplementary file 6 — Supplementary Data 3 [file 42003_2021_1661_MOESM6_ESM.zip › Supplementary Data 3 corrected/K_27_C.jpg]

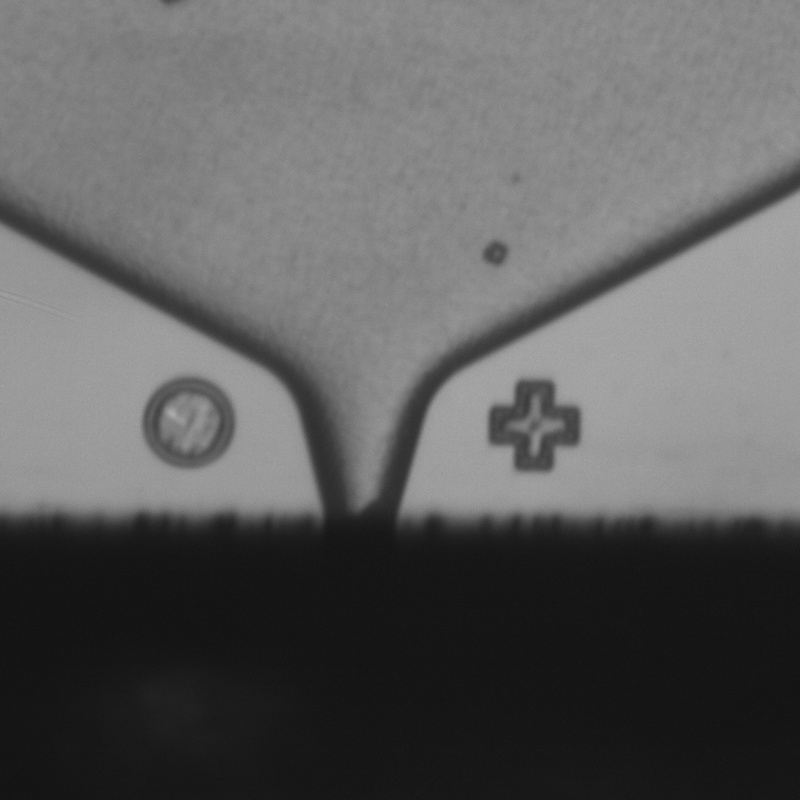

Supplement: Supplementary file 6 — Supplementary Data 3 [file 42003_2021_1661_MOESM6_ESM.zip › Supplementary Data 3 corrected/K_25_A.jpg]

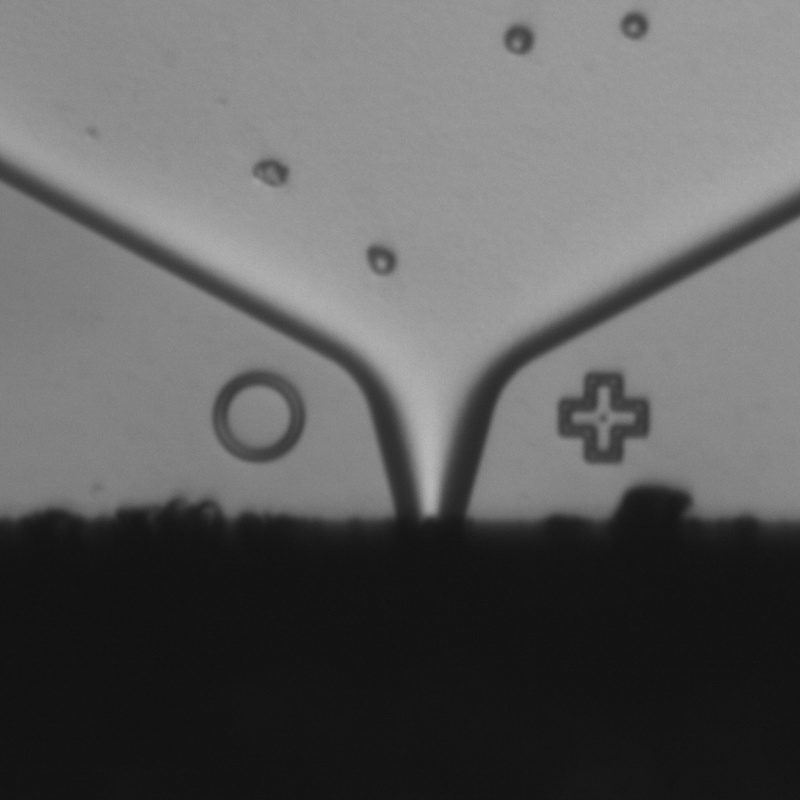

Supplement: Supplementary file 6 — Supplementary Data 3 [file 42003_2021_1661_MOESM6_ESM.zip › Supplementary Data 3 corrected/K_03_B.jpg]

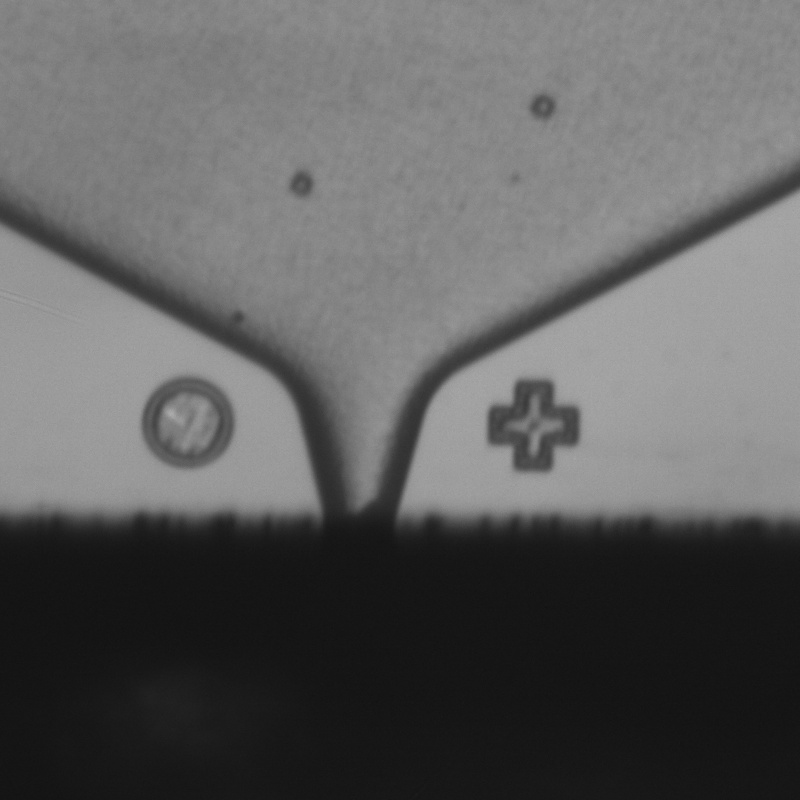

Supplement: Supplementary file 6 — Supplementary Data 3 [file 42003_2021_1661_MOESM6_ESM.zip › Supplementary Data 3 corrected/K_18_A.jpg]

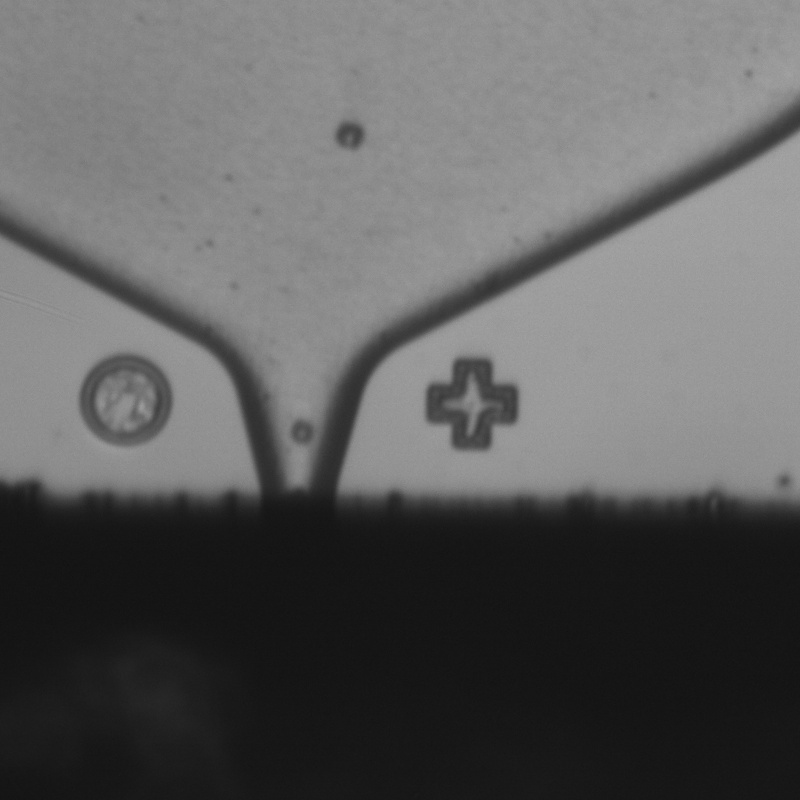

Supplement: Supplementary file 6 — Supplementary Data 3 [file 42003_2021_1661_MOESM6_ESM.zip › Supplementary Data 3 corrected/O_09_C.jpg]

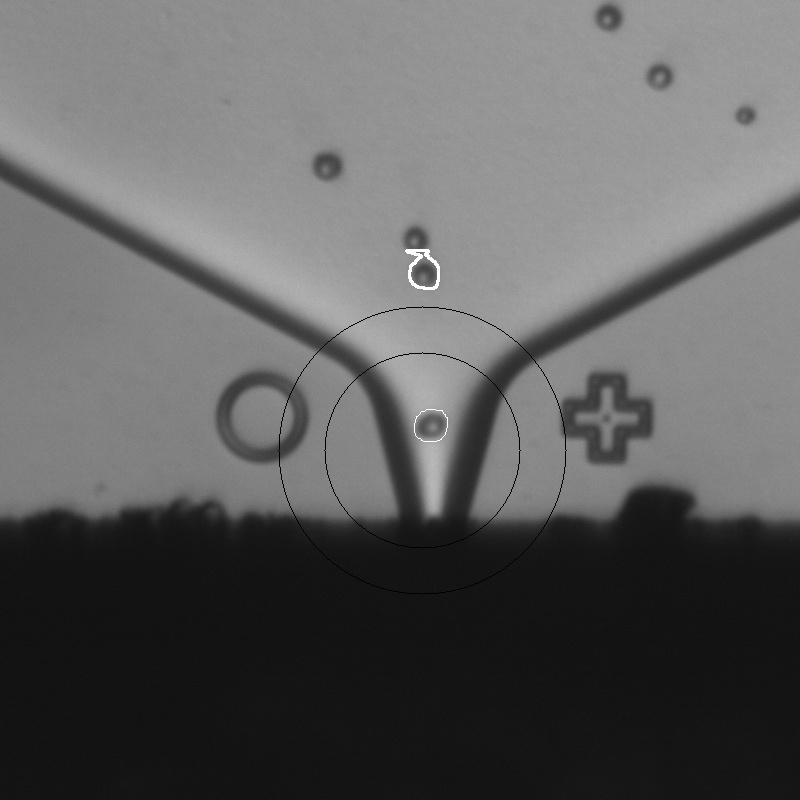

Supplement: Supplementary file 6 — Supplementary Data 3 [file 42003_2021_1661_MOESM6_ESM.zip › Supplementary Data 3 corrected/K_05_D.jpg]

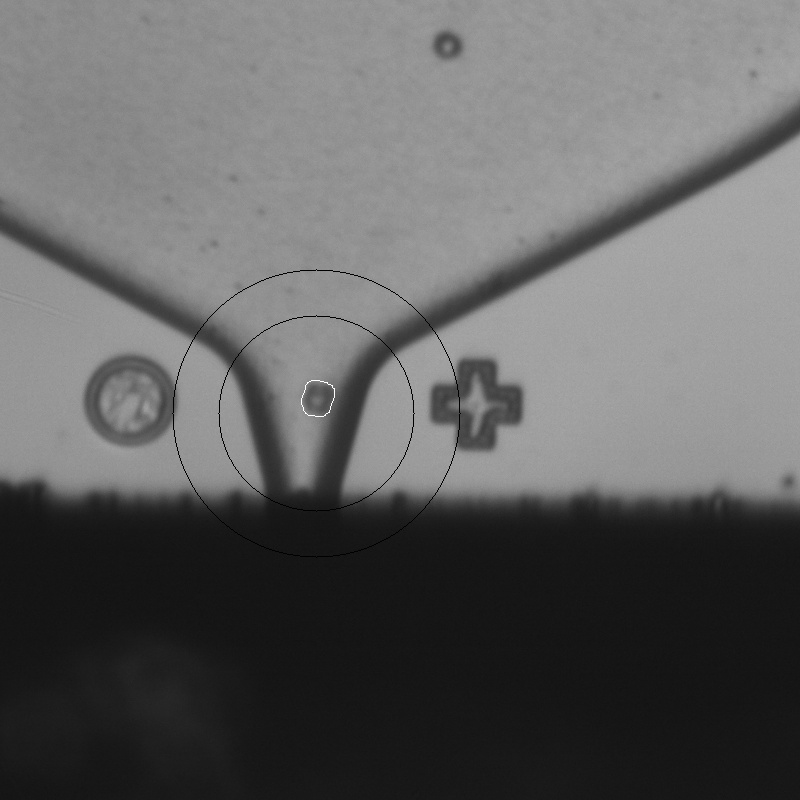

Supplement: Supplementary file 6 — Supplementary Data 3 [file 42003_2021_1661_MOESM6_ESM.zip › Supplementary Data 3 corrected/O_16_D.jpg]

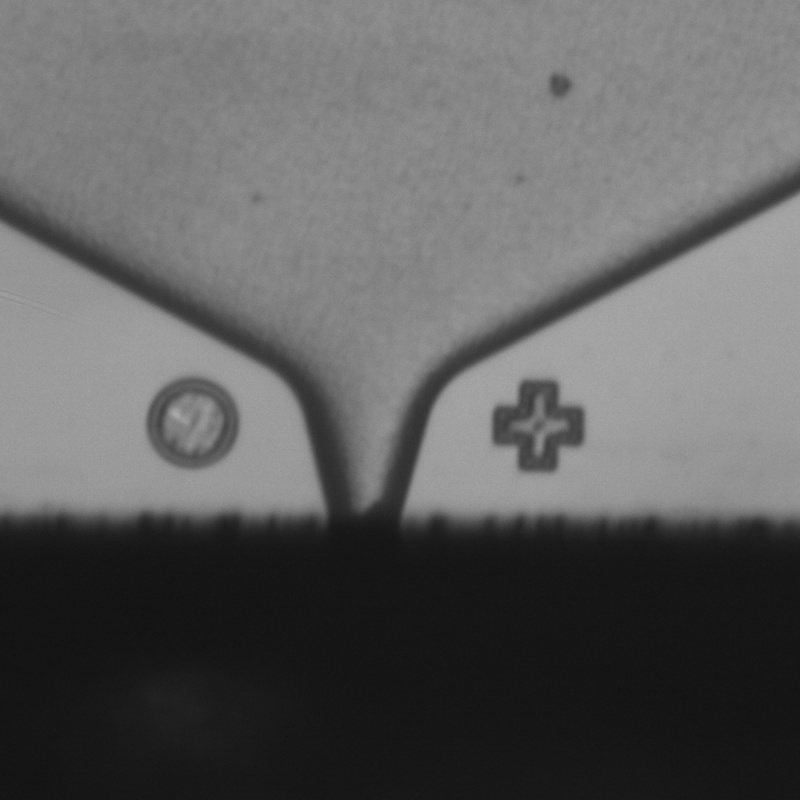

Supplement: Supplementary file 6 — Supplementary Data 3 [file 42003_2021_1661_MOESM6_ESM.zip › Supplementary Data 3 corrected/K_21_E.jpg]

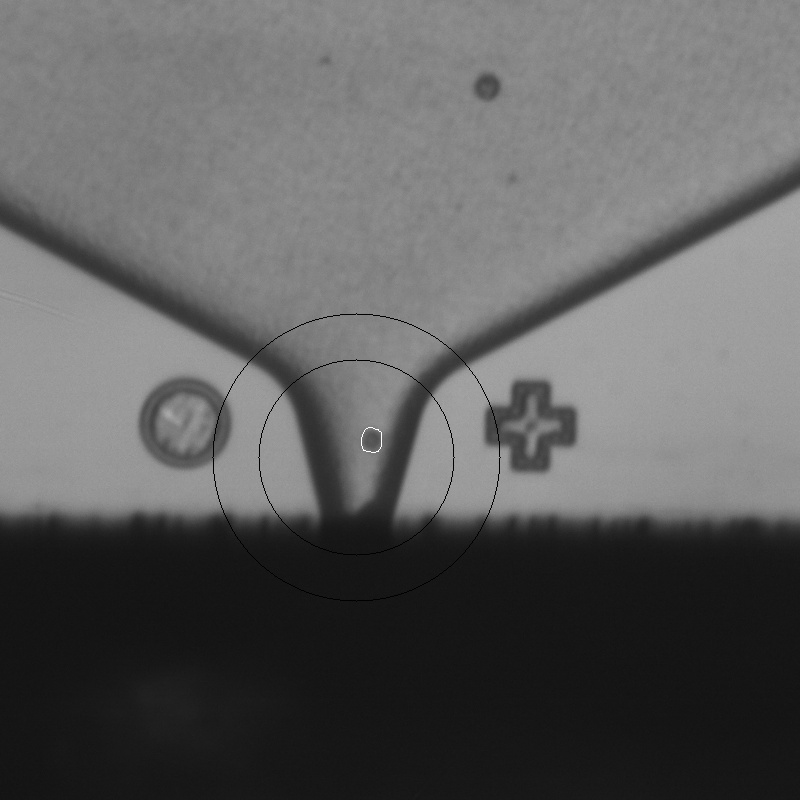

Supplement: Supplementary file 6 — Supplementary Data 3 [file 42003_2021_1661_MOESM6_ESM.zip › Supplementary Data 3 corrected/K_26_D.jpg]

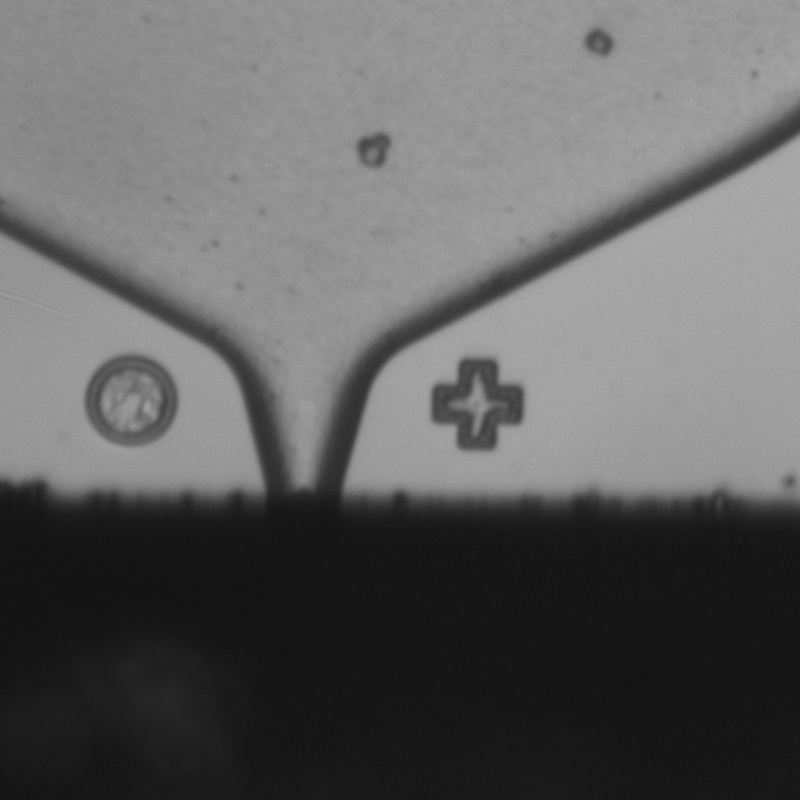

Supplement: Supplementary file 6 — Supplementary Data 3 [file 42003_2021_1661_MOESM6_ESM.zip › Supplementary Data 3 corrected/O_11_E.jpg]

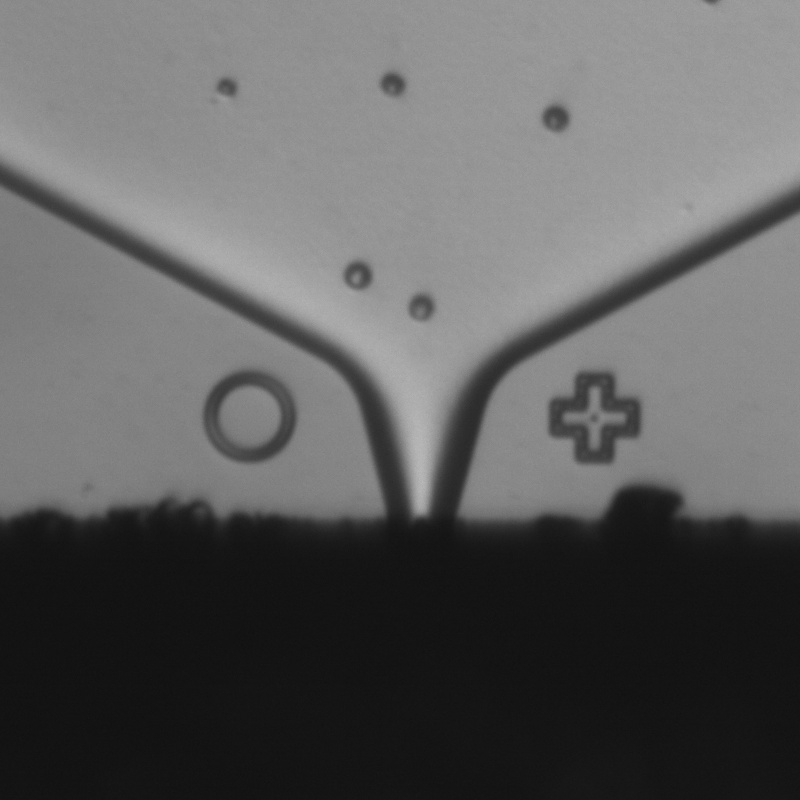

Supplement: Supplementary file 6 — Supplementary Data 3 [file 42003_2021_1661_MOESM6_ESM.zip › Supplementary Data 3 corrected/K_02_E.jpg]

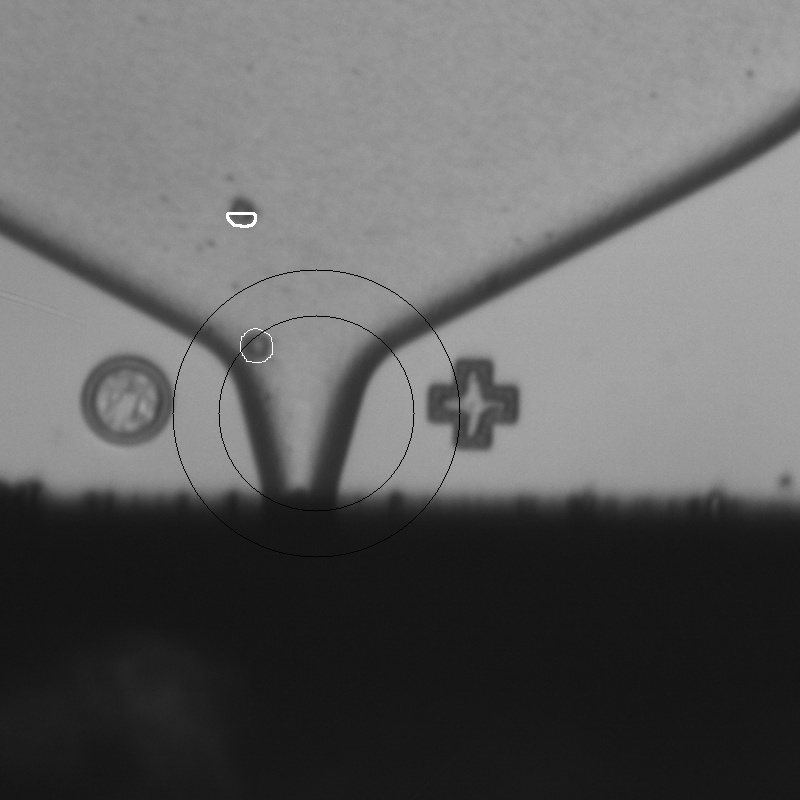

Supplement: Supplementary file 6 — Supplementary Data 3 [file 42003_2021_1661_MOESM6_ESM.zip › Supplementary Data 3 corrected/O_08_D.jpg]

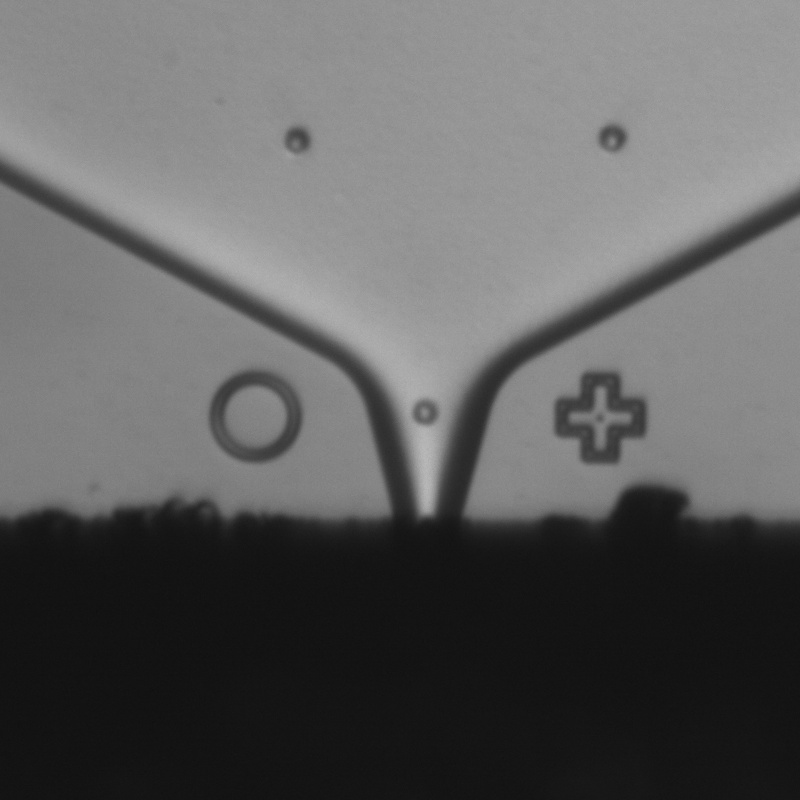

Supplement: Supplementary file 6 — Supplementary Data 3 [file 42003_2021_1661_MOESM6_ESM.zip › Supplementary Data 3 corrected/K_04_C.jpg]

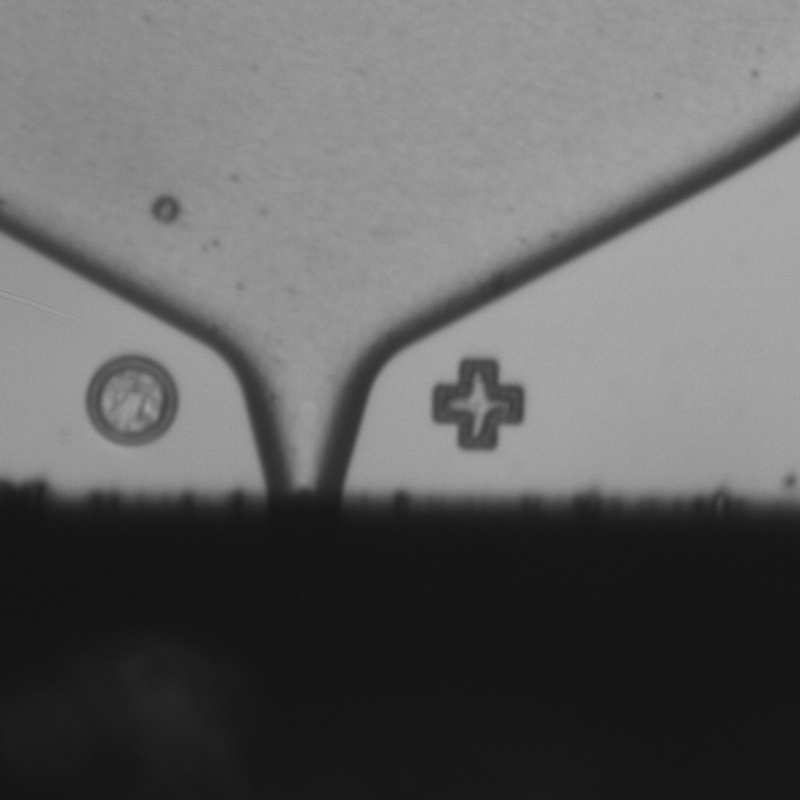

Supplement: Supplementary file 6 — Supplementary Data 3 [file 42003_2021_1661_MOESM6_ESM.zip › Supplementary Data 3 corrected/O_15_A.jpg]

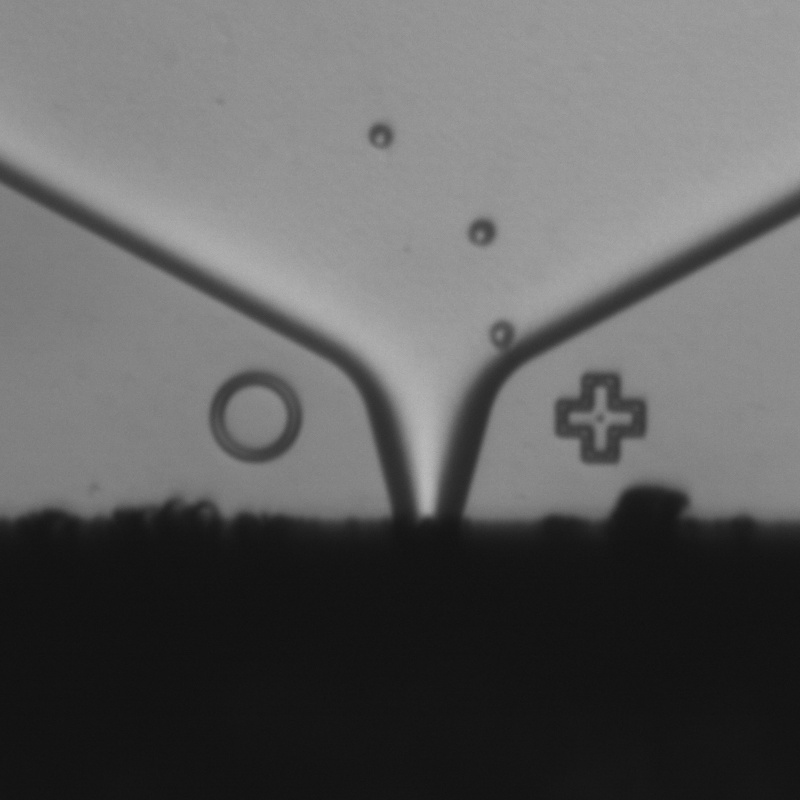

Supplement: Supplementary file 6 — Supplementary Data 3 [file 42003_2021_1661_MOESM6_ESM.zip › Supplementary Data 3 corrected/K_06_A.jpg]

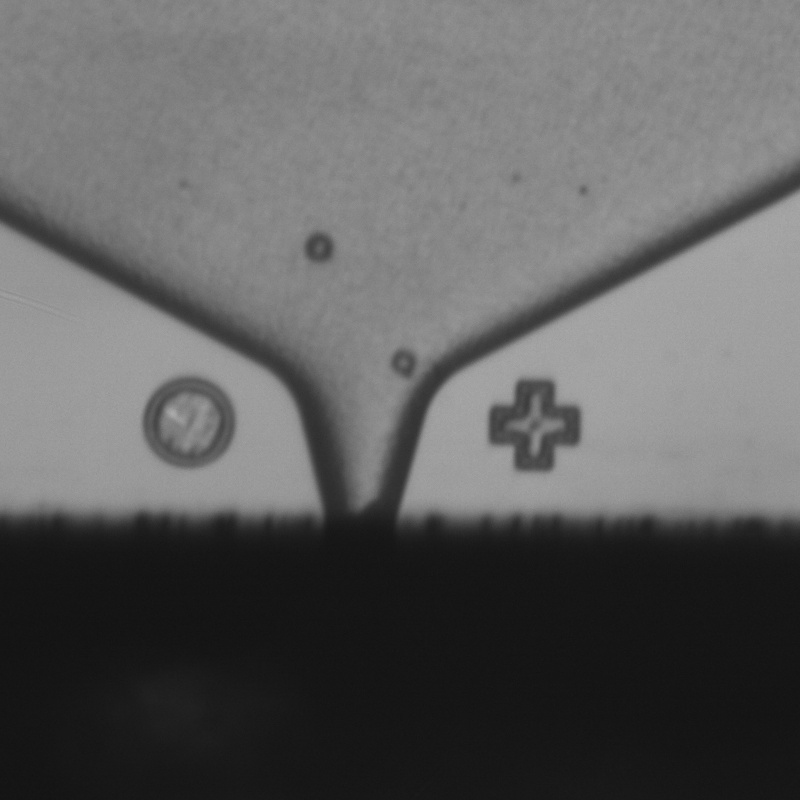

Supplement: Supplementary file 6 — Supplementary Data 3 [file 42003_2021_1661_MOESM6_ESM.zip › Supplementary Data 3 corrected/K_20_B.jpg]

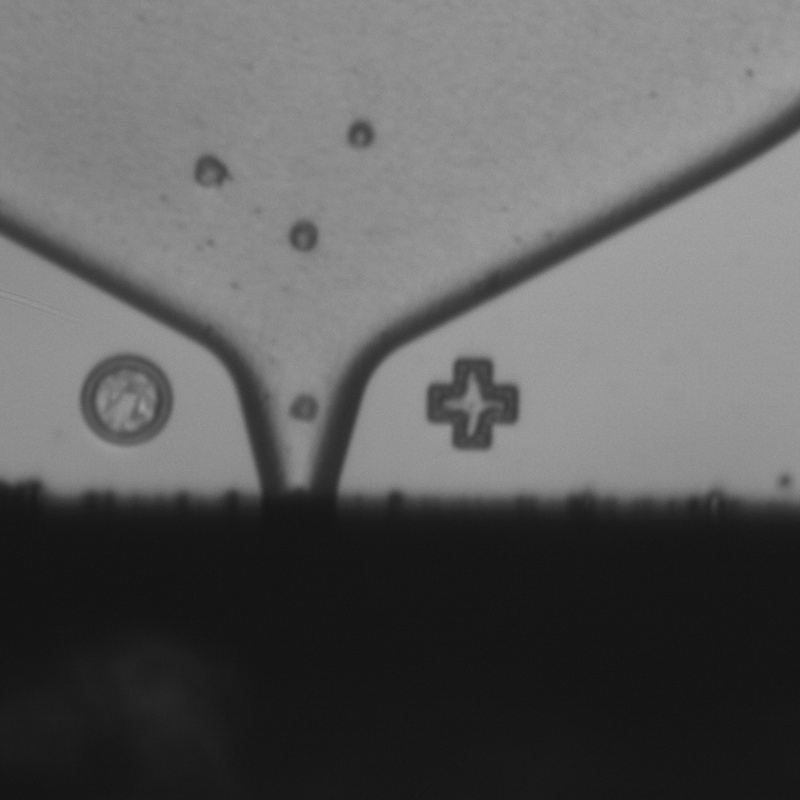

Supplement: Supplementary file 6 — Supplementary Data 3 [file 42003_2021_1661_MOESM6_ESM.zip › Supplementary Data 3 corrected/O_17_C.jpg]

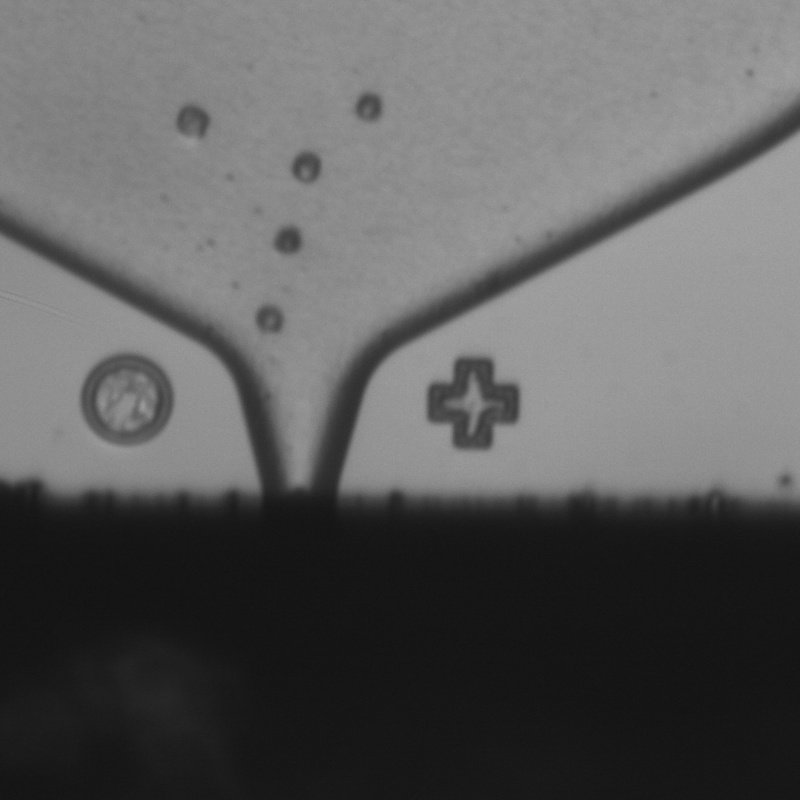

Supplement: Supplementary file 6 — Supplementary Data 3 [file 42003_2021_1661_MOESM6_ESM.zip › Supplementary Data 3 corrected/O_17_B.jpg]

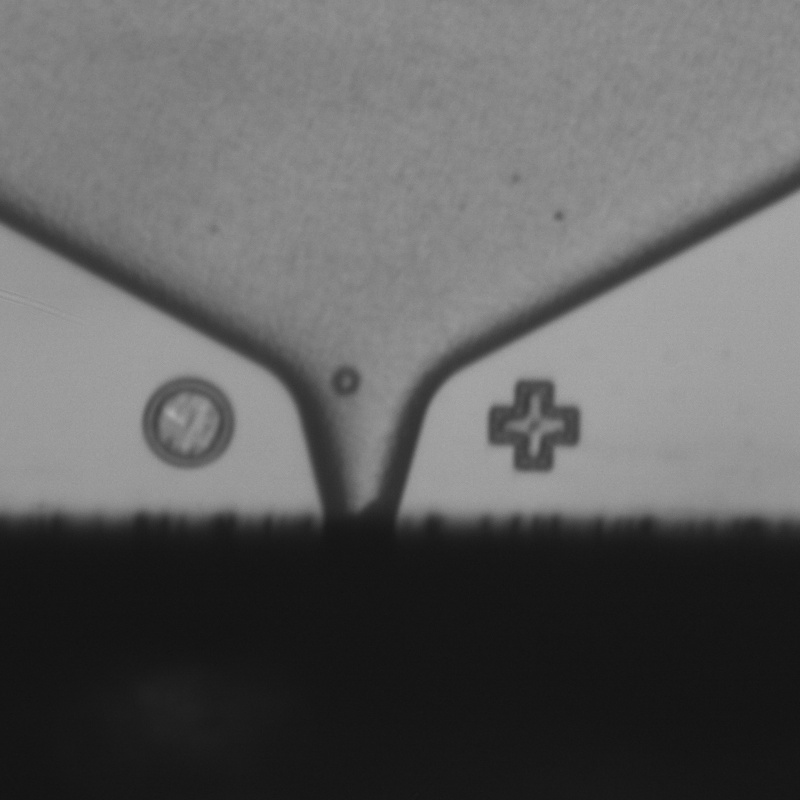

Supplement: Supplementary file 6 — Supplementary Data 3 [file 42003_2021_1661_MOESM6_ESM.zip › Supplementary Data 3 corrected/K_20_C.jpg]

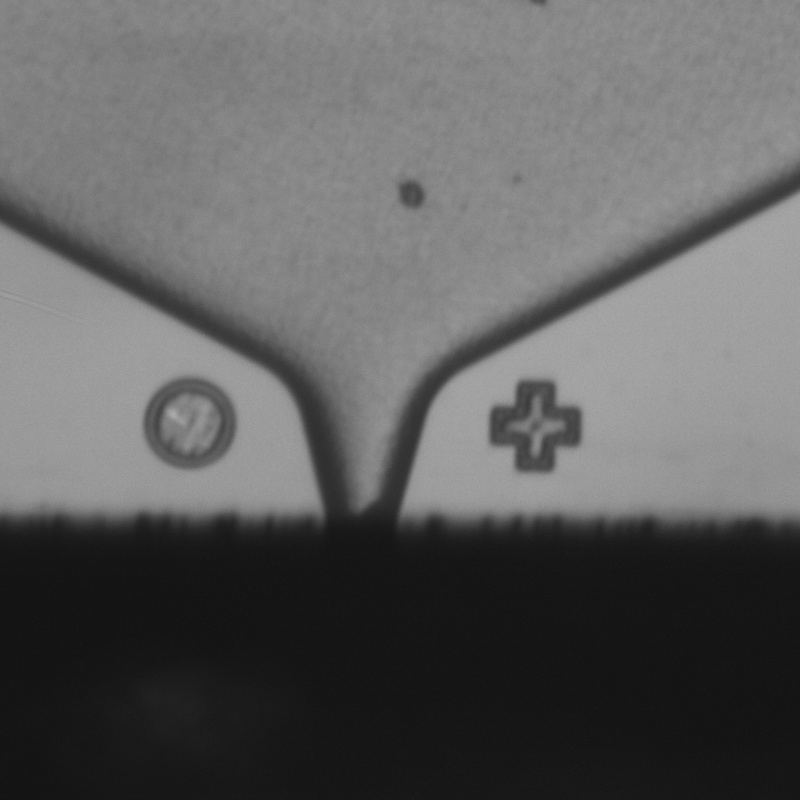

Supplement: Supplementary file 6 — Supplementary Data 3 [file 42003_2021_1661_MOESM6_ESM.zip › Supplementary Data 3 corrected/K_22_A.jpg]

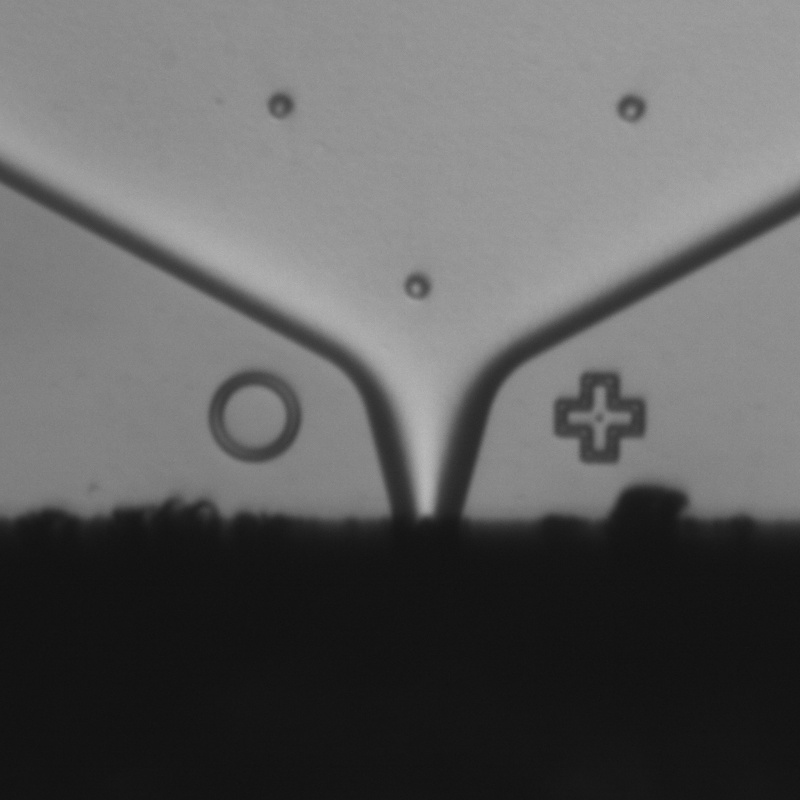

Supplement: Supplementary file 6 — Supplementary Data 3 [file 42003_2021_1661_MOESM6_ESM.zip › Supplementary Data 3 corrected/K_04_B.jpg]

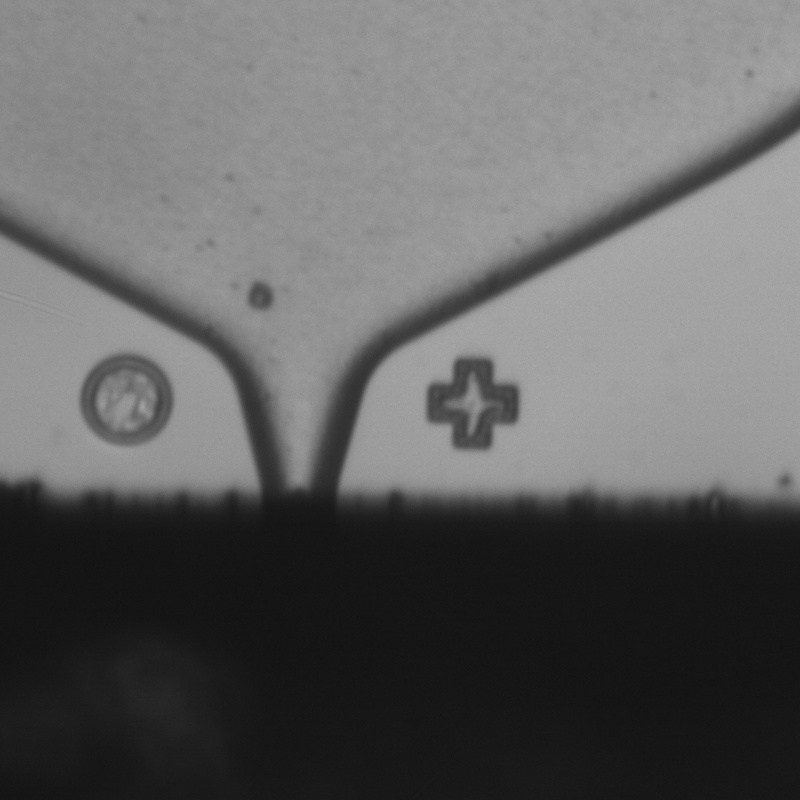

Supplement: Supplementary file 6 — Supplementary Data 3 [file 42003_2021_1661_MOESM6_ESM.zip › Supplementary Data 3 corrected/O_08_E.jpg]

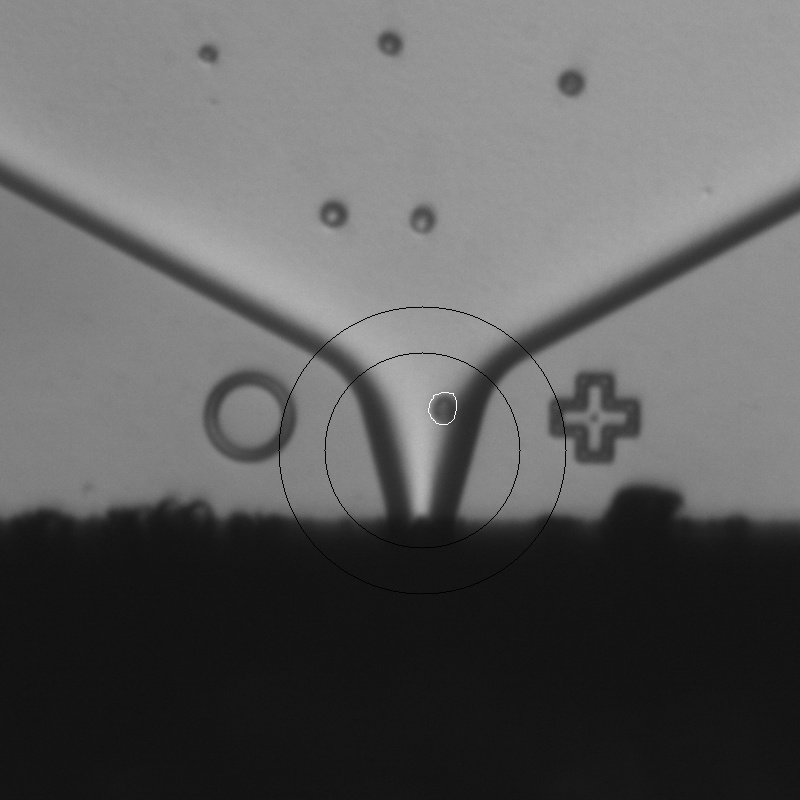

Supplement: Supplementary file 6 — Supplementary Data 3 [file 42003_2021_1661_MOESM6_ESM.zip › Supplementary Data 3 corrected/K_02_D.jpg]

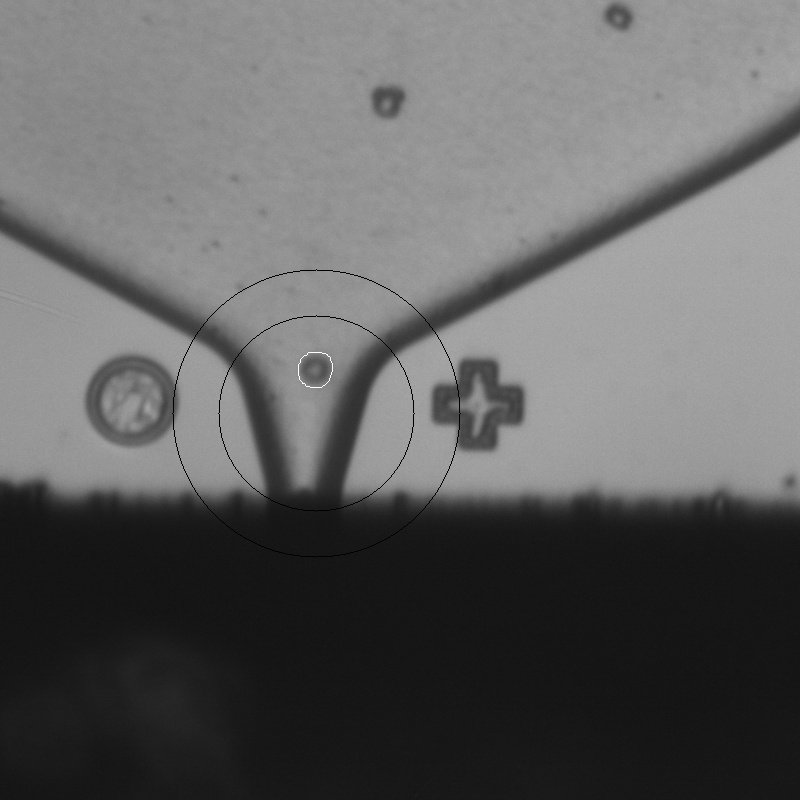

Supplement: Supplementary file 6 — Supplementary Data 3 [file 42003_2021_1661_MOESM6_ESM.zip › Supplementary Data 3 corrected/O_11_D.jpg]

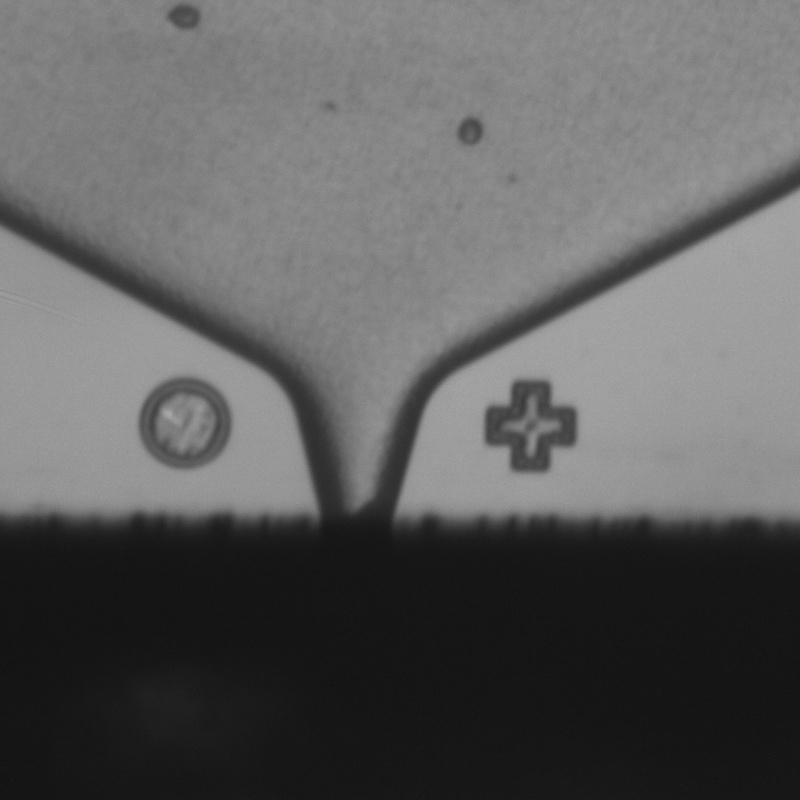

Supplement: Supplementary file 6 — Supplementary Data 3 [file 42003_2021_1661_MOESM6_ESM.zip › Supplementary Data 3 corrected/K_26_E.jpg]

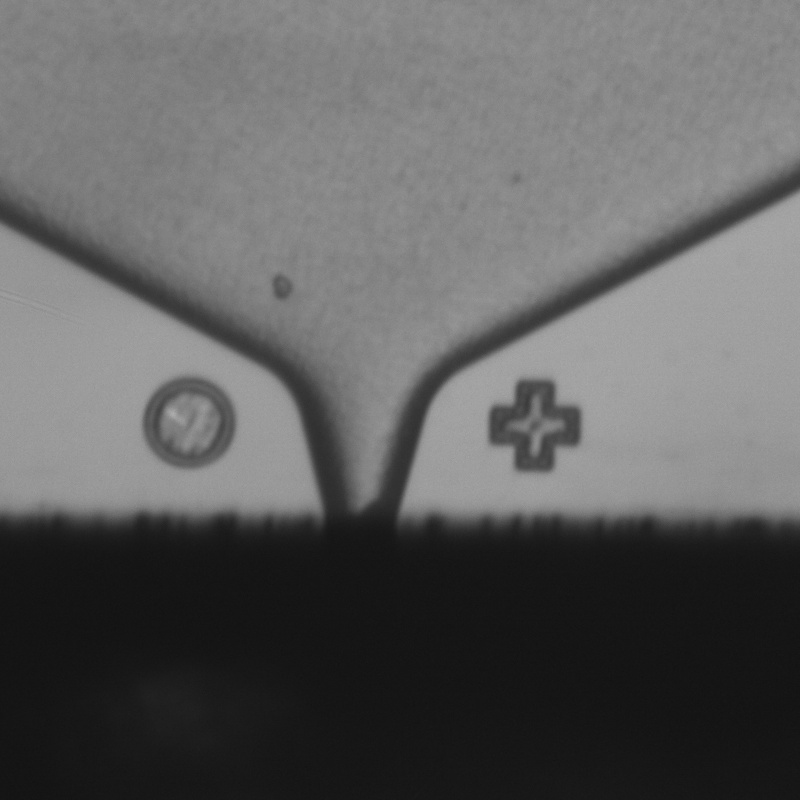

Supplement: Supplementary file 6 — Supplementary Data 3 [file 42003_2021_1661_MOESM6_ESM.zip › Supplementary Data 3 corrected/K_24_E.jpg]

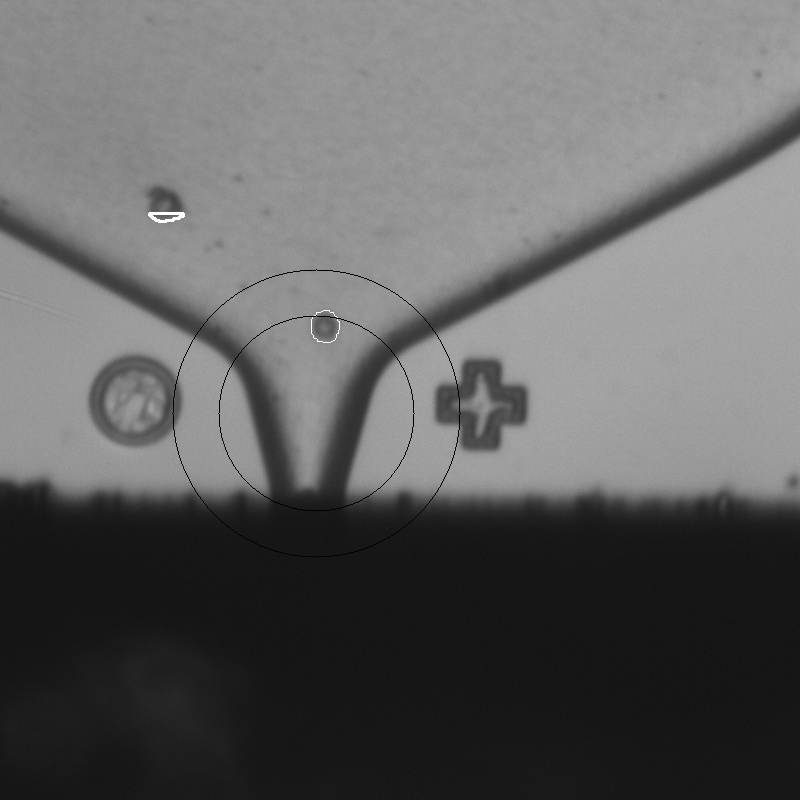

Supplement: Supplementary file 6 — Supplementary Data 3 [file 42003_2021_1661_MOESM6_ESM.zip › Supplementary Data 3 corrected/O_13_D.jpg]

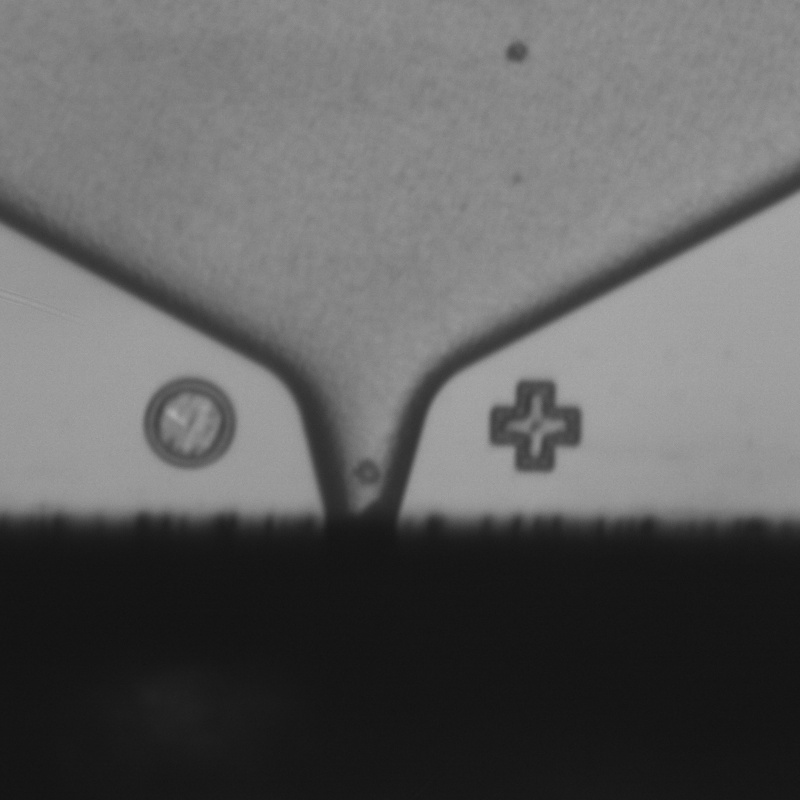

Supplement: Supplementary file 6 — Supplementary Data 3 [file 42003_2021_1661_MOESM6_ESM.zip › Supplementary Data 3 corrected/K_22_C.jpg]

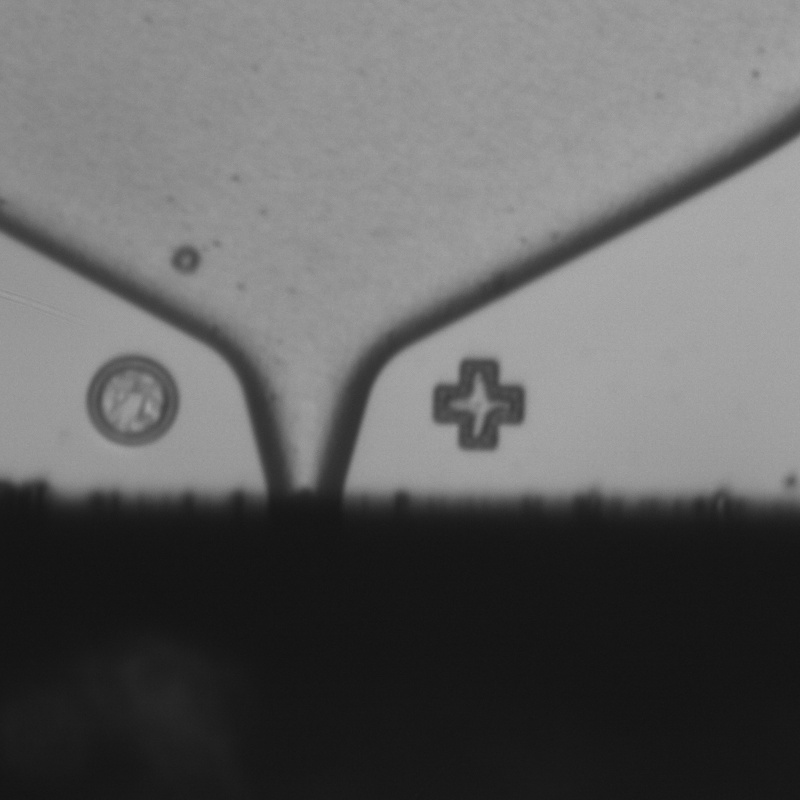

Supplement: Supplementary file 6 — Supplementary Data 3 [file 42003_2021_1661_MOESM6_ESM.zip › Supplementary Data 3 corrected/O_15_B.jpg]

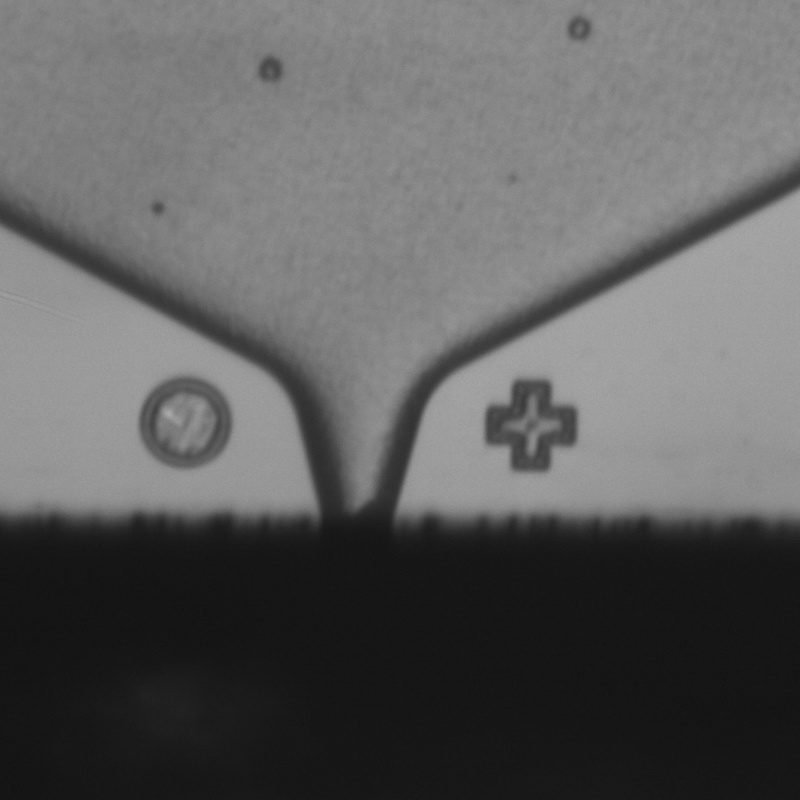

Supplement: Supplementary file 6 — Supplementary Data 3 [file 42003_2021_1661_MOESM6_ESM.zip › Supplementary Data 3 corrected/K_19_E.jpg]

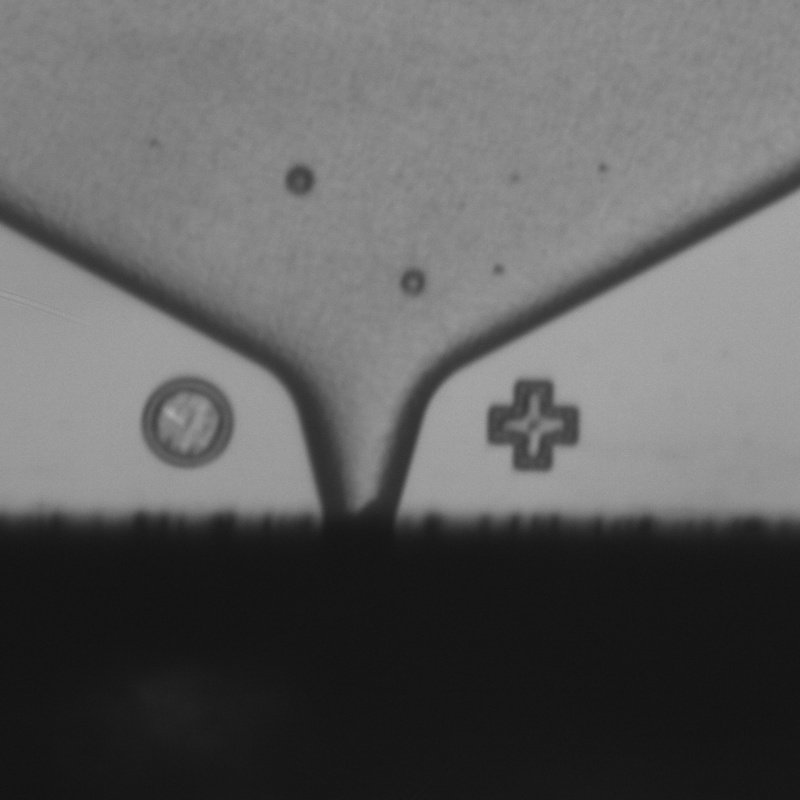

Supplement: Supplementary file 6 — Supplementary Data 3 [file 42003_2021_1661_MOESM6_ESM.zip › Supplementary Data 3 corrected/K_20_A.jpg]
