# Supplementary figures and images for: Bisulfite-free epigenomics and genomics of single cells through methylation-sensitive restriction (part 3 of 3)
Source: Commun Biol. 2021 Feb 1;4:153. doi: 10.1038/s42003-021-01661-w (PMC7851132; doi:10.1038/s42003-021-01661-w)

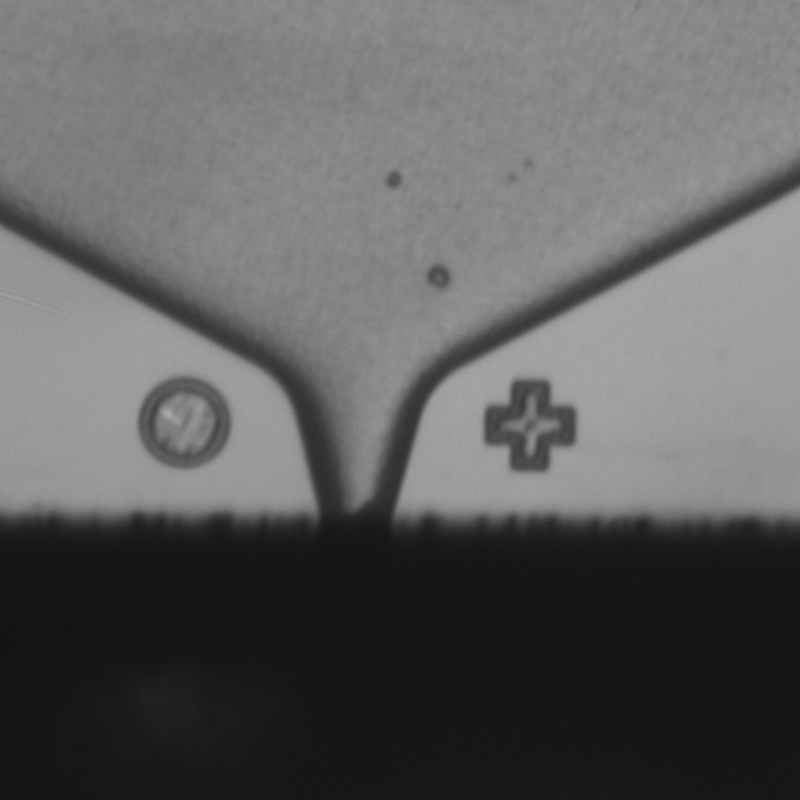

Supplement: Supplementary file 6 — Supplementary Data 3 [file 42003_2021_1661_MOESM6_ESM.zip › Supplementary Data 3 corrected/K_16_B.jpg]

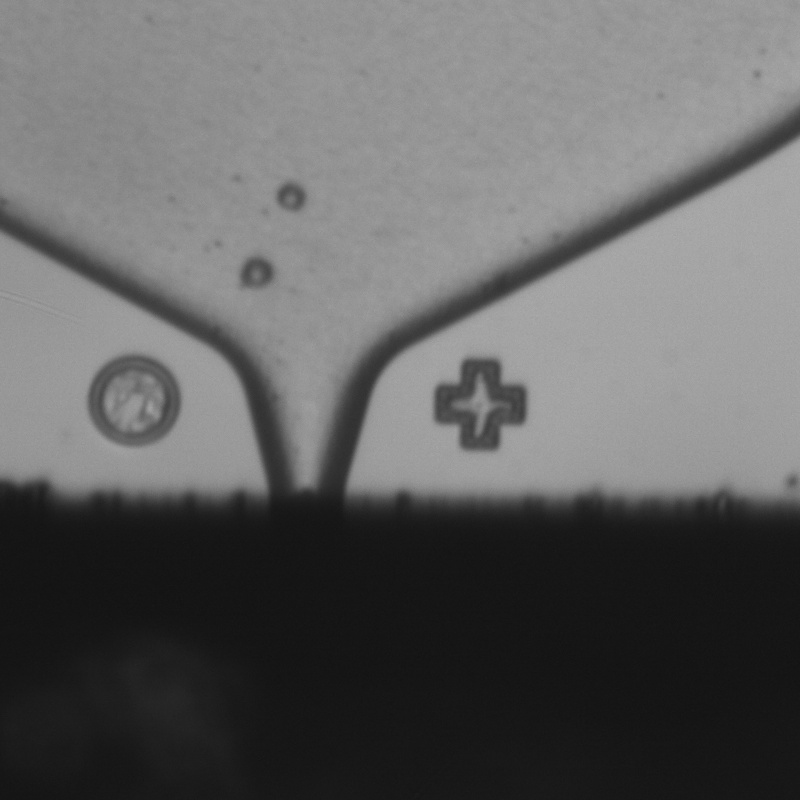

Supplement: Supplementary file 6 — Supplementary Data 3 [file 42003_2021_1661_MOESM6_ESM.zip › Supplementary Data 3 corrected/O_05_B.jpg]

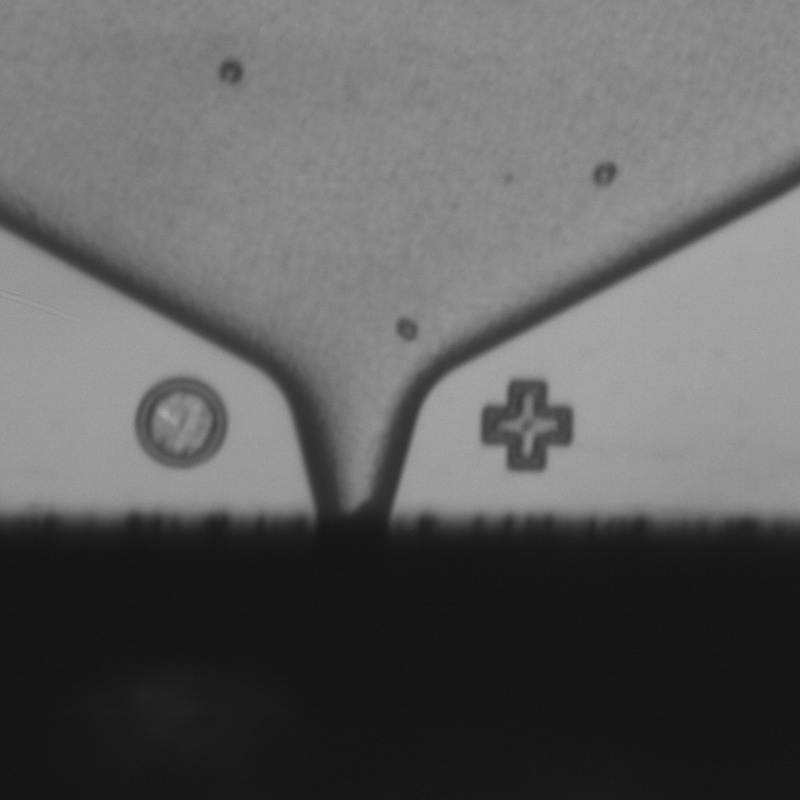

Supplement: Supplementary file 6 — Supplementary Data 3 [file 42003_2021_1661_MOESM6_ESM.zip › Supplementary Data 3 corrected/K_09_E.jpg]

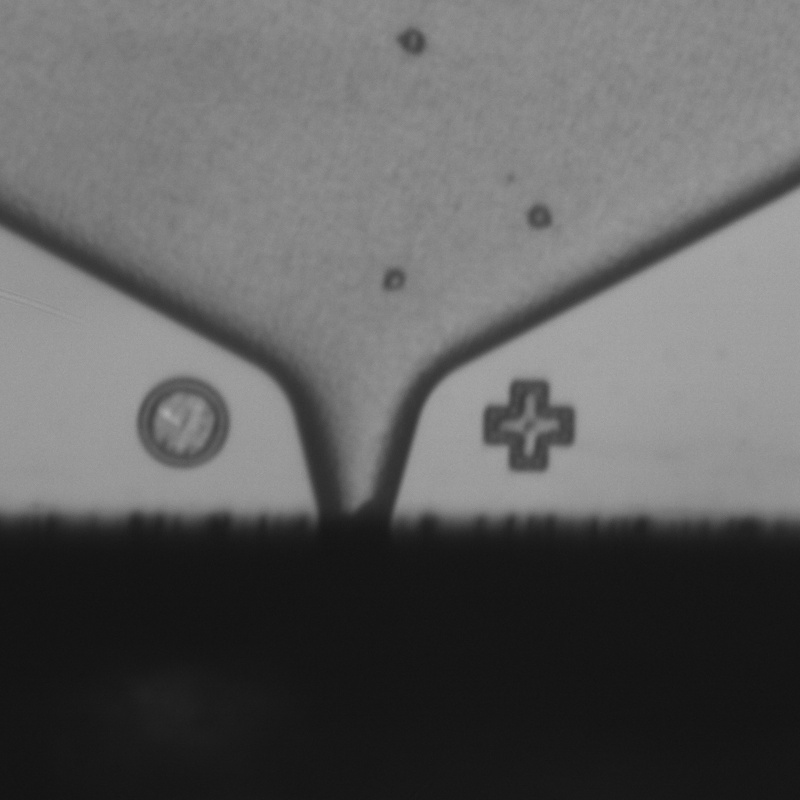

Supplement: Supplementary file 6 — Supplementary Data 3 [file 42003_2021_1661_MOESM6_ESM.zip › Supplementary Data 3 corrected/K_14_B.jpg]

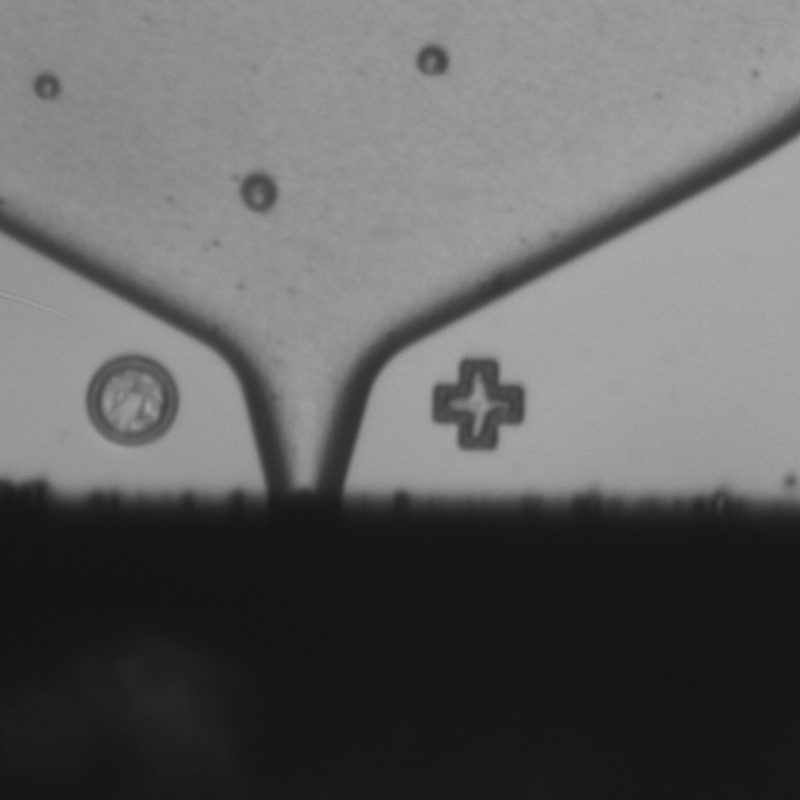

Supplement: Supplementary file 6 — Supplementary Data 3 [file 42003_2021_1661_MOESM6_ESM.zip › Supplementary Data 3 corrected/O_18_E.jpg]

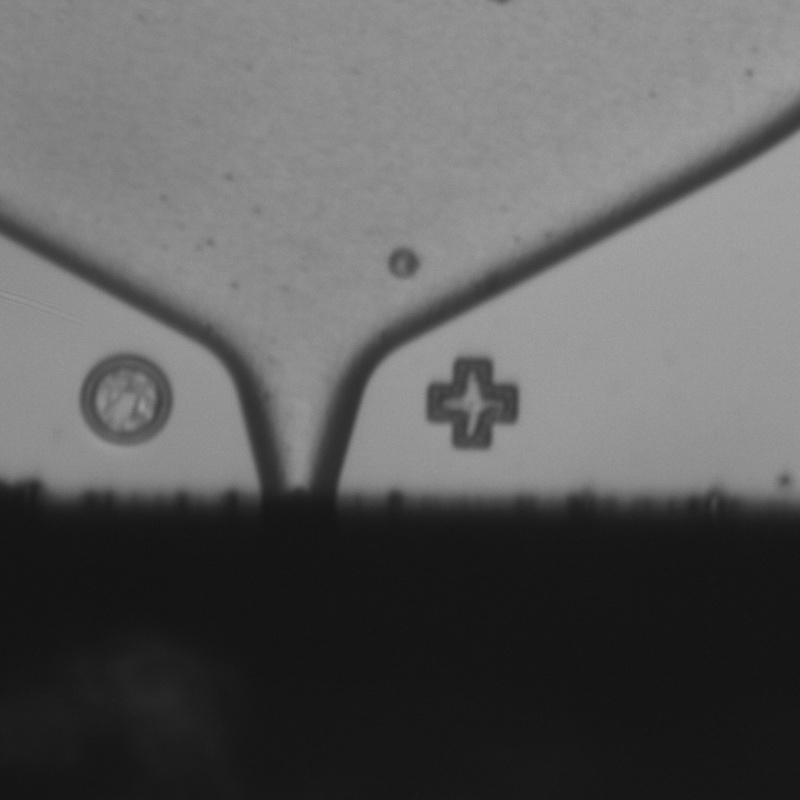

Supplement: Supplementary file 6 — Supplementary Data 3 [file 42003_2021_1661_MOESM6_ESM.zip › Supplementary Data 3 corrected/O_07_B.jpg]
