# Supplementary figures and images for: Bisulfite-free epigenomics and genomics of single cells through methylation-sensitive restriction (part 2 of 3)
Source: Commun Biol. 2021 Feb 1;4:153. doi: 10.1038/s42003-021-01661-w (PMC7851132; doi:10.1038/s42003-021-01661-w)

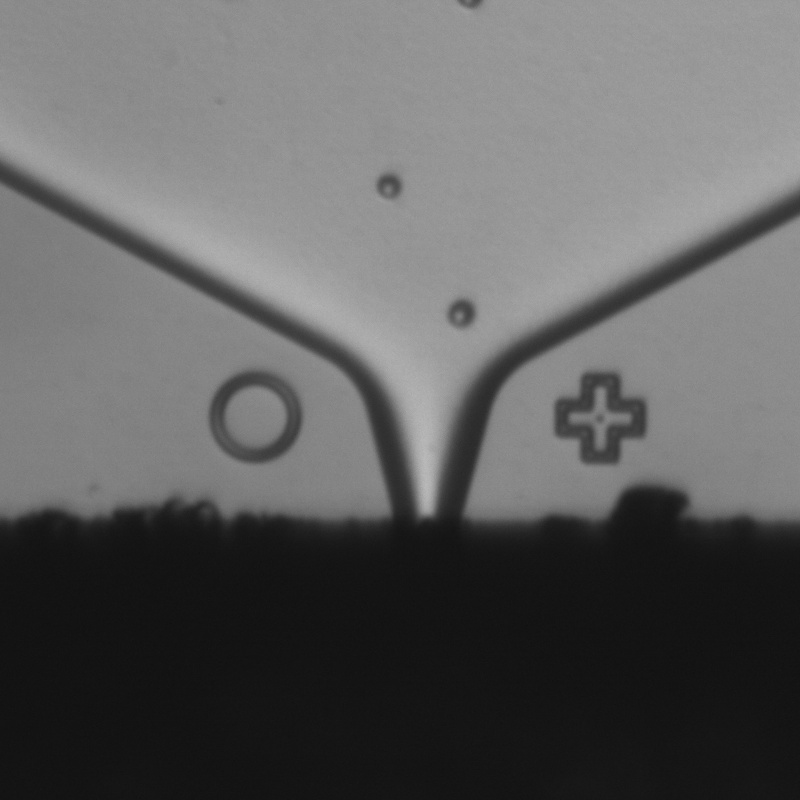

Supplement: Supplementary file 6 — Supplementary Data 3 [file 42003_2021_1661_MOESM6_ESM.zip › Supplementary Data 3 corrected/K_06_B.jpg]

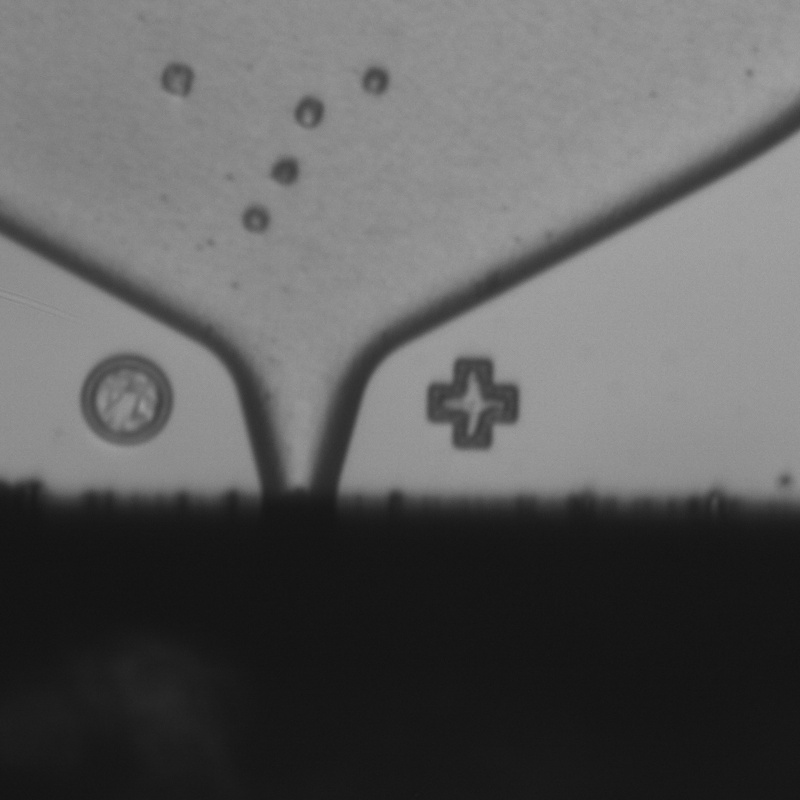

Supplement: Supplementary file 6 — Supplementary Data 3 [file 42003_2021_1661_MOESM6_ESM.zip › Supplementary Data 3 corrected/O_17_A.jpg]

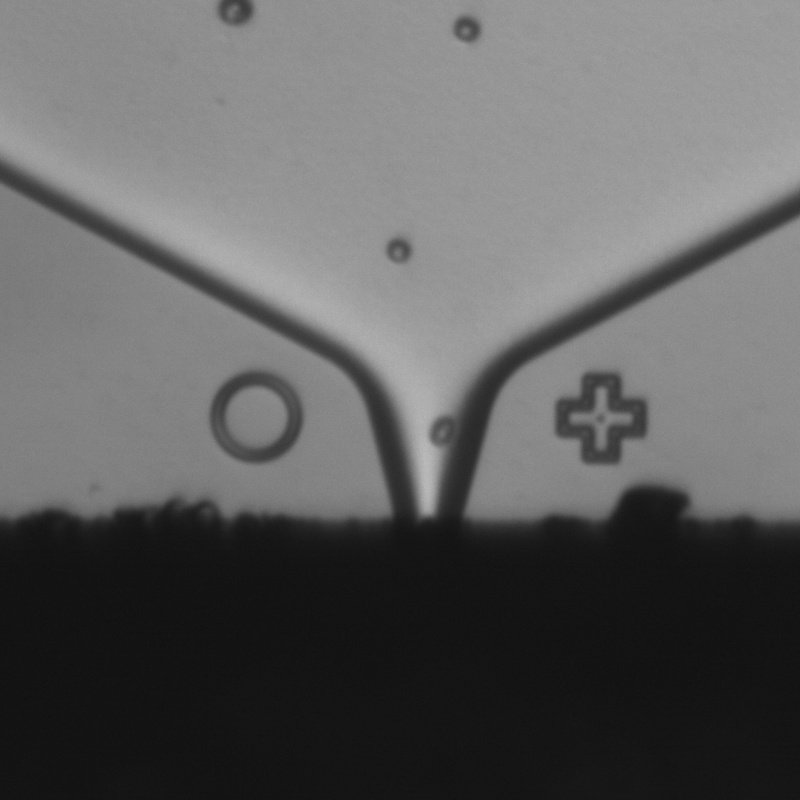

Supplement: Supplementary file 6 — Supplementary Data 3 [file 42003_2021_1661_MOESM6_ESM.zip › Supplementary Data 3 corrected/K_06_C.jpg]

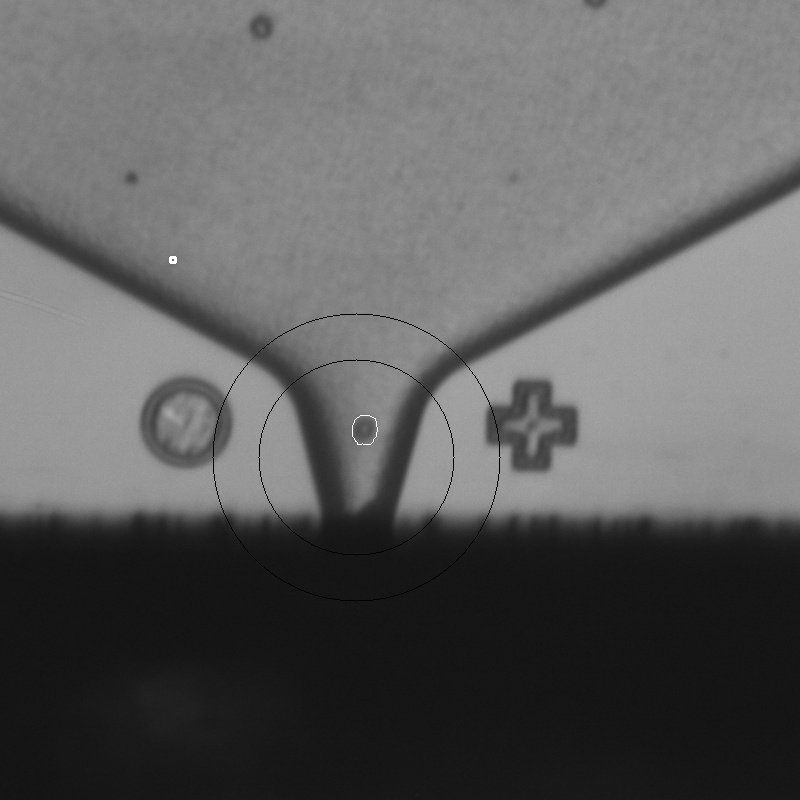

Supplement: Supplementary file 6 — Supplementary Data 3 [file 42003_2021_1661_MOESM6_ESM.zip › Supplementary Data 3 corrected/K_19_D.jpg]

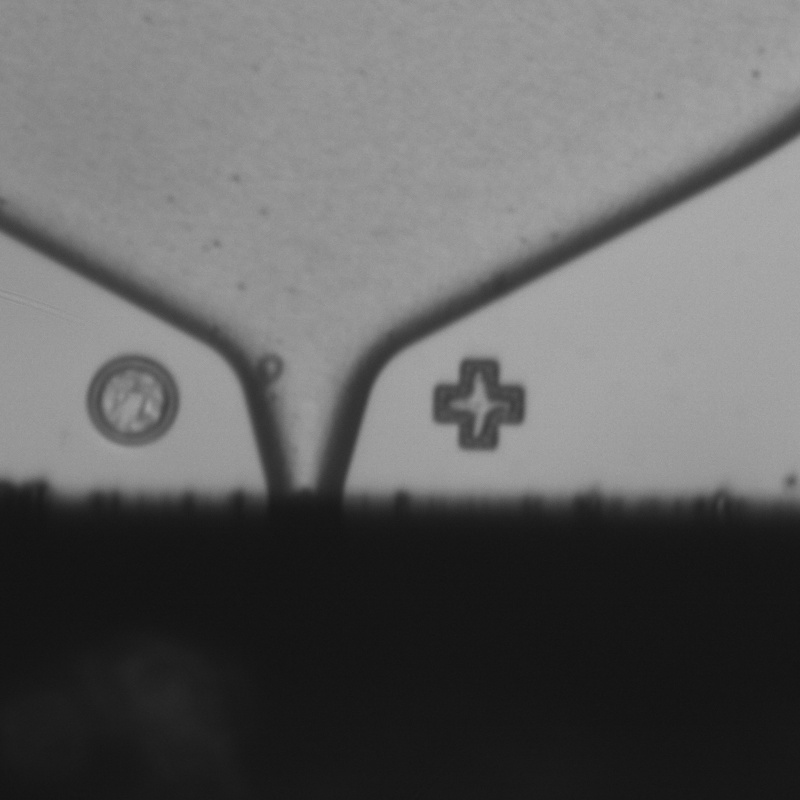

Supplement: Supplementary file 6 — Supplementary Data 3 [file 42003_2021_1661_MOESM6_ESM.zip › Supplementary Data 3 corrected/O_15_C.jpg]

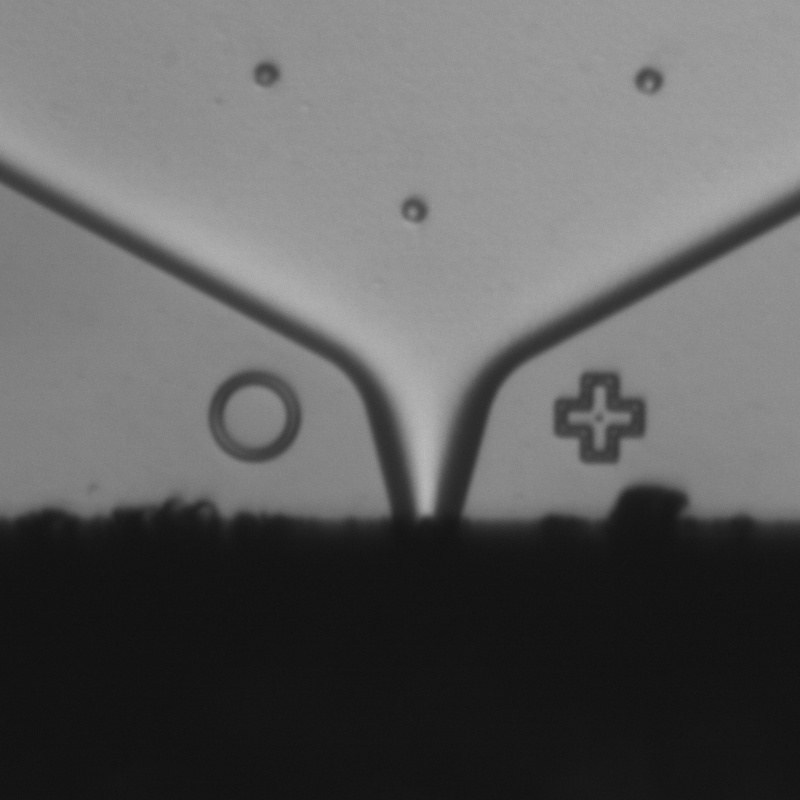

Supplement: Supplementary file 6 — Supplementary Data 3 [file 42003_2021_1661_MOESM6_ESM.zip › Supplementary Data 3 corrected/K_04_A.jpg]

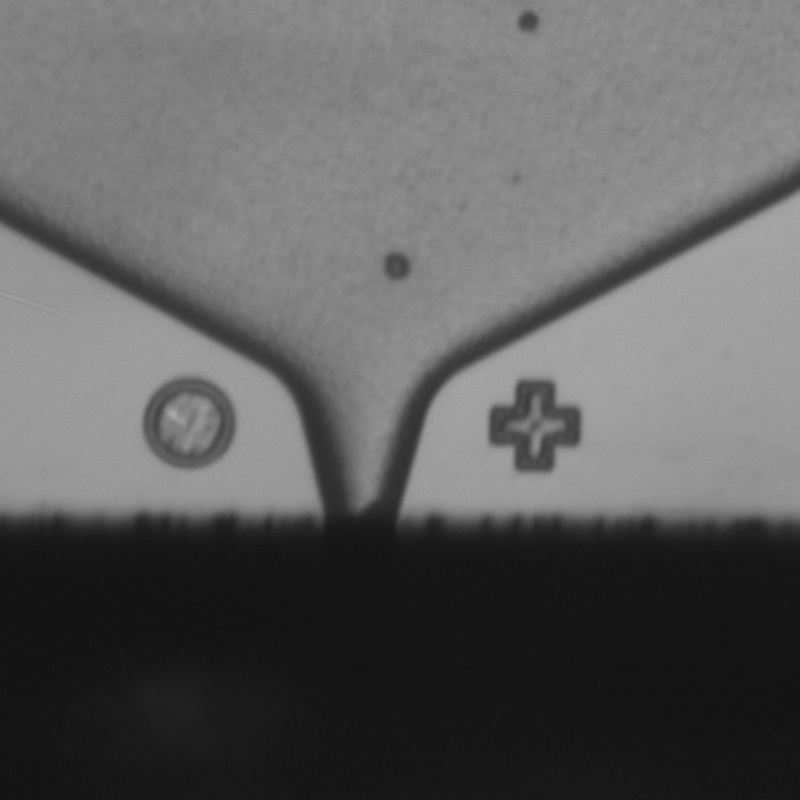

Supplement: Supplementary file 6 — Supplementary Data 3 [file 42003_2021_1661_MOESM6_ESM.zip › Supplementary Data 3 corrected/K_22_B.jpg]

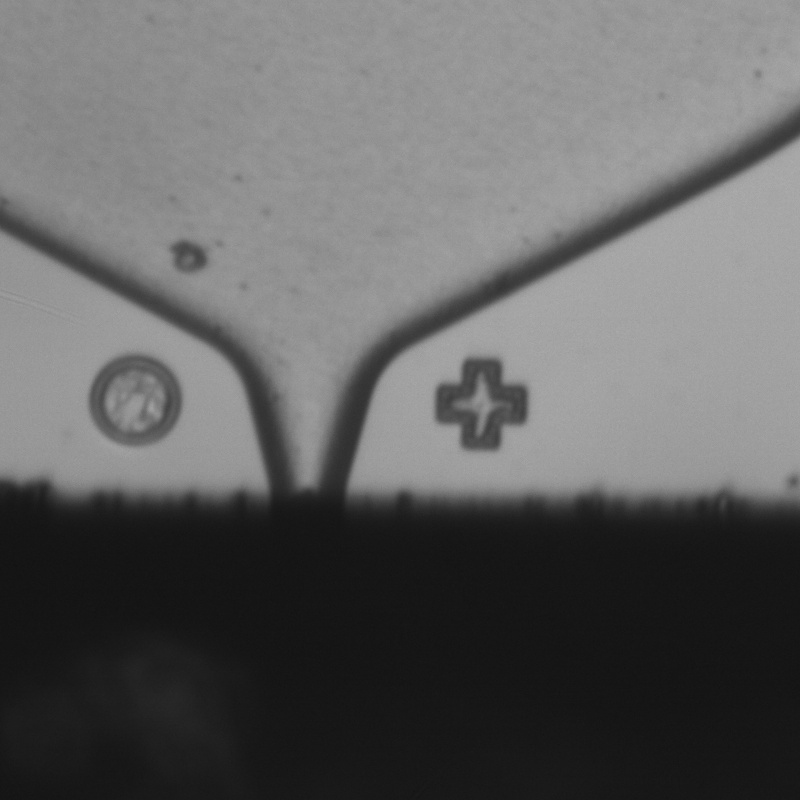

Supplement: Supplementary file 6 — Supplementary Data 3 [file 42003_2021_1661_MOESM6_ESM.zip › Supplementary Data 3 corrected/O_13_E.jpg]

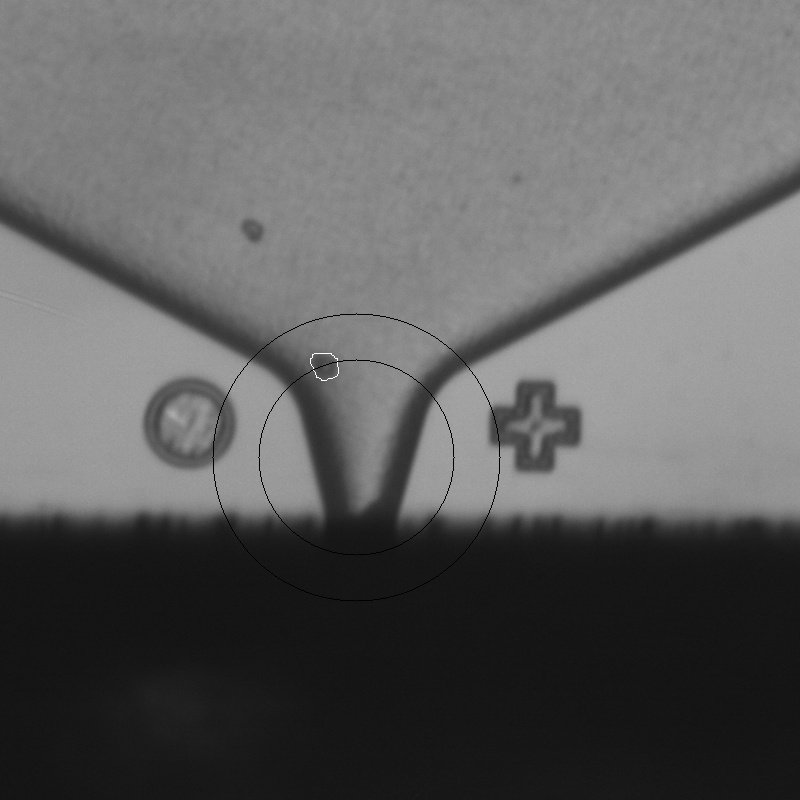

Supplement: Supplementary file 6 — Supplementary Data 3 [file 42003_2021_1661_MOESM6_ESM.zip › Supplementary Data 3 corrected/K_24_D.jpg]

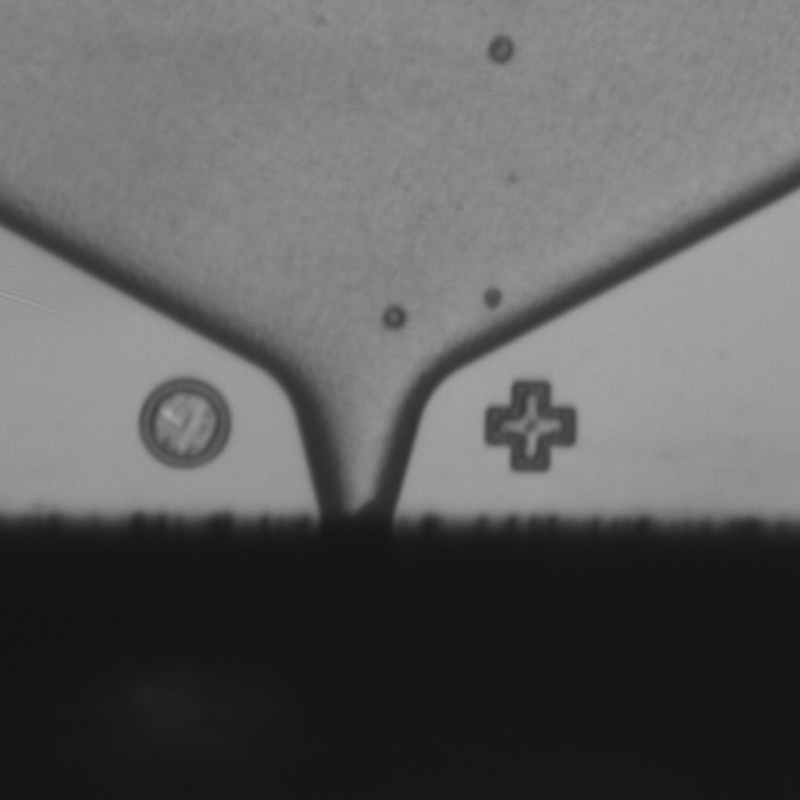

Supplement: Supplementary file 6 — Supplementary Data 3 [file 42003_2021_1661_MOESM6_ESM.zip › Supplementary Data 3 corrected/K_26_B.jpg]

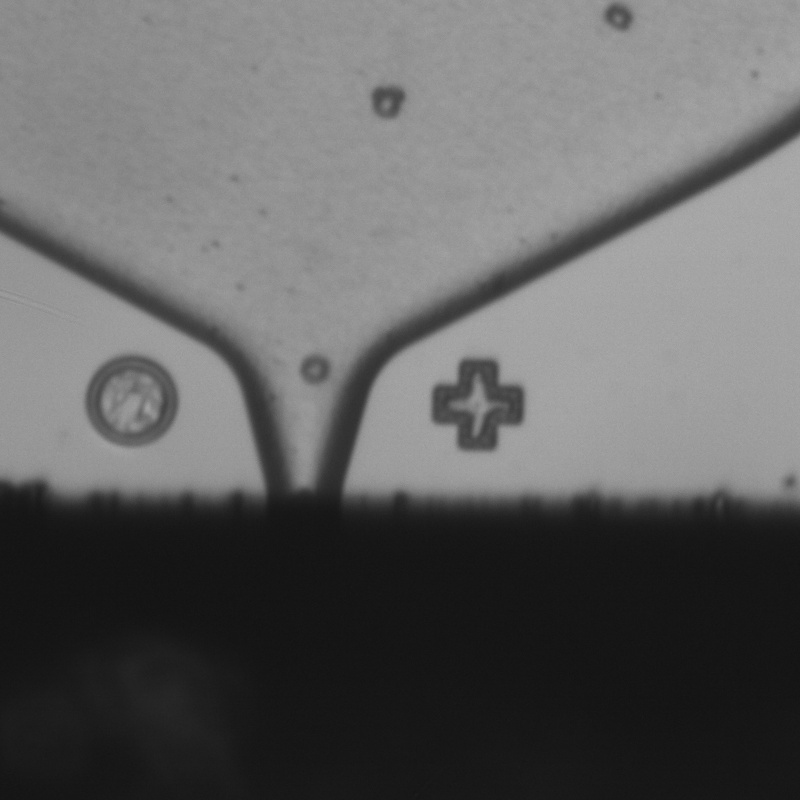

Supplement: Supplementary file 6 — Supplementary Data 3 [file 42003_2021_1661_MOESM6_ESM.zip › Supplementary Data 3 corrected/O_11_C.jpg]

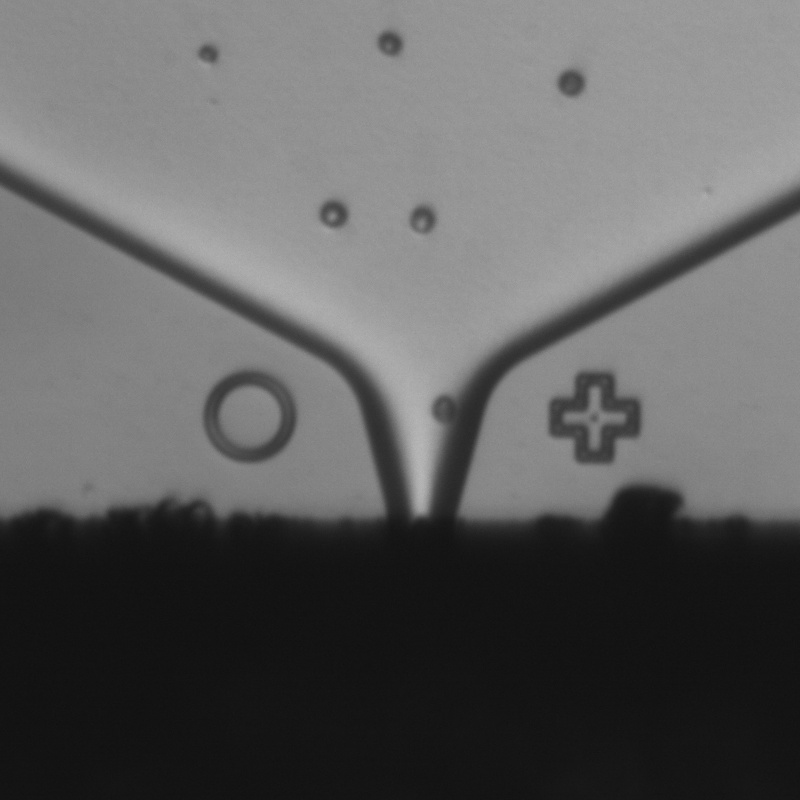

Supplement: Supplementary file 6 — Supplementary Data 3 [file 42003_2021_1661_MOESM6_ESM.zip › Supplementary Data 3 corrected/K_02_C.jpg]

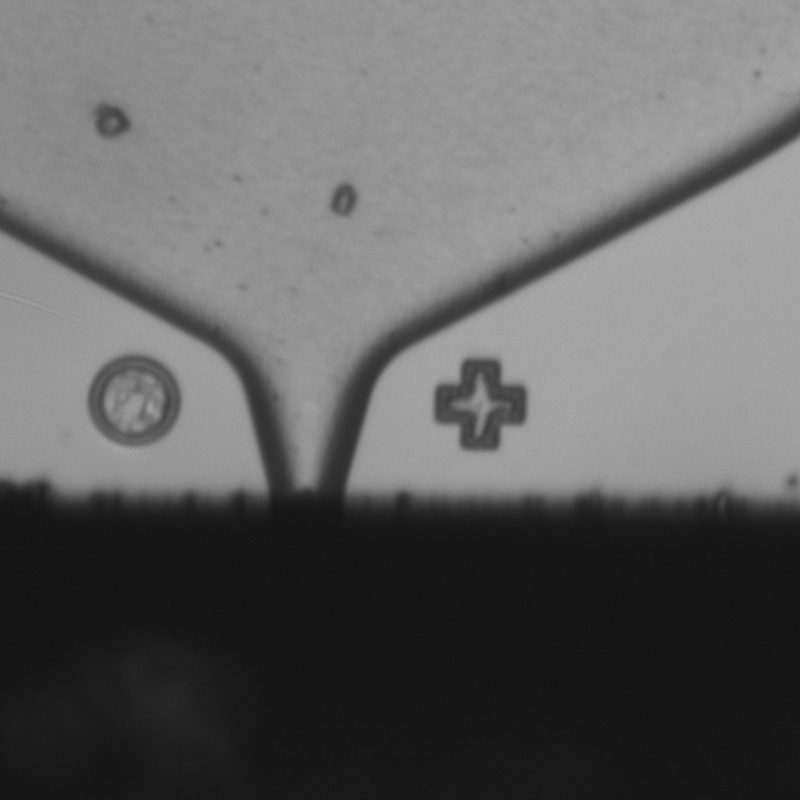

Supplement: Supplementary file 6 — Supplementary Data 3 [file 42003_2021_1661_MOESM6_ESM.zip › Supplementary Data 3 corrected/O_13_A.jpg]

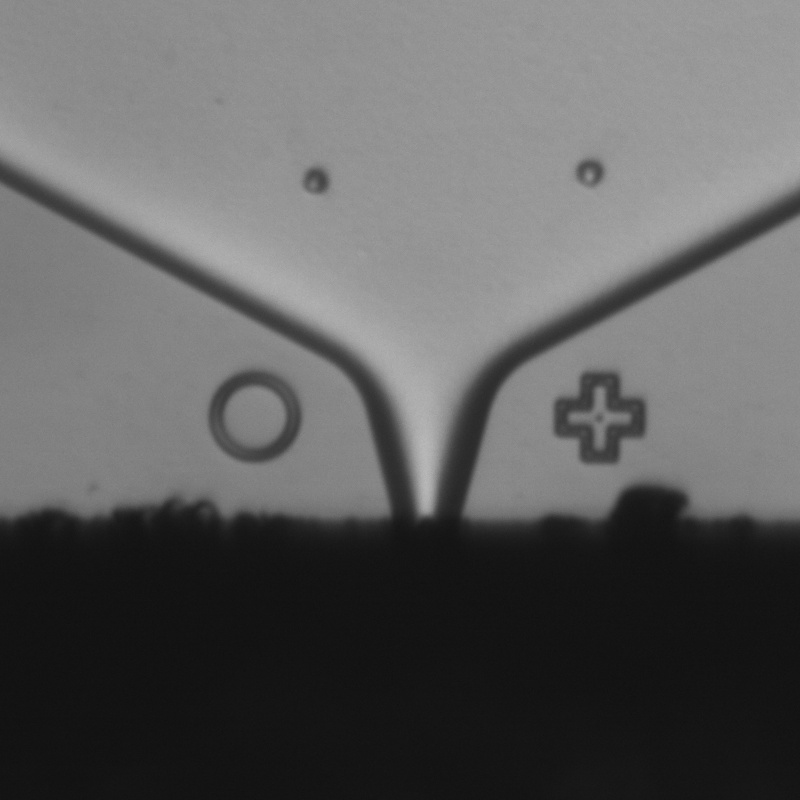

Supplement: Supplementary file 6 — Supplementary Data 3 [file 42003_2021_1661_MOESM6_ESM.zip › Supplementary Data 3 corrected/K_04_E.jpg]

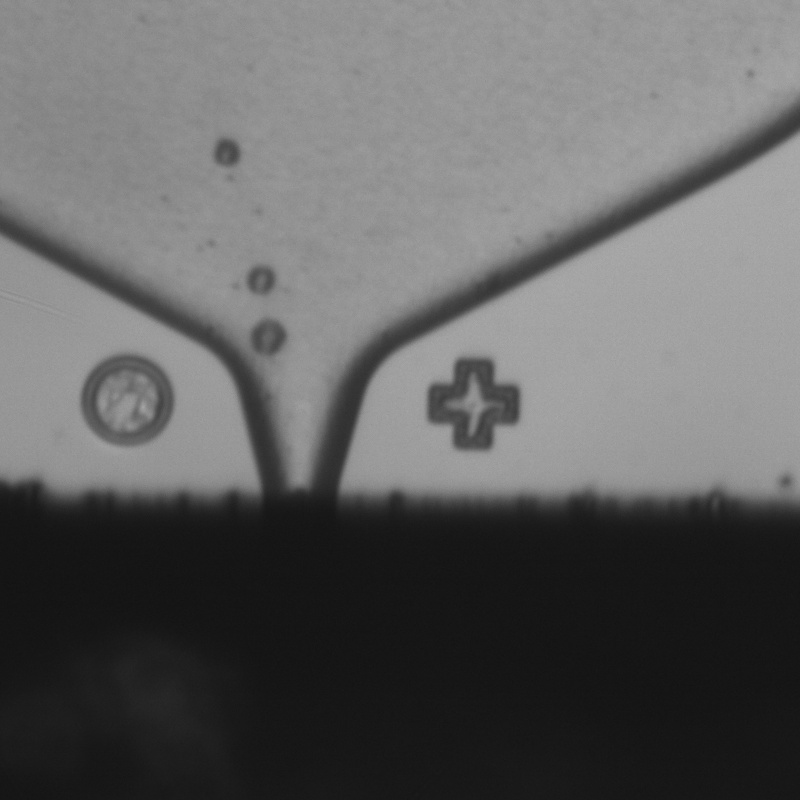

Supplement: Supplementary file 6 — Supplementary Data 3 [file 42003_2021_1661_MOESM6_ESM.zip › Supplementary Data 3 corrected/O_08_B.jpg]

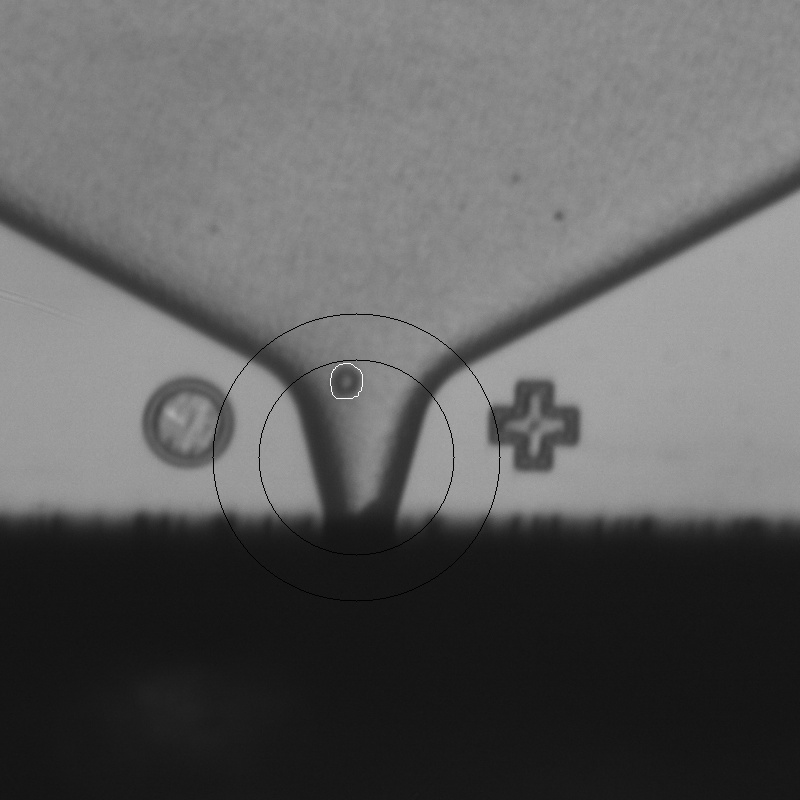

Supplement: Supplementary file 6 — Supplementary Data 3 [file 42003_2021_1661_MOESM6_ESM.zip › Supplementary Data 3 corrected/K_20_D.jpg]

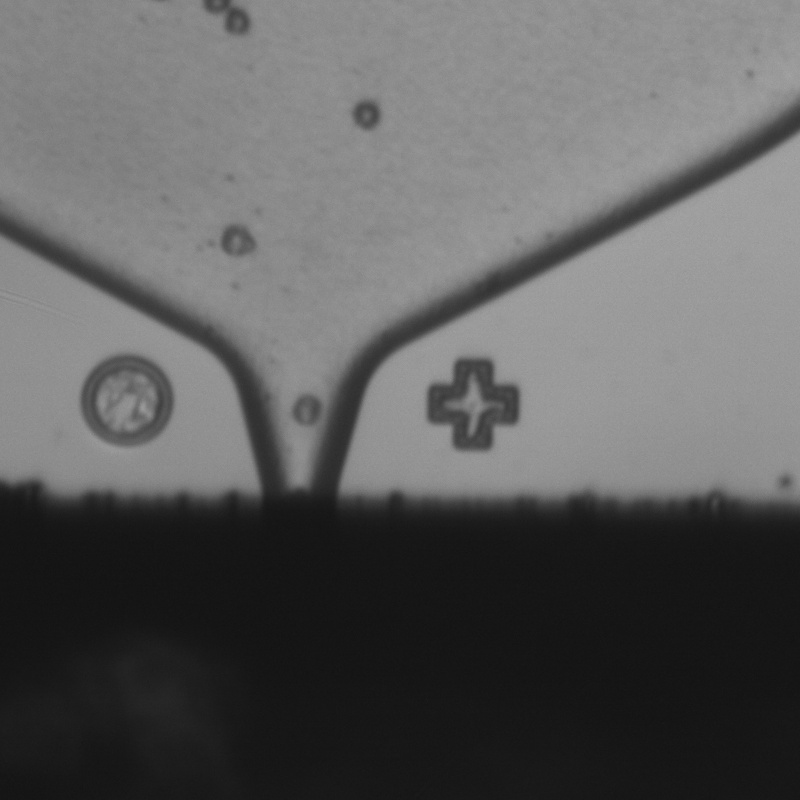

Supplement: Supplementary file 6 — Supplementary Data 3 [file 42003_2021_1661_MOESM6_ESM.zip › Supplementary Data 3 corrected/O_17_E.jpg]

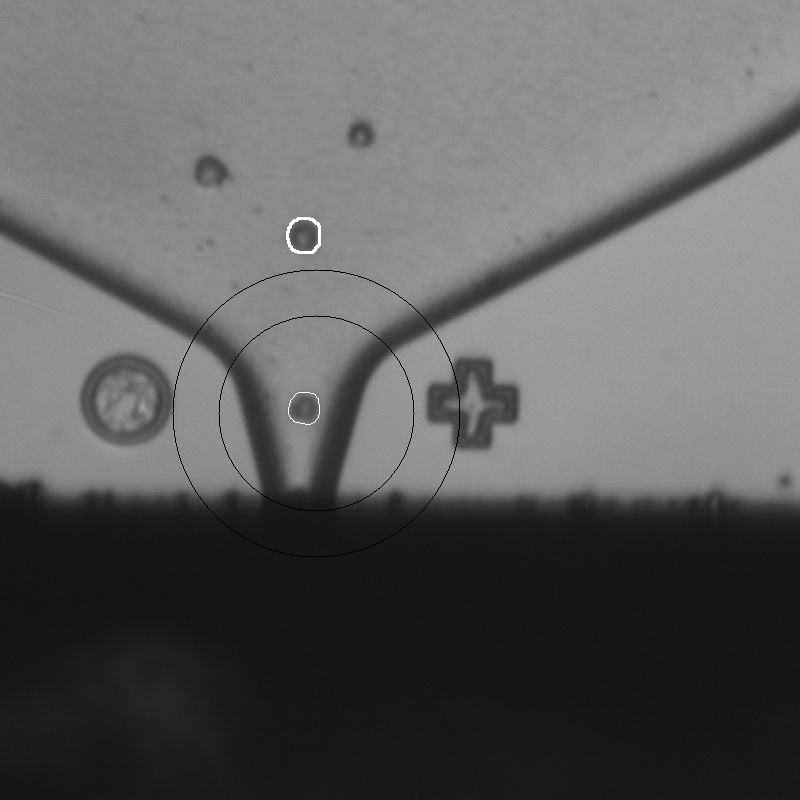

Supplement: Supplementary file 6 — Supplementary Data 3 [file 42003_2021_1661_MOESM6_ESM.zip › Supplementary Data 3 corrected/O_17_D.jpg]

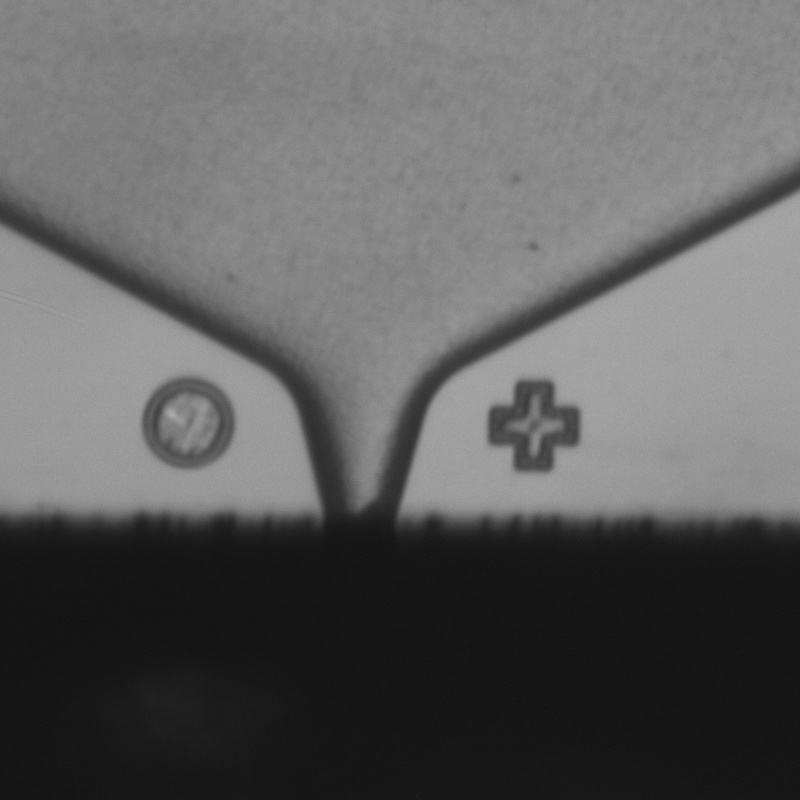

Supplement: Supplementary file 6 — Supplementary Data 3 [file 42003_2021_1661_MOESM6_ESM.zip › Supplementary Data 3 corrected/K_20_E.jpg]

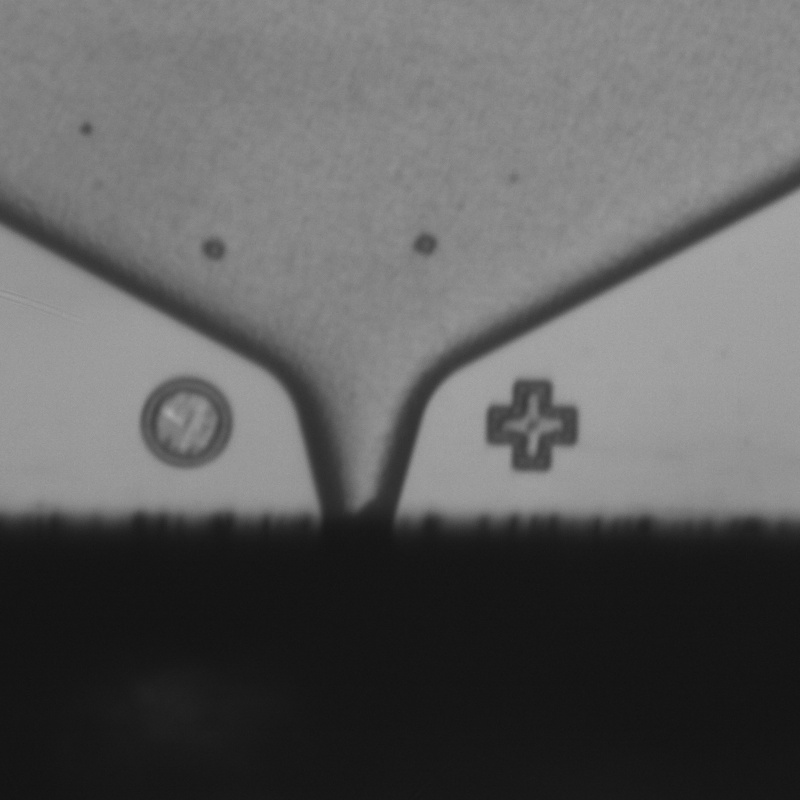

Supplement: Supplementary file 6 — Supplementary Data 3 [file 42003_2021_1661_MOESM6_ESM.zip › Supplementary Data 3 corrected/K_19_A.jpg]

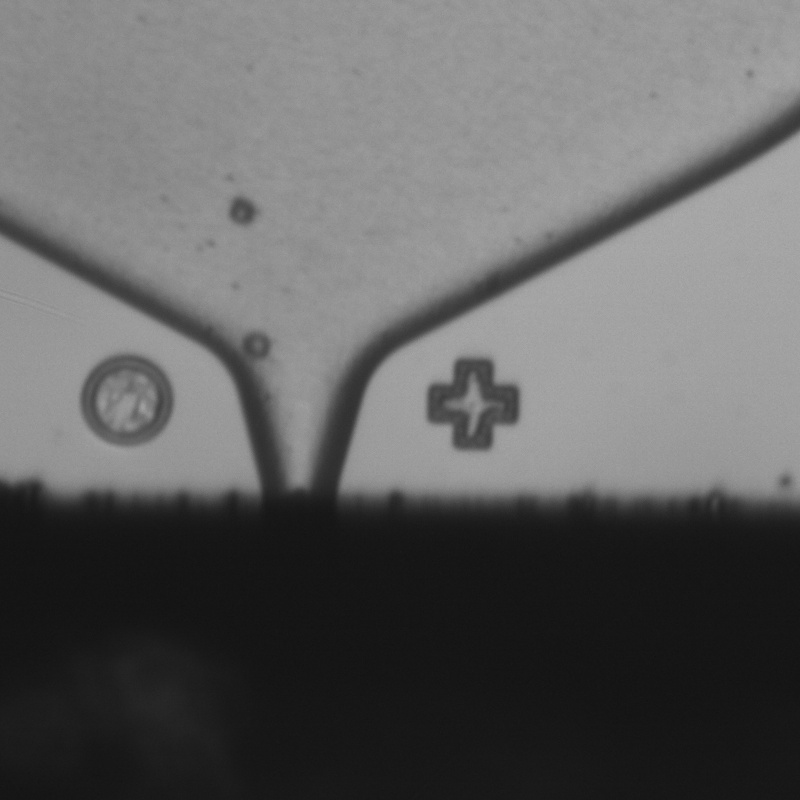

Supplement: Supplementary file 6 — Supplementary Data 3 [file 42003_2021_1661_MOESM6_ESM.zip › Supplementary Data 3 corrected/O_08_C.jpg]

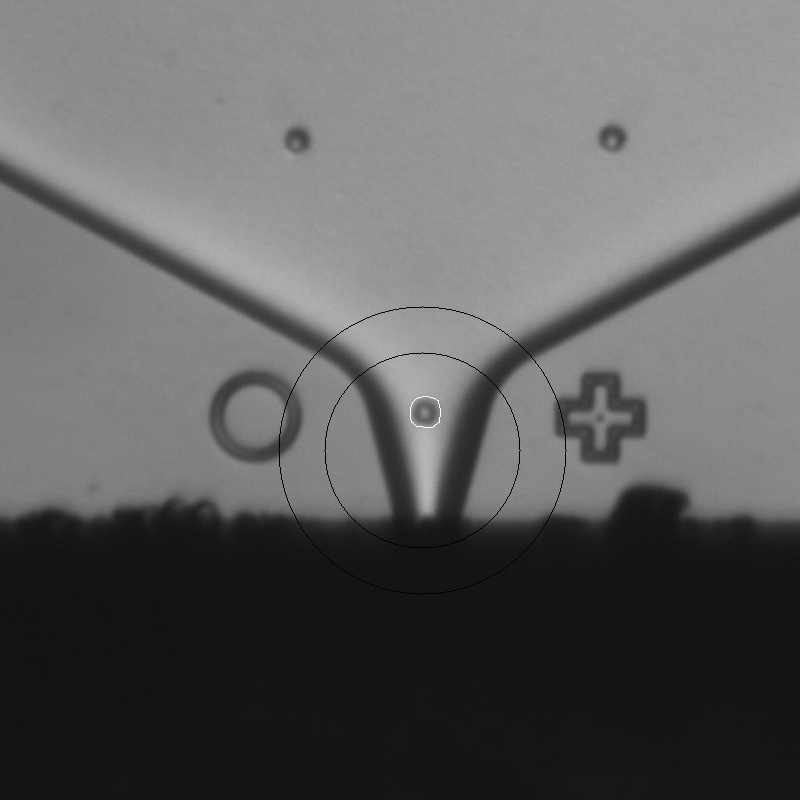

Supplement: Supplementary file 6 — Supplementary Data 3 [file 42003_2021_1661_MOESM6_ESM.zip › Supplementary Data 3 corrected/K_04_D.jpg]

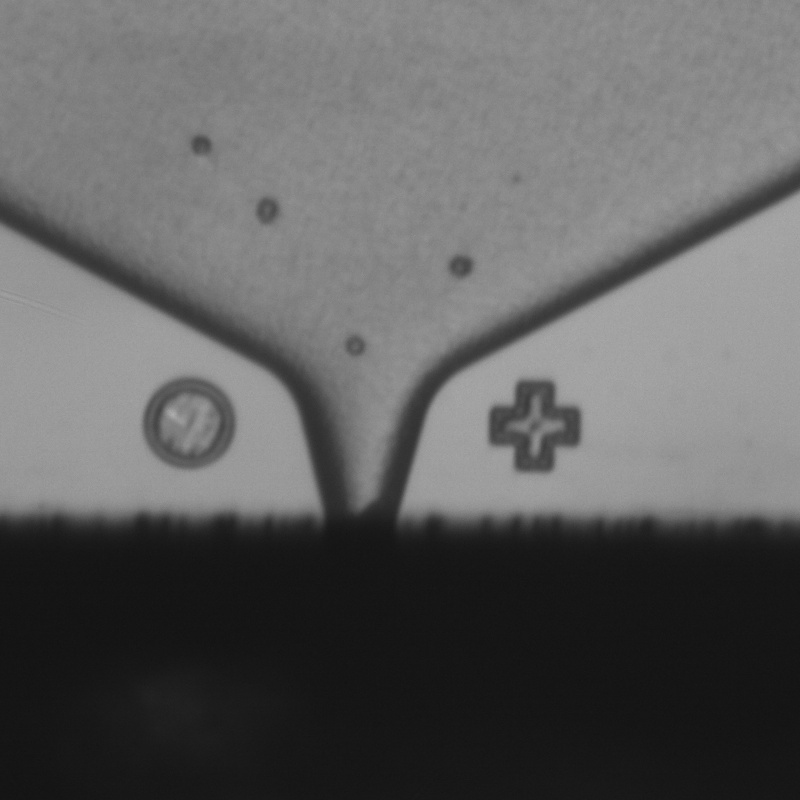

Supplement: Supplementary file 6 — Supplementary Data 3 [file 42003_2021_1661_MOESM6_ESM.zip › Supplementary Data 3 corrected/K_24_A.jpg]

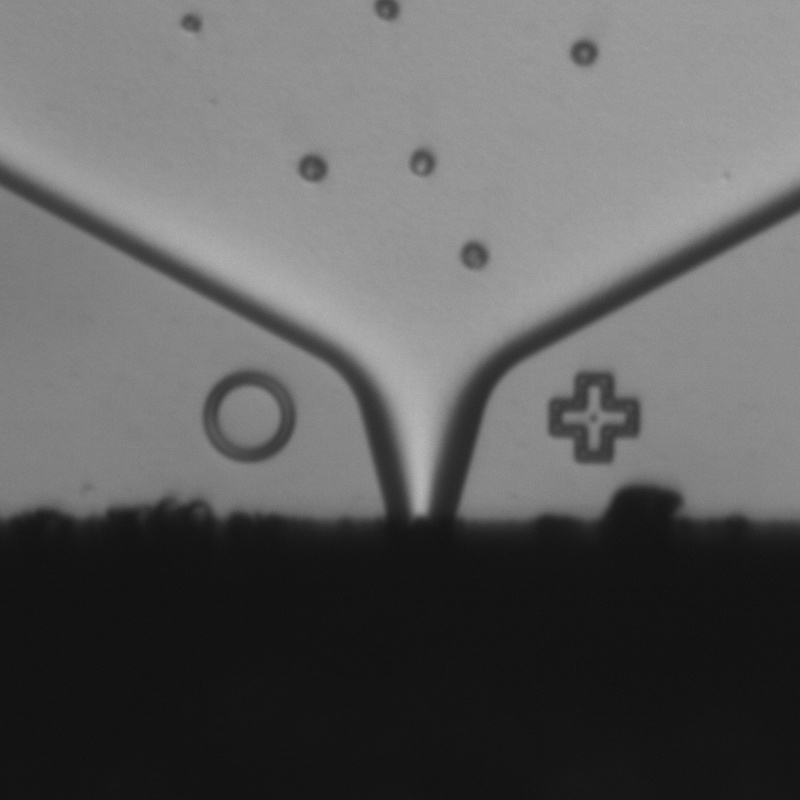

Supplement: Supplementary file 6 — Supplementary Data 3 [file 42003_2021_1661_MOESM6_ESM.zip › Supplementary Data 3 corrected/K_02_B.jpg]

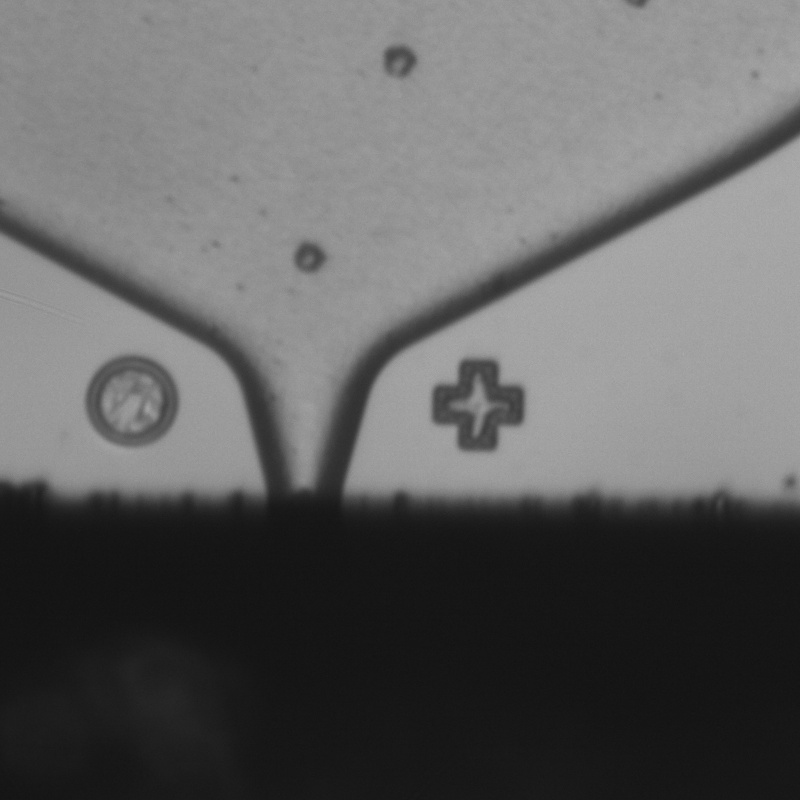

Supplement: Supplementary file 6 — Supplementary Data 3 [file 42003_2021_1661_MOESM6_ESM.zip › Supplementary Data 3 corrected/O_11_B.jpg]

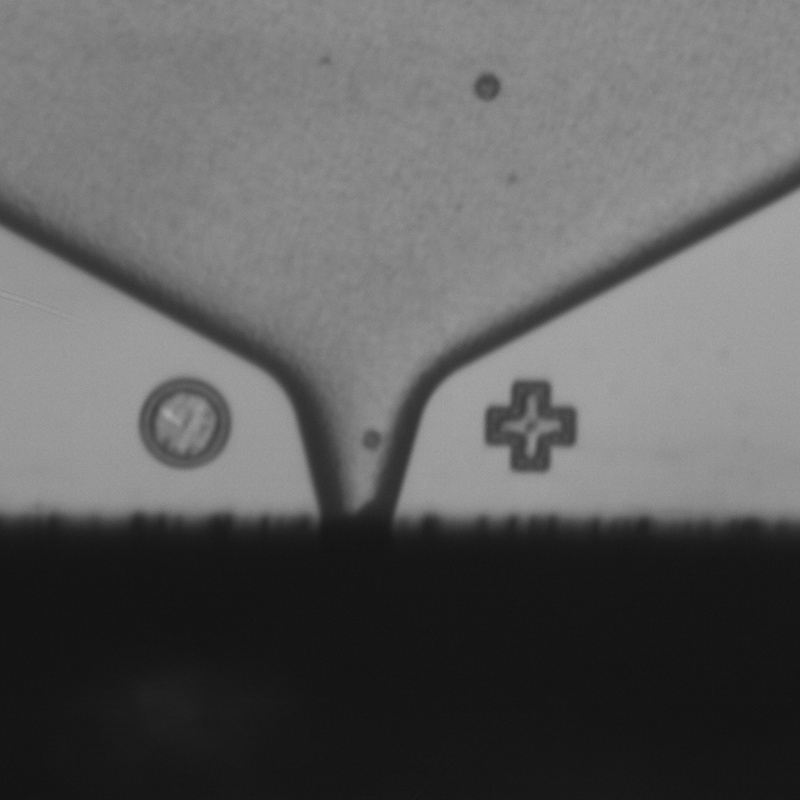

Supplement: Supplementary file 6 — Supplementary Data 3 [file 42003_2021_1661_MOESM6_ESM.zip › Supplementary Data 3 corrected/K_26_C.jpg]

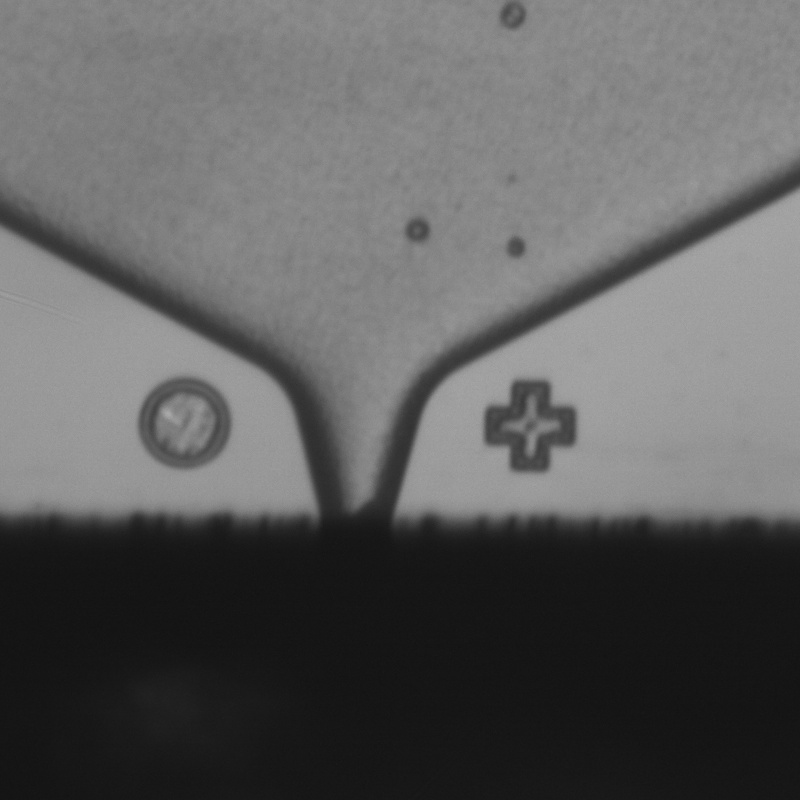

Supplement: Supplementary file 6 — Supplementary Data 3 [file 42003_2021_1661_MOESM6_ESM.zip › Supplementary Data 3 corrected/K_26_A.jpg]

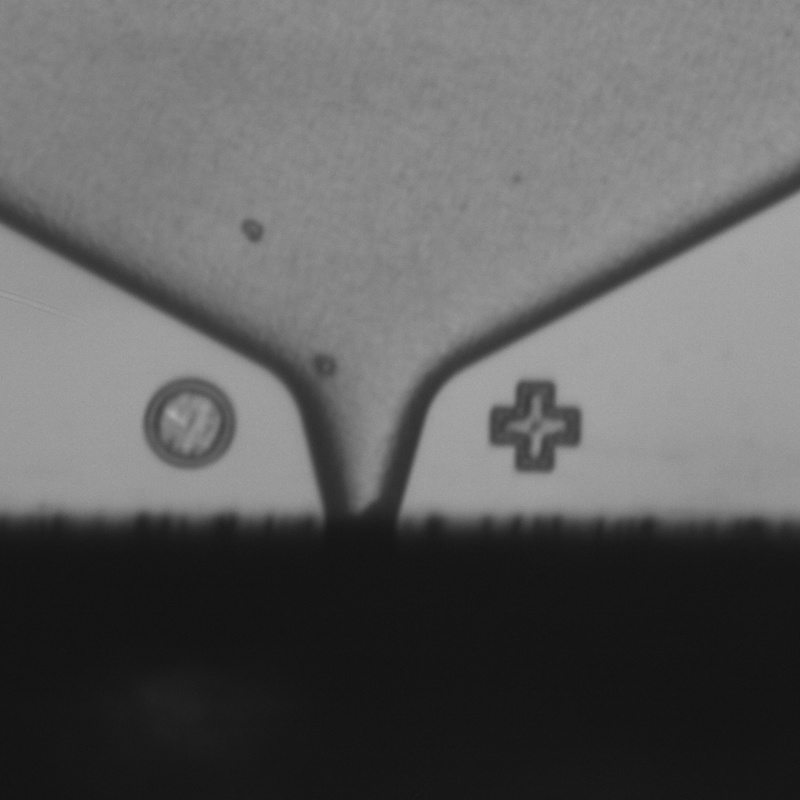

Supplement: Supplementary file 6 — Supplementary Data 3 [file 42003_2021_1661_MOESM6_ESM.zip › Supplementary Data 3 corrected/K_24_C.jpg]

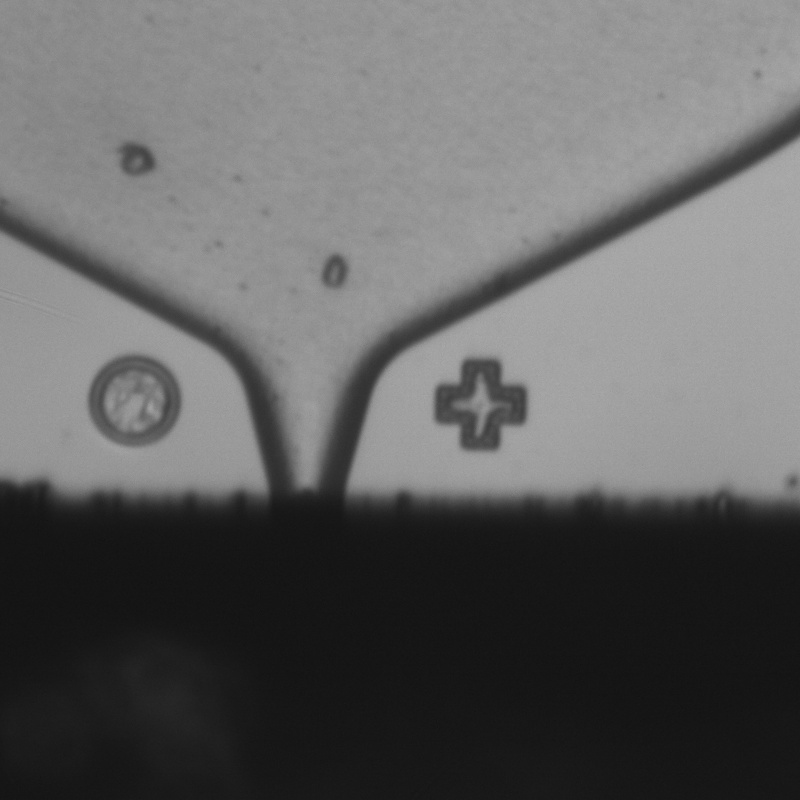

Supplement: Supplementary file 6 — Supplementary Data 3 [file 42003_2021_1661_MOESM6_ESM.zip › Supplementary Data 3 corrected/O_13_B.jpg]

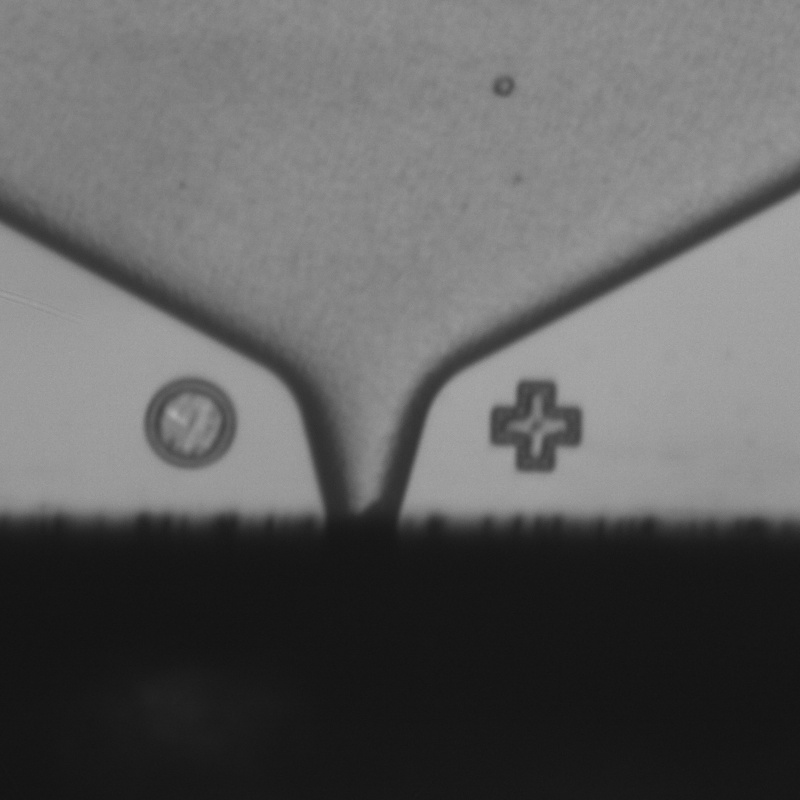

Supplement: Supplementary file 6 — Supplementary Data 3 [file 42003_2021_1661_MOESM6_ESM.zip › Supplementary Data 3 corrected/K_22_E.jpg]

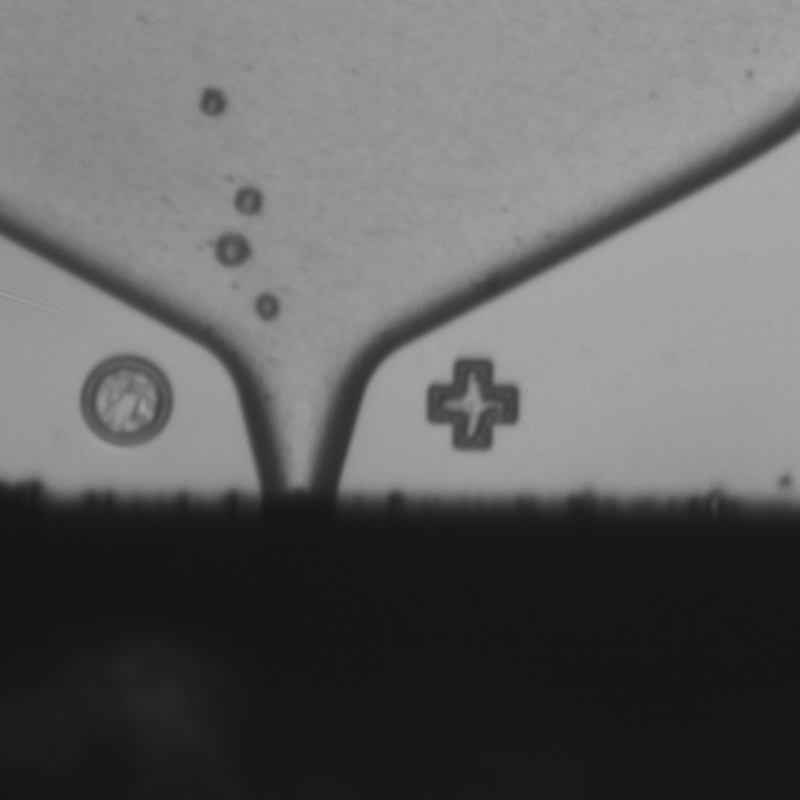

Supplement: Supplementary file 6 — Supplementary Data 3 [file 42003_2021_1661_MOESM6_ESM.zip › Supplementary Data 3 corrected/O_08_A.jpg]

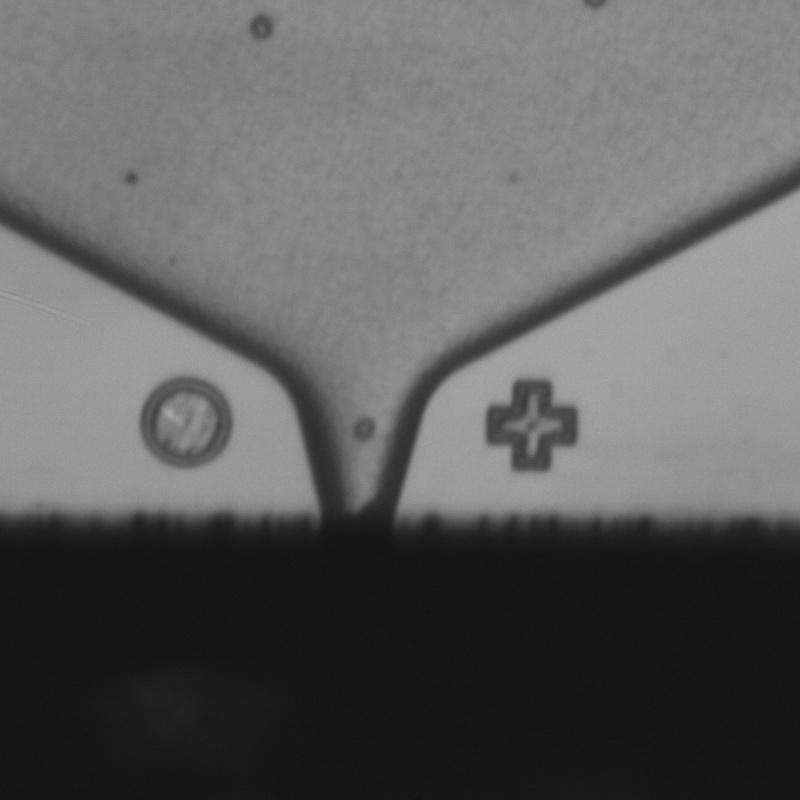

Supplement: Supplementary file 6 — Supplementary Data 3 [file 42003_2021_1661_MOESM6_ESM.zip › Supplementary Data 3 corrected/K_19_C.jpg]

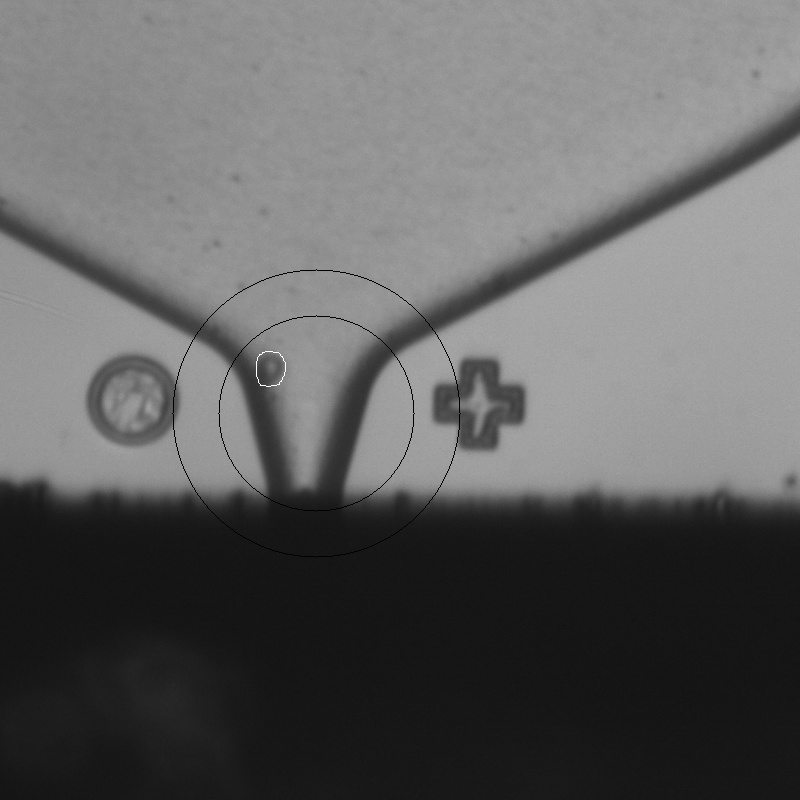

Supplement: Supplementary file 6 — Supplementary Data 3 [file 42003_2021_1661_MOESM6_ESM.zip › Supplementary Data 3 corrected/O_15_D.jpg]

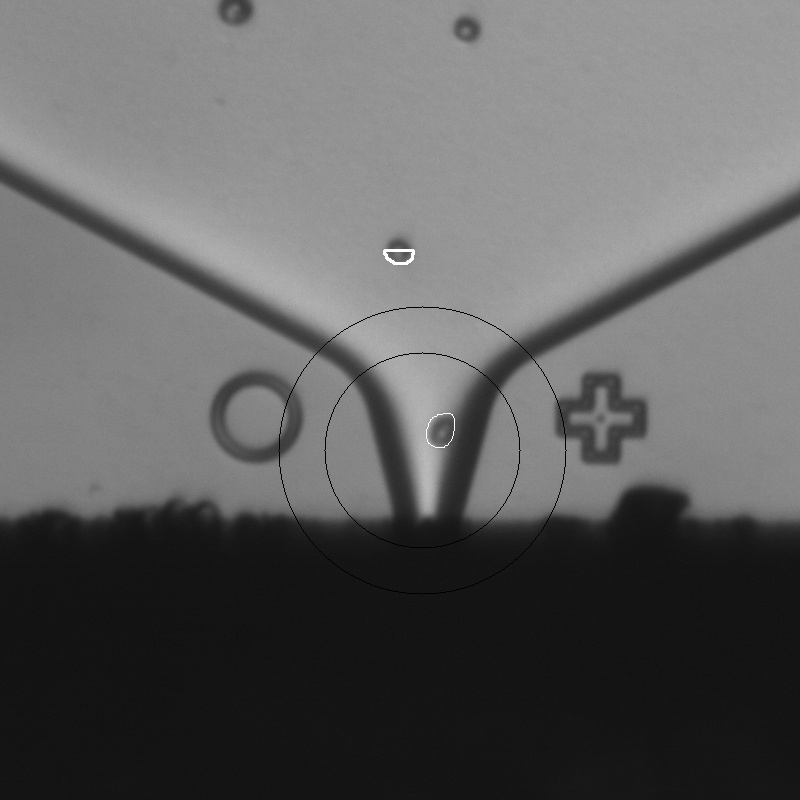

Supplement: Supplementary file 6 — Supplementary Data 3 [file 42003_2021_1661_MOESM6_ESM.zip › Supplementary Data 3 corrected/K_06_D.jpg]

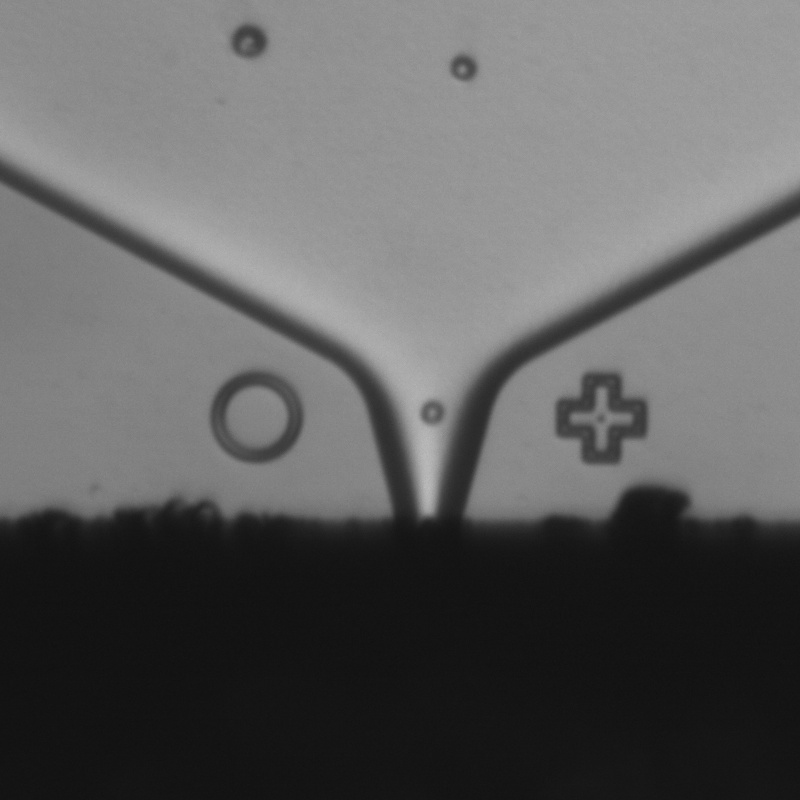

Supplement: Supplementary file 6 — Supplementary Data 3 [file 42003_2021_1661_MOESM6_ESM.zip › Supplementary Data 3 corrected/K_06_E.jpg]

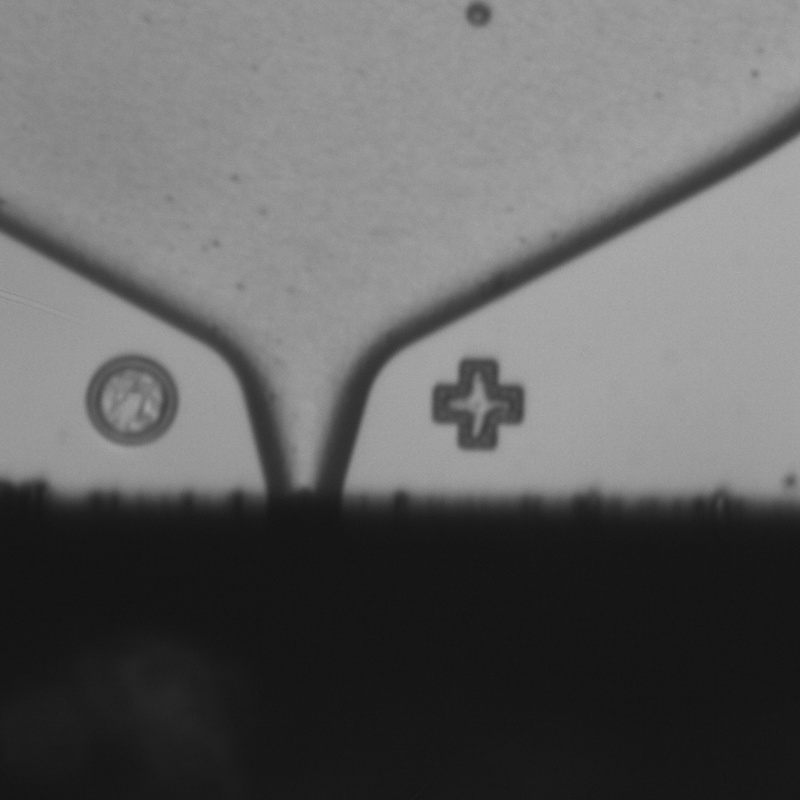

Supplement: Supplementary file 6 — Supplementary Data 3 [file 42003_2021_1661_MOESM6_ESM.zip › Supplementary Data 3 corrected/O_15_E.jpg]

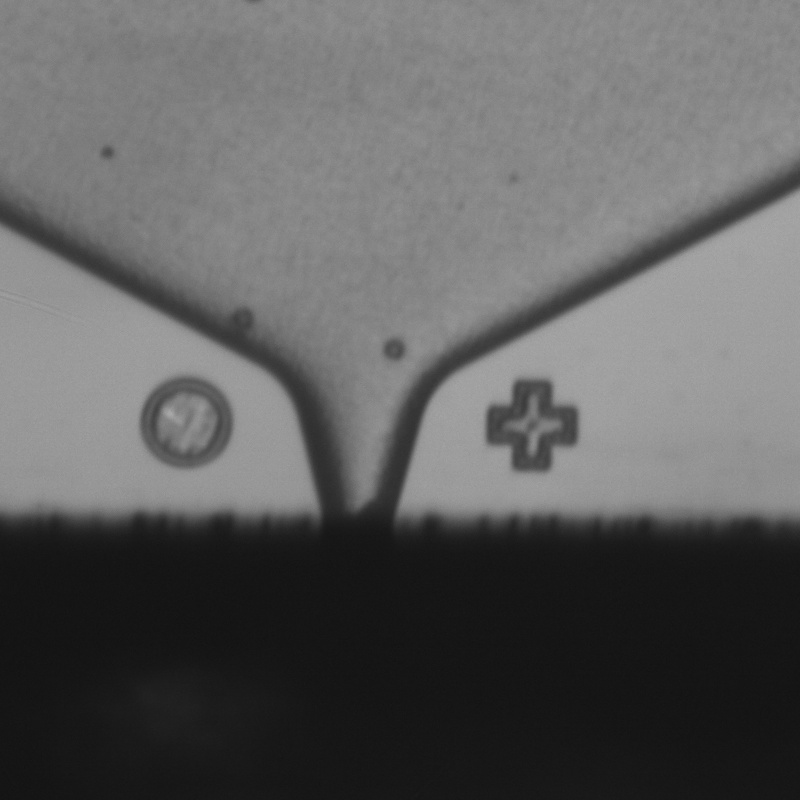

Supplement: Supplementary file 6 — Supplementary Data 3 [file 42003_2021_1661_MOESM6_ESM.zip › Supplementary Data 3 corrected/K_19_B.jpg]

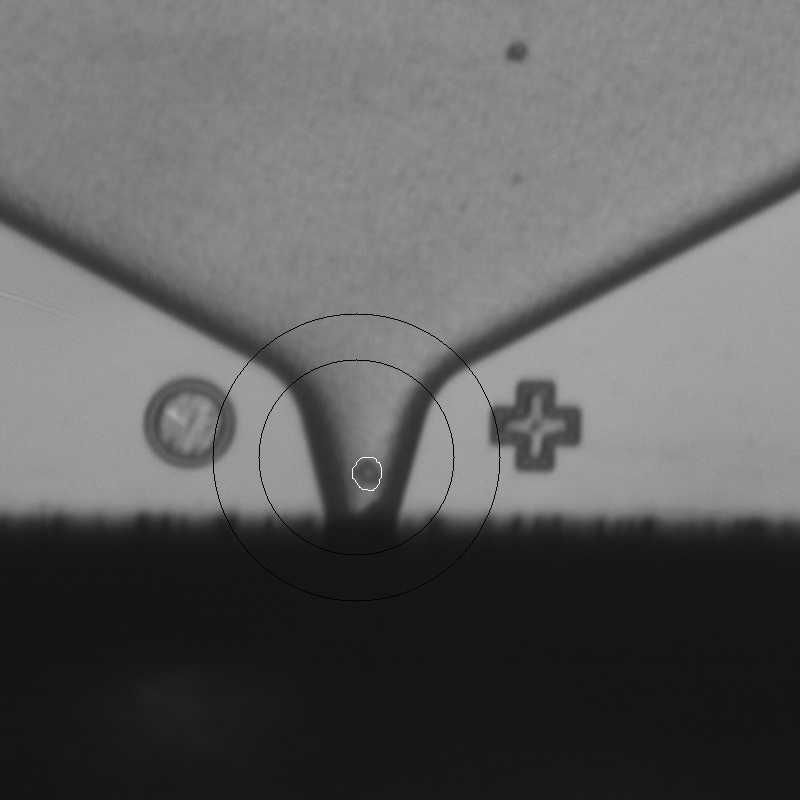

Supplement: Supplementary file 6 — Supplementary Data 3 [file 42003_2021_1661_MOESM6_ESM.zip › Supplementary Data 3 corrected/K_22_D.jpg]

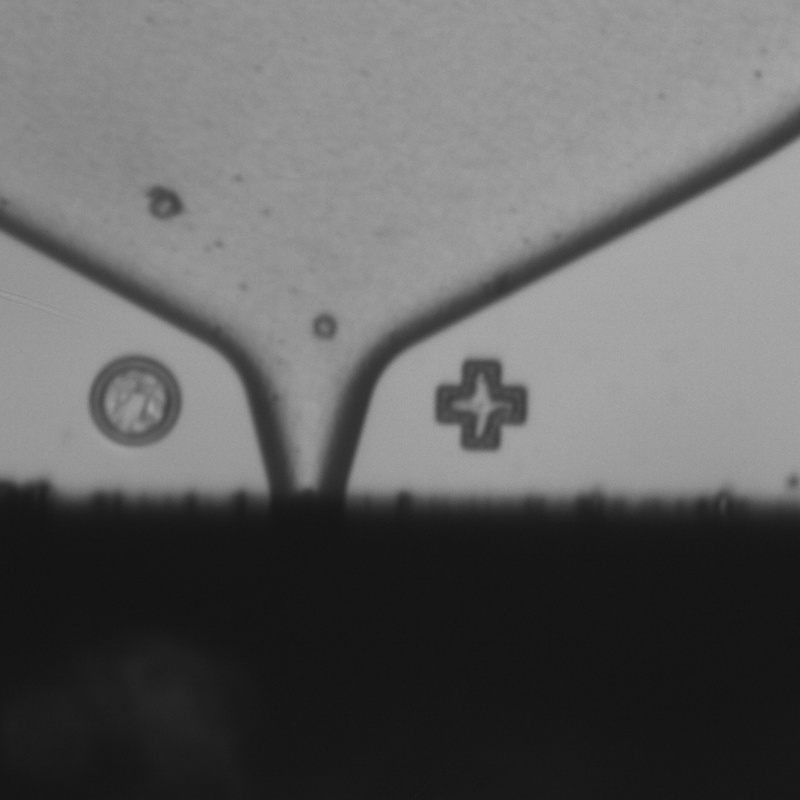

Supplement: Supplementary file 6 — Supplementary Data 3 [file 42003_2021_1661_MOESM6_ESM.zip › Supplementary Data 3 corrected/O_13_C.jpg]

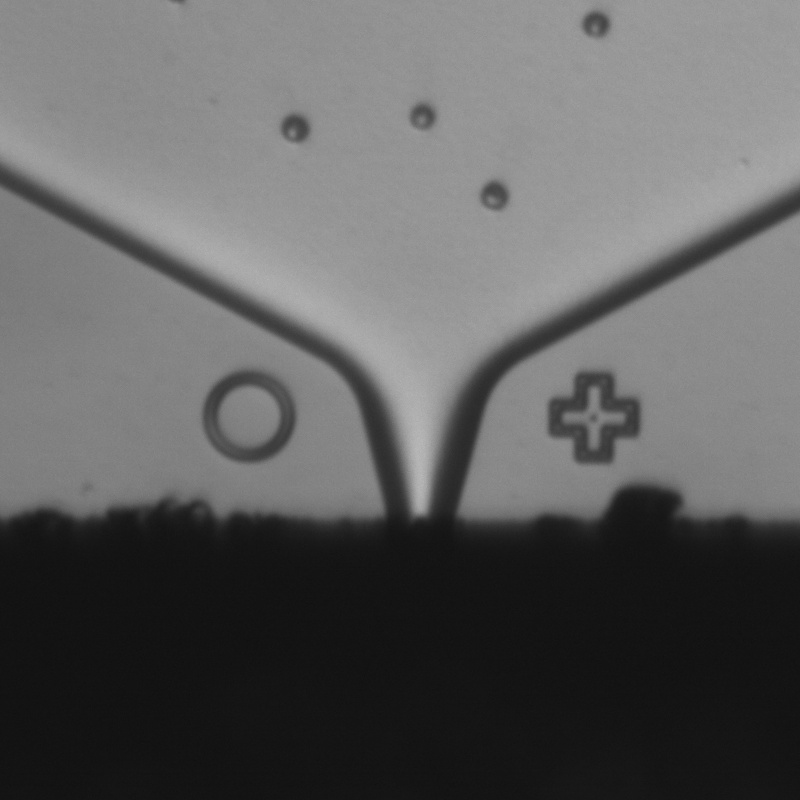

Supplement: Supplementary file 6 — Supplementary Data 3 [file 42003_2021_1661_MOESM6_ESM.zip › Supplementary Data 3 corrected/K_02_A.jpg]

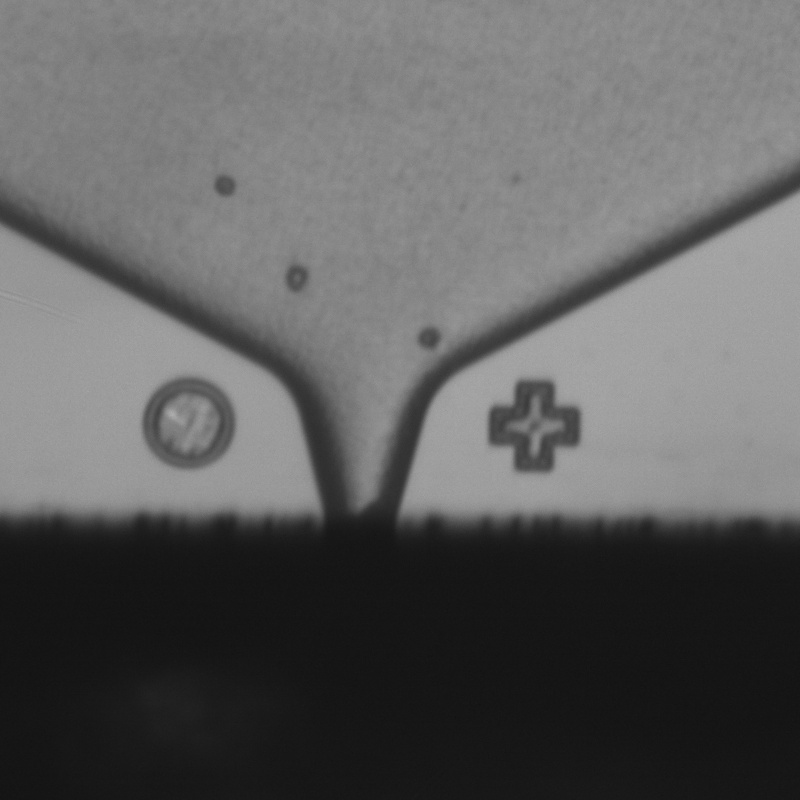

Supplement: Supplementary file 6 — Supplementary Data 3 [file 42003_2021_1661_MOESM6_ESM.zip › Supplementary Data 3 corrected/K_24_B.jpg]

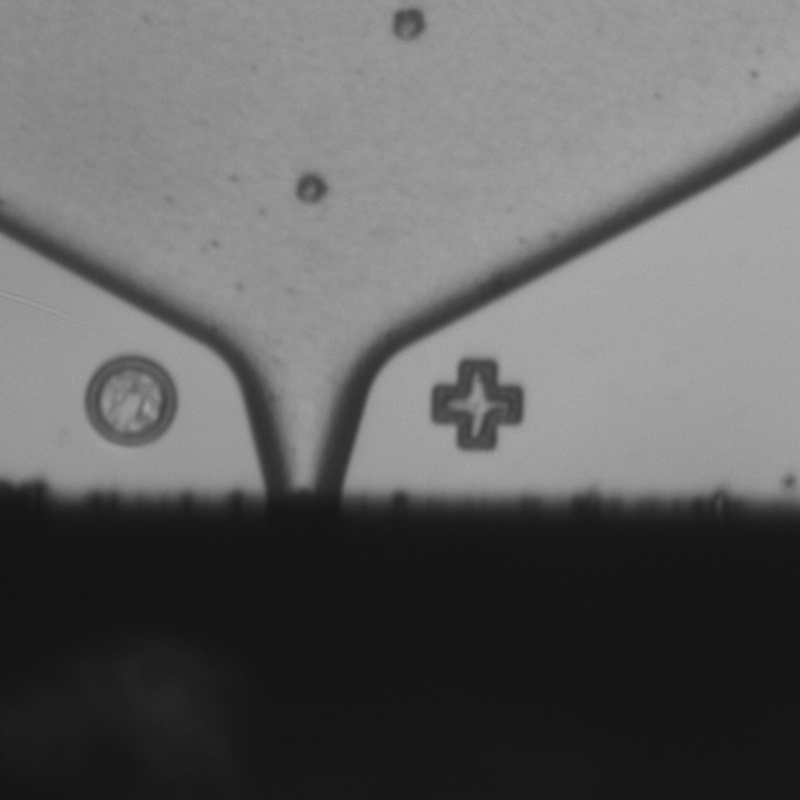

Supplement: Supplementary file 6 — Supplementary Data 3 [file 42003_2021_1661_MOESM6_ESM.zip › Supplementary Data 3 corrected/O_11_A.jpg]

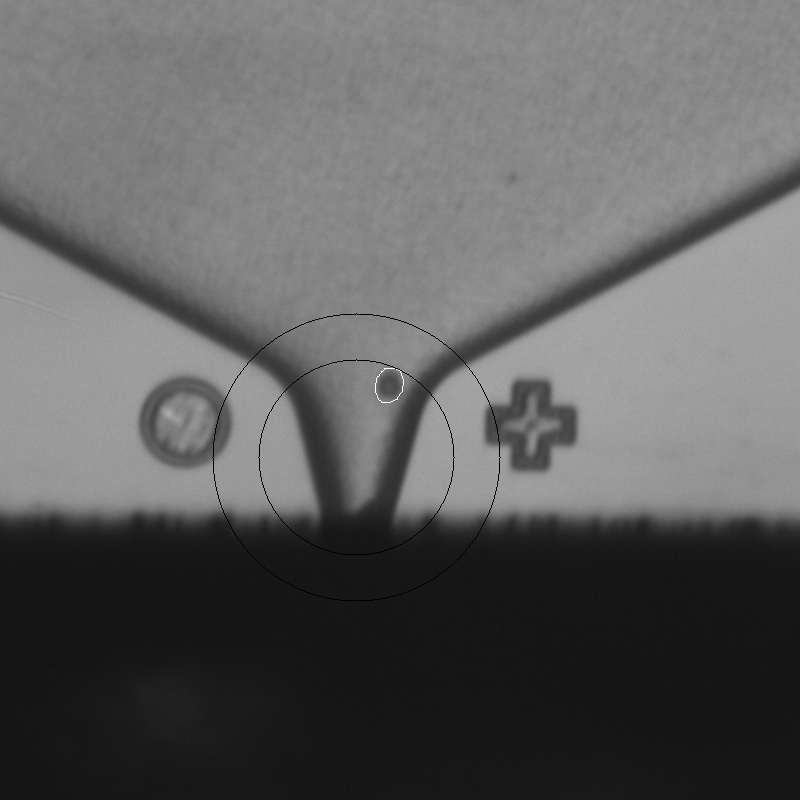

Supplement: Supplementary file 6 — Supplementary Data 3 [file 42003_2021_1661_MOESM6_ESM.zip › Supplementary Data 3 corrected/K_13_D.jpg]

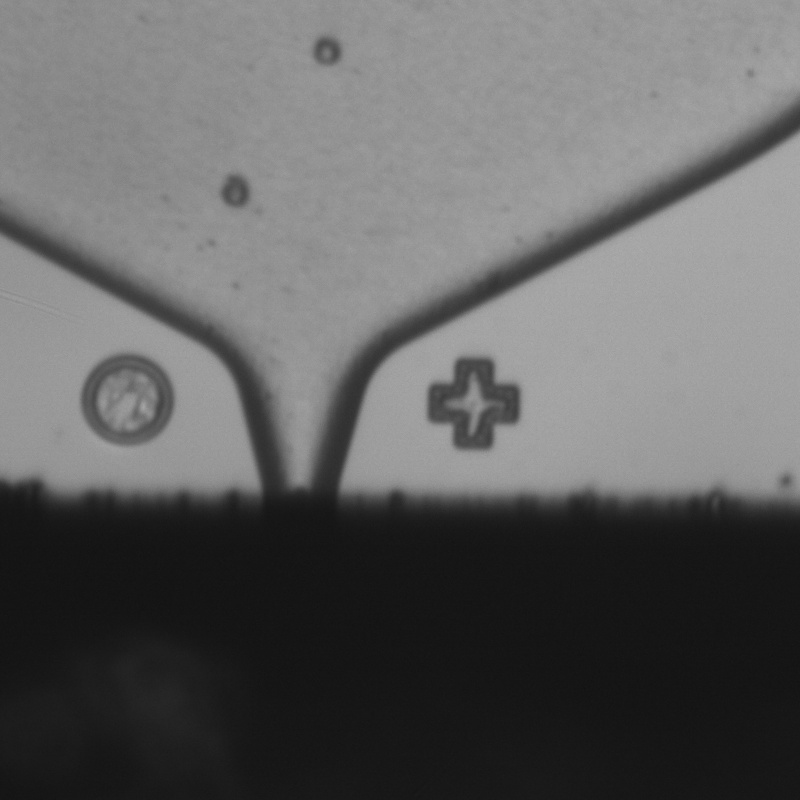

Supplement: Supplementary file 6 — Supplementary Data 3 [file 42003_2021_1661_MOESM6_ESM.zip › Supplementary Data 3 corrected/O_20_A.jpg]

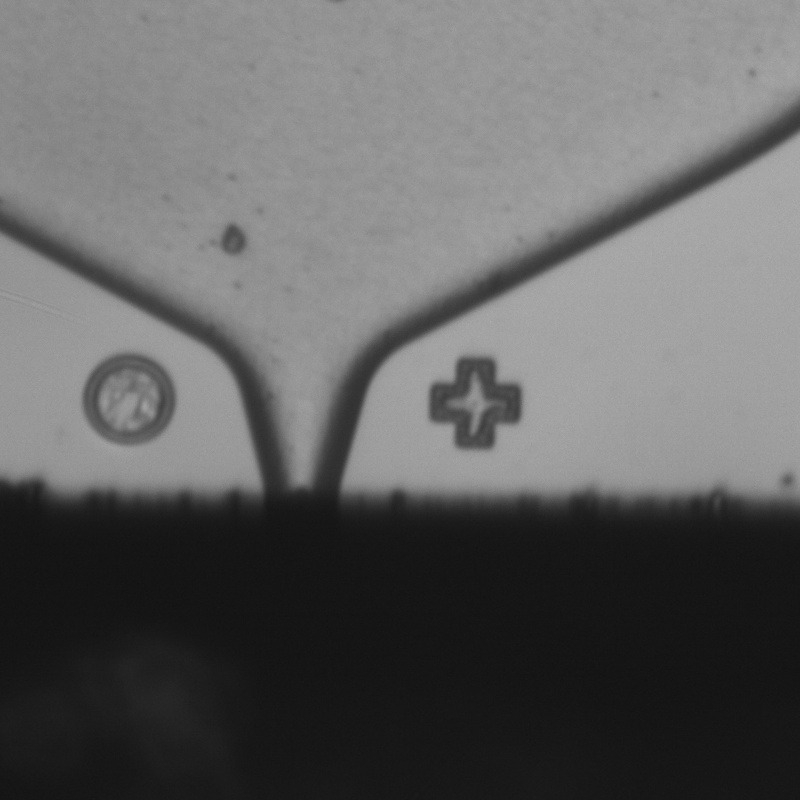

Supplement: Supplementary file 6 — Supplementary Data 3 [file 42003_2021_1661_MOESM6_ESM.zip › Supplementary Data 3 corrected/O_06_B.jpg]

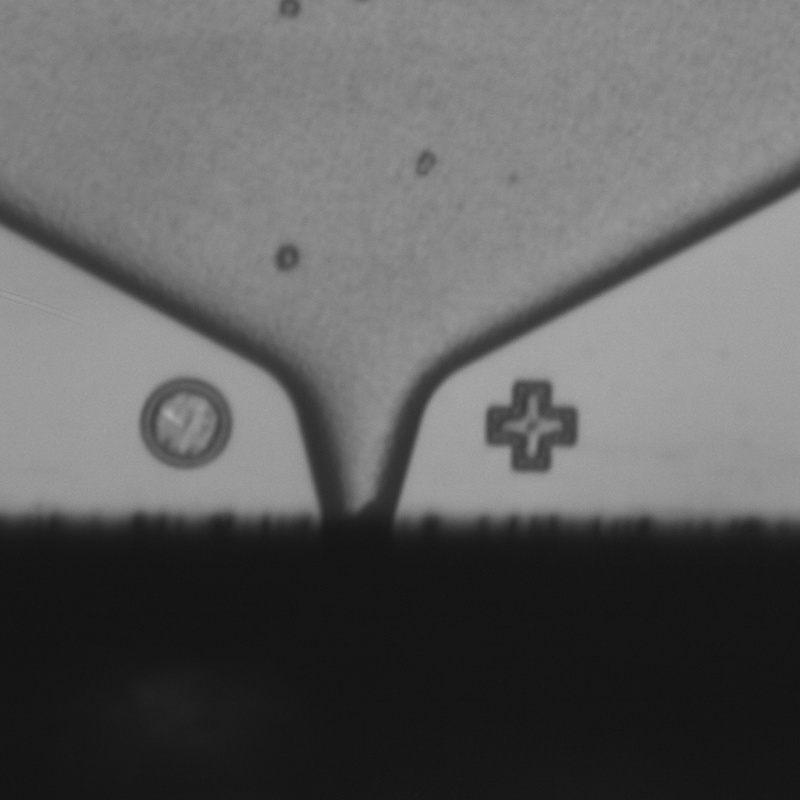

Supplement: Supplementary file 6 — Supplementary Data 3 [file 42003_2021_1661_MOESM6_ESM.zip › Supplementary Data 3 corrected/K_15_B.jpg]

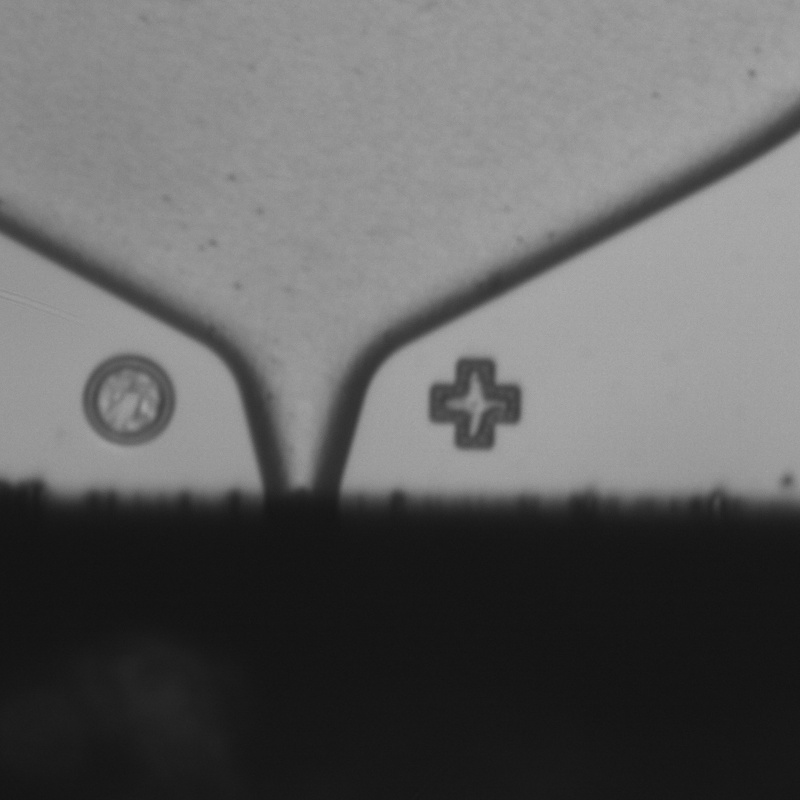

Supplement: Supplementary file 6 — Supplementary Data 3 [file 42003_2021_1661_MOESM6_ESM.zip › Supplementary Data 3 corrected/O_19_E.jpg]

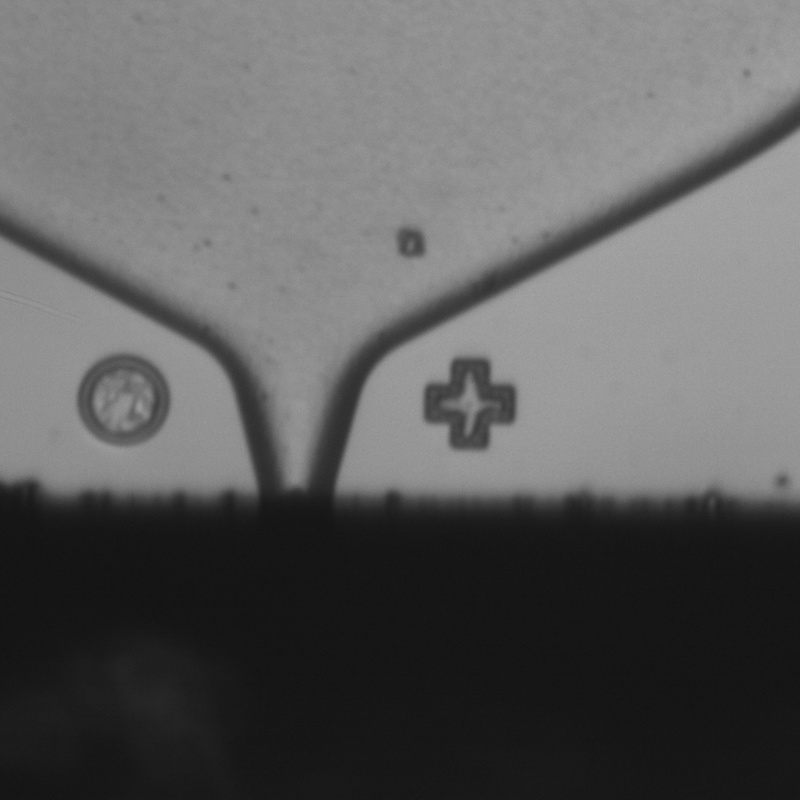

Supplement: Supplementary file 6 — Supplementary Data 3 [file 42003_2021_1661_MOESM6_ESM.zip › Supplementary Data 3 corrected/O_04_A.jpg]

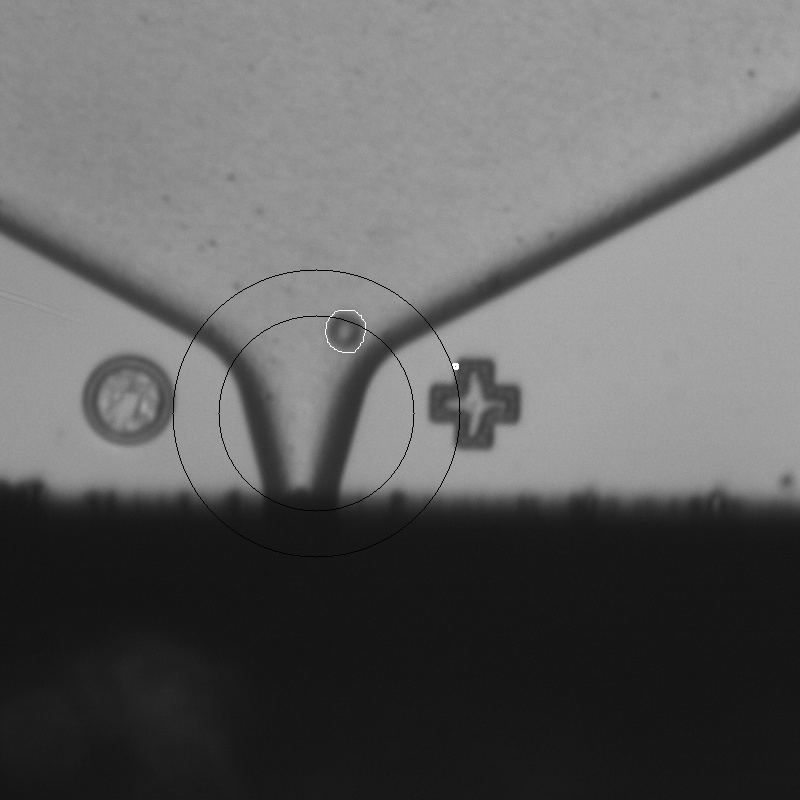

Supplement: Supplementary file 6 — Supplementary Data 3 [file 42003_2021_1661_MOESM6_ESM.zip › Supplementary Data 3 corrected/O_19_D.jpg]

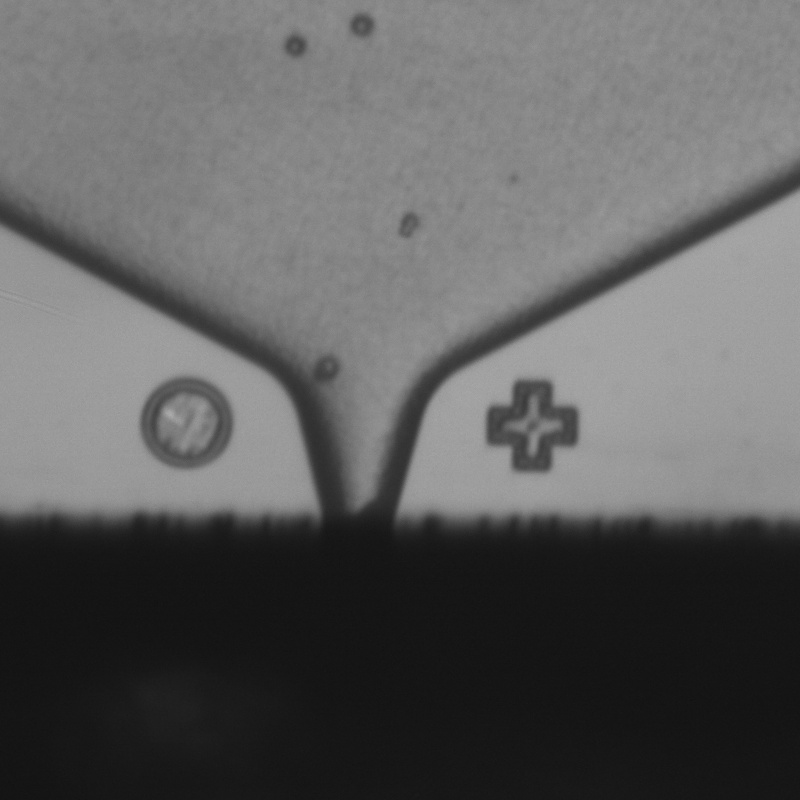

Supplement: Supplementary file 6 — Supplementary Data 3 [file 42003_2021_1661_MOESM6_ESM.zip › Supplementary Data 3 corrected/K_15_C.jpg]

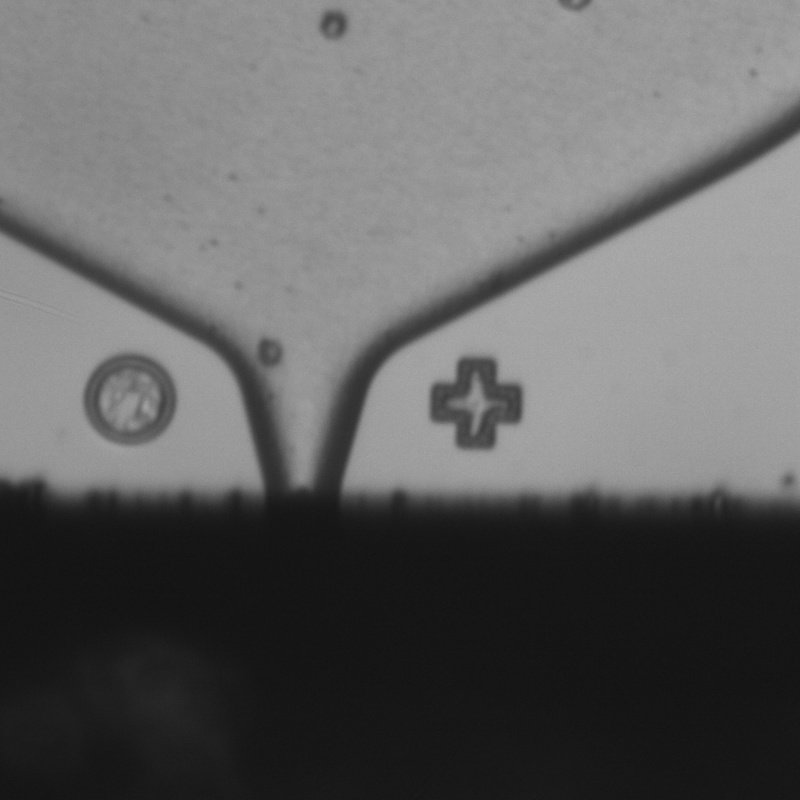

Supplement: Supplementary file 6 — Supplementary Data 3 [file 42003_2021_1661_MOESM6_ESM.zip › Supplementary Data 3 corrected/O_06_C.jpg]

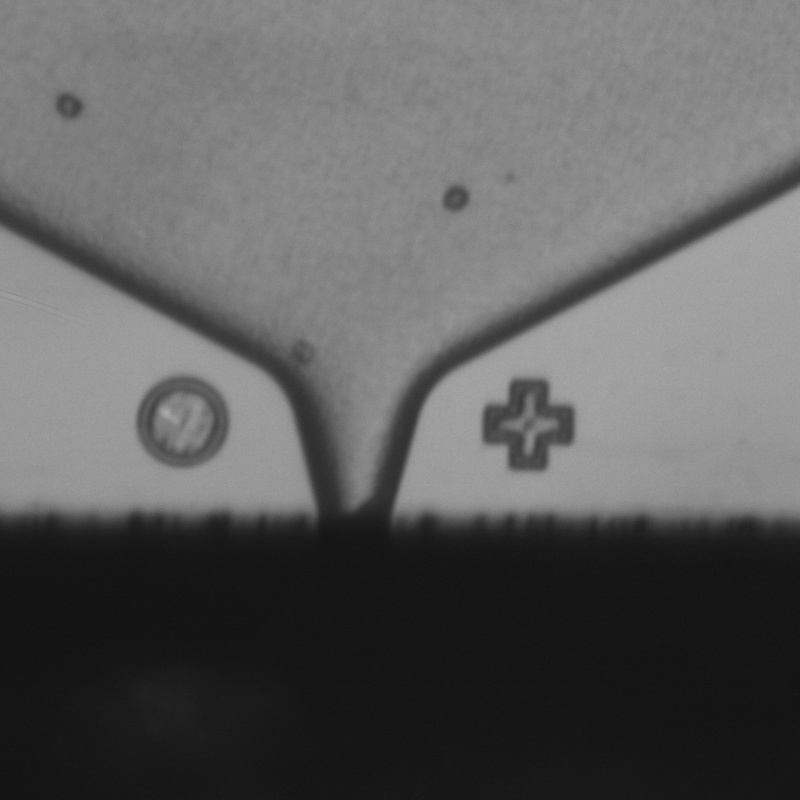

Supplement: Supplementary file 6 — Supplementary Data 3 [file 42003_2021_1661_MOESM6_ESM.zip › Supplementary Data 3 corrected/K_17_A.jpg]

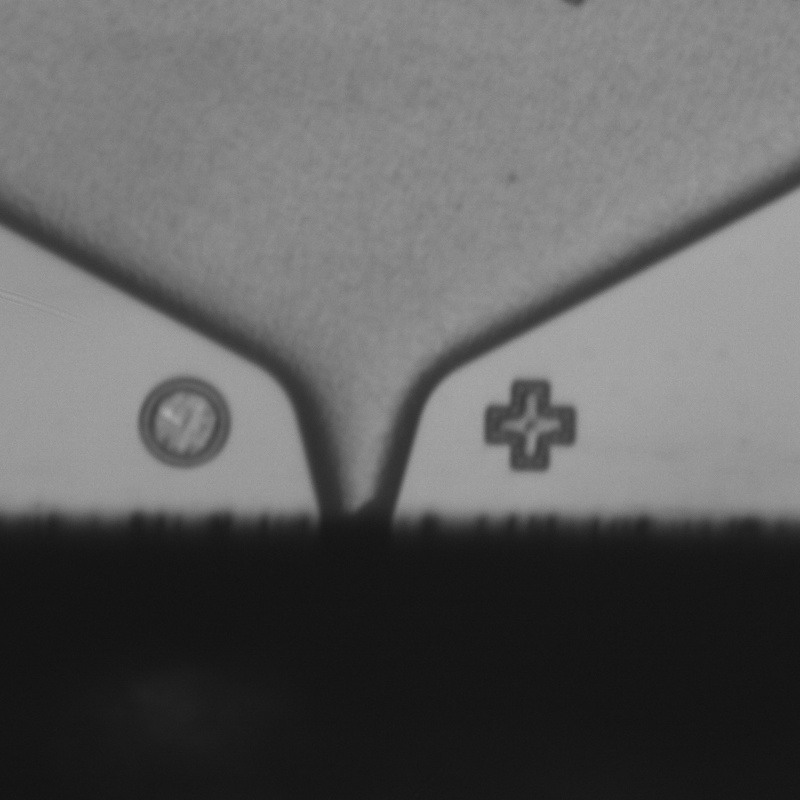

Supplement: Supplementary file 6 — Supplementary Data 3 [file 42003_2021_1661_MOESM6_ESM.zip › Supplementary Data 3 corrected/K_13_E.jpg]

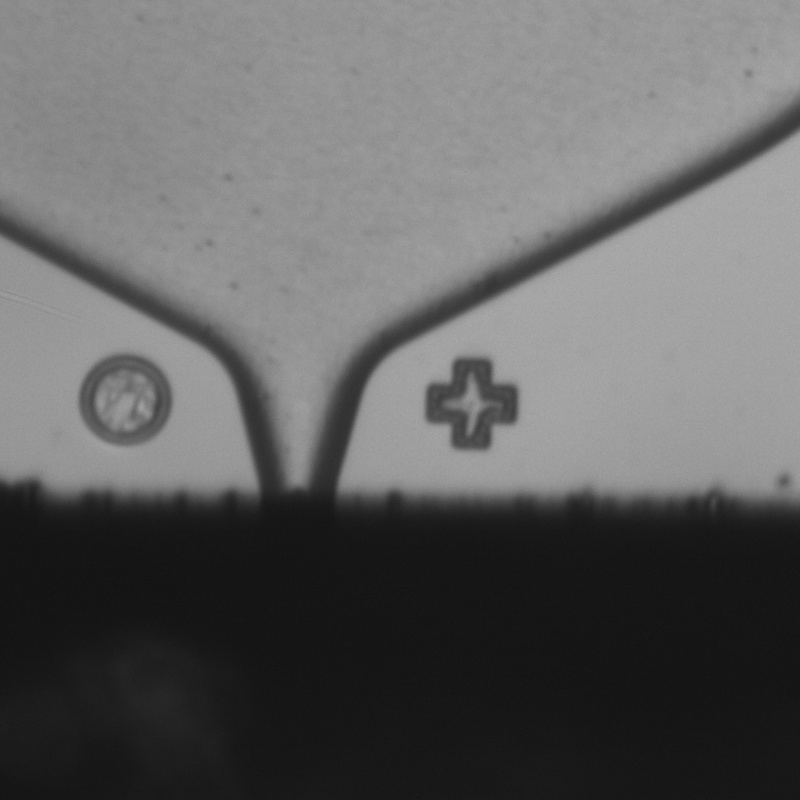

Supplement: Supplementary file 6 — Supplementary Data 3 [file 42003_2021_1661_MOESM6_ESM.zip › Supplementary Data 3 corrected/O_02_E.jpg]

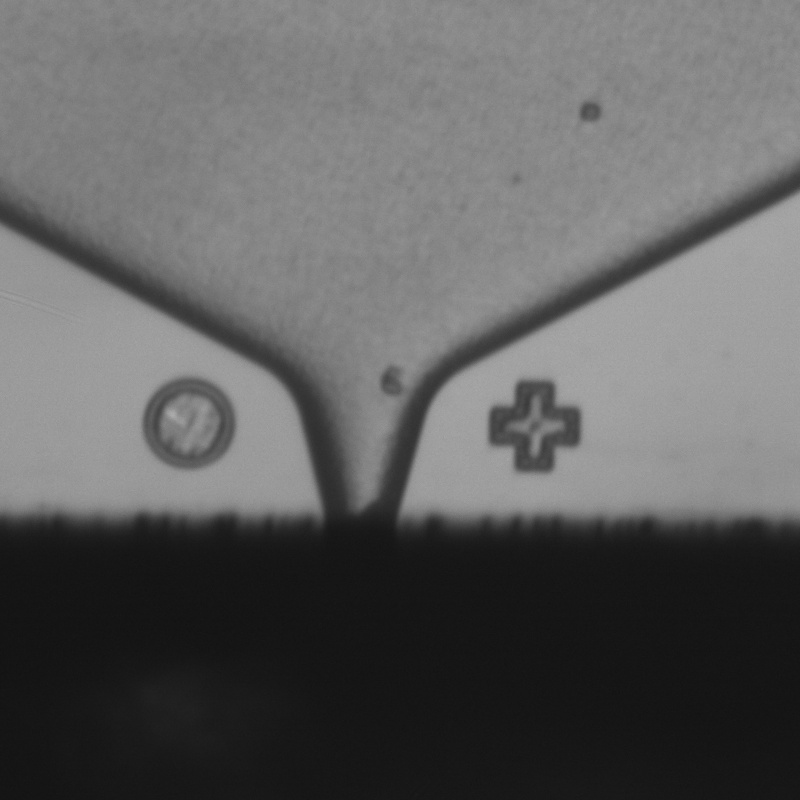

Supplement: Supplementary file 6 — Supplementary Data 3 [file 42003_2021_1661_MOESM6_ESM.zip › Supplementary Data 3 corrected/K_11_E.jpg]

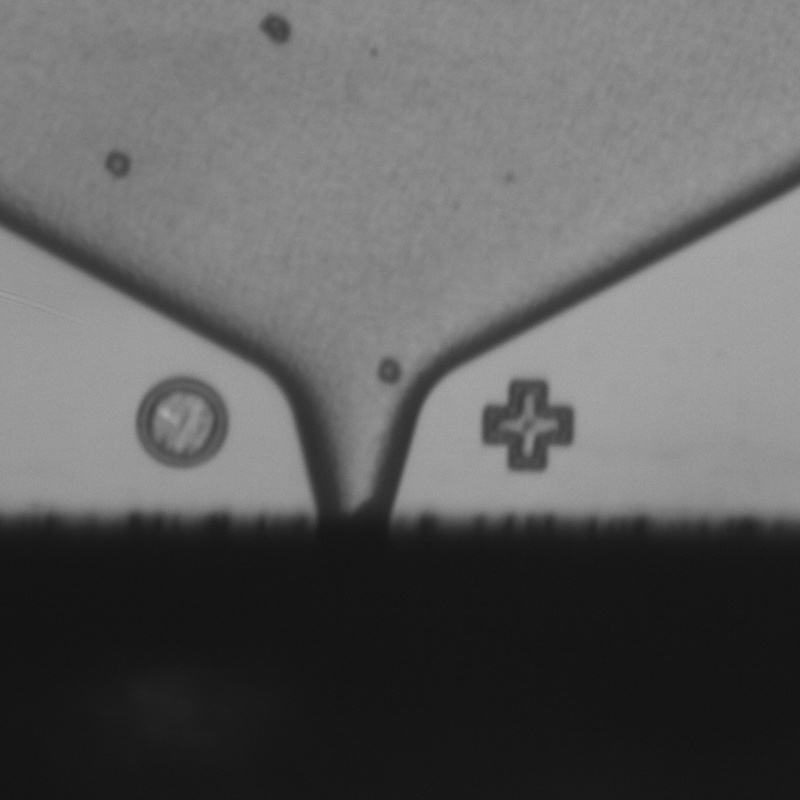

Supplement: Supplementary file 6 — Supplementary Data 3 [file 42003_2021_1661_MOESM6_ESM.zip › Supplementary Data 3 corrected/K_17_C.jpg]

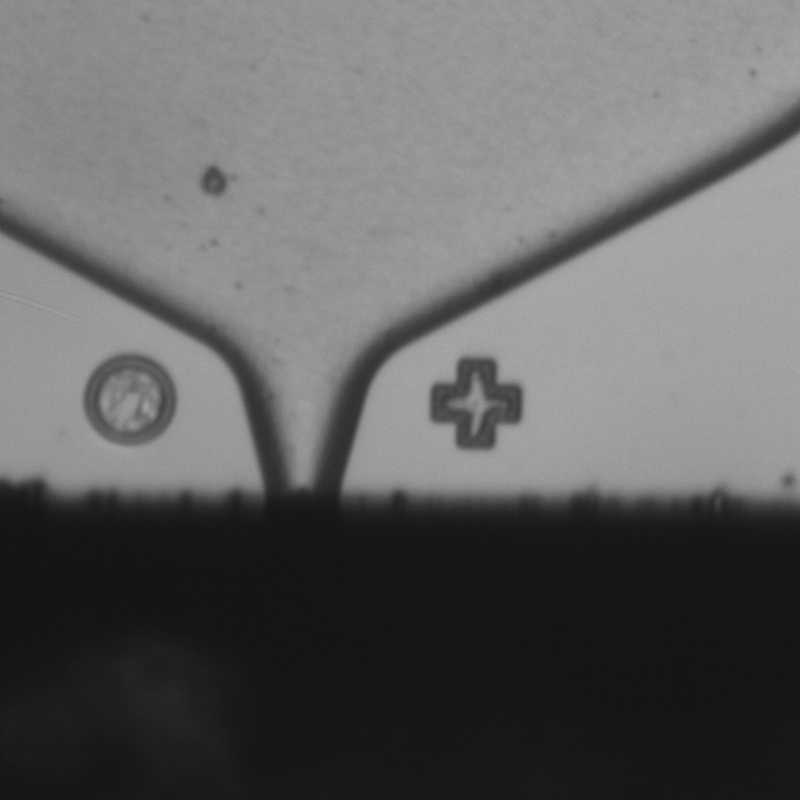

Supplement: Supplementary file 6 — Supplementary Data 3 [file 42003_2021_1661_MOESM6_ESM.zip › Supplementary Data 3 corrected/O_06_A.jpg]

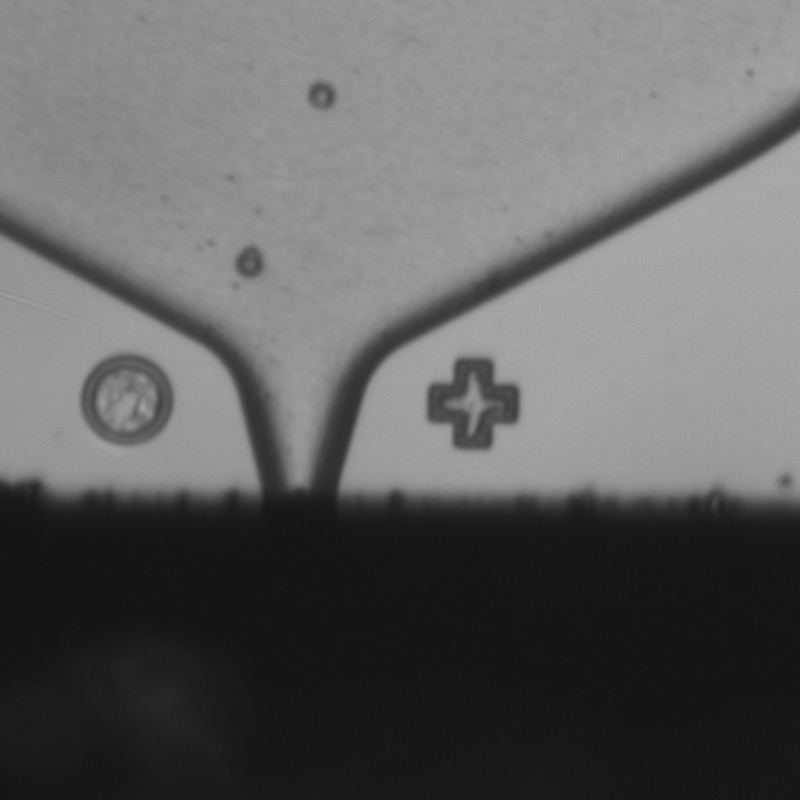

Supplement: Supplementary file 6 — Supplementary Data 3 [file 42003_2021_1661_MOESM6_ESM.zip › Supplementary Data 3 corrected/O_20_B.jpg]

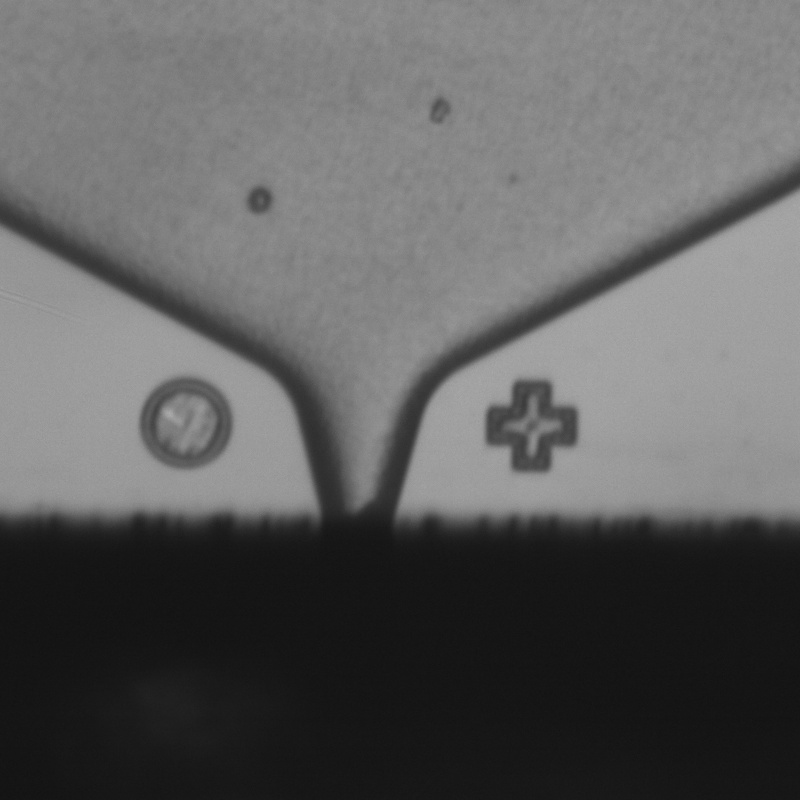

Supplement: Supplementary file 6 — Supplementary Data 3 [file 42003_2021_1661_MOESM6_ESM.zip › Supplementary Data 3 corrected/K_15_A.jpg]

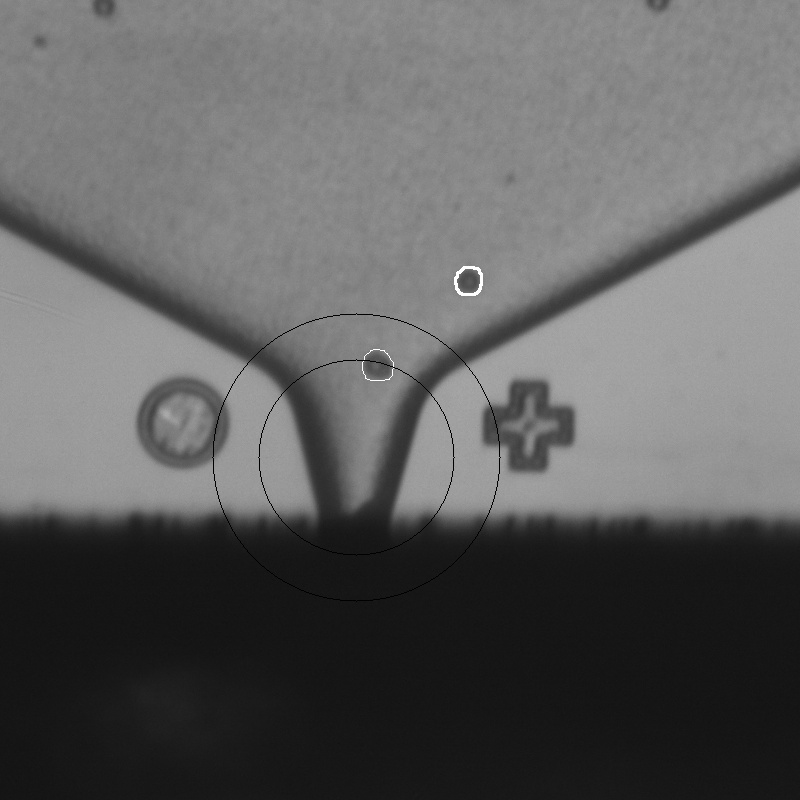

Supplement: Supplementary file 6 — Supplementary Data 3 [file 42003_2021_1661_MOESM6_ESM.zip › Supplementary Data 3 corrected/K_08_D.jpg]

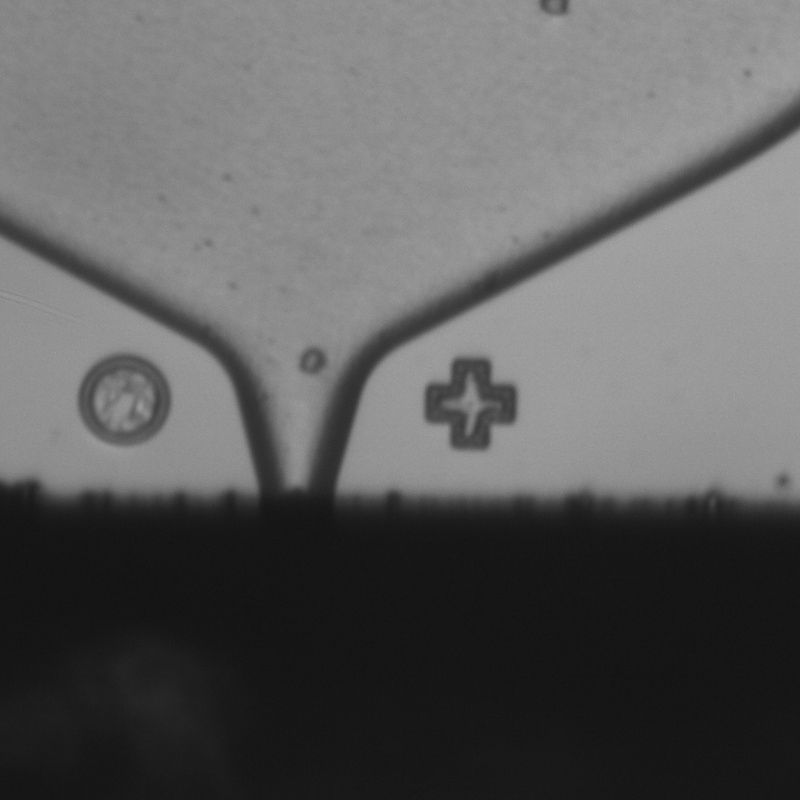

Supplement: Supplementary file 6 — Supplementary Data 3 [file 42003_2021_1661_MOESM6_ESM.zip › Supplementary Data 3 corrected/O_04_C.jpg]

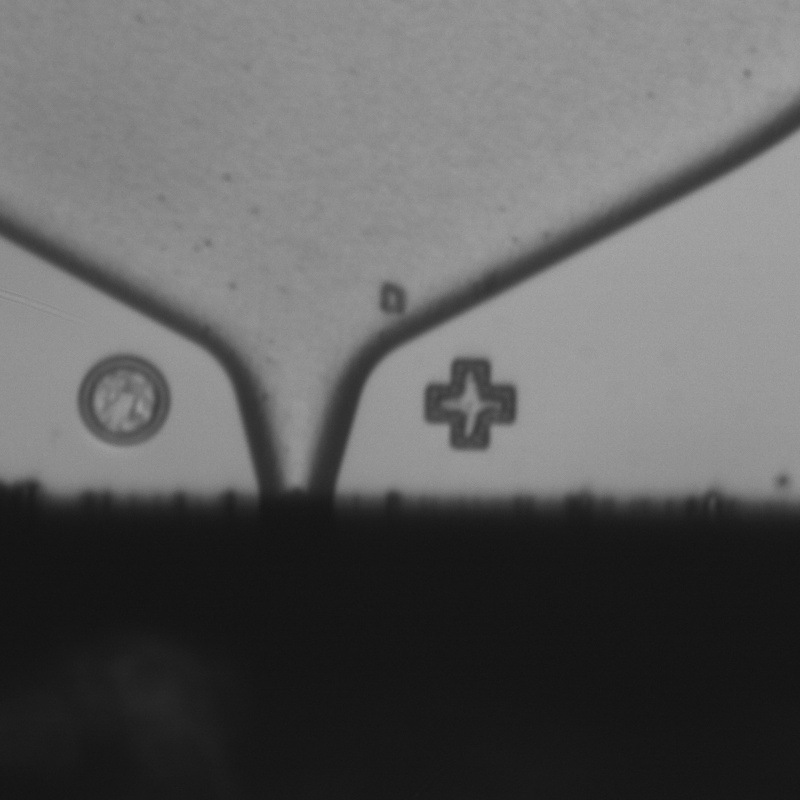

Supplement: Supplementary file 6 — Supplementary Data 3 [file 42003_2021_1661_MOESM6_ESM.zip › Supplementary Data 3 corrected/O_04_B.jpg]

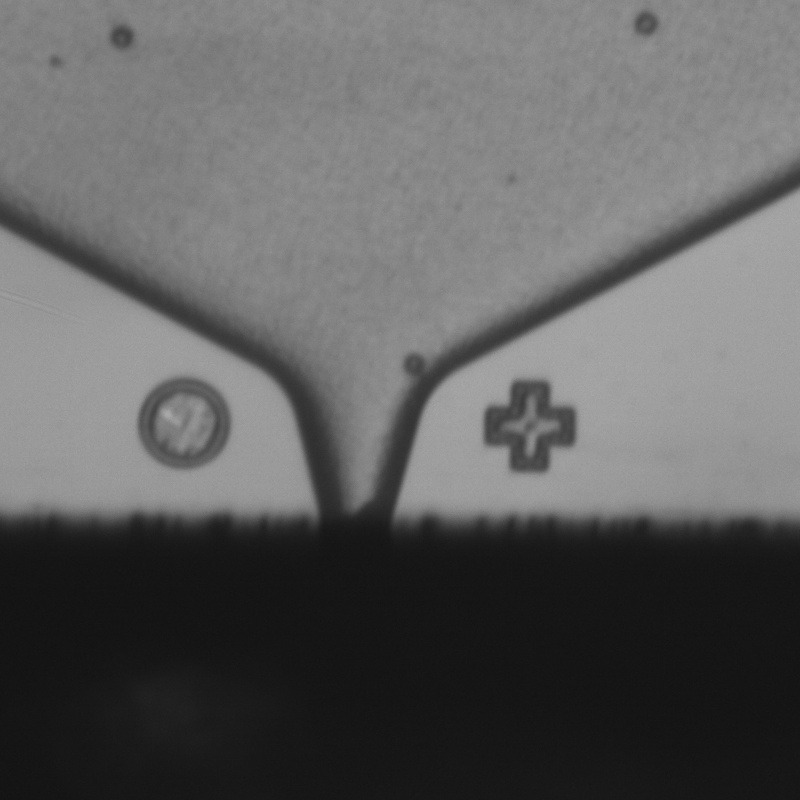

Supplement: Supplementary file 6 — Supplementary Data 3 [file 42003_2021_1661_MOESM6_ESM.zip › Supplementary Data 3 corrected/K_08_E.jpg]

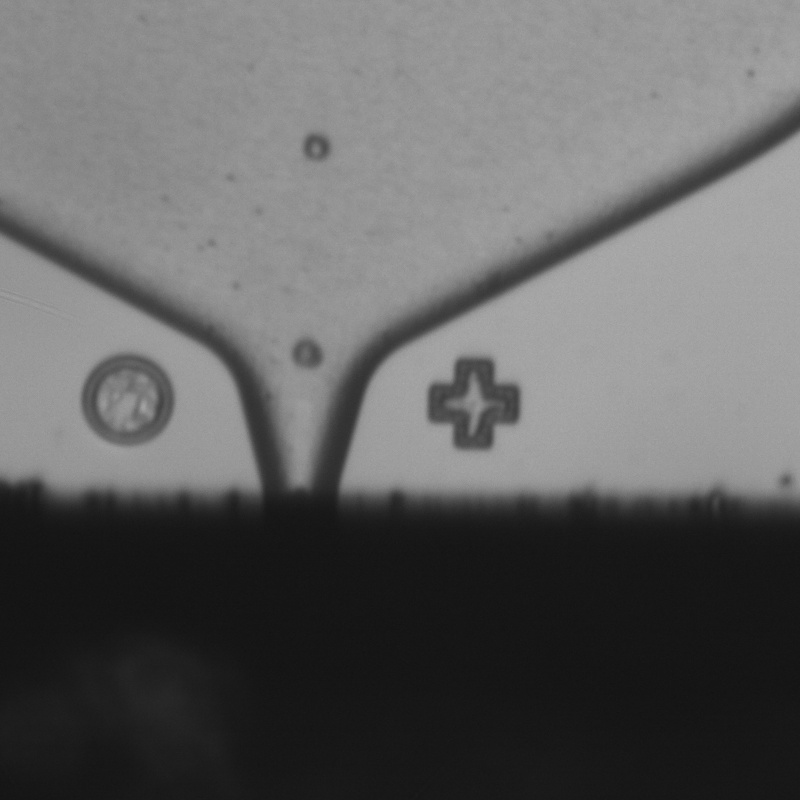

Supplement: Supplementary file 6 — Supplementary Data 3 [file 42003_2021_1661_MOESM6_ESM.zip › Supplementary Data 3 corrected/O_20_C.jpg]

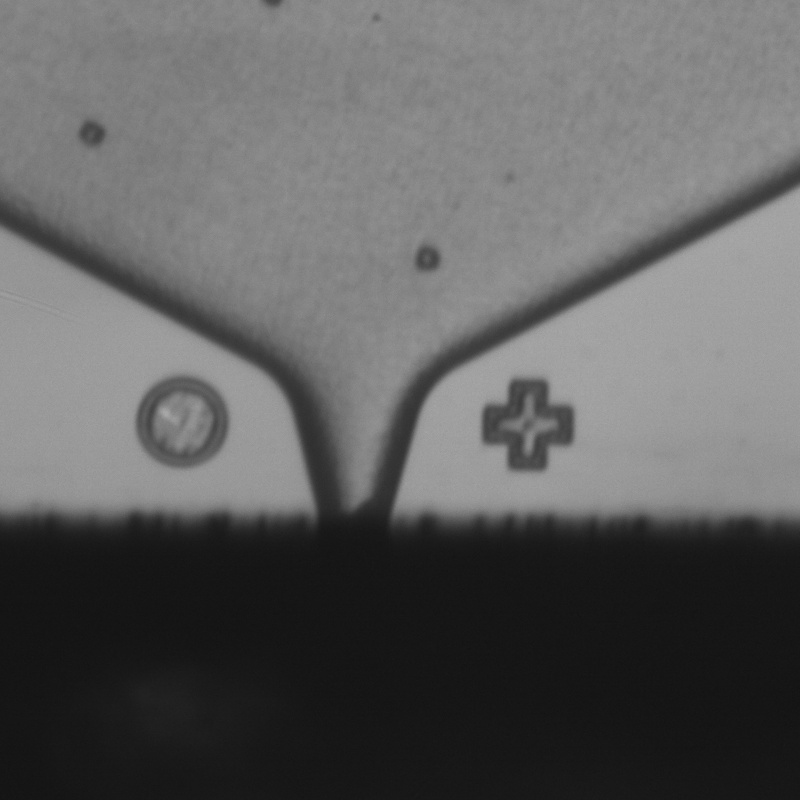

Supplement: Supplementary file 6 — Supplementary Data 3 [file 42003_2021_1661_MOESM6_ESM.zip › Supplementary Data 3 corrected/K_17_B.jpg]

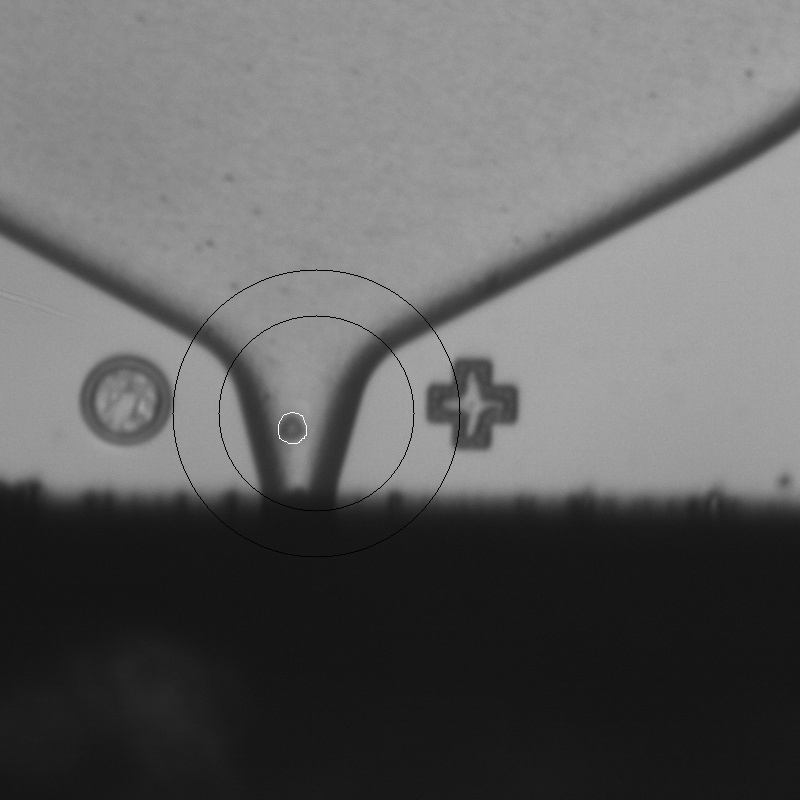

Supplement: Supplementary file 6 — Supplementary Data 3 [file 42003_2021_1661_MOESM6_ESM.zip › Supplementary Data 3 corrected/O_02_D.jpg]

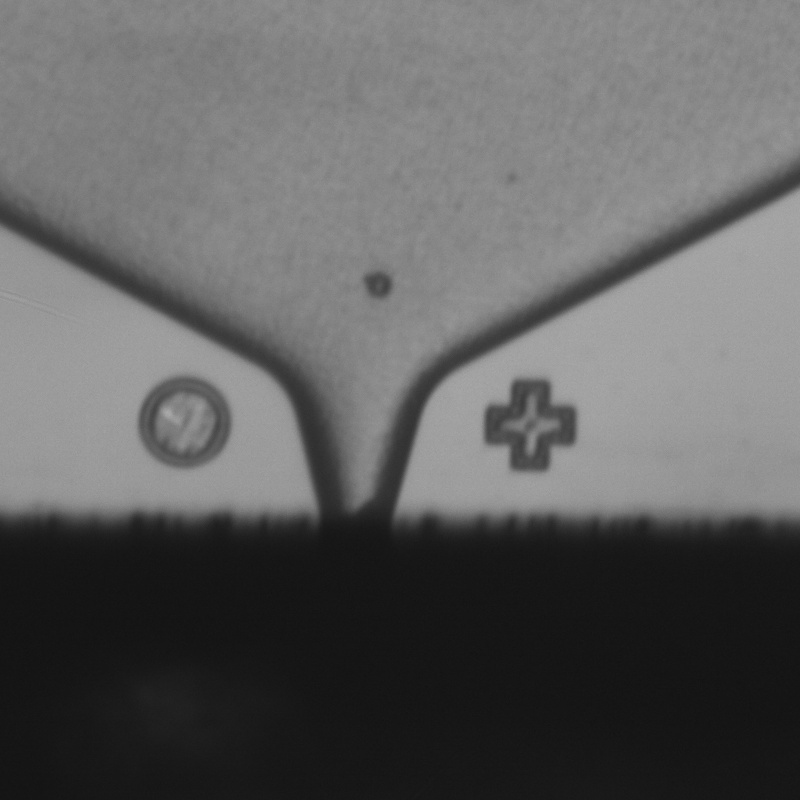

Supplement: Supplementary file 6 — Supplementary Data 3 [file 42003_2021_1661_MOESM6_ESM.zip › Supplementary Data 3 corrected/K_13_B.jpg]

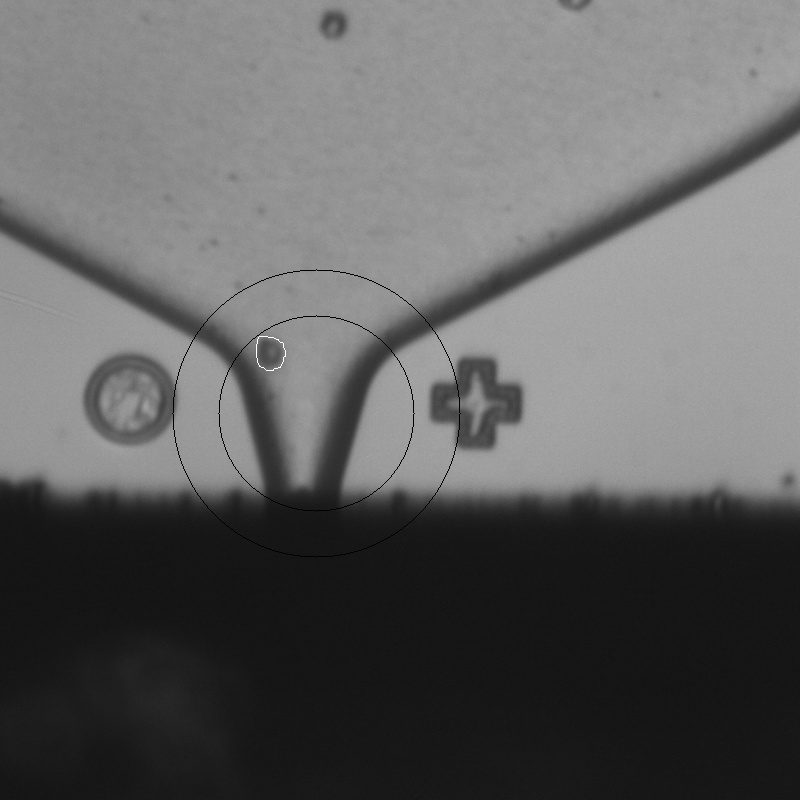

Supplement: Supplementary file 6 — Supplementary Data 3 [file 42003_2021_1661_MOESM6_ESM.zip › Supplementary Data 3 corrected/O_06_D.jpg]

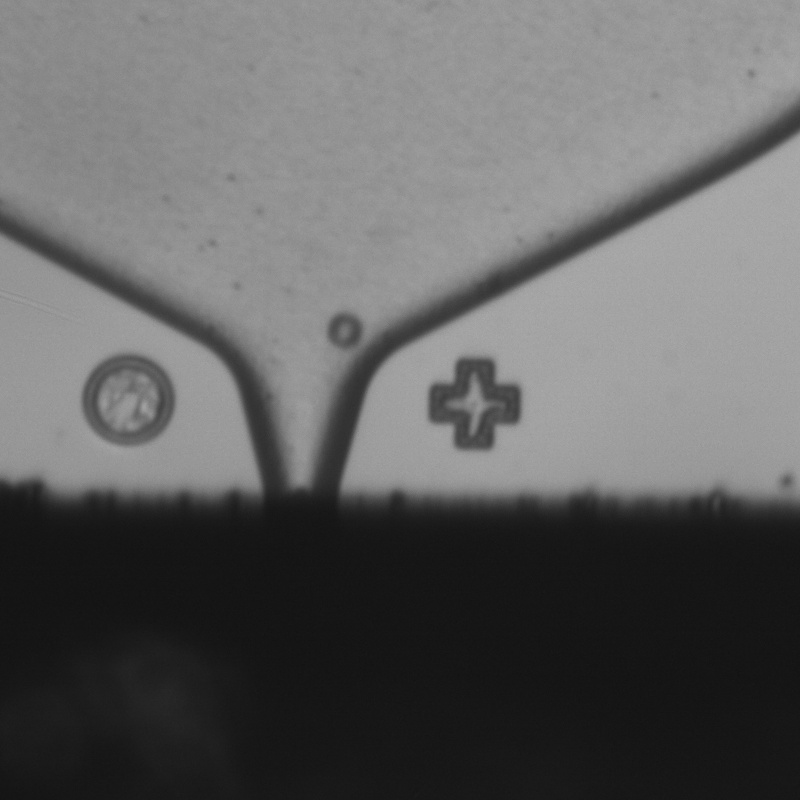

Supplement: Supplementary file 6 — Supplementary Data 3 [file 42003_2021_1661_MOESM6_ESM.zip › Supplementary Data 3 corrected/O_19_C.jpg]

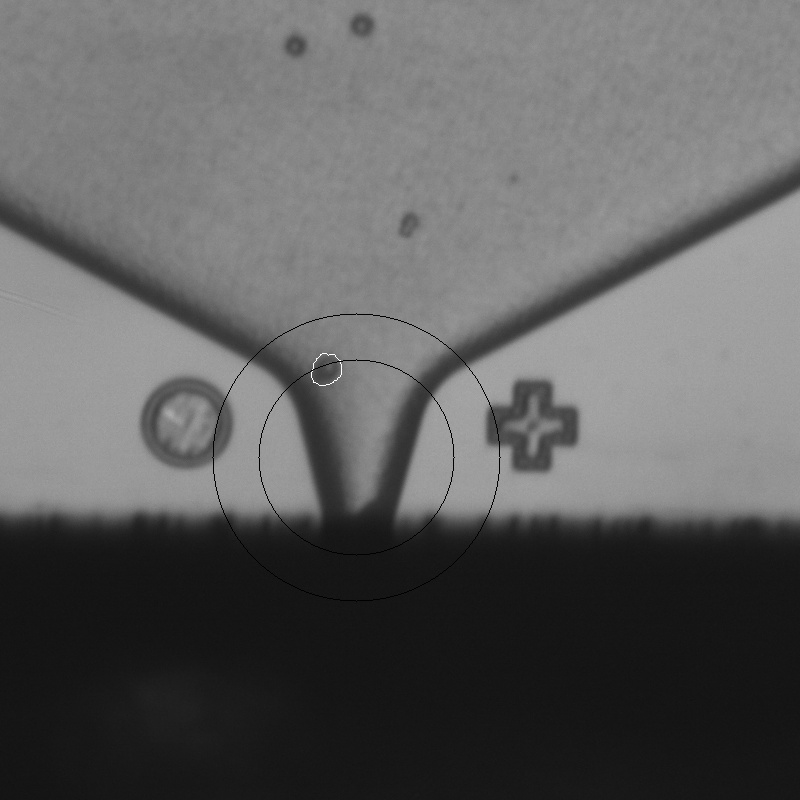

Supplement: Supplementary file 6 — Supplementary Data 3 [file 42003_2021_1661_MOESM6_ESM.zip › Supplementary Data 3 corrected/K_15_D.jpg]

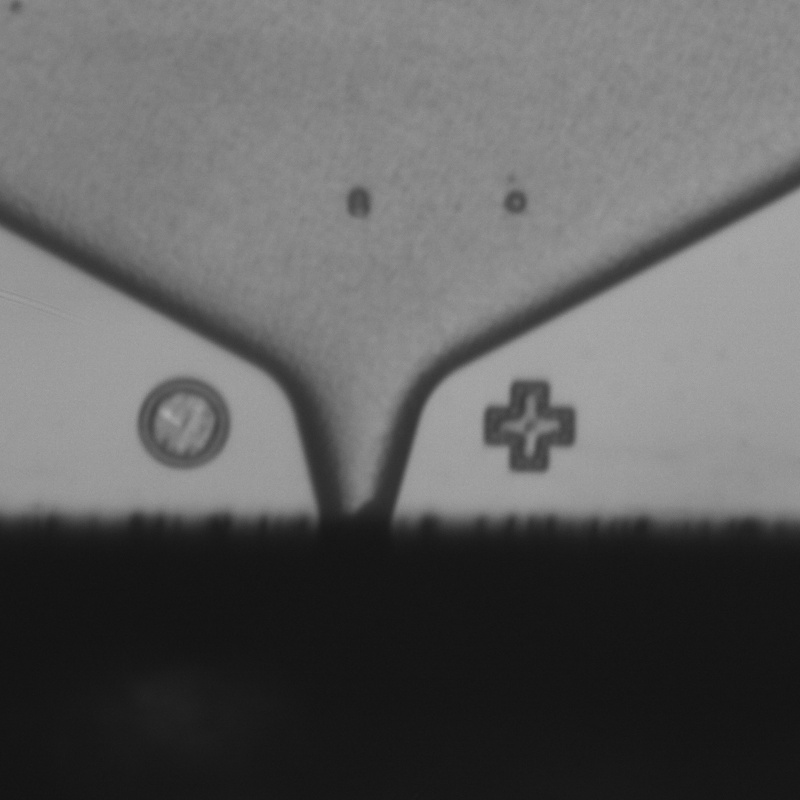

Supplement: Supplementary file 6 — Supplementary Data 3 [file 42003_2021_1661_MOESM6_ESM.zip › Supplementary Data 3 corrected/K_08_A.jpg]

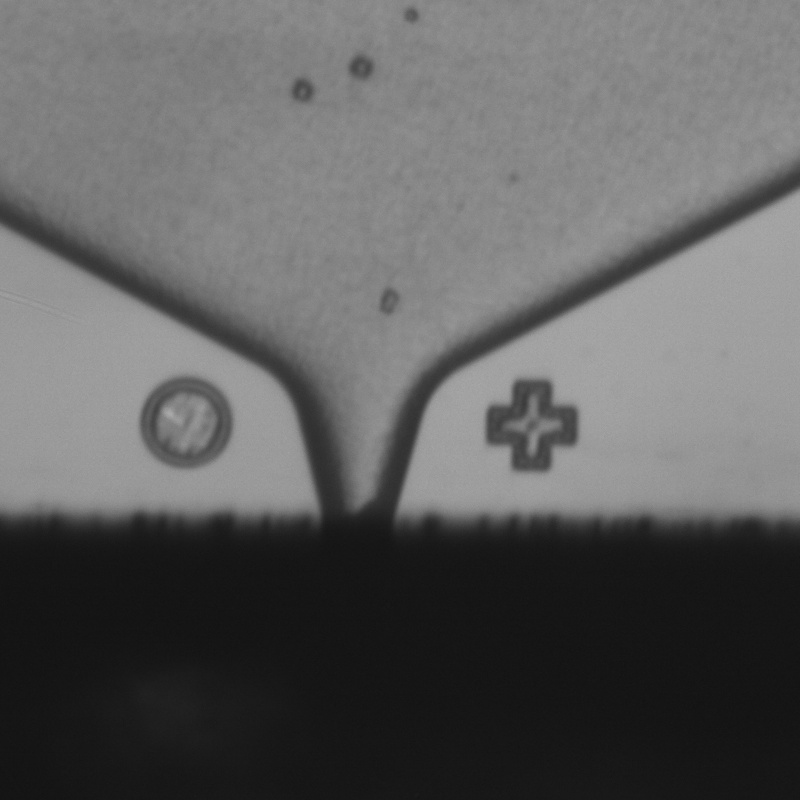

Supplement: Supplementary file 6 — Supplementary Data 3 [file 42003_2021_1661_MOESM6_ESM.zip › Supplementary Data 3 corrected/K_15_E.jpg]

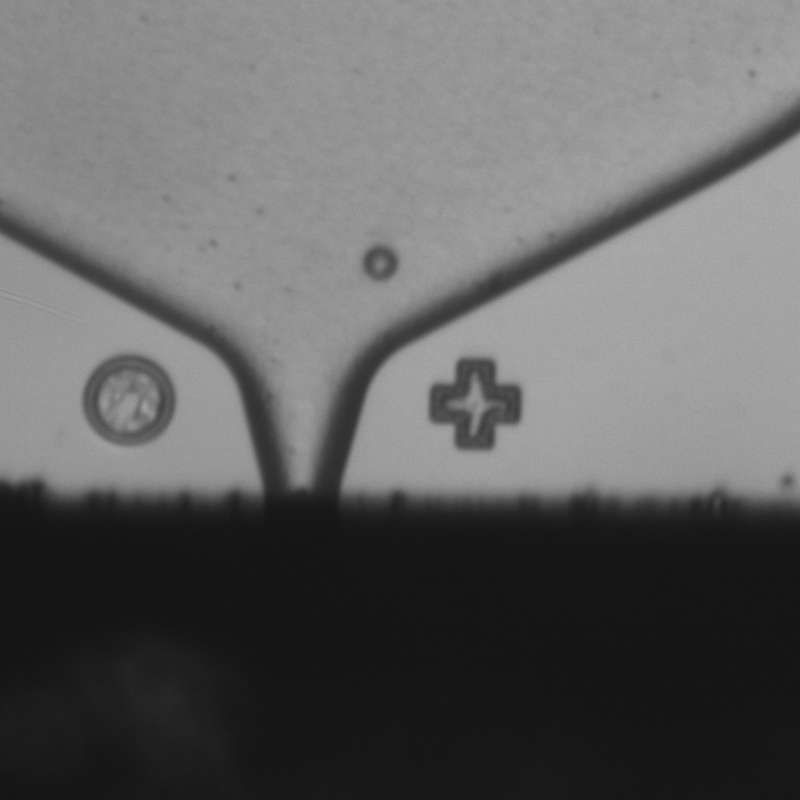

Supplement: Supplementary file 6 — Supplementary Data 3 [file 42003_2021_1661_MOESM6_ESM.zip › Supplementary Data 3 corrected/O_19_B.jpg]

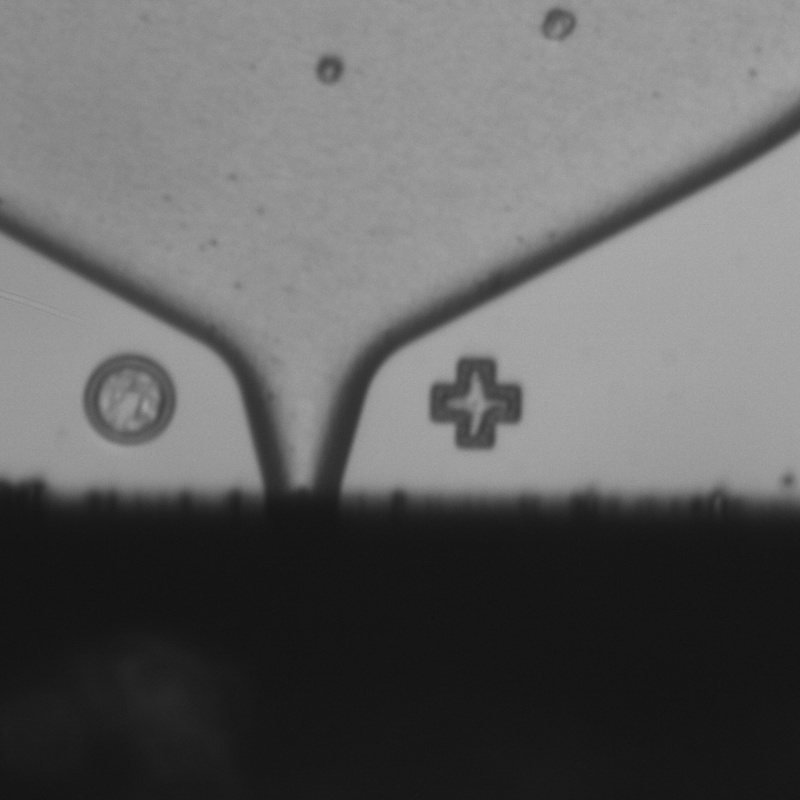

Supplement: Supplementary file 6 — Supplementary Data 3 [file 42003_2021_1661_MOESM6_ESM.zip › Supplementary Data 3 corrected/O_06_E.jpg]

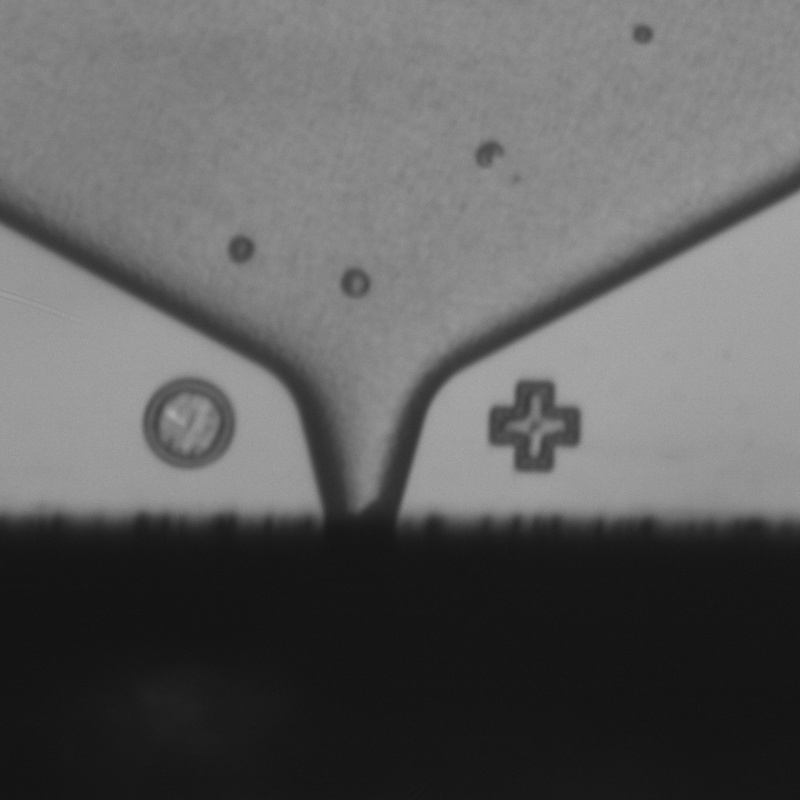

Supplement: Supplementary file 6 — Supplementary Data 3 [file 42003_2021_1661_MOESM6_ESM.zip › Supplementary Data 3 corrected/K_11_A.jpg]

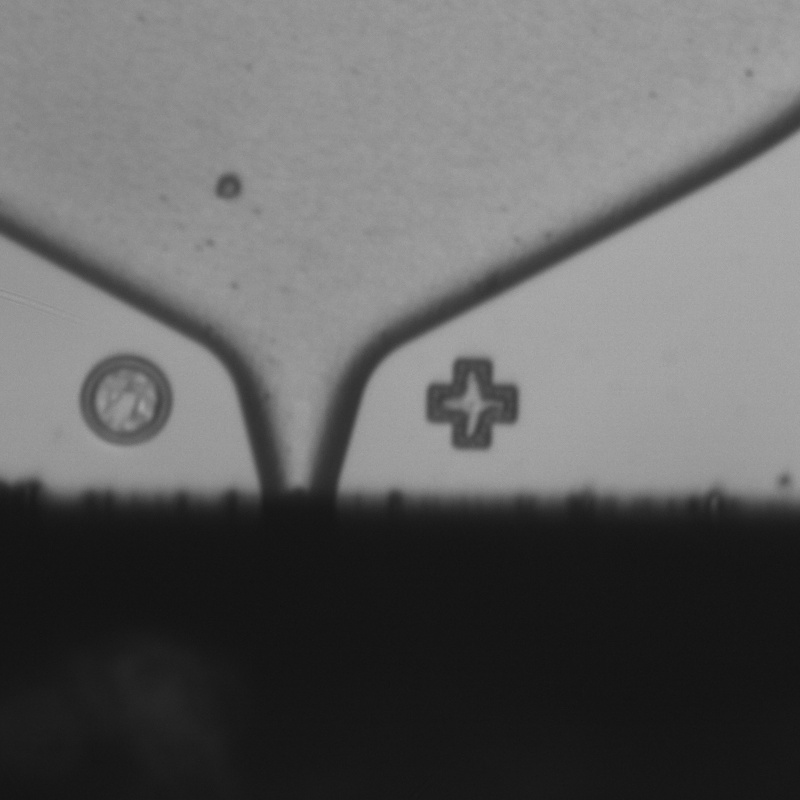

Supplement: Supplementary file 6 — Supplementary Data 3 [file 42003_2021_1661_MOESM6_ESM.zip › Supplementary Data 3 corrected/O_02_A.jpg]

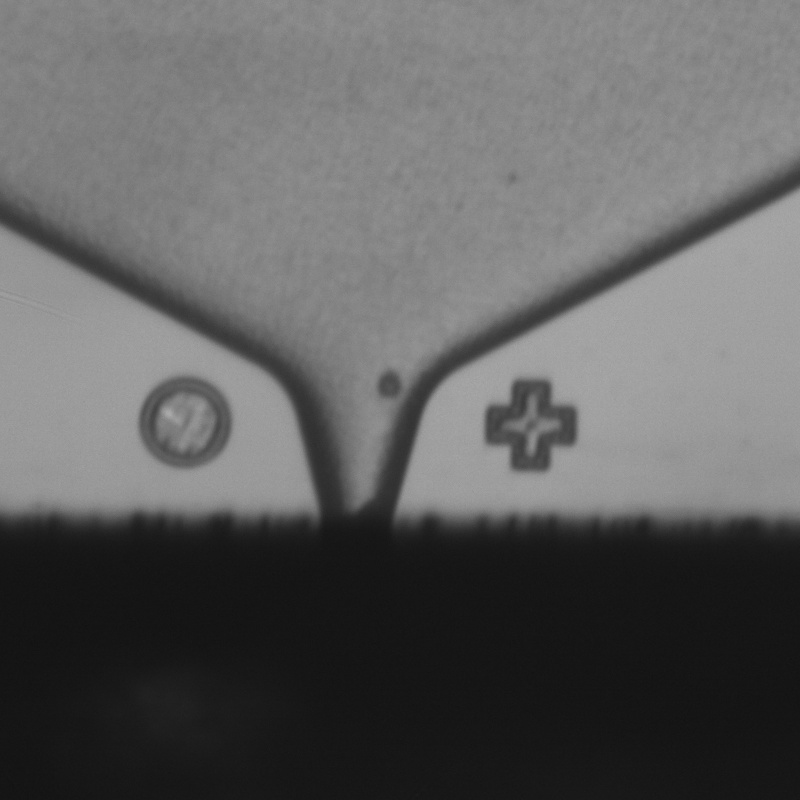

Supplement: Supplementary file 6 — Supplementary Data 3 [file 42003_2021_1661_MOESM6_ESM.zip › Supplementary Data 3 corrected/K_13_C.jpg]

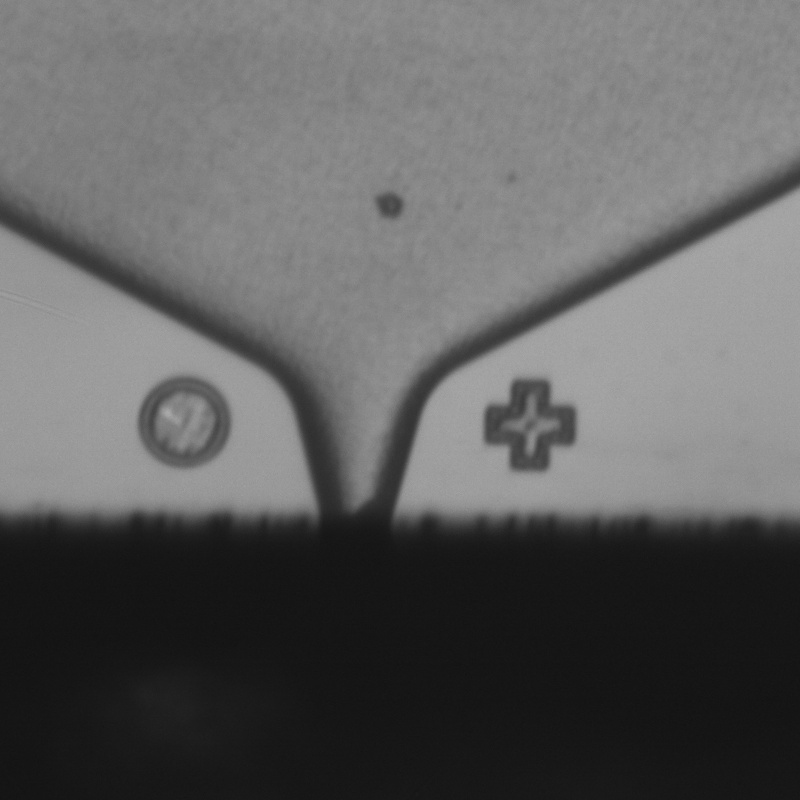

Supplement: Supplementary file 6 — Supplementary Data 3 [file 42003_2021_1661_MOESM6_ESM.zip › Supplementary Data 3 corrected/K_13_A.jpg]

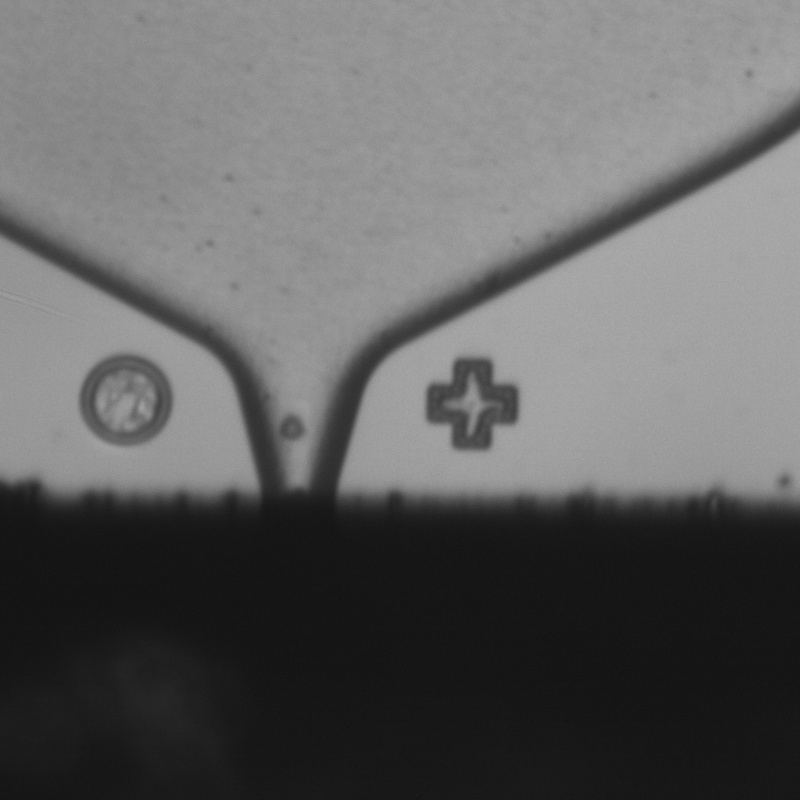

Supplement: Supplementary file 6 — Supplementary Data 3 [file 42003_2021_1661_MOESM6_ESM.zip › Supplementary Data 3 corrected/O_02_C.jpg]

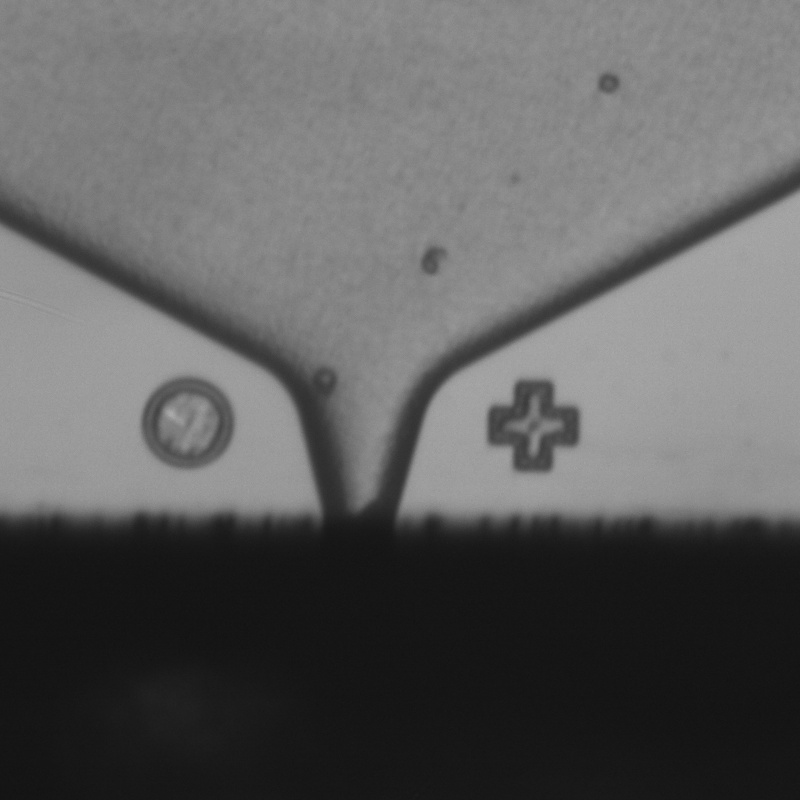

Supplement: Supplementary file 6 — Supplementary Data 3 [file 42003_2021_1661_MOESM6_ESM.zip › Supplementary Data 3 corrected/K_11_C.jpg]

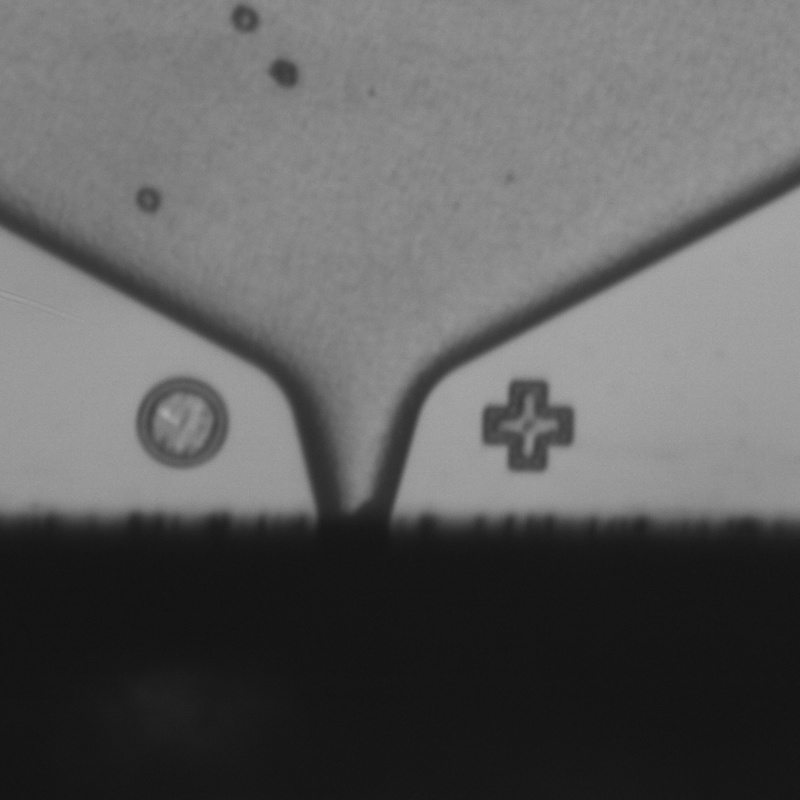

Supplement: Supplementary file 6 — Supplementary Data 3 [file 42003_2021_1661_MOESM6_ESM.zip › Supplementary Data 3 corrected/K_17_E.jpg]

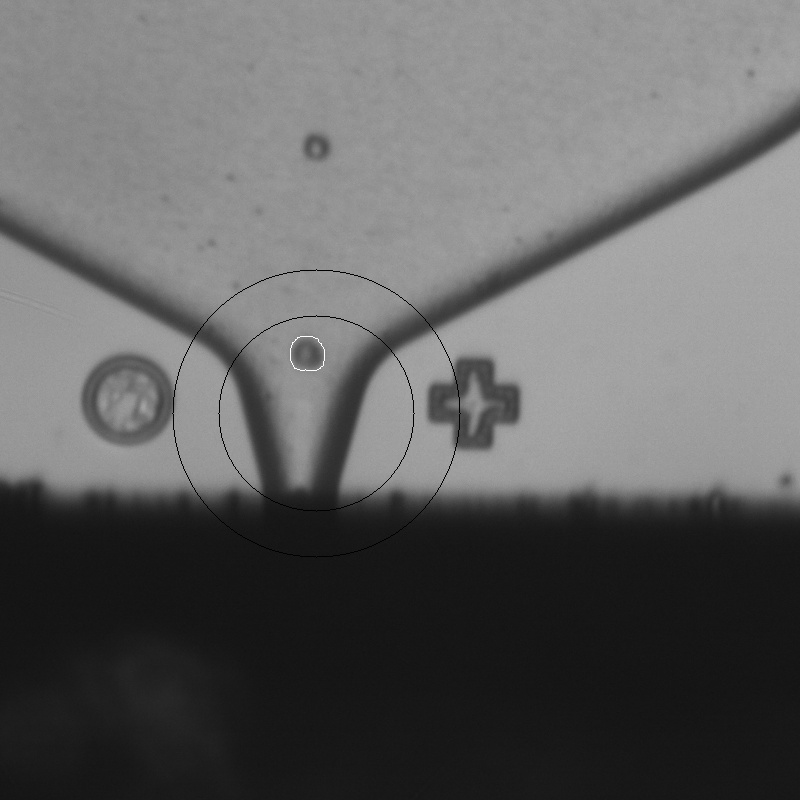

Supplement: Supplementary file 6 — Supplementary Data 3 [file 42003_2021_1661_MOESM6_ESM.zip › Supplementary Data 3 corrected/O_20_D.jpg]

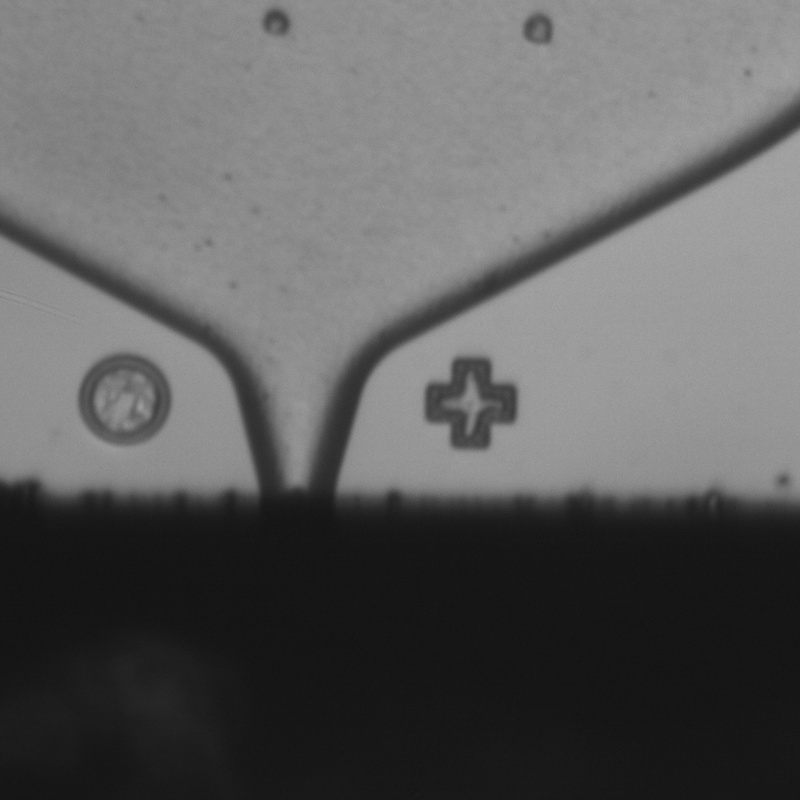

Supplement: Supplementary file 6 — Supplementary Data 3 [file 42003_2021_1661_MOESM6_ESM.zip › Supplementary Data 3 corrected/O_04_E.jpg]

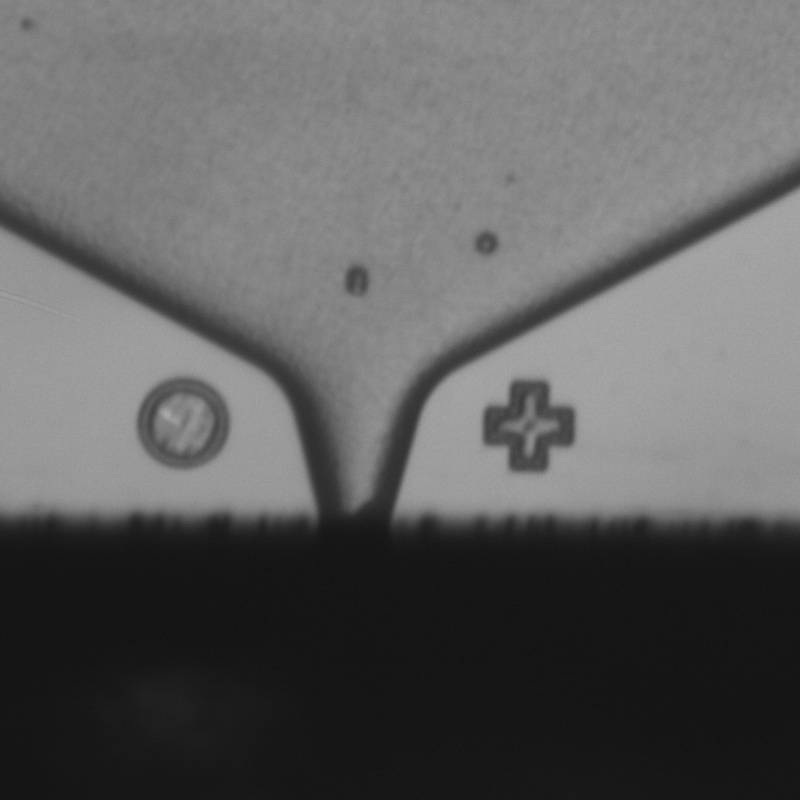

Supplement: Supplementary file 6 — Supplementary Data 3 [file 42003_2021_1661_MOESM6_ESM.zip › Supplementary Data 3 corrected/K_08_B.jpg]

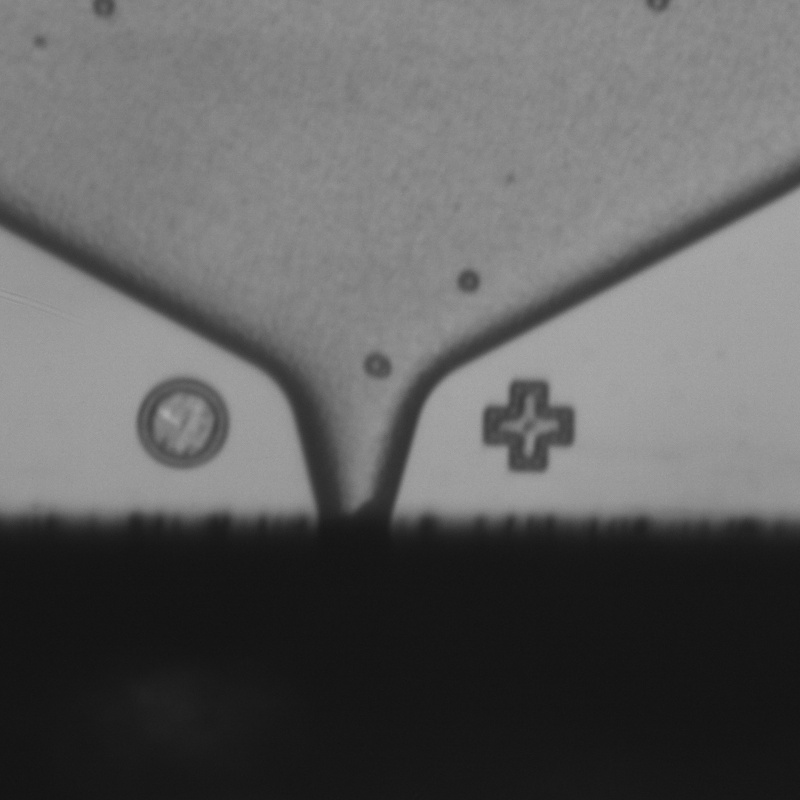

Supplement: Supplementary file 6 — Supplementary Data 3 [file 42003_2021_1661_MOESM6_ESM.zip › Supplementary Data 3 corrected/K_08_C.jpg]

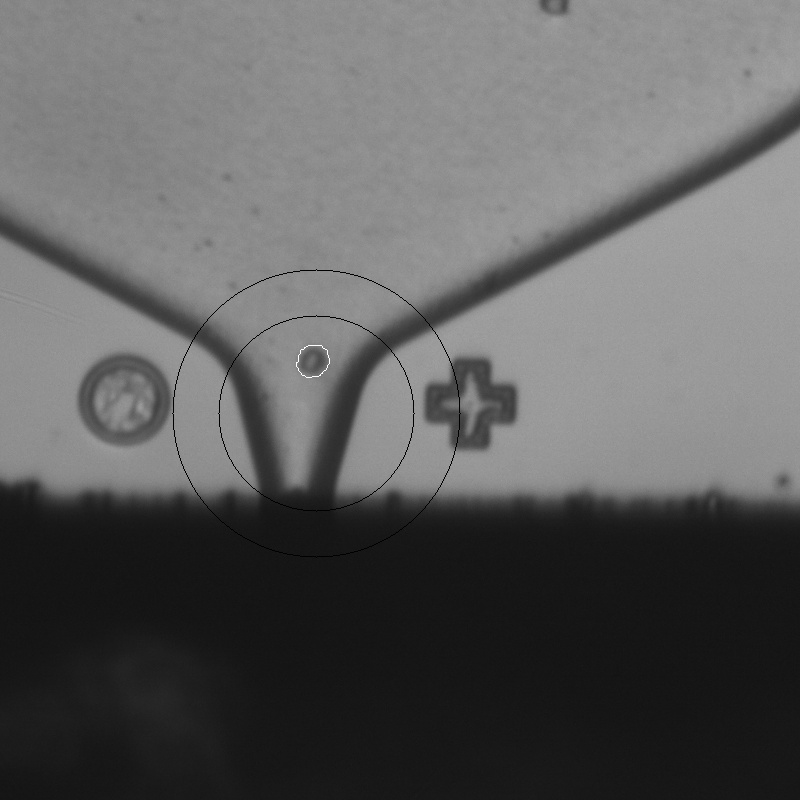

Supplement: Supplementary file 6 — Supplementary Data 3 [file 42003_2021_1661_MOESM6_ESM.zip › Supplementary Data 3 corrected/O_04_D.jpg]

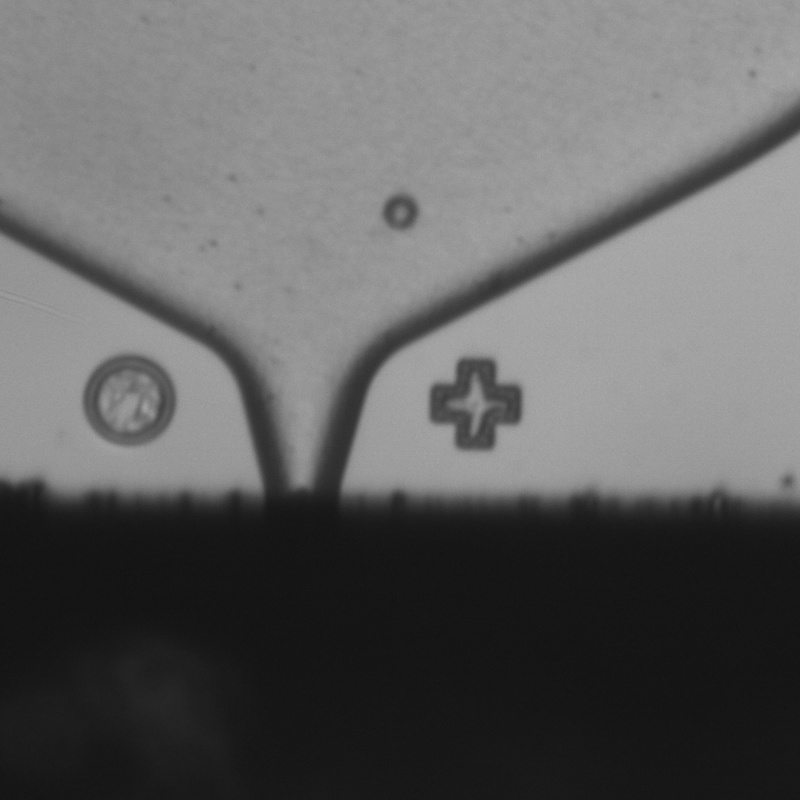

Supplement: Supplementary file 6 — Supplementary Data 3 [file 42003_2021_1661_MOESM6_ESM.zip › Supplementary Data 3 corrected/O_19_A.jpg]

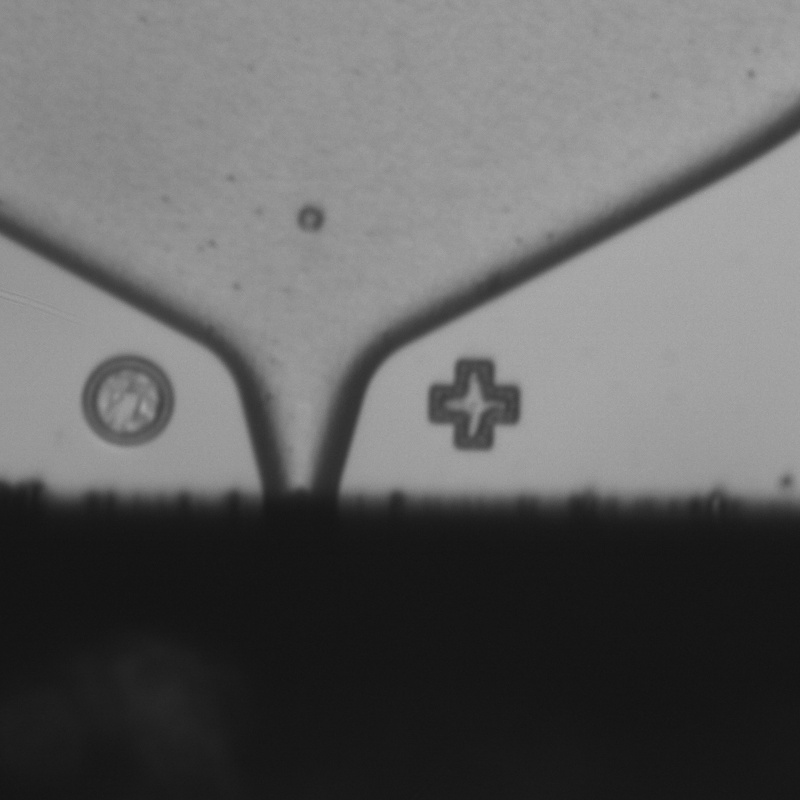

Supplement: Supplementary file 6 — Supplementary Data 3 [file 42003_2021_1661_MOESM6_ESM.zip › Supplementary Data 3 corrected/O_20_E.jpg]

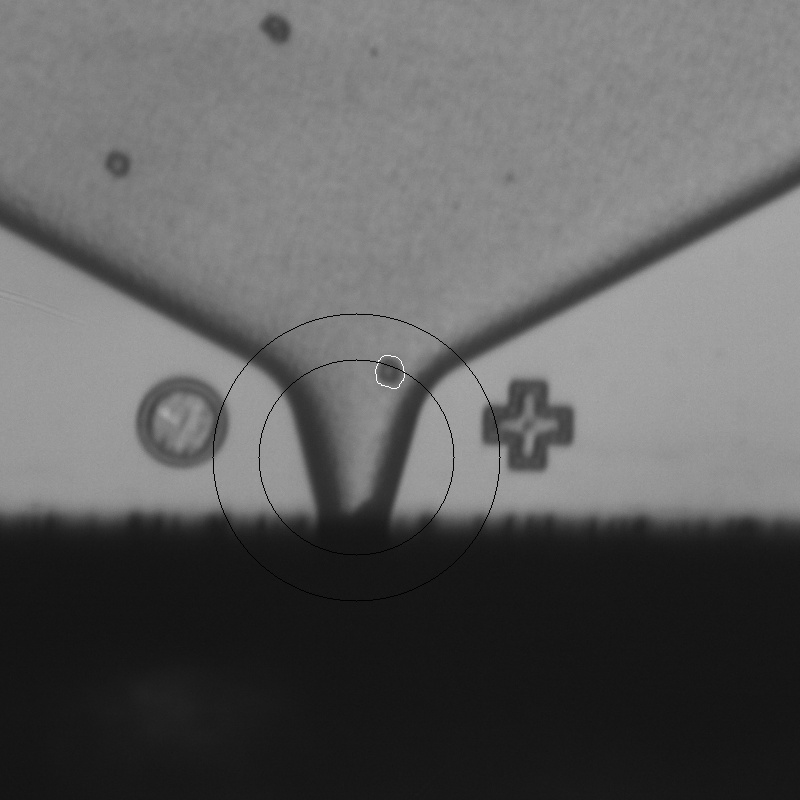

Supplement: Supplementary file 6 — Supplementary Data 3 [file 42003_2021_1661_MOESM6_ESM.zip › Supplementary Data 3 corrected/K_17_D.jpg]

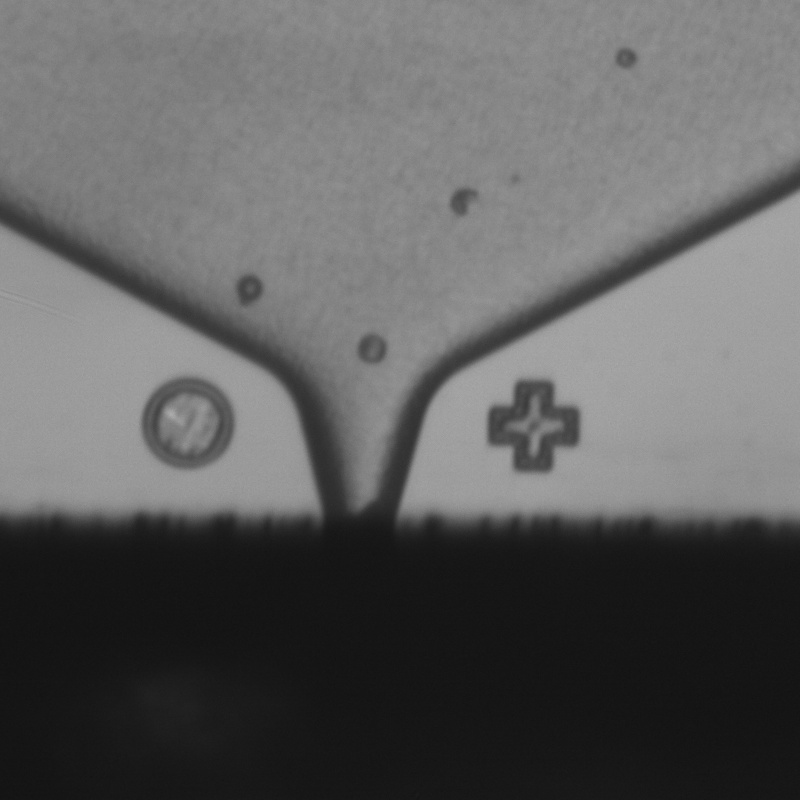

Supplement: Supplementary file 6 — Supplementary Data 3 [file 42003_2021_1661_MOESM6_ESM.zip › Supplementary Data 3 corrected/K_11_B.jpg]

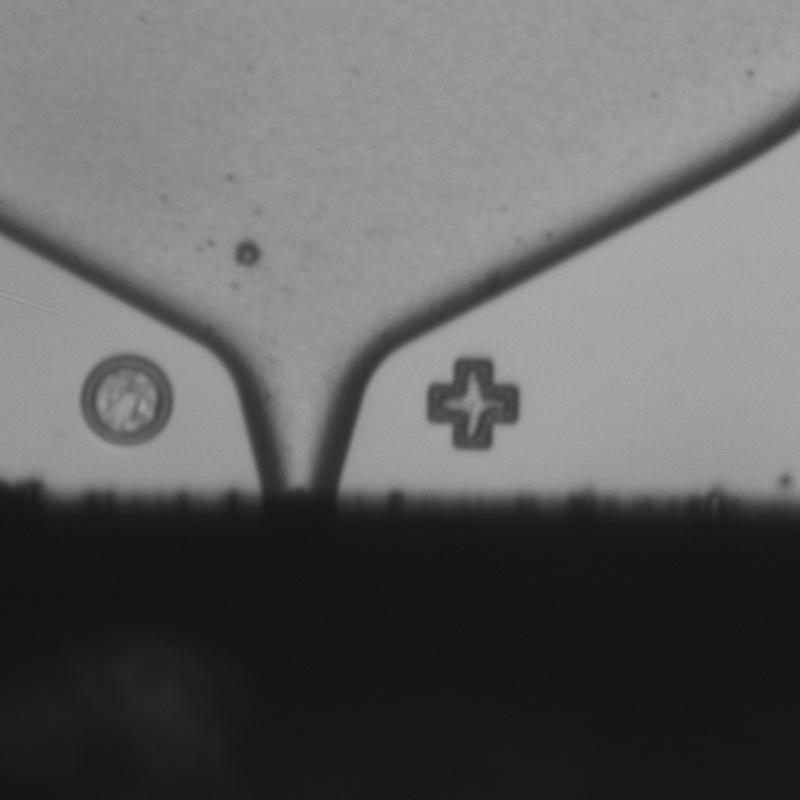

Supplement: Supplementary file 6 — Supplementary Data 3 [file 42003_2021_1661_MOESM6_ESM.zip › Supplementary Data 3 corrected/O_02_B.jpg]

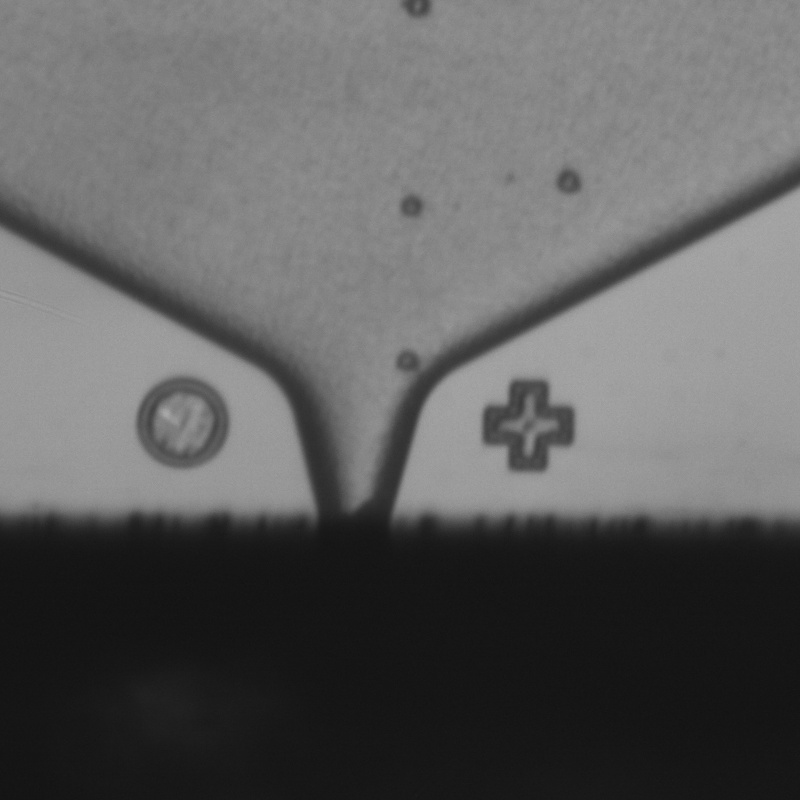

Supplement: Supplementary file 6 — Supplementary Data 3 [file 42003_2021_1661_MOESM6_ESM.zip › Supplementary Data 3 corrected/K_14_A.jpg]

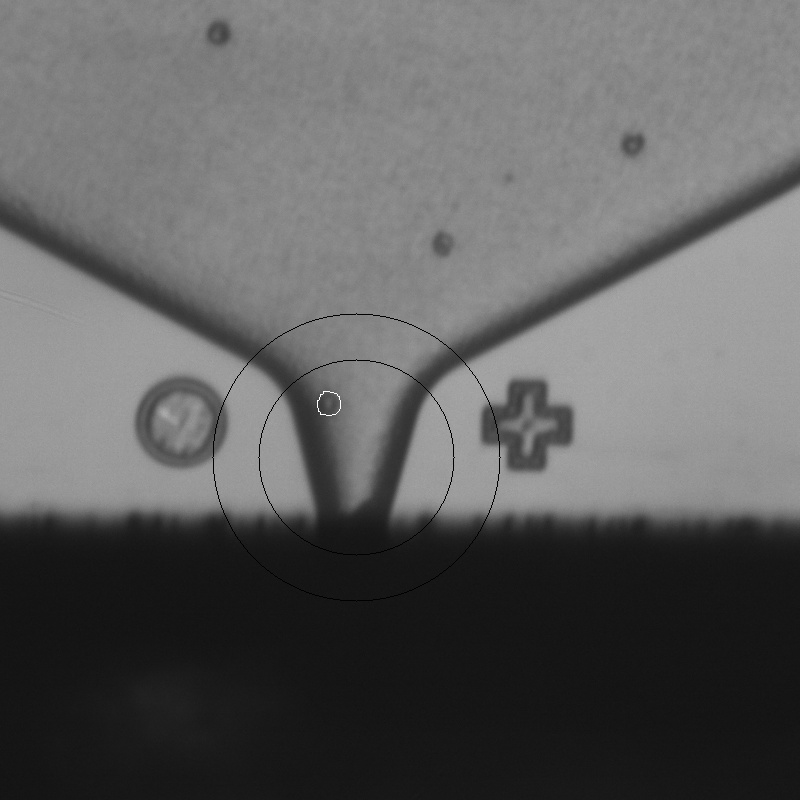

Supplement: Supplementary file 6 — Supplementary Data 3 [file 42003_2021_1661_MOESM6_ESM.zip › Supplementary Data 3 corrected/K_09_D.jpg]

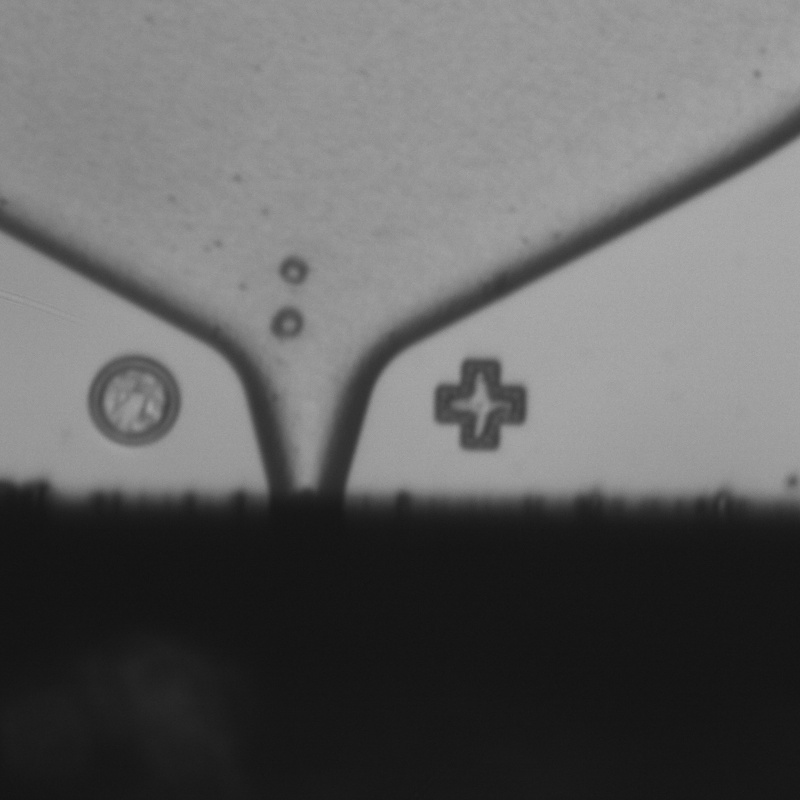

Supplement: Supplementary file 6 — Supplementary Data 3 [file 42003_2021_1661_MOESM6_ESM.zip › Supplementary Data 3 corrected/O_05_C.jpg]

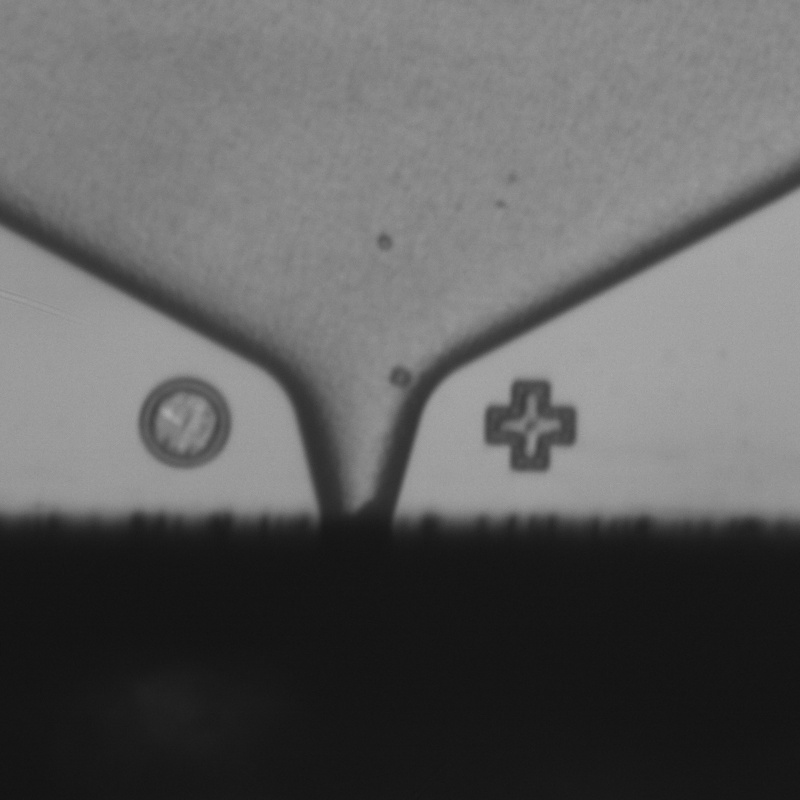

Supplement: Supplementary file 6 — Supplementary Data 3 [file 42003_2021_1661_MOESM6_ESM.zip › Supplementary Data 3 corrected/K_16_C.jpg]

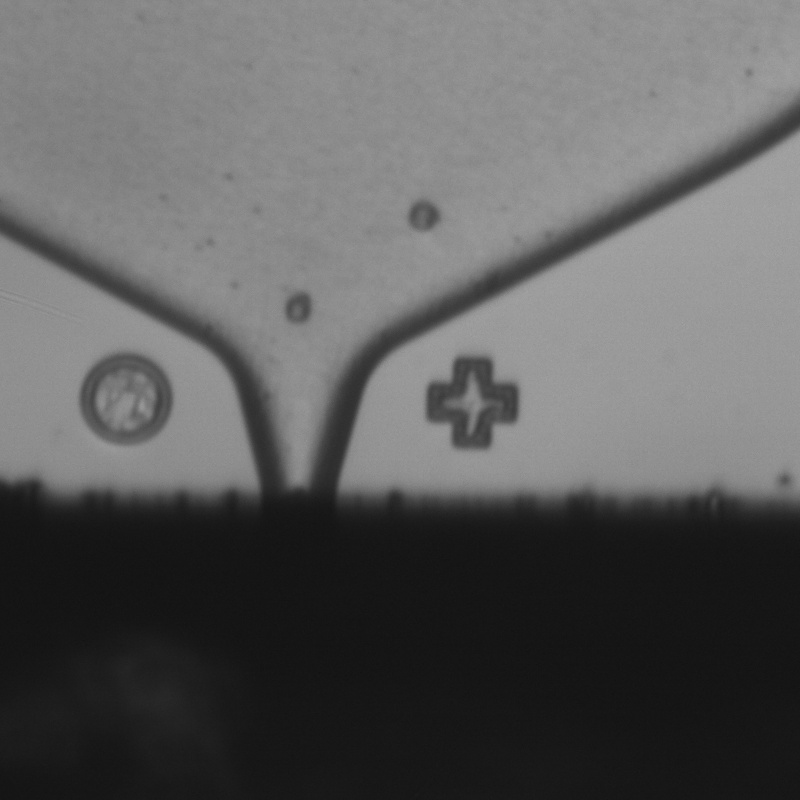

Supplement: Supplementary file 6 — Supplementary Data 3 [file 42003_2021_1661_MOESM6_ESM.zip › Supplementary Data 3 corrected/O_07_A.jpg]

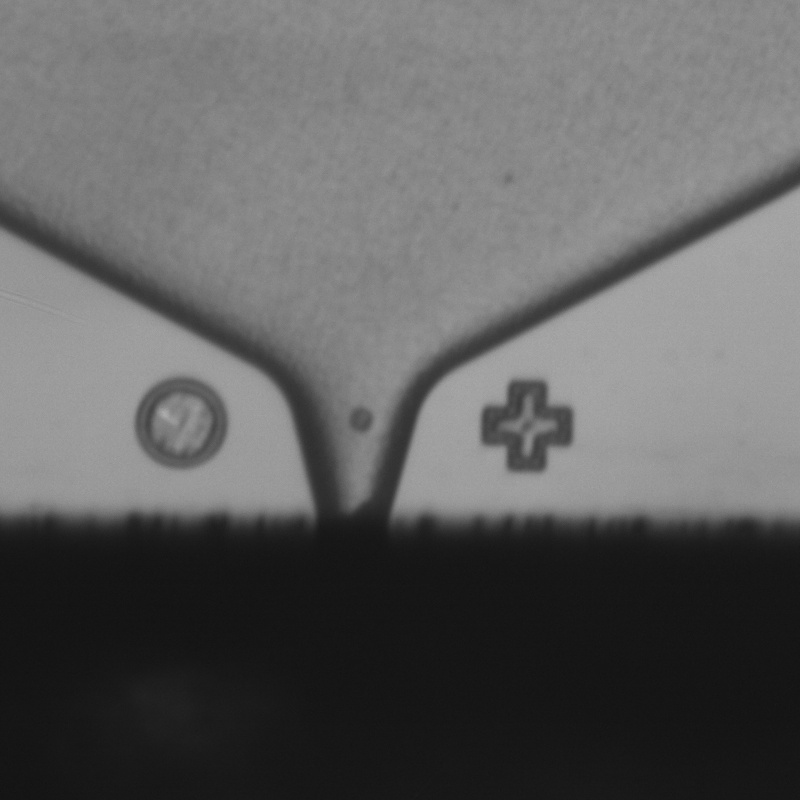

Supplement: Supplementary file 6 — Supplementary Data 3 [file 42003_2021_1661_MOESM6_ESM.zip › Supplementary Data 3 corrected/K_10_E.jpg]

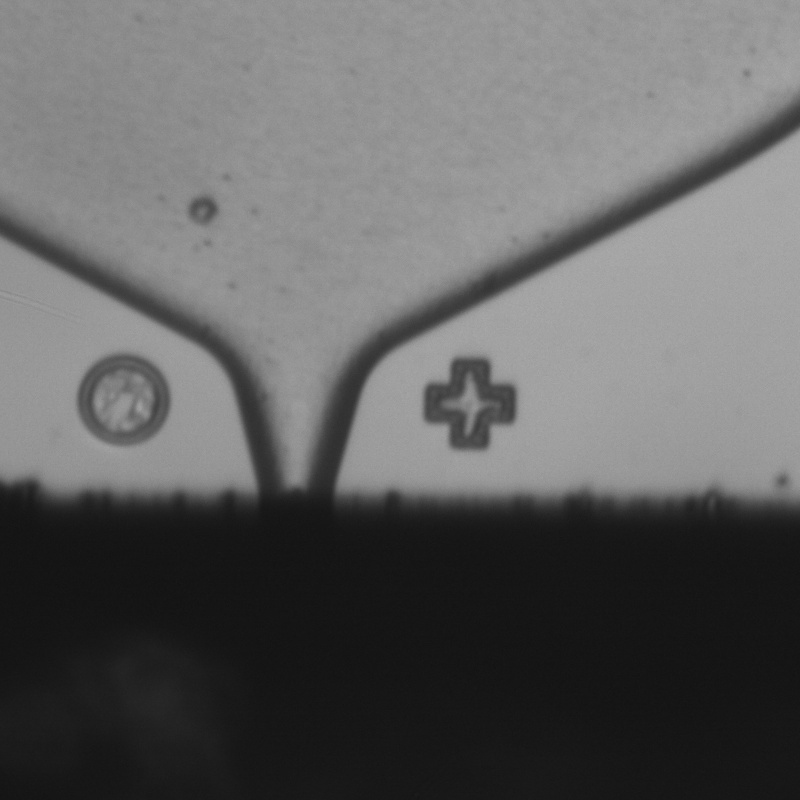

Supplement: Supplementary file 6 — Supplementary Data 3 [file 42003_2021_1661_MOESM6_ESM.zip › Supplementary Data 3 corrected/O_03_E.jpg]

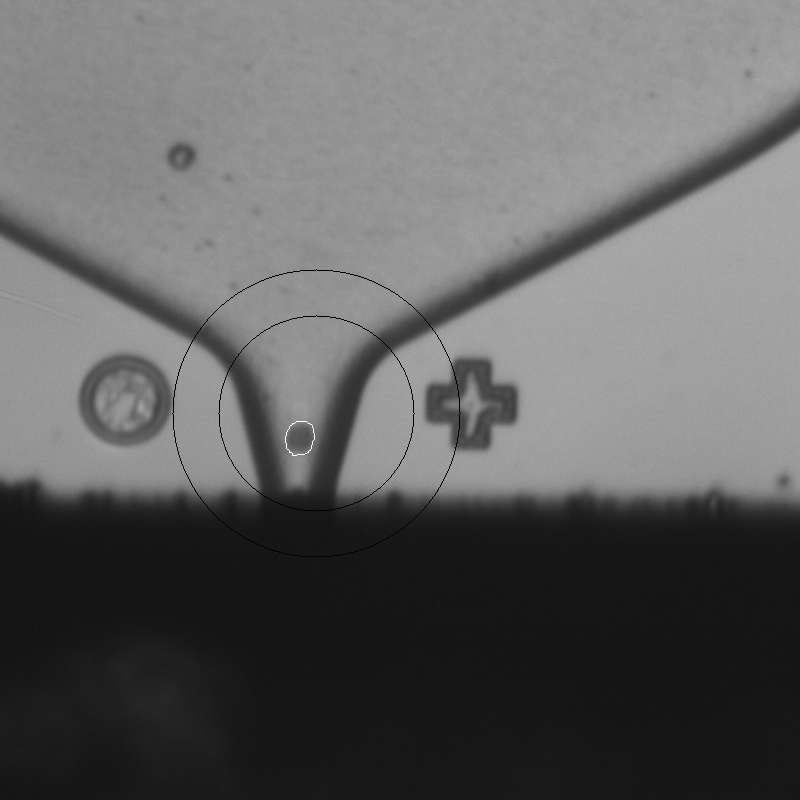

Supplement: Supplementary file 6 — Supplementary Data 3 [file 42003_2021_1661_MOESM6_ESM.zip › Supplementary Data 3 corrected/O_03_D.jpg]

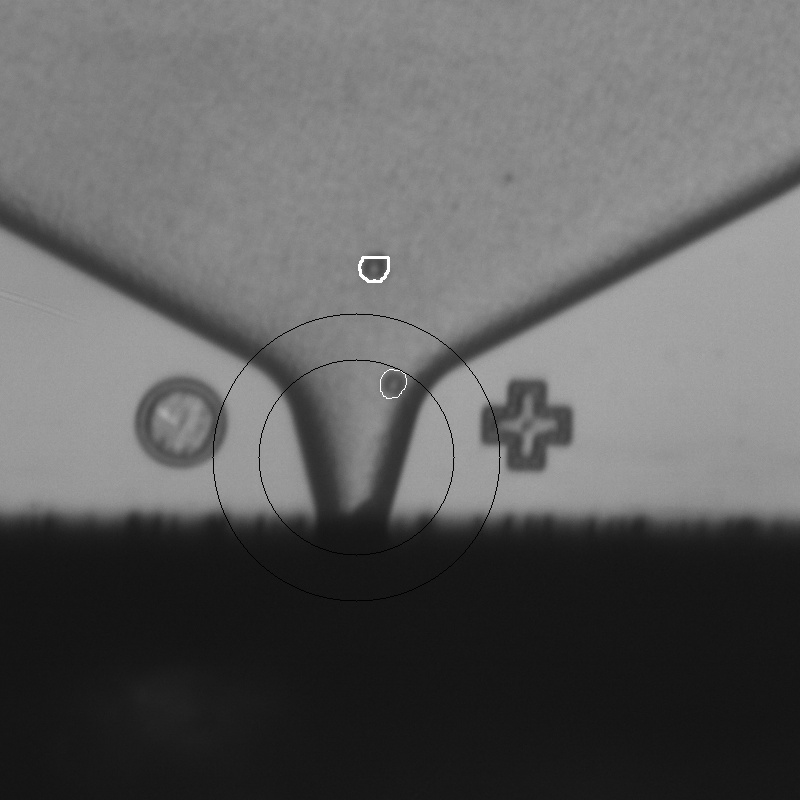

Supplement: Supplementary file 6 — Supplementary Data 3 [file 42003_2021_1661_MOESM6_ESM.zip › Supplementary Data 3 corrected/K_10_D.jpg]
