# Supplementary material for: Bisulfite-free epigenomics and genomics of single cells through methylation-sensitive restriction
Source: Commun Biol. 2021 Feb 1;4:153. doi: 10.1038/s42003-021-01661-w (PMC7851132; doi:10.1038/s42003-021-01661-w)
Supplement: Supplementary file 7 — Reporting Summary [file 42003_2021_1661_MOESM7_ESM.pdf]

nature research

Corresponding author(s): Heiko Becker

Last updated by author(s): Oct 16, 2020

Reporting Summary

Nature Research wishes to improve the reproducibility of the work that we publish. This form provides structure for consistency and transparency in reporting. For further information on Nature Research policies, see Authors & Referees and the Editorial Policy Checklist.

Statistics

For all statistical analyses, confirm that the following items are present in the figure legend, table legend, main text, or Methods section.

n/a	Confirmed	
☐	☒	The exact sample size (*n*) for each experimental group/condition, given as a discrete number and unit of measurement
☐	☒	A statement on whether measurements were taken from distinct samples or whether the same sample was measured repeatedly
☒	☐	The statistical test(s) used AND whether they are one- or two-sided<br>*Only common tests should be described solely by name; describe more complex techniques in the Methods section.*
☒	☐	A description of all covariates tested
☒	☐	A description of any assumptions or corrections, such as tests of normality and adjustment for multiple comparisons
☒	☐	A full description of the statistical parameters including central tendency (e.g. means) or other basic estimates (e.g. regression coefficient) AND variation (e.g. standard deviation) or associated estimates of uncertainty (e.g. confidence intervals)
☒	☐	For null hypothesis testing, the test statistic (e.g. *F*, *t*, *r*) with confidence intervals, effect sizes, degrees of freedom and *P* value noted<br>*Give P values as exact values whenever suitable.*
☒	☐	For Bayesian analysis, information on the choice of priors and Markov chain Monte Carlo settings
☒	☐	For hierarchical and complex designs, identification of the appropriate level for tests and full reporting of outcomes
☐	☒	Estimates of effect sizes (e.g. Cohen's *d*, Pearson's *r*), indicating how they were calculated

Our web collection on statistics for biologists contains articles on many of the points above.

Software and code

Policy information about availability of computer code

Data collection	Libraries were sequenced on an Illumina HiSeq 2000 machine using 125 bp paired-end mode (K_01 - K_07) or on a Illumina NovaSeq 6000 machine using 150 bp paired-end mode (K_08 - K27 and O_01 - O_20)
Data analysis	For the extraction of DNA methylation from epi-gSCAR data, NGS reads were analyzed using a custom bioinformatic pipeline (which was automated using Snakemake (version 5.3.0) in Python (version 3.6). After removal of Illumina adapters, overlapping paired-end reads were merged and non-overlapping reads were converted to singletons using BBMerge to obtain single-read information. Next, GAT-Adapter sequences were removed. The resulting pre-processed merged and unmerged reads were filtered for reads containing either 5´ poly(d)T or 3´ poly(d)A tailed HhaI scars separately (motif: GCGAAAAAA or TTTTTTCGC; Hamming distance = 1). Poly(d)T and poly(d)A tails were removed, resulting in reads containing 5´ or 3´ HhaI scars, respectively (5´-scar file and 3´-scar file). Separately, poly(d)T and poly(d)A tails were removed from GAT-Adapter trimmed reads (all-read file). All trimming and filtering steps were performed using BBDuk. Reads were subjected to quality control by FastQC and 5´ or 3´ HhaI scar-containing reads were mapped to the human assembly GRCh37 (hg19) with BWA-MEM separately with soft trimming enabled. Samtools was used to remove secondary and supplementary alignments and alignments with MAPQ smaller than 10. Alignment intervals were generated with the bamtobed command of bedtools and reduced to the outermost three 5´ or 3´ nucleotides, respectively. Scar intervals were filtered for nucleotide-precise overlap with HhaI sites and CpGs in the human genome and assigned as cut HhaI sites. In order to identify uncut (intact) HhaI sites, the all-read file was aligned accordingly, since all reads can potentially contain intact HhaI sites. Next, all HhaI sites of the human genome were expanded by 1 bp on either side as a safety margin and only completely covered intervals were assigned as uncut HhaI sites. Intact sites overlapping with cut HhaI sites (e.g. GCGCG(A)n-3') were excluded from the output since complete digestion of sites close to DNA ends cannot be guaranteed. Overlap of uncut with cut sites revealed sites of heterozygous methylation. All other uncut and cut HhaI sites were assigned as methylated or unmethylated, respectively. All CpG or HhaI sites of the human genome, which were covered by WGBS datasets of Kasumi-1 or OCI-AML3, respectively, were defined as informative for epi-gSCAR datasets.

For manuscripts utilizing custom algorithms or software that are central to the research but not yet described in published literature, software must be made available to editors/reviewers. We strongly encourage code deposition in a community repository (e.g. GitHub). See the Nature Research guidelines for submitting code & software for further information.

Data

Policy information about availability of data

All manuscripts must include a data availability statement. This statement should provide the following information, where applicable:

- Accession codes, unique identifiers, or web links for publicly available datasets
- A list of figures that have associated raw data
- A description of any restrictions on data availability

All sequencing data and processed single-cell DNA methylation data have been deposited in the Gene Expression Omnibus (GEO) database under accession GSE131723. Publicly available ChIP-seq and RNA-seq data sets used in this study were obtained from the GEO data portal with the following accessions: GSE29225, GSE62847, GSE83660, GSM1844449, GSM1844483, GSM3024903, GSM3032904, GSM3024909, GSM3032912. MALBAC and MDA datasets were obtained from the European Nucleotide Archive (SRS2062840) and the Sequence Read Archive (SRR617646 and SRR5219394). Publicly available Human SNP Array 6.0 array data was downloaded from the GEO (GSM888549). Kasumi-1 Human SNP Array 6.0 array data is available at EMBL-EBI ArrayExpress (E-MTAB-4950).

Figures with associated raw data:
Figure 1 d,e
Figure 2 a,b
Figure 3 a-g
Supplementary Figure 4
Supplementary Figure 6
Supplementary Figure 7
Supplementary Figure 8
Supplementary Figure 9
Supplementary Figure 10
Supplementary Figure 11

Field-specific reporting

Please select the one below that is the best fit for your research. If you are not sure, read the appropriate sections before making your selection.

☒ Life sciences ☐ Behavioural & social sciences ☐ Ecological, evolutionary & environmental sciences

For a reference copy of the document with all sections, see nature.com/documents/nr-reporting-summary-flat.pdf

Life sciences study design

All studies must disclose on these points even when the disclosure is negative.

Sample size	No sample size calculation was performed
Data exclusions	After sample processing and sequencing, one sample was found to originate from two cells as visually confirmed. This sample was excluded.
Replication	The paper describes the development of a method to analyze single cells - each cell represents a replication itself.
Randomization	The paper describes the development of a method - randomization is not applicable for this purpose.
Blinding	The paper describes the development of a method - blinding is not applicable for this purpose.

Reporting for specific materials, systems and methods

We require information from authors about some types of materials, experimental systems and methods used in many studies. Here, indicate whether each material, system or method listed is relevant to your study. If you are not sure if a list item applies to your research, read the appropriate section before selecting a response.

Materials & experimental systems

n/a	Involved in the study	
☒	☐	Antibodies
☐	☒	Eukaryotic cell lines
☒	☐	Palaeontology
☒	☐	Animals and other organisms
☒	☐	Human research participants
☒	☐	Clinical data

Methods

n/a	Involved in the study	
☒	☐	ChIP-seq
☒	☐	Flow cytometry
☒	☐	MRI-based neuroimaging

Eukaryotic cell lines

Policy information about cell lines

Cell line source(s)	The AML-derived cell lines Kasumi-1 and OCI-AML3 were provided by the research group of Michael Lübbert (University of Freiburg) who obtained it from DSMZ (No. ACC 220 and ACC 582; Braunschweig, Germany).
Authentication	The cell lines were obtained from repositories.
Mycoplasma contamination	Kasumi-1 and OCI-AML3 were tested negative for mycoplasma contamination.
Commonly misidentified lines (See ICLAC register)	No commonly misidentified cell line was used in this study.
